# Rare Earth Starting Materials and Methodologies for Synthetic Chemistry

**DOI:** 10.1021/acs.chemrev.1c00842

**Published:** 2022-01-31

**Authors:** Fabrizio Ortu

**Affiliations:** School of Chemistry, University of Leicester, LE1 7RH Leicester, U.K.

## Abstract

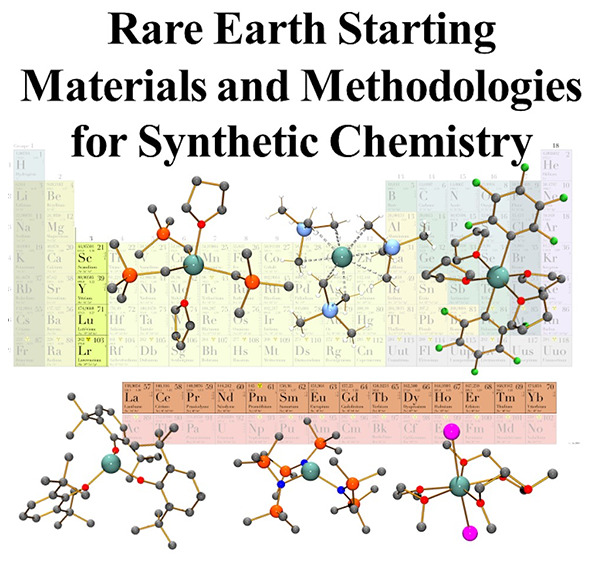

The number of rare earth (RE) starting materials used in synthesis is staggering, ranging from simple binary metal-halide salts to borohydrides and “designer reagents” such as alkyl and organoaluminate complexes. This review collates the most important starting materials used in RE synthetic chemistry, including essential information on their preparations and uses in modern synthetic methodologies. The review is divided by starting material category and supporting ligands (*i.e.*, metals as synthetic precursors, halides, borohydrides, nitrogen donors, oxygen donors, triflates, and organometallic reagents), and in each section relevant synthetic methodologies and applications are discussed.

## 1 Introduction

### 1.1 Motivation for the Review

The organometallic and coordination chemistry of the rare earth (RE) and lanthanoid (Ln) metals (Sc, Y, La, and Ce–Lu) was overshadowed for the best part of the 20th century by the popularity and wide applicability of transition-metal (TM) complexes. This historical disparity finds its roots in how research into the chemistry of REs and TMs developed through the centuries. While the first TM organometallic complexes were identified in the 19th century by pioneers of the ilk of Bunsen, Frankland, and Mond, attempts to stabilize RE–C bonds were largely unsuccessful until the 1950s.^1^ Finally, in 1954 Wilkinson and Birmingham succeeded in the stabilization of the first RE organometallic complexes, RE(Cp)_3_ (RE = Sc, Y, La, Ce, Pr, Nd, Sm, and Gd; Cp = {C_5_H_5_}^−^),^2^ over a century after Frankland’s preparation of dialkylzinc species.^3^ Following Wilkinson and Birmingham’s breakthrough, scientists started closing this historical 100 year gap with TM chemistry, and since then, RE organometallic and coordination chemistry has developed from mere curiosity to a burgeoning research field.^4−9^ A key component of these discoveries is the use of ligand architectures that can fulfill the electronic and steric demands of the large and electropositive RE ions. This step-change in RE chemistry was also propelled by the many advances in anaerobic manipulation techniques and ligand design, which in turn led to discoveries that challenged common assumptions and opened unexpected research avenues.^7,10^

Crucially, these advances have largely been supported by the preparation of adequate starting materials for anaerobic synthesis and the development of innovative synthetic strategies. In particular, the number of RE starting materials is staggering, constituting an immense library that synthetic chemists can access to design their own unique methodologies. All this essential information is spread over 100 years’ worth of literature and laboratory work, carried out by a huge number of researchers across the whole world. It is not uncommon for different research teams to devise their own unique methodologies and synthetic strategies, the nuances of which are often passed on as word of mouth—sometimes constituting a religious rite of passage for new starters. This review work provides a systematic account of the most important synthons used in RE synthetic chemistry, covering their preparations and applications as synthetic precursors. Despite the publication of several reviews and book chapters on many of the topics included in this work (*vide infra*, section 1.3), the last comprehensive account on the preparation of RE starting materials and their application in synthesis was provided by Edelmann in 1997.^9^ Since then the field has seen an immense amount of progress and the number of new starting materials at the disposal of synthetic RE chemists has increased significantly. The aim is to compile an up-to-date account of the most important synthons and methodologies that constitute the synthetic toolbox for any researcher approaching RE chemistry, thus providing an essential point of reference especially for those at the beginning of their journey into the f-block world.

### 1.2 Scope of the Review

Throughout the review the terms “rare earth” and “lanthanoid” will be used. The term “rare earth” will be used to describe all group 3 and lanthanoid elements, with the exception of the highly radioactive and rare promethium. The abbreviation “RE” in chemical formulas is used to describe group 3 metals (Sc and Y) and lanthanoids (La–Lu) collectively, whereas the abbreviation Ln will be used whenever a specific reaction or methodology applies exclusively to La–Lu or divalent species.

The aim of this review is to illustrate essential aspects of synthetic methods applied to RE chemistry, focusing primarily on the preparation of starting materials and their most important applications as synthetic precursors. In order to make this work more focused, the starting materials and methods described in this review will primarily relate to anaerobic synthesis. The review is subdivided into starting material categories, and dedicated synthetic methods are discussed within each section: metals as synthetic precursors (section 3), halides (section 4), borohydrides (section 5), amides (section 6), oxygen donors (section 7), triflates (section 8), and organometallics (section 9). With the exception of section 3, each section contains tables listing selected starting materials for each individual category of this review. In the case of organometallic reagents (section 9), Zimmermann and Anwander published a very comprehensive account in 2010,^11^ so this review will provide a broad overview of the topic and illustrate the progress achieved over the past decade. This review work covers all relevant literature published up to December 2021.

### 1.3 Previous Reviews

Over the last 70 years a significant number of reviews have been written on the individual categories covered herein. Selected reviews which are relevant for this work are listed below:

- *Metals as synthetic precursors*:^12,13^
  - Guo, Z.; Huo, R.; Tan, Y. Q.; Blair, V.; Deacon, G. B.; Junk, P. C. Synthesis of Reactive Rare Earth Complexes by Redox Transmetalation/Protolysis Reactions-A Simple and Convenient Method; 2020 (ref (12))
  - Cloke, F. G. N. Zero Oxidation State Compounds of Scandium, Yttrium, and the Lanthanides; 1993 (ref (13))
- *Halides:*(14,15)
  - Taylor, M. D. Preparation of Anhydrous Lanthanon Halides; 1962 (ref (14))
  - Meyer, G. The Rare Earth Elements – The Divalent State in Solid Rare Earth Metal Halides; 2012 (ref (15))
- *Borohydrides:*(16−21)
  - Marks, T. J.; Kolb, J. R. Covalent Transition Metal, Lanthanide, and Actinide Tetrahydroborate Complexes; 1977 (ref (16))
  - Ephritikhine, M. Synthesis, Structure, and Reactions of Hydride, Borohydride, and Aluminohydride Compounds of the f-Elements; 1997 (ref (17))
  - Makhaev, V. D. Structural and Dynamic Properties of Tetrahydroborate Complexes; 2000 (ref (18))
  - Visseaux, M.; Bonnet, F. Borohydride Complexes of Rare Earths, and Their Applications in Various Organic Transformations; 2011 (ref (19))
  - Guillaume, S. M.; Maron, L.; Roesky, P. W. Catalytic Behavior of Rare-Earth Borohydride Complexes in Polymerization of Polar Monomers; 2014 (ref (20))
  - Paskevicius, M.; Jepsen, L. H.; Schouwink, P.; Černý, R.; Ravnsbæk, D. B.; Filinchuk, Y.; Dornheim, M.; Besenbacher, F.; Jensen, T. R. Metal Borohydrides and Derivatives–Synthesis, Structure and Properties; 2017 (ref (21))
- *Amides:*(22−24)
  - Anwander, R. Lanthanide Amides; 2005 (ref (22))
  - Lappert, M. F.; Protchenko, A.; Power, P. P.; Seeber, A. Metal Amide Chemistry; 2009 (ref (23))
  - Goodwin, C. A. P.; Mills, D. P. Silylamides: Toward a Half-Century of Stabilizing Remarkable f-Element Chemistry; 2017 (ref (24))
- *Oxygen donors:*(25−30)
  - Bradley, D. C.; Mehrotram R. C.; Gaurm D. P. Metal Akoxides; 1978 (ref (25))
  - Mehrotra, R. C.; Singh, A.; Tripathi, U. M. Recent Advances in Alkoxo and Aryloxo Chemistry of Scandium, Yttrium and Lanthanides; 1991 (ref (26))
  - Bradley, D. C.; Mehrotra, R. C.; Rothwell, I. P.; Singh, A. Alkoxo and Aryloxo Derivatives of Metals; 2001 (ref (27))
  - Anwander, R. Routes to Monomeric Lanthanide Alkoxides; 2005 (ref (28))
  - Boyle, T. J.; Ottley, L. A. M. Advances in Structurally Characterized Lanthanide Alkoxide, Aryloxide and Silyloxide Compounds; 2008 (ref (29))
  - Parmar, V.; Mills, D. P.; Winpenny, R. E. P. Mononuclear Dysprosium Alkoxide and Aryloxide Single-Molecule Magnets; 2021 (ref (30))
- *Triflates:*(31)
  - Lawrance, G. A. Coordinated Trifluoromethanesulfonate and Fluorosulfate; 1986 (ref (31))
- *Organometallics:*(4,5,11,32−38)
  - Cotton, F. A. Alkyls and Aryls of Transition Metals; 1955 (ref (4))
  - Davidson, P. J.; Lappert, M. F.; Pearce, R. Metal σ-Hydrocarbyls, MR_*n*_. Stoichiometry, Structures, Stabilities, and Thermal Decomposition Pathways; 1976 (ref (32))
  - Bochkarev, M. N.; Kalinina, G. S.; Bochkarev, L. N. Advances in the Chemistry of Organolanthanides; 1985 (ref (33))
  - Schumann, H.; Meese-Marktscheffel, J. A.; Esser, L. Synthesis, Structure, and Reactivity of Organometallic π-Complexes of the Rare Earths in the Oxidation State Ln^3+^ with Aromatic Ligands; 1995 (ref (34))
  - Cotton, S. A. Aspects of the Lanthanide-Carbon σ-Bond; 1997 (ref (5))
  - Deacon, G. B.; Forsyth, C. M.; Nickel, S. Bis(pentafluorophenyl)mercury–a versatile synthon in organo-, organooxo-, and organoimido-lanthanoid chemistry; 2002 (ref (35))
  - Edelmann, F. T.; Freckmann, D. M. M.; Schumann, H. Synthesis and Structural Chemistry of Non-Cyclopentadienyl Organolanthanide Complexes; 2002 (ref (36))
  - Zimmermann, M.; Anwander, R. Homoleptic Rare-Earth Metal Complexes Containing Ln–C σ-Bonds; 2010 (ref (11))
  - Lyubov, D.; Trifonov, A. A. Ln(II) Alkyl Complexes: From Elusive Exotics to Catalytic Applications; 2021 (ref (37))
  - Izod, K. Alkyl, Carbonyl and Cyanide Complexes of the Group 3 Metals and Lanthanides; 2021 (ref (38))

A significant number of reviews have also been published on general synthetic methods in RE chemistry. Some of the most relevant for this work are listed below:^6,8,39−43^

- Evans, W. J. The Organometallic Chemistry of the Lanthanide Elements in Low Oxidation States; 1987 (ref (40))
- Edelmann, F. T. Scandium, Yttrium, and the Lanthanide and Actinide Elements, Excluding their Zero Oxidation State Complexes; 1995 (ref (6))
- Edelmann, F. T. Lanthanides and Actinides; 1997 (ref (9))
- Anwander, R. Principles in Organolanthanide Chemistry; 1999 (ref (8))
- Anwander, R. Herrmann, W. A. Features of Organolanthanide Complexes; 2005 (ref (39))
- Liddle, S. T. Lanthanides: Organometallic Chemistry Fundamental Properties; 2012 (ref (41))
- Nicholas, H. M.; Mills, D. P. Lanthanides: Divalent Organometallic Chemistry; 2017 (ref (42))
- Ortu, F.; Mills, D. P. Low Coordinate Rare Earth and Actinide Complexes; 2019 (ref (7))
- Layfield, R. A. Lanthanides; 2021 (ref (43))

## 2 General Considerations on the Reactivity of REs

Unlike TMs, RE metals are not involved in classic two-electron reactions like oxidative addition and reductive elimination. Therefore, synthetic strategies are mostly limited to a handful of key reactions: (1) *salt elimination*, (2) *metathesis*, (3) *protonolysis* (also referred to as *protolysis*), and (4) *insertion*/*oxidation*.^9,44^ This is not an exhaustive list, as other more specialized methodologies have been also implemented in RE chemistry and will be discussed in more detail in the various sections of this review.

### 2.1 Salt Elimination/Metathesis

In salt elimination/metathesis reactions, a RE salt precursor, LnX_2_ or REX_3_ (X = halide, borohydride, triflate, *etc*.), is reacted with a ligand transfer reagent M(L)_*n*_ (*n* = 1, 2 depending on the oxidation state of the metal and formal charge of the ligand), producing a new RE complex and eliminating an inorganic salt (eq 1). Classic ligand transfer reagents are group 1 and group 2 salts of a variety of bases, such as hydrocarbyls, amides, alkoxides, and aryloxides. Additionally, the methodology entails the elimination of highly insoluble inorganic salts with very high lattice energies, thus providing a strong driving force for the reaction. For these reasons, this is one of the most popular methodologies used in RE synthetic chemistry and can be applied to the synthesis of both homoleptic and heteroleptic species, though the occurrence of salt occlusion can prevent the isolation of clean products.^8,41^
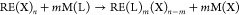
1

The availability of different halide sources offers several possibilities in terms of lattice energy and solubility of the byproducts of salt elimination reactions. Estimates of lattice energies (eq 2) can be obtained from simple electrostatic considerations:
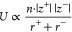
2where the lattice energy, *U*, is proportional to the number of ions (*n*) and their charges (*z*^+^ and *z*^–^) and inversely proportional to their ionic radii (*r*^+^ and *r*^–^), *i.e.,* the distance between the two charges.^45^ Smaller ions will give the highest lattice energy values (LiF, 1034 kJ mol^–1^), while larger ions will have the smallest lattice enthalpies (CsI, 613 kJ mol^–1^) (Table 1).^46^ Additionally, the relative strength of the RE–X bonds should also be taken into account: RE^3+^ ions are hard Lewis acids, and the bond strength with halides decreases descending the group. One additional consideration is the solubility of the alkali metal salts. Often salt elimination reactions have to be carried out in ethereal solvents because of the poor solubility of starting materials and reagents. However, alkali metal salt can have some solubility in certain organic solvents, which can have a detrimental effect on the outcome of salt elimination protocols and make purification procedures extremely challenging (Table 1).

**Table 1:** Lattice Energies (*U*)^46^ and Solubilities of Selected Alkali Metal Halides in THF at 25 °C^47−49^

	*U* (kJ mol^–1^)	solubility (mg/mL)
LiF	1034	–
LiCl	864	49.5
LiBr	820	388
LiI	764	552^a^
NaCl	790	0.20
NaBr	754	0.15
NaI	705	29.98
KCl	720	0.30
KBr	691	0.06–0.1
KI	650	0.001

a Solubility in diethyl ether.^50^

### 2.2 Metathesis

Metathesis reactions produce a ligand exchange between two metals (eq 3), based on differences in their affinities for different donors. For example, this is a method that can be employed for the preparation of RE organometallics complexes (*i.e.*, transmetalation), such as the metathesis reaction between aryloxide complexes and Li{CH(SiMe_3_)_2_} to give [RE{CH(SiMe_3_)_2_}_3_].^51^ This method is particularly useful for the preparation of homoleptic complexes, as the occurrence of ligand scrambling often prevents the isolation of heteroleptic species.
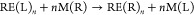
3

### 2.3 Protonolysis/Protolysis

RE starting materials can perform acid/base reactions with protic substrates, accompanied by the highly favorable elimination of volatile gases or liquids (eq 4). Such reactions are classically driven by differences in p*K*_a_ (Table 2), though other important factors can come into play (*e.g.*, saturation of metal coordination sphere or use of chelating ligands). However, simple acid/base considerations are often a good indication when planning a methodology that makes use of these reactions.
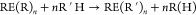
4

**Table 2:** p*K*_a_ Values of Selected Acids in H_2_O^7,11,52^

acid	p*K*_a_
CH_4_	48
Ph_3_CH	31.5
C_5_H_2_Me_4_	26.1^a^
HN(SiMe_3_)_2_	25.8
HN(SiHMe_2_)_2_	22.6
C_5_H_6_	15
HOC_6_H_3_Me_3_-2,4,6	10.87
HOC_6_H_6_	9.97
HN(Me)(C_6_H_5_)	4.85
H_2_NC_6_H_5_	4.58

a Value in DMSO.

### 2.4 Insertion/Oxidation

RE metals can sometimes react directly with substrates, either by inserting into a polar bond (*e.g.*, a carbon–halogen bond, eq 5) or by reducing the target donor (eq 6). The former method has been used very rarely, and it mainly applies to divalent REs that are able to behave like *pseudo*-alkaline earth (AE) metals (section 3.2). Oxidation reactions instead are more versatile, though their main applications are limited to Sm, Eu, and Yb; these are usually performed on protic substrates and accompanied by the elimination of hydrogen (sections 3.1 and 3.3). Both insertion and oxidation reactions can be sluggish with RE metals, and better results are often achieved when metals have been activated. Oxidation reactions have also been combined with transmetalation reactions by using redox transmetalating reagents, such as organomercurials (section 3.4).
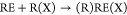
5
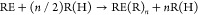
6

## 3 Metals as Synthetic Precursors

The use of REs in their metallic form as primary starting materials for synthesis has been investigated for over a century. For example, high-temperature reaction of RE powders with dry HCl or iodine is an established methodology for the preparation of solvent-free RECl_3_, and most REs react readily with iodine or 1,2-dioodoethane in ethereal solvents to afford di- and trihalide species (*vide infra*, section 4.1). REs can also react directly with organic substrates (section 3.3), though often activation strategies have to be employed which circumvent the relative inertia of the metal. Examples of this approach are (1) activation in liquid ammonia (section 3.1), (2) activation with Hg or HgCl_2_ (section 3.3), (3) redox transmetalation (section 3.4), and (4) metal vapor synthesis (section 3.5). Additionally, in some cases REs can also react directly with substrates in a fashion similar to the insertion of Mg into C–X bonds (section 3.2).

### 3.1 Activation in Liquid Ammonia

**Scheme 1:**
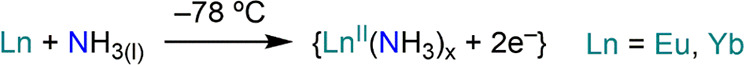
Synthesis of Ammonia Solution of Divalent Lns

**Figure 1:**
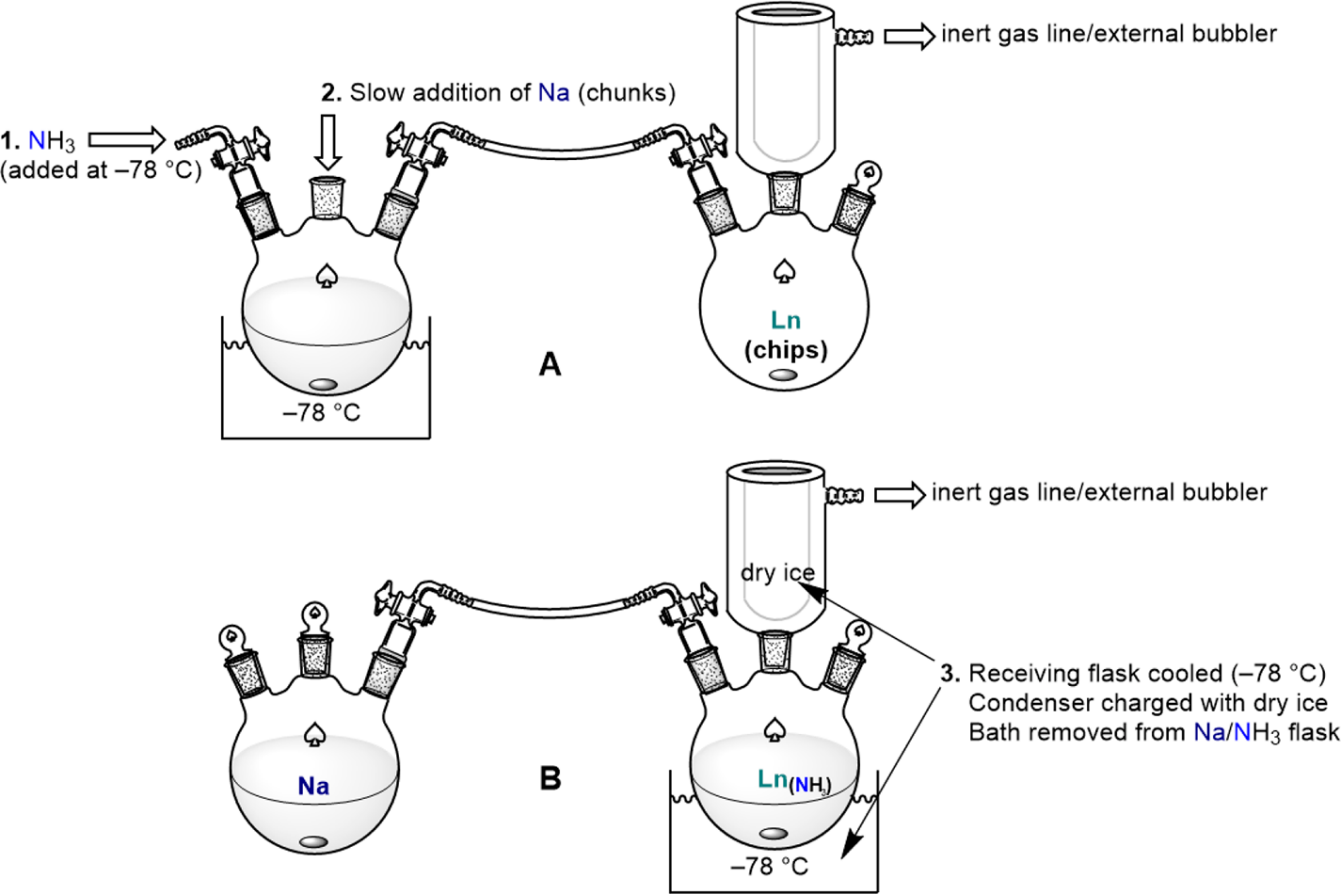
Illustration of basic setup for the preparation of ammoniacal solutions of divalent Lns. Alternatively, modification of Schlenk line manifolds can be implemented to optimize ammonia transfer and workup procedures.^55^

At the turn of the 20th century, the use of liquid ammonia as an alternative nonaqueous solvent sparked the interest of the scientific community,^53^ leading to the development of new reaction apparatuses and experimental techniques which set criteria still used today for experimental work with ammoniacal solutions (Figure 1).^54,55^ Pioneering work by Joannis,^53^ Mentrel,^56^ Roederer,^57^ and Moissan^58−60^ focused on the solubilization of alkali and AE metals and the properties of resulting solutions. Kraus further developed this new research field by studying various s-block metals in liquid ammonia and first proposed the presence of solvated electrons is such media.^61−63^ Around the same time, Cottrel focused his efforts on the dissolution of Mg and Ca and their reactivity with acetylene.^64^ The first ammoniacal solutions of any f-element were first reported by Watt *et al.* in 1950, who described the use of liquid ammonia on various U and Th salts (*e.g.*, iodates, oxalates, peroxides).^65^ Warf and Korst decided to extend this study to the classic divalent Lns, owing to their resemblance to the heavy AE elements.^66^ The heavy AEs share many similarities with classic divalent Lns Eu, Sm, and Yb, such as the stability of the +2 oxidation state and ionic radii (Ca^2+^, 1.00 Å; Yb^2+^, 1.02 Å; Sm^2+^,1.17 Å; Eu^2+^, 1.22 Å; Sr^2+^, 1.18 Å).^67^ Because of this their coordination chemistry has often been developed in parallel.^68^ By using freshly distilled ammonia, Warf and Korst were able to dissolve Eu and Yb at −78 °C, while no reaction was observed with Sm (Scheme 1 and Figure 1).

The nature of the cation–electron ion pair was further corroborated by EPR studies on Eu ammoniacal solutions performed by Catterall and Symons in 1964;^69,70^ additionally, the electronic spectra of Eu and Yb species were recorded a year later by Waugh and co-workers.^71^ Interestingly, Peer and Lagowski were able to dissolve various other Lns (Sm, Tb, Er, and Tm) in ammonia *via* co-condensation and recorded the electronic spectra of the resulting solutions; both Sm and Tb were identified to be in their divalent state, while definitive conclusions could not be drawn for Er and Tm.^72^

**Scheme 2:**
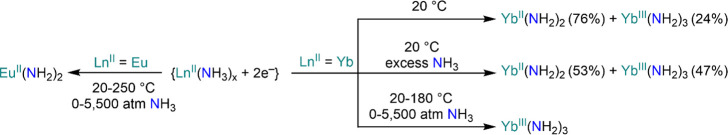
Decomposition of Ammoniacal Solutions of Eu and Yb and Formation of Divalent and Trivalent Amides Eu(NH_2_)_2_, Yb(NH_2_)_2_, and Yb(NH_2_)_3_^73^

Eu and Yb ammonia solutions are relatively stable when stored under anaerobic conditions. However, they decompose over time to generate the parent amide Ln(NH_2_)_2_, similar to what is observed with alkali and AE metals (Scheme 2).^73^ In the case of Yb, depending on reaction conditions, degradation of ammoniacal solutions can also produce oxidized trivalent amide Yb(NH_2_)_3_.^73^ Divalent Eu(NH_2_)_2_ is obtained also under ammonothermal reaction conditions (up to 5000 atm of NH_3_), while for Yb the same reaction conditions generate pure trivalent Yb amide or the salt Na[Yb(NH_2_)_4_].^74^ The use of ammonothermal conditions with Lns has limited synthetic utility and has attracted interest mostly for the preparation of new semiconductors;^75^ also, supercritical ammonia (160 °C) has been used to prepare metal sulfide salts of Yb, [Yb(NH_3_)_8_][M(S_4_)_2_]·NH_3_ (M = Cu, Ag), and La, [La(NH_3_)_9_][Cu(S_4_)_2_].^76^ Additionally, Müller-Buschbaum and Quitmann reported the molecular structure of complex [Sm(NH_3_)_9_][Sm(Pyr)_6_] (Pyr = pyrrolide, {C_4_H_4_N}^−^), obtained from the direct reaction of pyrrole with Sm metal and pyrrole under solvothermal conditions, followed by treatment with liquid ammonia.^77^ Recently, Kraus and co-workers investigated the preparation of Eu(II), Yb(II), and Ho(III) azides by reacting the pure metal with AgN_3_ in liquid ammonia, leading to the structural identification of ammino-adducts [Ho_2_(μ-NH_2_)_3_(NH_3_)_10_](N_3_)_3_·(NH_3_)_1.25_ and [Yb(NH_3_)_8_](N_3_)_2_.^78,79^

**Scheme 3:**
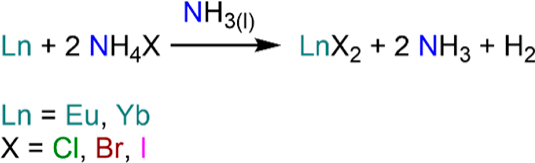
Synthesis of Eu and Yb Divalent Halides in Liquid NH_3_^81^

The ready availability of soluble forms of divalent Lns gives access to the preparation of simple binary species. Such was the observation of Salot and Warf in 1968, who postulated the formation of YbI_2_ from the reaction of Yb metal and NH_4_I in liquid NH_3_.^80^ Shortly after, Howell and Pytlewski followed a similar synthetic protocol and reported the preparation of six unsolvated LnX_2_ (Ln = Eu, Yb; X = Cl, Br, I) species (Scheme 3);^81^ this methodology has been used extensively by synthetic chemists since then (*vide infra* for preparation and uses of LnX_2_ precursors in synthesis, section 4.1).^82^ When Sm is employed, this methodology leads to the formation of trivalent species.^80^

**Scheme 4:**
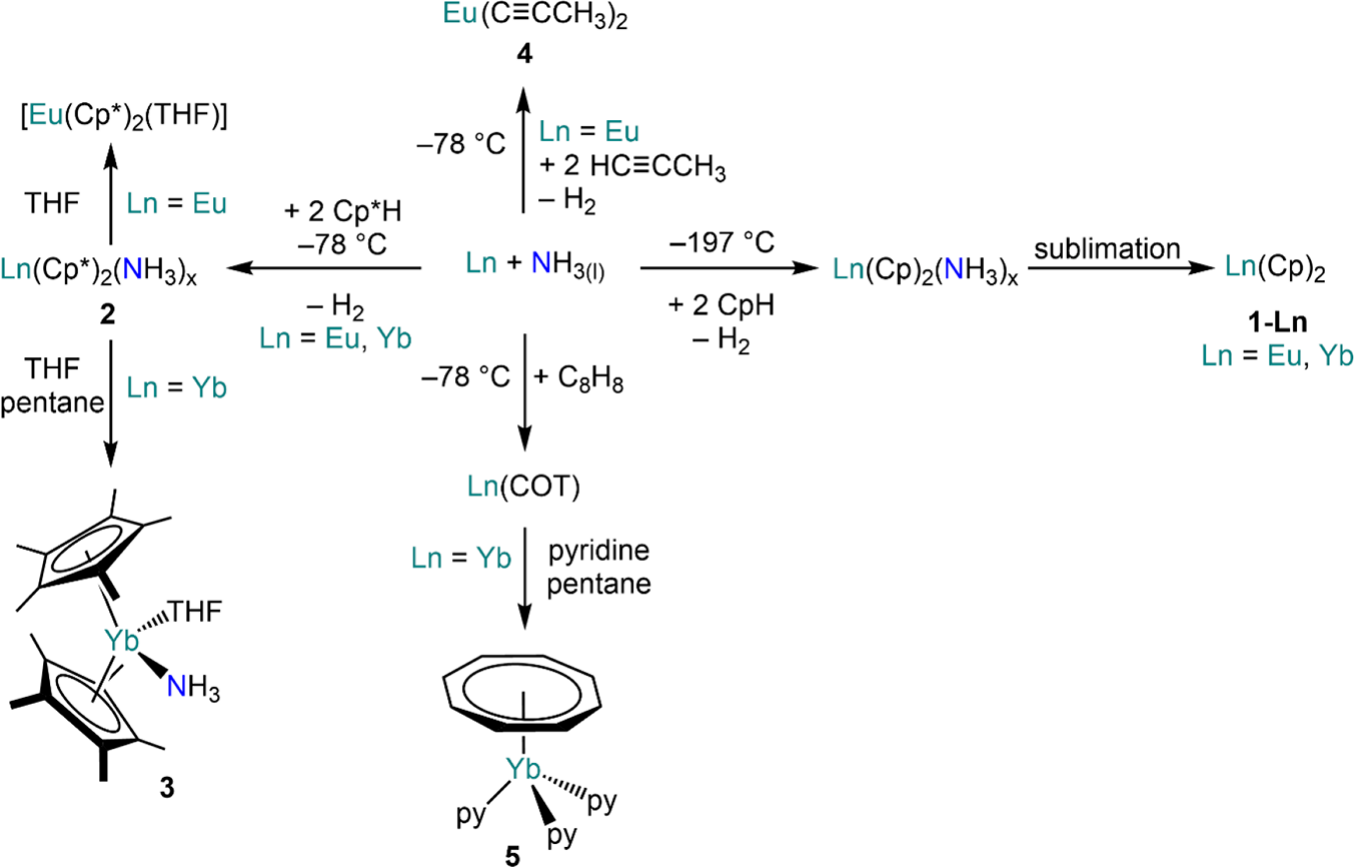
Synthesis of Divalent Ln Organometallic Complexes in Liquid NH_3_^83−87^

**Scheme 5:**
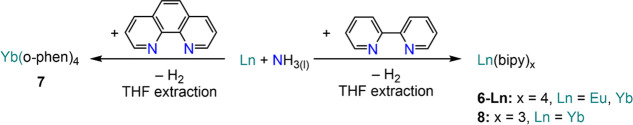
Reduction of 2,2′-Bipyridine and Phenantroline with Ln Ammoniacal Solutions^88,89^

Owing to the highly reducing nature of these ammoniacal Ln solutions, their synthetic utility was first explored in the 1960s. Fischer and Fischer reacted freshly distilled cyclopentadiene with an ammoniacal solution of europium or ytterbium, followed by sublimation to yield analytically pure metallocene Ln(Cp)_2_ (**1-Ln**; Ln = Eu, Yb; Cp = {C_5_H_5_}^−^) (Scheme 4).^83^ Similarly, Wayda *et al.* were able to synthesize their Cp* (Cp* = {C_5_Me_5_}^−^) analogues Ln(Cp*)_2_(NH_3_)_*x*_ (**2**), though only the ammoniate metallocene derivative [Yb(Cp*)_2_(NH_3_)(THF)] (**3**) was structurally authenticated (Scheme 4).^84^ A similar approach has also been used for the preparation of other organometallic derivatives, such as Ln(COT) (COT = {C_8_H_8_}^−^)^85^ and Eu(II) propynide Eu(C≡CCH_3_)_2_ (**4**)_._^86^ Wayda *et al.* followed the original Ln(COT) synthesis developed by Thomas and Hayes^85^ and were able to crystallize the Yb derivative by slow diffusion of pentane in a pyridine solution, thus obtaining the piano-stool complex [Yb(COT)(py)_3_] (**5**·3py, py = pyridine).^87^ Around the same time of Fischer and Fischer’s metallocene synthesis, Ln ammoniacal solutions were also used to for the reduction of 2,2′-bipyridine and phenantroline (Scheme 5), leading to the isolation of Ln(bipy)_4_ (**6-Ln**; Ln = Eu, Yb; bipy = 2,2′-bipyridine) and Yb(phen)_4_ (**7**, phen = phenantroline);^88^ Pappalardo also reported the preparation of Yb(bipy)_3_ (**8**) using a similar methodology.^89^

**Scheme 6:**
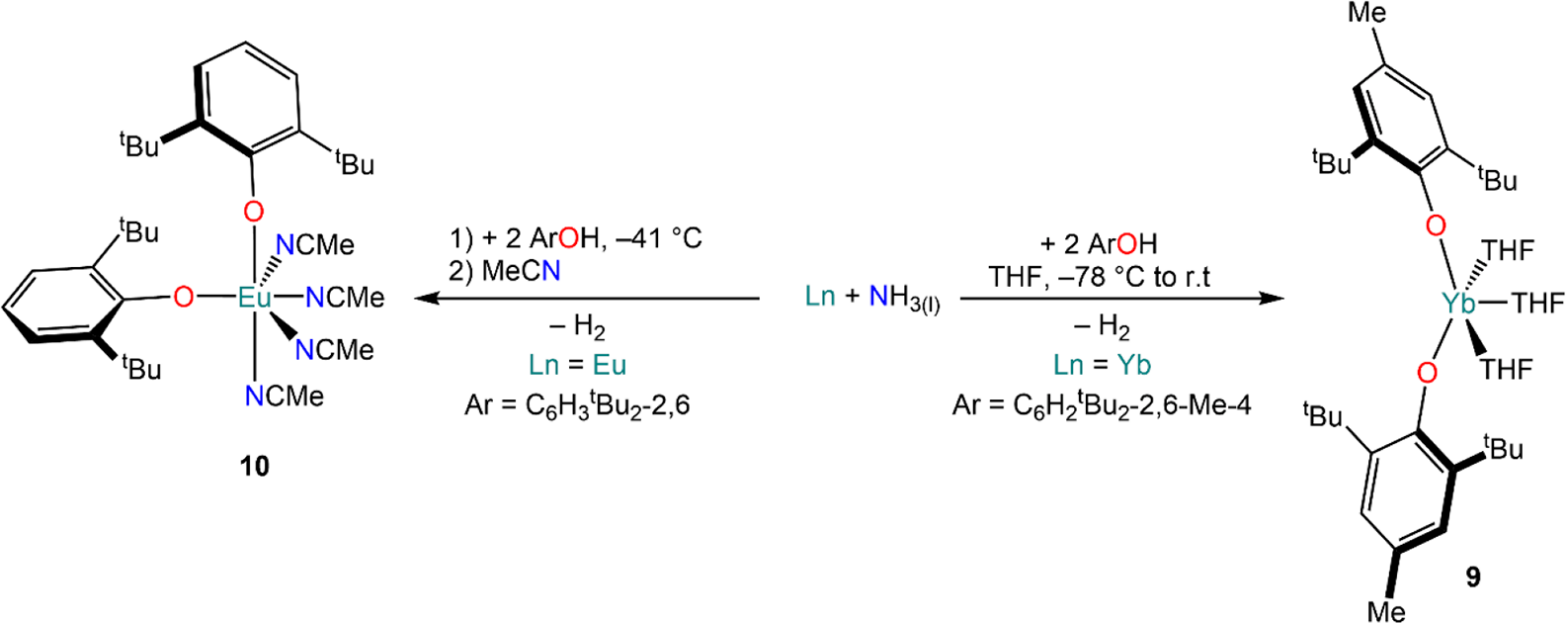
Synthesis of Ln(II) Aryloxide Complexes **9** and **10** from Ln Ammoniacal Solutions^90,92^

Smith and co-workers also used Yb ammoniacal solutions to prepare the bis-aryloxide Yb complex [Yb(ODbmp)_2_(THF)_3_] (**9**, Scheme 6) (ODbmp = OC_6_H_2_^t^Bu_2_-2,6-Me-4),^90^ which was originally prepared *via* protonolysis or redox transmetalation (*vide infra*, sections 3.4 and 7.1).^91^ Similarly, Evans *et al.* isolated the Eu^2+^ complex [Eu(ODb)_2_(NCMe)_4_] (**10**, ODb = OC_6_H_3_^t^Bu_2_-2,6) by dissolving a Eu ingot and 2,6-di-*tert*-butylphenol in liquid ammonia, followed by extractions with acetonitrile (Scheme 6).^92^ However, when 2,6-diisopropylphenol (HODipp) is employed, the hydroxo-bridged Eu(II) complex [Eu_4_(ODipp)_2_(μ-ODipp)_4_(μ_3_–OH)_2_(NCMe)_6_] is obtained; attempts by Evans *et al.* to use this methodology to obtain a hydroxo-free Eu(II) derivative were not successful.^92^

**Scheme 7:**
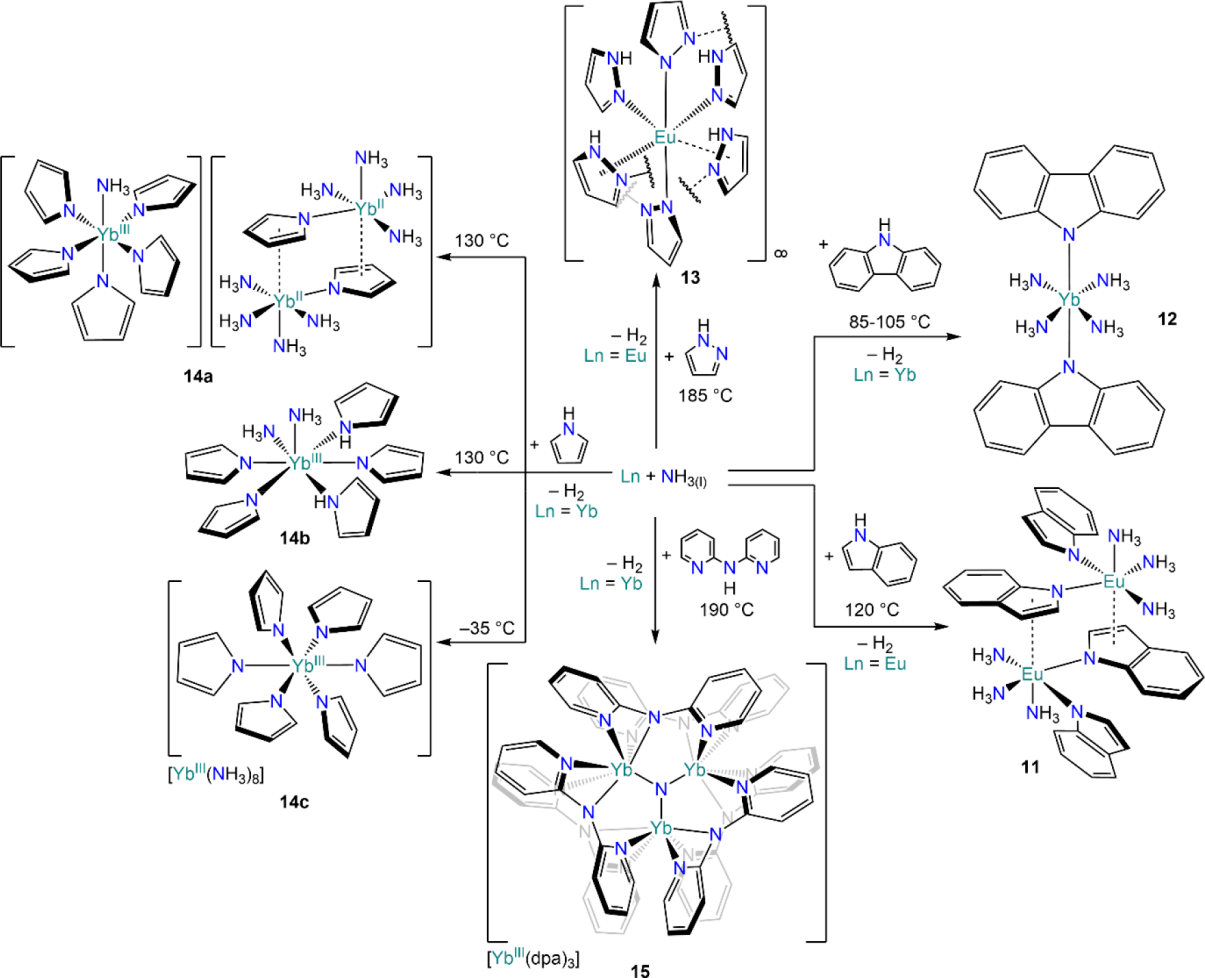
Reactivity of Yb and Eu with *N*-Heterocycles and Amines Using Ammoniacal Solutions or Ammonia-Activated Metals^77,93−98,100^

This synthetic approach has been revisited more recently by Müller-Buschbaum and co-workers, who have used similar methodologies for the activation of a variety of nitrogen-containing heterocycles, *e.g.*, indole (**11**), carbazole (**12**), pyrazole (**13**), and pyrrole (**14**) (Scheme 7).^77,93−98^ These reactions in some cases are accompanied by concomitant formation of {NH_2_}^−^ from ammonolysis.^95^ In their work, Müller-Buschbaum and co-workers carried out reactions in the absence of solvents and under solvothermal conditions, though in some cases low-temperature reactivity was also achieved, such as with the synthesis of [Yb(NH_3_)_8_][Yb(pyr)_6_] (**14c**, pyr = pyrrolide, {C_4_H_4_N}^−^).^97^ This approach is particularly interesting for modern RE synthetic chemistry, as it can provide an access route to very challenging complexes avoiding the use of coordinating solvents.^98^ In the case of the reaction between Yb and carbazole (C_12_H_8_NH, CarbH), *N-*phenylpiperazine (Phpip) was added to aid crystallization of the target complex [Yb(Carb)_2_(NH_3_)_4_] (**12**, Carb = carbazolide, {C_12_H_8_N}^−^), though this product cannot be separated from cocrystallized CarbH·Phpip.^94^ It is also noteworthy that Müller-Buschbaum and co-workers extended this work to various divalent and trivalent REs by reacting metal powders with amines, either directly or in the presence of trace amounts of Hg under solvothermal conditions (*vide infra*, sections 3.3 and 3.4).^77,99^ Remarkably, the use of ammoniacal Yb solutions in the presence of 2,2′-dipyridylamine afforded the isolation of the first RE molecular nitride [Yb_3_N(dpa)_6_][Yb(dpa)_3_] (**15**, dpa = 2,2′-dipyridylamide, {(C_6_H_4_N)_2_N}^−^).^100^

### 3.2 Insertion into C–X Bonds

**Scheme 8:**
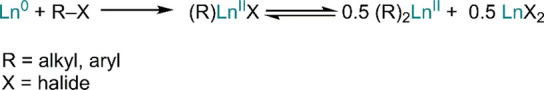
Insertion Reaction of Ln Metal into C–X Bonds and Schlenk-Type Equilibrium

Eu, Sm, and Yb resemble the chemistry of Sr and Ca respectively, owing in particular to the stability of the divalent state and ionic radii (see section 3.1). Organocalcium “heavy Grignard” reagents, (R)CaX (R = alkyl, aryl; X = halide), were originally investigated in the early 20th century as potential alternatives to classic organomagnesium analogues;^101−103^ additionally, several structurally authenticated examples have been reported by Westerhausen and co-workers over the last two decades,^104,105^ and recently this chemistry has also been extended to Sr and Ba.^106^ Consequently, classic divalent Lns (Eu, Sm and Yb) have also been investigated for the preparation of *pseudo-*Grignard reagents, *i.e.* (R)LnX (R = alkyl, aryl; X = halide).^107,108^ Unlike for Mg, heavy group 2 Grignard reagents readily undergo Schlenk-type degradation affording dialkyl and dihalide species;^104,105^ therefore, owing to the similarities of classic divalent Lns with the heavy AE metals, this type of degradation is also a likely occurrence for a putative (R)LnX *pseudo-*Grignard derivative (Scheme 8).

**Scheme 9:**
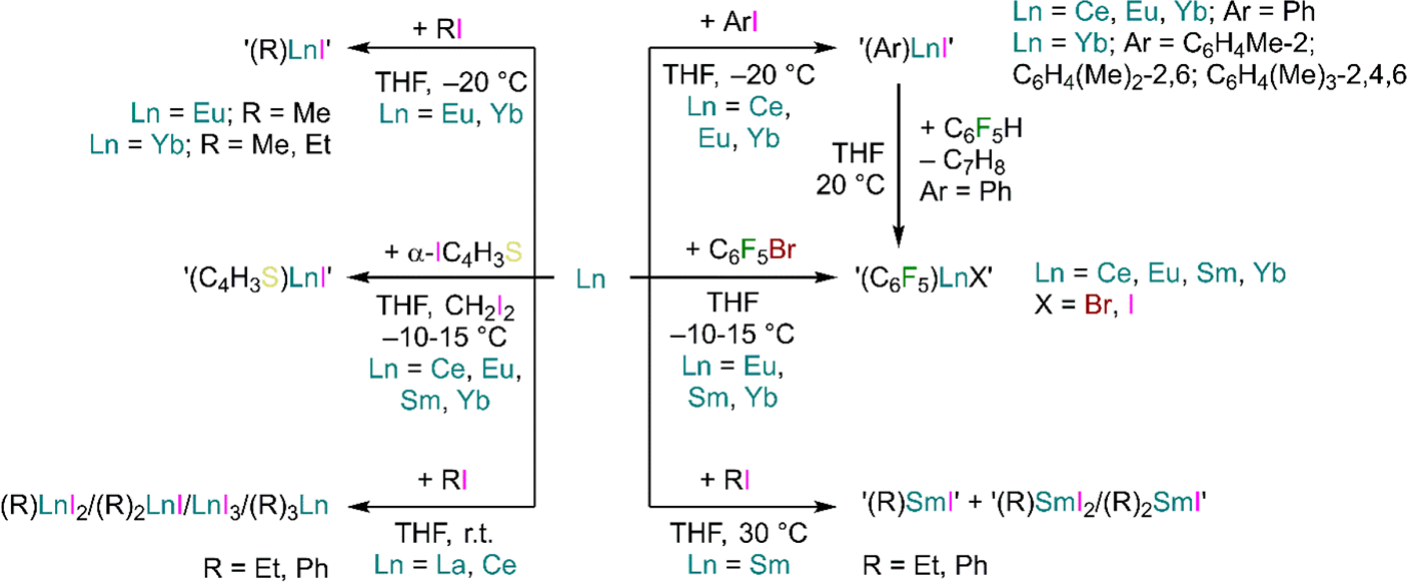
Synthesis of Ln *pseudo*-Grignard Derivatives by D. F. Evans,^107,108^ Beletskaya,^109^ and Co-workers

**Scheme 10:**
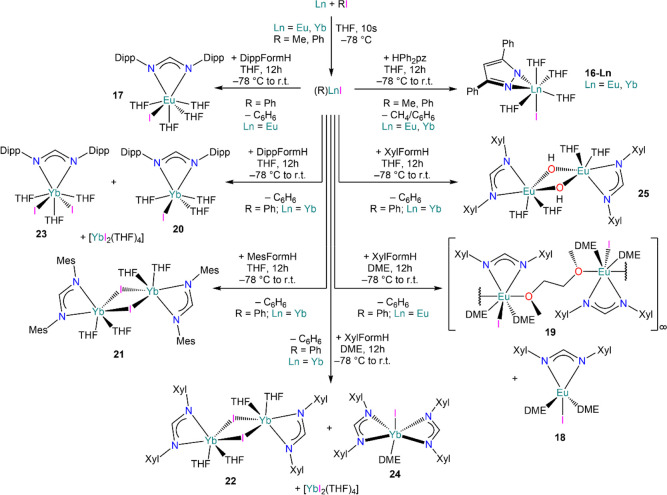
Synthesis of Ln Formamidinate Complexes *via* Protonolysis Using (Ph)LnI^112,113^

**Scheme 11:**
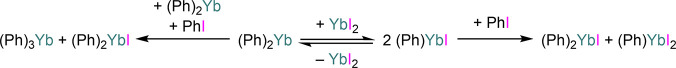
Schlenk-Type Equilibrium and Degradation of (Ph)YbI Proposed by Junk and Co-workers^113^

D. F. Evans *et al.* first reported the reaction of Yb and Eu metal with alkyl and aryl iodides in THF at −20 °C (Scheme 9).^107,108^ In the case of Yb, magnetic susceptibility measurements revealed the presence of small quantities of Yb^3+^ ion (Yb^3+^: [Xe]4*f*^13^, μ_eff_ = 4.5 μ_B_), with Yb^2+^ as the predominant ion (Yb^2+^: [Xe]4*f*^14^, μ_eff_ = 0 μ_B_);^108^ in the case of europium, the magnetic moment of (Ph)EuI was measured at 7.5 μ_B_, close to the value for free Eu^2+^ (Eu^2+^: [Xe]4*f*^7^_,_ μ_eff_ = 7.5 μ_B_; Eu^3+^: [Xe]4*f*^6^_,_ μ_eff_ = 1.5 μ_B_).^108^ In the case of Sm metal, reactivity with iodobenzene and iodoethane is sluggish, requiring higher temperatures compared to Eu and Yb and affording a much higher proportion of trivalent species.^108^ Moreover, further reactivity tests with Gd and Er showed no reactivity, while La and Ce are converted into mixtures of trivalent species, *i.e.*, (R)REI_2_, (R)_2_REI, RE(R)_3_, and REI_3_.^108^ Beletskaya and co-workers further investigated oxidative addition of Ce, Eu, Sm, and Yb with iodothiophenes and bromopentafluorobenzene in the presence of an initiator, *e.g.*, diiodoethane or dibromoethane (Scheme 9).^109^ The authors reported the formation of organoderivatives (C_4_H_3_S)LnI (Ln = Ce, Eu, Sm and Yb) and (C_6_F_5_)LnBr (Ln = Eu, Sm and Yb), though their formation was confirmed only *via* reactivity with Ph_3_SnCl and detection of corresponding Ph_3_SnAr derivative (Ar = C_4_H_3_S, C_6_F_5_).^109^ Ce has a very unstable divalent state (Ce^3+^/Ce^2+^: E = −3.2 V);^110,111^ therefore, it cannot be excluded that reactivity with iodothiophene produced trivalent (C_4_H_3_S)CeI_2_ or (C_4_H_3_S)_2_CeI rather than divalent (C_4_H_3_S)_2_CeI, as both trivalent species can in turn generate Ph_3_Sn(C_4_H_3_S) upon reaction with Ph_3_SnCl.

(Ph)LnI (Ln = Eu, Yb) performs well as protonolysis reagent toward 3,5-diphenylpyrazole (HPh_2_pz) to give [Ln(Ph_2_pz)(I)(THF)_4_] (**16-Ln**, Ln = Eu, Yb; Ph_2_pz = {3,5-Ph_2_C_3_HN_2_}^−^; Scheme 10);^112^ the same outcome was obtained also with freshly prepared (Me)LnI.^113^ Ali *et al.* further exploited the utility of (Ph)LnI by performing protonolysis reactions with a family of amidines, ArFormH (ArNCHNAr; Ar = C_6_H_3_Me_2_-2,6 – Xyl, C_6_H_2_Me_3_-2,4,6 – Mes, C_6_H_3_^i^Pr_2_-2,6 – Dipp), leading to the formation of divalent Ln complexes [Eu(DippForm)(I)(THF)_4_] (**17**), [Eu(XylForm)(I)(DME)_2_] (**18**), [Eu(XylForm)(I)(κ^1^-DME)(μ_2_:κ^2^*O*-DME)]_∞_ (**19**), [Yb(DippForm)(I)(THF)_3_] (**20**), [{Yb(MesForm)(μ-I)(THF)_2_}_2_] (**21**) and [{Yb(XylForm)(μ-I)(THF)_2_}_2_] (**22**) (Scheme 10); however, the authors also observed the formation of several trivalent derivatives during the isolation of these species.^113^ To gain a better understating of the chemical behavior of these reagents, Junk and co-workers revisited the synthesis of putative (Ph)YbI first described by D. F. Evans and proposed the occurrence of Schlenk-type equilibria and disproportionation reactions (Scheme 10).^112,113^ The authors attempted to identify the components of these degradations and initially isolated [Yb(I)_2_(THF)_4_] and trivalent [Yb(Ph)_3_(THF)_3_]. Additionally, when phenyl iodide is reacted with Yb in DME, fractional crystallization affords [Yb(I)_2_(DME)_3_] and the mixed-valent separated ion pair complex [Yb(DME)_4_][Yb(Ph)_4_(DME)]_2_.^112^ Subsequently, Junk and co-workers ascribed the formation of the trivalent Yb formamidinate species [Yb(DippForm)(I)_2_(THF)_3_] (**23**) and [Yb(XylForm)_2_(I)(DME)] (**24**), together with the hydroxo-bridged Eu complex [{Eu(XylForm)(I)(μ-OH)(THF)_2_}_2_] (**25**), to the presence of other trivalent Yb derivatives, *e.g.*, (Ph)YbI_2_ and (Ph)_2_YbI (Scheme 11).^113^

**Scheme 12:**
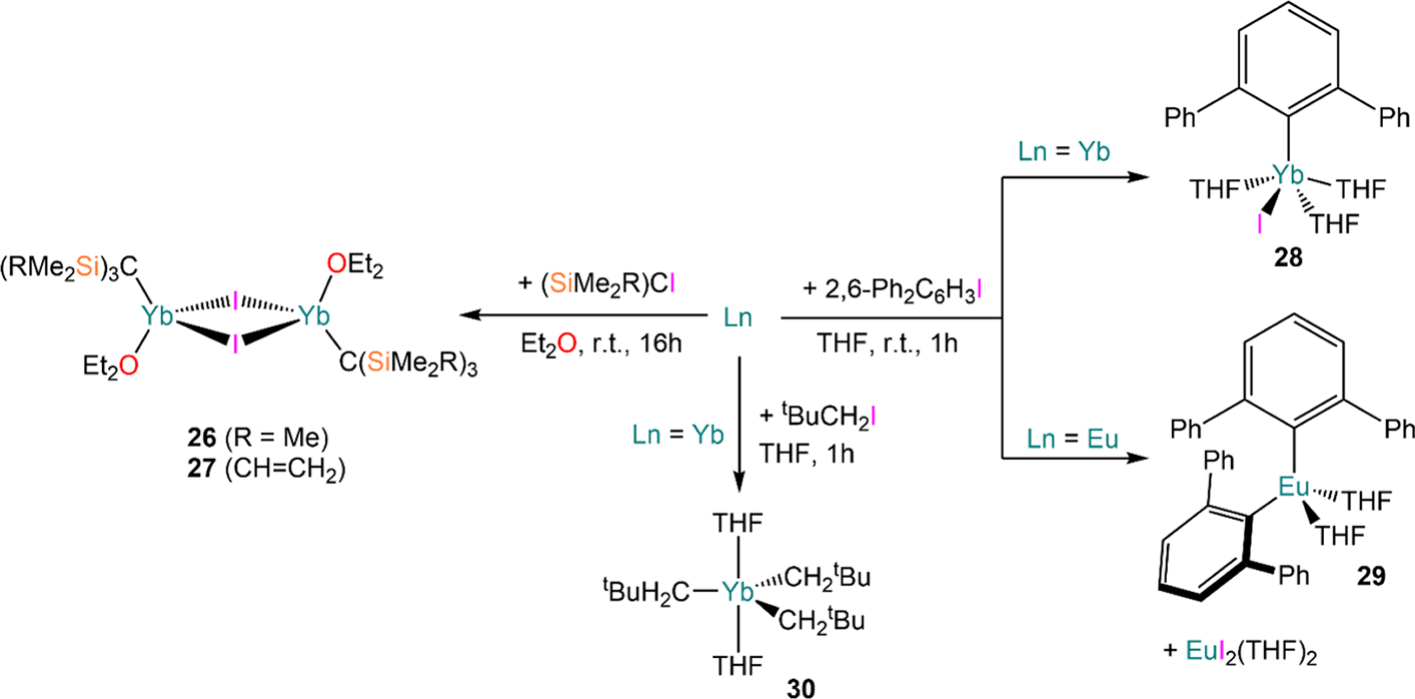
Synthesis of First Structurally Authenticated Ln *pseudo*-Grignard Species by Smith (**26** and **27**)^114,115^ and Niemeyer (**28**),^116^ Together with Synthesis of Diaryl Eu Complex **29**(116) and Trivalent Yb Derivative **30**(117)

The first structurally authenticated Ln *pseudo*-Grignard complexes [{Yb[C(SiMe_3_)_3_](μ-I)(OEt_2_)}_2_] (**26**) and [{Yb[C(SiMe_2_CH = CH_2_)_3_](μ-I)(OEt_2_)}_2_] (**27**) were reported by Smith in 1994^114^ and 1996,^115^ obtained from the direct reaction of Yb powder with (SiMe_2_R)_3_CI (R = Me, CH = CH_2_) in diethyl ether (Scheme 12). Moreover, Niemeyer and Heckmann isolated aryl complex [Yb(C_6_H_3_Ph__2__-2,6)(I)(THF)_3_] (**28**) from the reaction between 2,6-Ph_2_C_6_H_3_I and Yb powder, while use of Eu powder led to the isolation of bis-aryl complex [Eu(C_6_H_3_Ph_2_-2,6)_2_(THF)_2_] (**29**) (Scheme 12).^116^ Such a difference in reactivity between Yb and Eu is an indication of the presence of a Schlenk-type equilibrium, which is more pronounced for the larger Eu^2+^ cation. Confirmation of this observation came also from the isolation of EuI_2_(THF)_2_ as a byproduct of the reaction. Conversely, treatment of Yb powder with neopentyl iodide, ^t^BuCH_2_I, leads to further oxidation to the trivalent Yb complex [Yb(CH_2_^t^Bu)_3_(THF)_2_] (**30**) with concomitant formation of solvated YbI_2_ (Scheme 12).^117^ Niemeyer proposed that the divalent *pseudo*-Grignard species (^t^BuCH_2_)YbI is initially formed, which then reacts with a further equivalent of ^t^BuCH_2_I to yield (^t^BuCH_2_)YbI_2_; the latter species undergoes Schlenk-type equilibrium yielding **30** and YbI_3_. Finally, YbI_3_ reacts with excess Yb metal to form YbI_2_.^117^

### 3.3 Direct Metalation

**Scheme 13:**
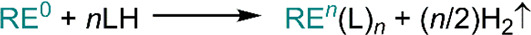
Direct Reaction of RE Metals with Protic Substrates

RE metals can react directly with protic substrates, thus providing a very efficient synthetic route which is accompanied by the evolution of hydrogen as a byproduct (Scheme 13).^118^ However, this is a relatively rare synthetic approach with limited scope, especially compared to other widely used synthetic methodologies such as salt metathesis, transamination, or transmetalation reactions; additionally, in some cases harsh reaction conditions and additives are required.

**Scheme 14:**
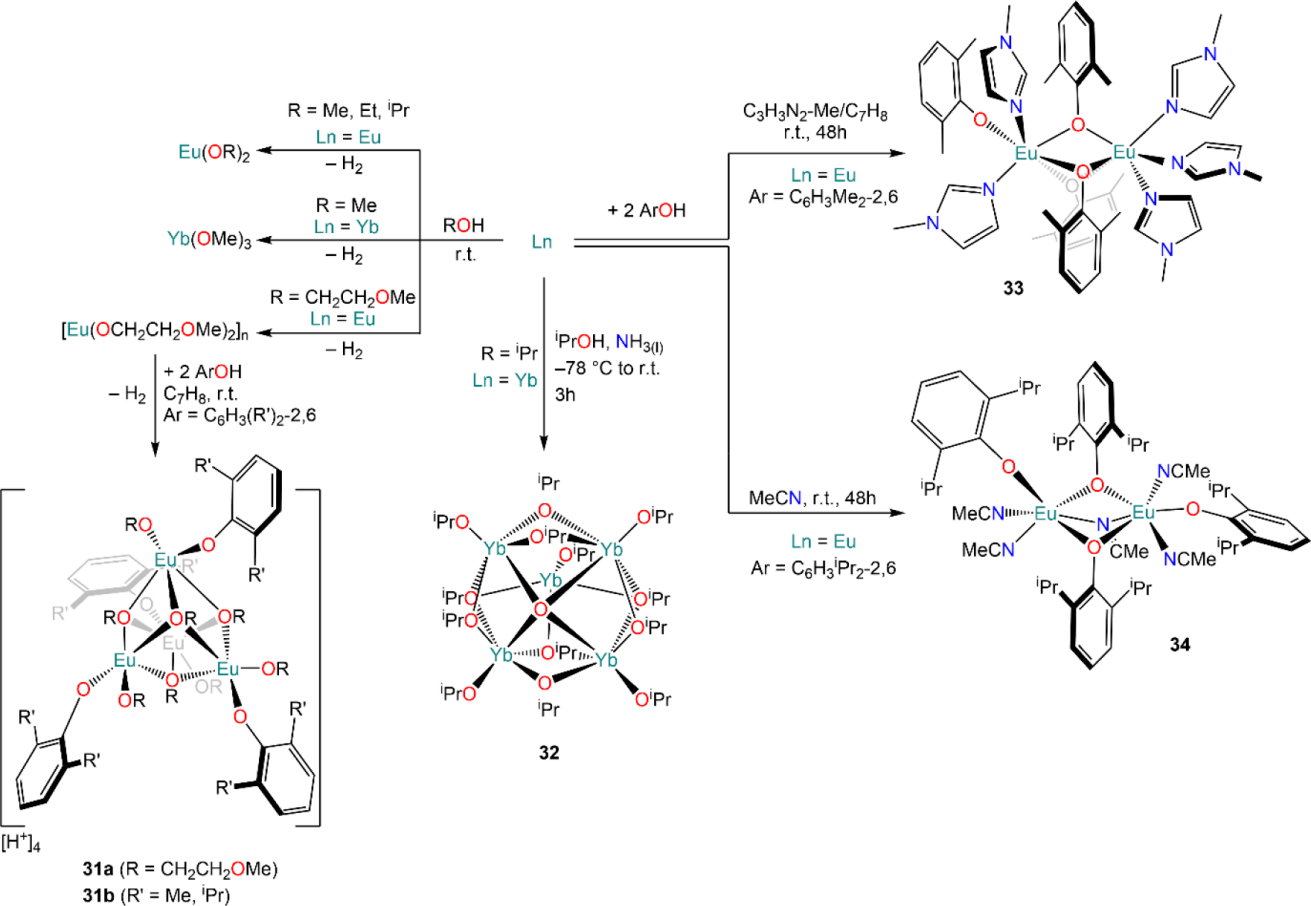
Direct Reaction of Eu and Yb with Alcohols and Application in Protonolysis Reactions^119−121^

Evans and Pires de Matos reported independently the reactivity of Ln metals with alcohols.^119−121^ The reaction of Eu metal with 2-methoxyethanol proceeds smoothly at room temperature and affords polymeric [Eu(OCH_2_CH_2_OMe)_2_]_*n*_ in good yields, which Evans and co-workers employed as protonolysis reagent toward substituted phenols HOC_6_H_4_Me_2_-2,6 (HOXyl) and HOC_6_H_3_^i^Pr_2_-2,6 (HODipp), affording the heteroleptic aryloxide complexes [{Eu(μ_3_:η^2^-OCH_2_CH_2_OMe)(η^2^-OCH_2_CH_2_OMe)(OC_6_H_3_R_2_-2,6)-][H^+^]}_4_] (**31a**, R = Me; **31b**, R = ^i^Pr) (Scheme 14).^119^ Pires de Matos and co-workers obtained Eu(OMe)_2_, Eu(OEt)_2_, Eu(O^i^Pr)_2_, and Yb(OMe)_3_ by treating metal powders with methanol, ethanol, or isopropanol (Scheme 14);^121^ in the case of the reaction between Yb and isopropanol, activation with ammonia was required and the only isolated product was the cluster [Yb_5_O(O^i^Pr)_13_] (**32**), previously prepared by Bradley and co-workers by using catalytic amounts of HgCl_2_ in the reaction (*vide infra*, Scheme 16).^122^ In the same report, Carretas *et al.* illustrated the use of metal vapor synthesis (MVS) techniques for the preparation of the same alkoxides and reported an improvement of the overall yields in all cases.^121^ Moreover, Evans and Greci investigated the use of different solvents to facilitate the direct reaction of Eu metal with HOXyl and HODipp. When HOXyl was reacted with Eu ingots in *N-*methylimidazole (C_3_H_3_N_2_-Me), the bridged aryloxide complex [Eu_2_(OXyl)(μ-OXyl)_3_(C_3_H_3_N_2_-Me)_5_] (**33**) was obtained (Scheme 14); similarly, treatment of HODipp with Eu ingots in acetonitrile yielded the aryloxide complex [Eu_2_(ODipp)_2_(μ-ODipp)_2_(MeCN)_4_(μ-NCMe)] (**34**).^120^

**Scheme 15:**
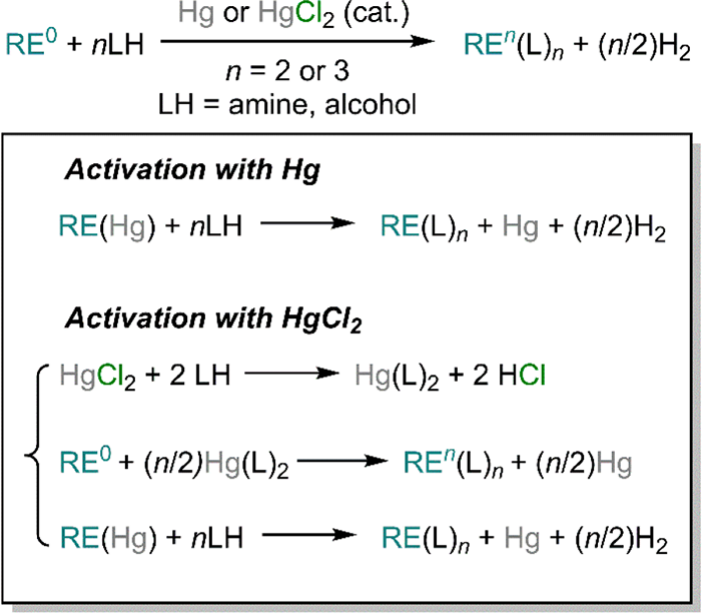
Direct Reaction between RE Metals and Protic Substrates with Catalytic Amounts of Hg or HgCl_2_

Direct metalation reactions can also be facilitated by using small quantities of Hg or HgCl_2_. The proposed mechanism involves the amalgamation of metallic mercury with the RE metals, which are readily oxidized to their trivalent or divalent state upon reaction with protic substrates, accompanied by formation of hydrogen (Scheme 15).^123^ When HgCl_2_ is employed, metallic mercury is likely formed from the direct reaction with the substrate (usually amines or alcohols), leading to the formation of Hg(L)_2_ with the concomitant production of HCl. The transient Hg amide or alkoxide then reacts with the RE metal powder *via* redox transmetalation (*vide infra*), thus producing the desired RE derivative, RE(L)_3_ or Ln(L)_2_, and regenerating metallic Hg (Scheme 15).

**Scheme 16:**
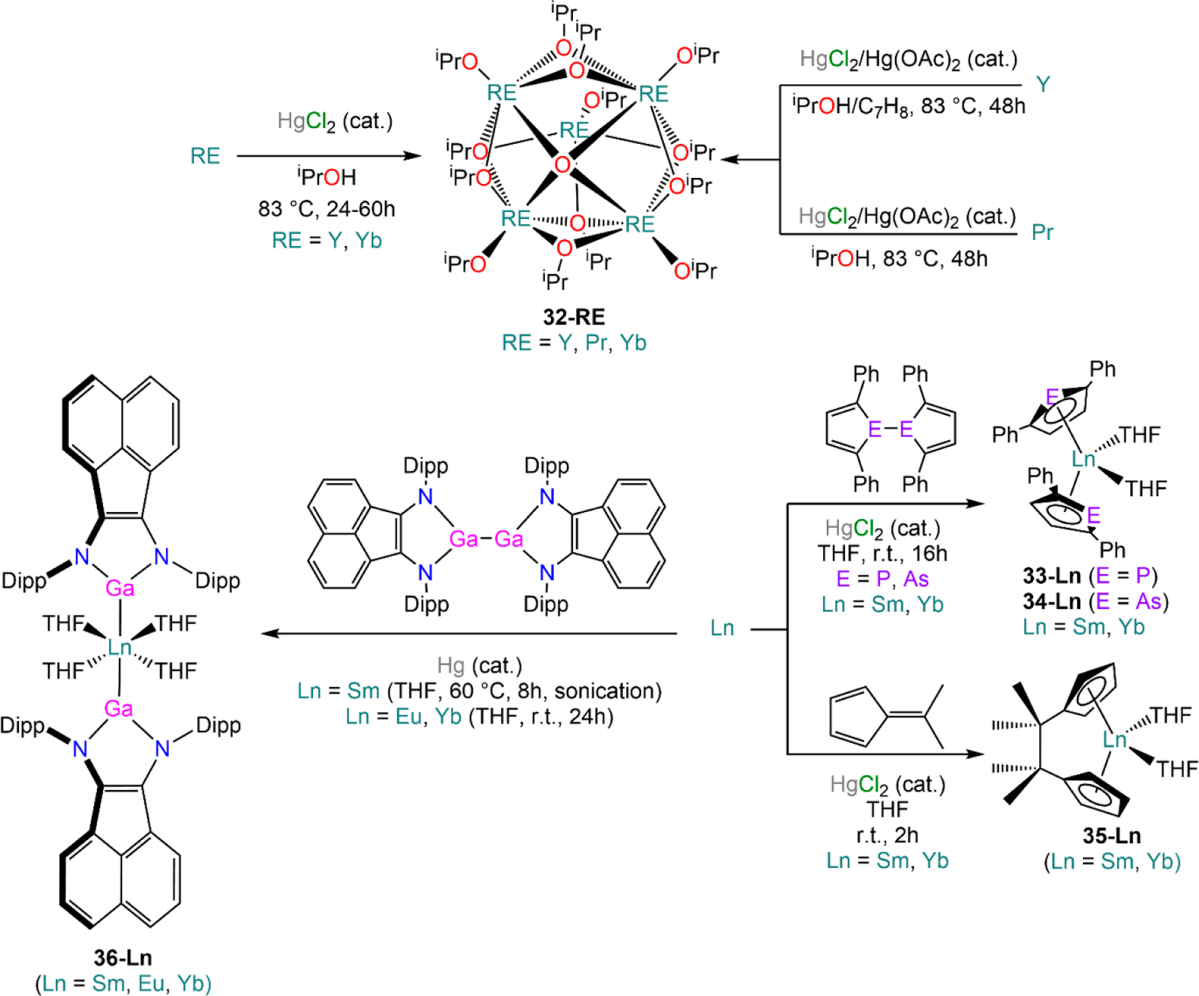
Examples of Activation with HgCl_2_ and Hg: Formation of oxo-Bridged Clusters [RE_5_O(O^i^Pr)_13_] (**32-RE**; RE = Y, Pr, Yb) *via* Direct Reaction between RE Metals and Isopropanol,^122,124,125,127,128^ and Synthesis of *bis*-Phospholyls **33-Ln**,^129^*bis*-Arsolyls **34-Ln**,^130^*ansa*-Metallocene Complexes **35-Ln**,^131^ and *bis*-Gallyl Complex **36-Ln**(132)

Mazdiyasni and co-workers were the first to report the direct activation of isopropanol with RE metals in the presence of HgCl_2_ (occasionally combined with Hg(OAc)_2_ or HgI_2_) under reflux, and they discovered that the use of excess HgCl_2_ led to the presence of RE chlorides in the final products.^124,125^ Deacon *et al.* subsequently demonstrated that solvated RECl_3_ can indeed be prepared conveniently from metal powders and HgCl_2_ (*vide infra*, section 4.2).^126^ In Mazdiyasni’s original reports, the authors identified the formation of the expected isopropoxide species RE(O^i^Pr)_3_ for all metals but Eu, which formed the divalent Eu(O^i^Pr)_2_ instead.^124,125^ These results were later revisited by Hubert-Pfalzgraf, Caulton, and Bradley, who studied the products of these direct reactions *via* single-crystal XRD studies and identified the formation of the oxo-bridged pentametallic clusters [RE_5_O(O^i^Pr)_13_] (**32-RE**; RE = Y, Pr, Yb) as the main products, rather than the expected homoleptic isopropoxide species (Scheme 16).^122,127,128^ Nief and Mathey used a similar method to synthesize [Ln(η^5^-C_4_H_2_PPh_2_-2,5)_2_(THF)_2_] (**33-Ln**; Ln = Sm, Yb) and *bis*-arsolyl [Ln(η^5^-C_4_H_2_AsPh_2_-2,5)_2_(THF)_2_] (**34-Ln**; Ln = Sm, Yb), obtained from the reaction of metal powders with *bis*-phospholyl or *bis*-arsolyl dimers in the presence of catalytic amounts of HgCl_2_ (Scheme 16).^129,130^ Additionally, Edelman and Recknagel showed that this strategy can be used in the reaction of dimethylfulvene with Ln metal powders (Ln = Sm, Yb), generating *ansa*-metallocene complexes of formula [Ln{(C_5_H_4_)_2_C_2_Me_4_}(THF)_2_] (**35-Ln**) (Scheme 16).^131^ Finally, Roesky and co-workers synthesized the gallyl lanthanide complexes [Ln{Ga(Dipp-Bian)}_2_(THF)_4_] (**36-Ln**; Ln = Sm, Eu, Yb; Dipp-Bian = 1,2-bis[(2,6-diisopropylphenyl)imino]acenaphthene) from the reduction of dimeric precursor [{Ga(Dipp-Bian)}_2_] with Ln powders activated with Hg.^132^

**Scheme 17:**
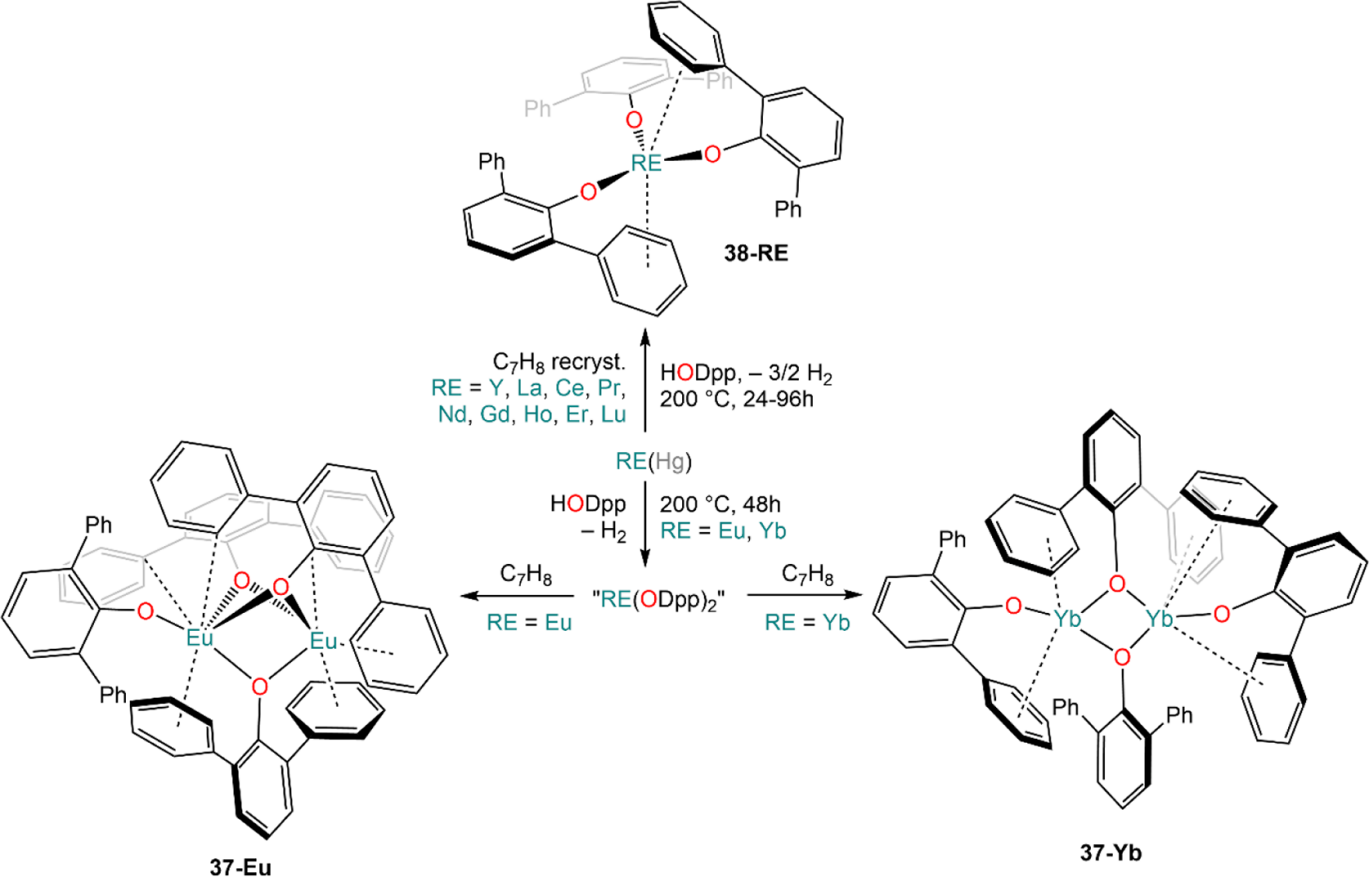
Synthesis of Aryloxides **37** and **38***via* Direct Activation of HODipp Using RE(Hg) Amalgam^134,135^

**Scheme 18:**
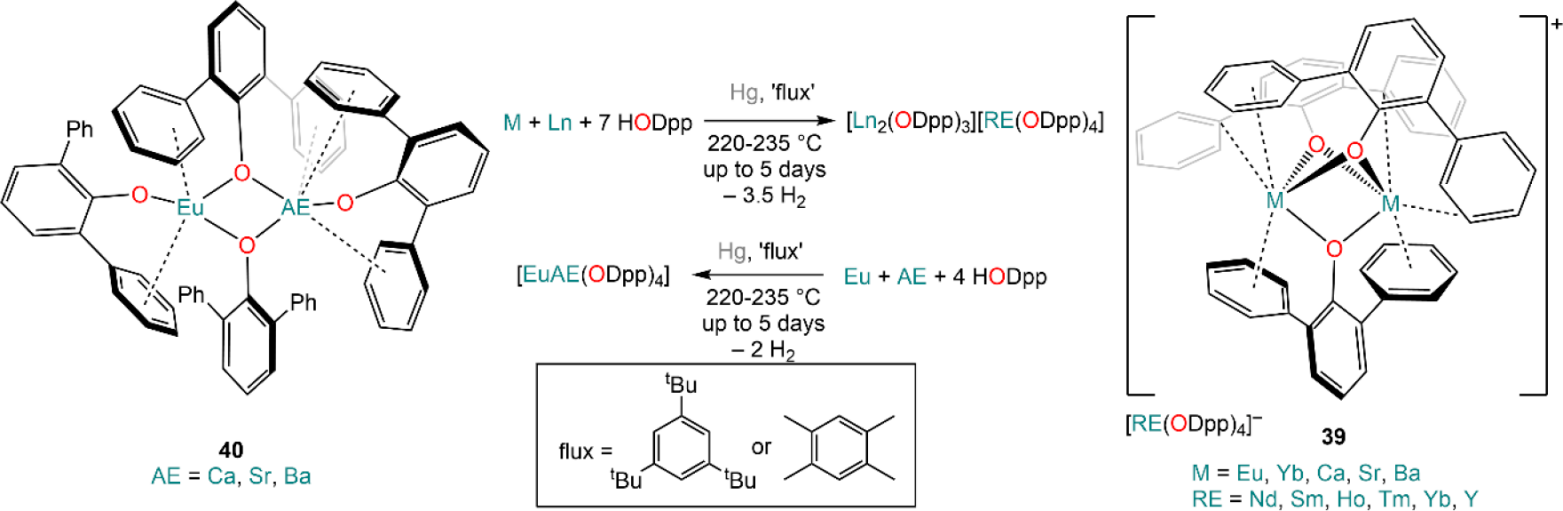
Synthesis of Heterobimetallic RE and AE Complexes Using Mercury Amalgam and Fluxes under Solvothermal Conditions^140^

This synthetic strategy was revisited by Deacon, Junk, and Müller-Buschbaum, who studied the activation of a variety of protic substrates such as substituted phenols and heterocyclic amines.^98,133^ In this new methodology, proligands are usually solids and the reactions are carried out without solvents and in the presence of Hg or HgCl_2_, operating under solvothermal conditions, *i.e.*, heating at temperatures higher than the melting point of the proligand. These types of reactions are usually carried out in partially evacuated sealed vessels (*e.g.*, Carius tubes or sealed glass ampules).^98,133^ Deacon and co-workers focused their efforts on the use of sterically demanding aryloxides and substituted pyrazolates, comprising all of the RE metals in their methodologies. Because of the absence of coordinating solvents in these reactions, homoleptic species are usually obtained with various degrees of nuclearity depending on the ligands’ steric features, ionic radii of the RE metals employed, reaction times, and crystal packing interactions in the solid state. When Eu or Yb are reacted with HOC_6_H_4_Ph_2_-2,6 (HODpp) directly, very little conversion is observed even after prolonged reaction time and under forceful conditions; when the same reactions are carried out in the presence of Hg (Scheme 17), the desired Ln(ODpp)_2_ species are generated, which can then be recrystallized from toluene to give dimeric [{Eu(ODpp)}(μ-ODpp)_3_Eu] (**37-Eu**) and [{Yb(ODpp)}_2_(μ-ODpp)_2_] (**37-Yb**) (Scheme 18).^134^ Similarly, when classic trivalent REs are employed, the expected tris-aryloxo complexes, [RE(ODpp)_3_] (**38-RE**; RE = Y, La, Ce, Pr, Nd, Gd, Ho, Er, Lu), are obtained (Scheme 17); also in the case of RE^3+^ ions, direct reactivity in the absence of Hg leads to very little conversion into the desired products.^123^ Deacon and Junk successfully extended this methodology to other REs (including Sc)^135^ and aryloxides comprising different substitution patterns of the aryl groups,^136−138^ including buttressing substituents in 3- and 5-positions.^135^ Additionally, reactions can also be carried out in the presence of fluxes (*e.g.*, 1,3,5-tri*tert*-butylbenzene and 1,2,4,5-tetramethylbenzene) which increase the contact between metal and alcohol. This methodology has proven to be particularly useful for the preparation of heterobimetallic compounds and can be employed also for the synthesis of bimetallic systems incorporating alkali^139^ and AE metals (**39** and **40**, Scheme 18).^140^ One of the great advantages of these methodologies is that resulting complexes can spontaneously crystallize from the reaction mixtures in the Carius tubes upon cooling, together with avoiding the preparation of tailored starting materials and using coordinating solvents. However, in some cases recrystallizations have to be carried out, and owing to the insolubility of these species in nonpolar solvent media, these usually have to be performed at very high temperatures (*e.g.*, toluene at 190 °C).^140^

**Scheme 19:**
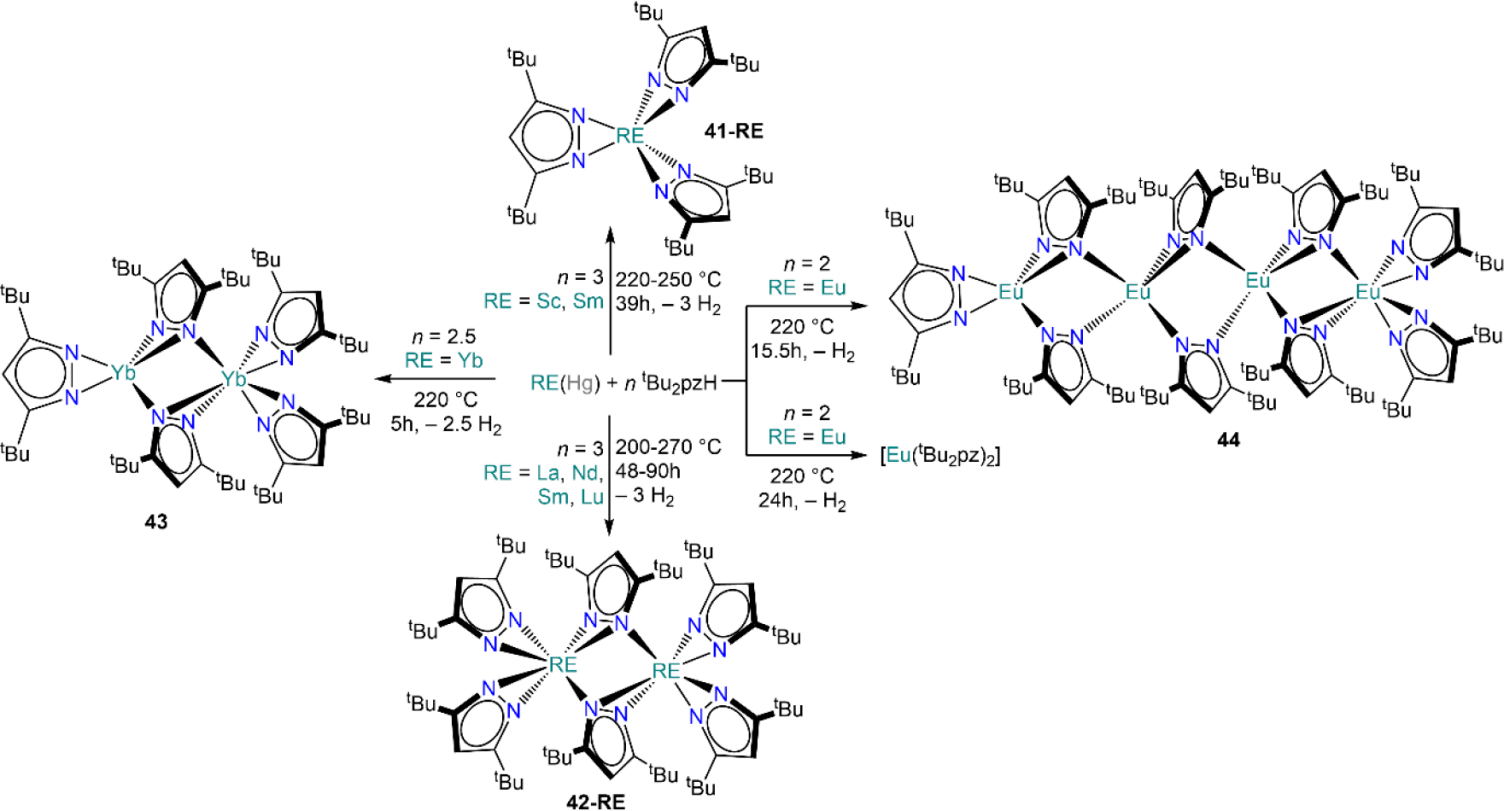
Examples of RE Pyrazolates Synthesized by Deacon and Co-workers Using Mercury Amalgam under Solvothermal Conditions^141,143^

Reactivity of REs (Sc, La, Nd, Eu, Sm, Yb, and Lu) with bis-*tert*-butylpyrazole (^t^Bu_2_pzH) in the presence of Hg and under solvothermal conditions delivers monomeric, [RE(^t^Bu_2_pz)_3_] (**41-RE**; RE = Sc, Sm), and dimeric species, [RE_2_(^t^Bu_2_pz)_6_] (**42-RE**; RE = Nd, Sm, Lu).^141^ Similarly, **41-Sc** can also be obtained *via* direct synthesis in the presence of mercury metal at temperatures between 270 and 300 °C.^142^ When divalent Eu and Yb are employed, the methodology produces slightly different outcomes, leading for example to the isolation of the mixed-valent Yb(II)/(III) complex [Yb_2_(^t^Bu_2_pz)_5_] (**43**, Scheme 19).^143^ Interestingly, these methodologies are affected by subtle variations in reaction conditions. For example, in the case of Eu heating of the reaction with ^t^Bu_2_pzH at 220 °C for 24 h affords the monomeric complex [Eu(^t^Bu_2_pz)_2_] (though no structural validation has been provided for this conformation), while after heating for 15.5 h the tetrameric species [Eu_4_(^t^Bu_2_pz)_8_] (**44**) is obtained instead (Scheme 19).^141^ Similar to the use of substituted phenols (*vide supra*, Scheme 17), this is a very effective methodology for the synthesis of homoleptic solvent-free RE complexes and has also been extended to other pyrazoles featuring varying degrees of substitution.^144^ Kempe and Deacon have also reported the direct reaction of Yb metal with substituted aminopyridines, leading to the formation of homoleptic divalent and trivalent Yb complexes depending on the ligand employed.^145^

**Scheme 20:**
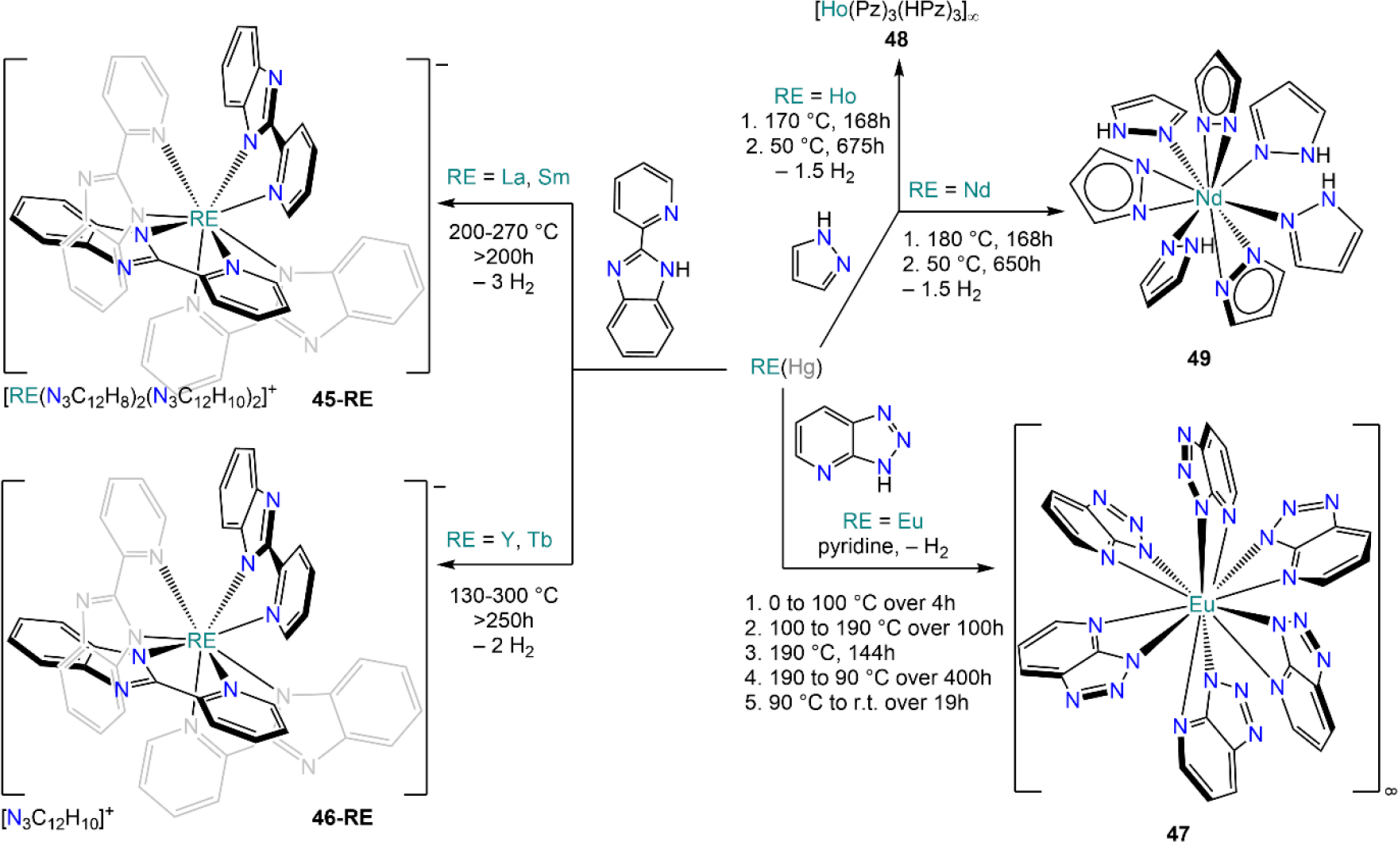
Synthesis of RE Amides *via* Solvothermal Synthesis with Mercury Amalgam by Müller-Buschbaum and Co-workers^146−148^

Müller-Buschbaum and co-workers extended this approach to other heterocyclic *N-*donors including *1H*-1,2,3-benzotriazolo[4,5-*b*]pyridine (**45-RE**, RE = La, Sm; **46-RE**, RE = Y, Tb),^146^ benzimidazole (**47**),^147^ and unsubstituted pyrazole (**48** and **49**, Scheme 20).^148^ All the reactions were performed in evacuated reaction vessels and under solvothermal conditions “in the melt”, with varying reaction temperatures depending on the substrates. The methods employed by Müller-Buschbaum are perfectly suited for the preparation of homoleptic derivatives with several REs, though in some cases proligands are also included in the resulting complexes, either trapped in the lattice through solid-state packing interactions or by coordinating directly to the metal centers (Scheme 20).

**Scheme 21:**
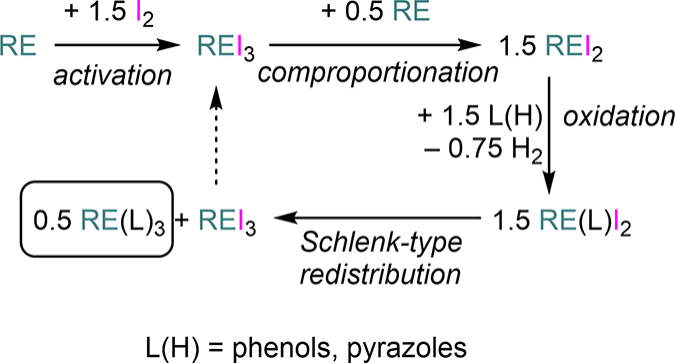
Activation of REs with Iodine or REI_3_ and Reaction with Protic Substrates

Another activation strategy consists of the use of iodine, which is a methodology commonly employed for the activation of Mg in the preparation of Grignard reagents;^149^ REs can also react directly with I_2_ to give REI_2_ and REI_3_ salts (*vide infra*, sections 4.1 and 4.2). Small amounts of iodine have been used by Junk, Deacon, and co-workers to activate a range of REs (Y, La, Nd, Sm, Eu, Dy, and Yb) and react them with variously substituted phenols and pyrazoles.^150−153^ The exact nature of the mechanism involved in this methodology has not been fully identified; however, Junk and Deacon invoked the formation of highly reactive divalent REI_2_ salts, obtained from the comproportionation reaction between REI_3_ and RE.^150^ The transient divalent species is oxidized by protic proligand substrates, followed by Schlenk-type rearrangement to give homoleptic RE(L)_3_ (L = alkoxide, pyrazolate) complexes together with the regeneration of REI_3_ (Scheme 21).

**Scheme 22:**
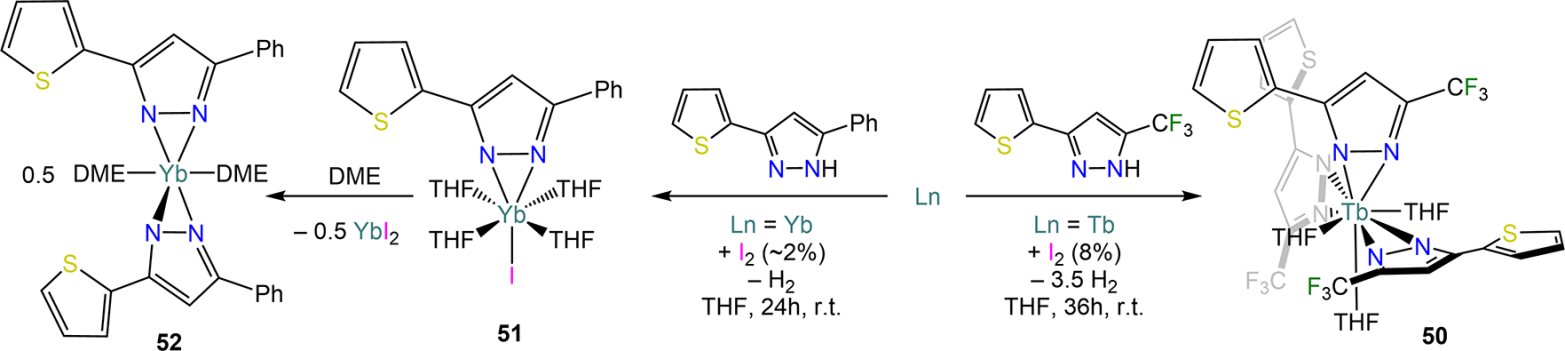
Reactivity of Substituted Pyrazoles with Ln Metals (Ln = Yb, Tb) in the Presence of Iodine Reported by Deacon and Junk^151,152^

**Scheme 23:**
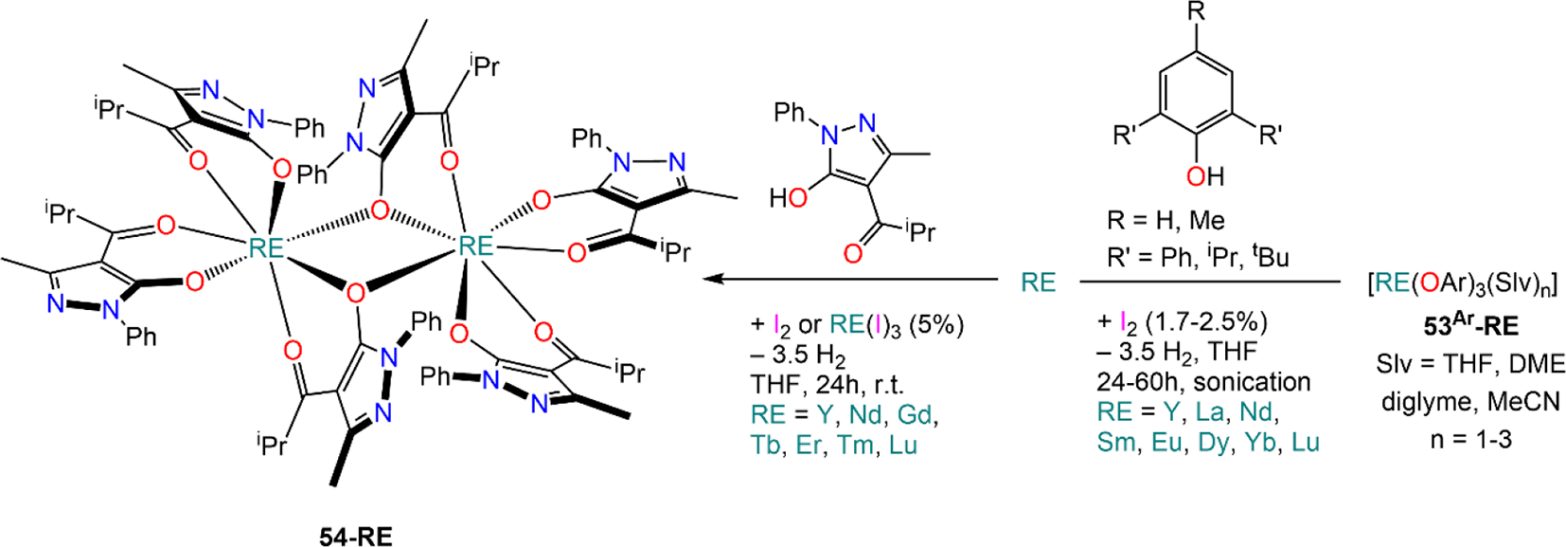
Reactivity of Iodine-Activated RE Metals with Alcohols by Junk, Deacon^150,153^ and Bochkarev^156^

Junk and Deacon have reported the reaction of Yb and Tb metal with variously substituted pyrazoles, C_3_H_2_N_2_(CF_3_)-1-(C_4_H_3_S)-3 (HTtfpz) and C_3_H_2_N_2_-Ph-1-(C_4_H_3_S)-3 (HPhtpz) (Scheme 22).^151,152^ The former affords THF-solvated complex [Tb(Ttfpz)_3_(THF)_3_] (**50**) when reacted with Tb filings and few crystals of iodine (*ca.* 8%), though the product is isolated in rather low yields. In the case of the reaction of HPhtpz with Yb, the intermediate [Yb(Phtpz)(I)(THF)_4_] (**51**) is first isolated, which upon treatment with DME affords the *bis*-pyrazolate complex [Yb(Phtpz)_2_(DME)_2_] (**52**). Interestingly, this behavior is not observed in the reactions involving substituted phenols, where homoleptic species of the type [RE(OAr)_3_(Slv)_*n*_] (**53**^**Ar**^**-RE**; OAr = ODipp, OMes, ODpp, ODbmp; Slv = THF, DME, diglyme, MeCN; *n* = 1–3)^150,153^ are usually obtained even with Yb.^150^ Very recently, Junk, Deacon, and co-workers have also used a similar methodology for the preparation of homoleptic Ln(II) and heteroleptic RE(III) formamidinates.^154,155^ Bochkarev and co-workers have also shown that small amounts of preformed REI_3_ (*ca.* 5 mol %) can be used in conjunction with metallic REs to give very similar results in the reaction with 1-phenyl-3-methyl-4-isobutyryl-5-pyrazolone (PMIP), to give dimeric complexes [{RE(PMIP)(μ-PMIP)_2_}_2_] (**54-RE**; RE = Y, Nd, Gd, Tb, Er, Tm, Lu) (Scheme 23).^156^ Mashima *et al.* have also employed a similar methodology for the synthesis of RE(III) cyclooctatetraenyl complexes; in their synthetic strategy, RE metals are reacted with cyclooctatetraene in the presence of equimolar amounts of iodine, 1,2-dibromoethane, or Ph_3_PCl_2_ in hot THF, affording heteroleptic COT complexes with the formula [RE(COT)(I)(THF)_*n*_] (RE = La, Ce, Pr, *n* = 3; RE = Nd, *n* = 2; Ln = Sm, *n* = 1), [{Sm(COT)(μ-X)(THF)_2_}_2_] (X = Cl, Br).^157^

**Scheme 24:**
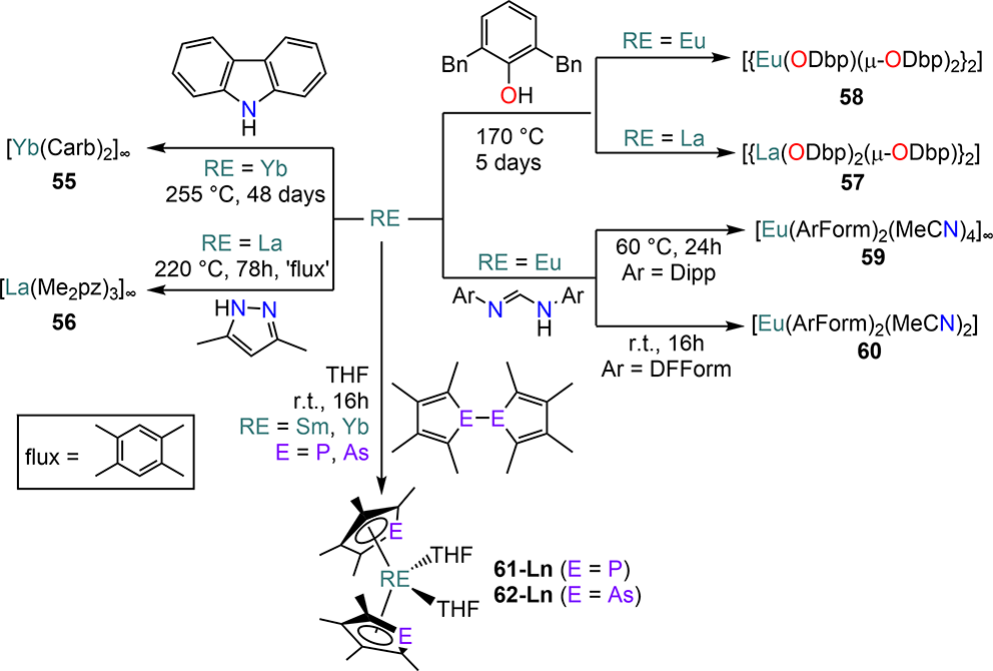
Direct Reactivity of RE Metals with Protic Substrates, *bis*-Phospholyls and *bis*-Arsolyls and in the Absence of Activators^94,129,136,158−160^

In some cases, Ln and RE metals can react directly without the aid of an activating agent, though this is a rare occurrence. For example, Müller-Buschbaum and Quitmann showed that carbazole reacts directly with Yb under solvothermal conditions (255 °C, carbazole mp 246 °C, Scheme 24) after a quick initiation at 280 °C, to form the homoleptic coordination polymer [Yb(Carb)_2_]_∞_ (**55**).^158^ The outcome of this reaction is analogous to that obtained with Yb ammoniacal solutions of Yb, though requiring harsher conditions and long reaction times (*ca.* 48 days).^94^ Anwander and co-workers have also shown that 3,4-dimethylpyrazole reacts with La powder at high temperatures (220 °C) under partial vacuum (Scheme 24), leading to the formation of coordination polymer [La(Me_2_pz)_3_]_∞_ (**56**).^159^ Similarly, some REs are also able to react directly with pyridylbenzimidazoles under solvothermal conditions and in the absence of Hg.^147^ Additionally, in 2005 Junk and co-workers reported the direct reaction of La, Eu, and Yb with 2,6-dibenzylphenol (HODbp) carried out at 170 °C (Scheme 24).^136^ The reactions with La and Eu proceed smoothly: in the case of La, the trivalent bimetallic complex [{La(ODbp)_2_(μ-ODbp)}_2_] (**57**, ODbp = {O–C_6_H_3_(CH_2_Ph)_2_-2,6}^−^) is isolated, whereas the divalent species [{Eu(ODbp)(μ-ODbp)}_2_] (**58**) is obtained when Eu metal is employed.^136^ Eu can also react directly with *N*,*N*′-bis(aryl)formamidines (ArFormH, Ar = Dipp, DF; DF = C_6_H_3_F_2_-2,6) in acetonitrile to give complexes of formula [Eu(ArForm)_2_(CH_3_CN)_2_] (**59**, Ar = Dipp; **60**, Ar = DF), while Yb requires the presence of catalytic amounts of Hg to obtain analogous complexes (Scheme 24).^160^ Nief and Mathey also showed that Sm and Yb powders can react directly with biphospholyls and biarsolyls in THF at room temperature (Scheme 24), breaking the E–E (E = P, As) bond and forming *bis*-phospholyl and arsolyl complexes [Ln(η^5^-C_4_Me_4_P)_2_(THF)_2_] (**61-Ln**) and [Ln(η^5^-C_4_Me_4_As)_2_(THF)_2_] (**62-Ln** Ln = Sm, Yb).^129,130^

### 3.4 Redox Transmetalation

**Scheme 25:**
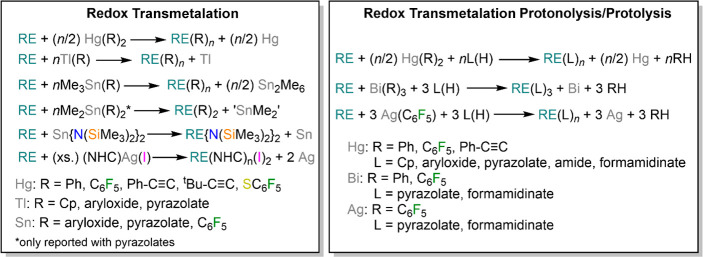
RT and RTP Reactions of RE Metals with Hg, Tl, Sn, Bi, and Ag Reagents

**Figure 2:**
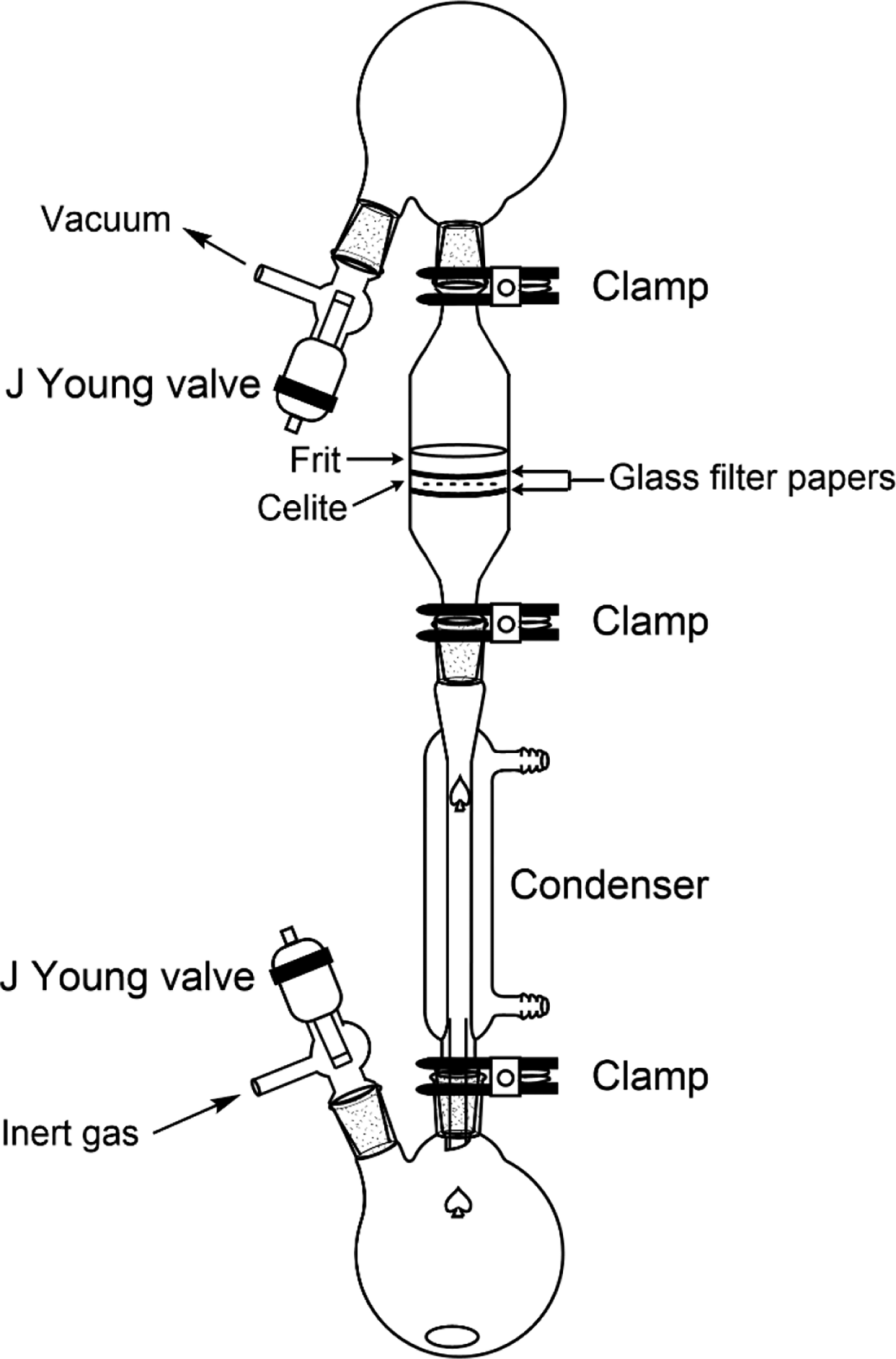
Sketch of the apparatus originally developed by Deacon and co-workers for RT and RTP reactions with Hg and Tl reagents. *Note:* All joints are kept grease-free by using PTFE sleeves. Reproduced with permission from ref (179). Copyright 1990 Wiley.

Direct reactivity of REs with proligands often requires prior activation of the metal or some other activation strategy (see sections 3.1, 3.2 and 3.3). However, there are some examples of direct reactivity in which the RE metal does not require activation and is involved in a redox exchange with another metal-containing species. Such species are typically transmetalating reagents, with organomercurials as the most popular choices (*e.g.*, HgPh_2_, Hg(C_6_F_5_)_2_, and Hg(CCPh)_2_);^12,35,161−165^ however, these methodologies have also been extended to include other redox active metals, such as Sn,^90,166,167^ Tl,^91,168−174^ Bi,^175,176^ and Ag.^177^ The first reaction of an RE with an organomercurial reagent was reported in 1945 by Gilman and Jones, who reacted La metal with HgPh_2_ though they were not able to identify the reactivity products.^178^ Broadly speaking, these methodologies can be divided into two categories: (1) redox transmetalation (RT) reactions and (2) redox transmetalation combined with ligand exchange, also termed redox transmetalation protonolysis or protolysis (RTP) (Scheme 25).^12^ Though initially developed for classic divalent Lns (Yb and Eu), RT and RTP reactions can also be used for the synthesis of trivalent RE derivatives.^12^ RT/RTP reactions have been pioneered by Deacon and co-workers since the 1970s, who developed the first applications in RE coordination and organometallic chemistry and also engineered bespoke reaction apparatuses (Figure 2) for facile removal of hazardous byproducts (*i.e.*, Hg and Tl).^179^ Their implementation in synthetic protocols has given access to complexes supported by a wide array of ligands such as cyclopentadienyl,^168,169,172,173,180^ carboranes,^181^ monodentate amides,^90,182−185^ bidentate amides,^160,186−191^ pyrazolates,^166,174,175^ aryloxides,^91,170,171,183,192^ thiolates^193,194^ and *N-*heterocyclic carbenes (NHCs).^195,196^ These methodologies have been covered extensively in other review articles;^12,35^ therefore, this section will focus on key examples that best demonstrate their applications in RE chemistry.

**Scheme 26:**
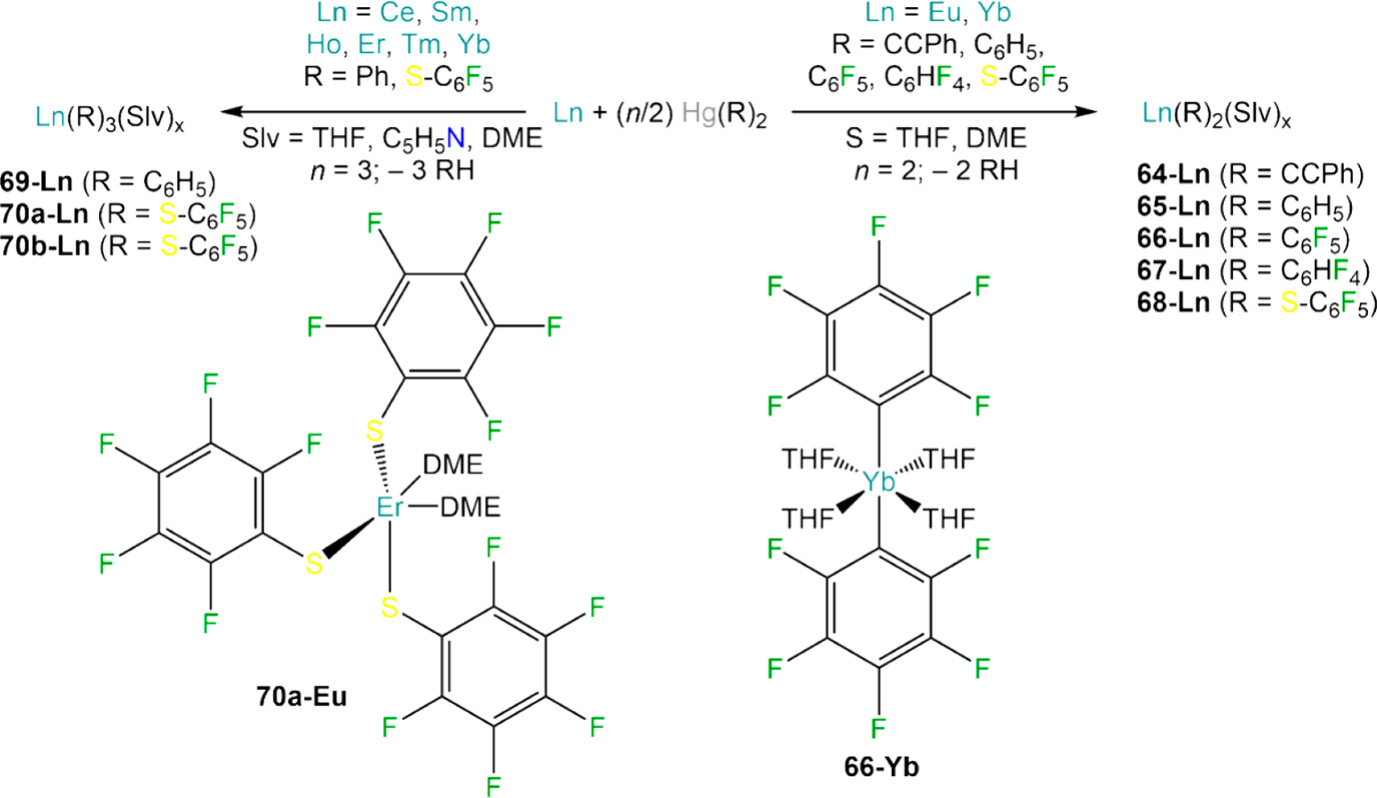
RT Reactions of Organomercurial Reagents with Divalent and Trivalent Lns^161−165,193,194^

In general, RT and RTP methodologies are very useful for obtaining homoleptic complexes, though it should be noted that coordinating solvents are usually required for these reactions and solvated derivatives are often isolated as a result. In some cases activation of the metal is required, which can be achieved by one of the methods illustrated in previous sections (*e.g.*, addition of small quantities of Hg, I_2_, and REI_3_). Deacon pioneered the use of Hg reagents in RT and RTP reactions and reported the preparation of several organoytterbium and organoeuropium complexes, Ln(R)_2_ (Ln = Eu, Yb; **64-Ln**, R = CCPh; **65-Ln**, CC^t^Bu; **66-Ln**, Ph; **67-Ln** C_6_F_5_; **68-Ln** o-HC_6_F_4_; **68-Ln** p-HC_6_F_4_), by reacting metal powder with the corresponding organomercurial reagent in ethereal solvents (Scheme 26).^161−165^ While Yb reacts smoothly with organomercurials, in the case of Eu, addition of Hg metal is necessary for the reaction to take place. Interestingly, reactivity of Sm powder with Hg(R)_2_ reagents is less straightforward; when Hg(C_6_F_5_)_2_ is employed, Sm(C_6_F_5_)_2_ or Sm(C_6_F_5_)_3_ cannot be isolated and various decomposition products are obtained instead, *e.g.*, SmF_2_/SmF_3_, Sm(C_6_F_5_)(F)_2_, and fluorohydrocarbons,^164,197^ and similar issues are encountered when using other trivalent Lns. Bochkarev and co-workers were the first to report the synthesis of [Er(Ph)_3_(THF)_3_] (**69-Er**) and [Tm(Ph)_3_(THF)_3_] (**69-Tm**) from the reaction of metal powders and HgPh_2_ in the presence of small quantities corresponding triiodides (ErI_3_ and TmI_3_), though the isolation of such species is extremely challenging.^176^ Despite the difficulties in isolating putative RE(R)_3_ (R = CCPh, Ph, C_6_F_5_) *via* RT, reactivity *in situ* of trivalent REs with organomercurials is used extensively as a first step in RTP reactions with various protic substrates (*vide infra*). Nonetheless, RT reactions with fluorinated thiols proceed smoothly to give fluorinated thiolate complexes [Ln(S–C_6_F_5_)_3_(Slv)_*x*_] (**70a-Ln**; Ln = Ho, Er, Yb; Slv = THF, DME, C_5_H_5_N; *x* = 2–3) and [{Ln(S–C_6_F_5_)_2_(μ–S-C_6_F_5_)(THF)_*x*_}_2_] (**70b-Ln**; Ln = Ce, Sm; *x* = 1, 2), which can also be obtained using RTP methodologies involving Hg(C_6_F_5_)_2_ (Scheme 26).^193,194^

**Scheme 27:**
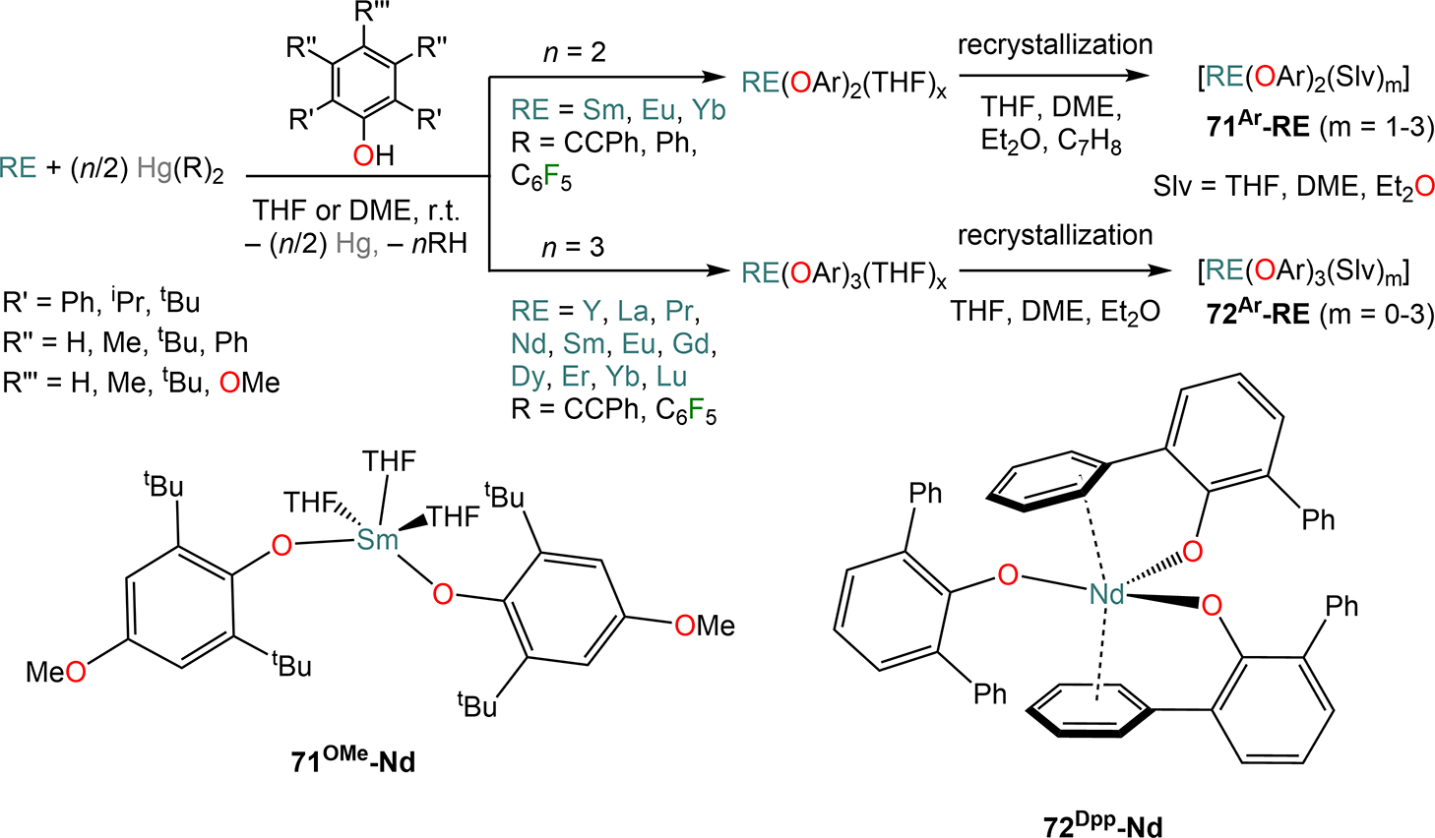
RTP Reactions of RE Metals and Organomercurials with Substituted Phenols^91,112,123,135,153,183,198,199^

Deacon and co-workers have shown that Hg-mediated RTP reactions are extremely effective for the preparation of RE(II) and RE(III) complexes with various aryloxide (OAr) ligands (Scheme 27).^91,112,123,135,153,183,198,199^ These reactions are typically carried out at room temperature with relative short reaction times, and the resulting complexes are usually crystallized as THF adducts, [RE(OAr)_*n*_(THF)_*m*_] (**71**^**Ar**^**-RE**, *n* = 2, RE = Sm, Eu, Yb; **72**^**Ar**^**-RE**, *n* = 3, RE = Y, La, Pr, Nd, Sm, Eu, Gd, Dy, Er, Yb, Lu; *m* = 0–3), though in some cases solvent-free monomeric complexes can also be isolated, *e.g.* [Nd(ODpp)_3_] (**72**^**Dipp**^**-Nd**, Scheme 27).^200^ The outcome of these RTP reactions can be affected by the nature of the RE involved, the choice of organomercurial reagents, and aryloxide ligand. In the case of Yb and Eu, the corresponding divalent complexes **71**^**Ar**^**-RE** are usually obtained, which can be crystallized as THF adducts.^200^ Interestingly, the use of different Hg(R)_2_ reagents can selectively switch oxidation of Yb metal between Yb(II) and Yb(III); this was demonstrated by Deacon *et al.* in the reaction with 3,5-substituted 2,6-diphenylphenols: reactivity between Yb powder, 2,6-diphenyl-3,5-dimethylphenol and HgPh_2_ leads to the Yb(II) complex **71**^**Ar**^**-Yb**, whereas when Hg(C_6_F_5_)_2_ is used the trivalent complex **72**^**Ar**^**-RE** is isolated instead.^135^ In some cases isolating pure divalent or trivalent complexes can be problematic, like in the case of RTP reactions with Hg(C_6_F_5_)_2_ and the phenol HOC_6_H_2_-2,6-^t^Bu-4-OMe (HOAr^OMe^). When Yb is employed, the divalent complex [Yb(OAr^OMe^)_2_(THF)_3_] (**71**^**OMe**^**-RE**) is obtained in very good yields, while in the case of Sm the corresponding divalent and trivalent complexes [Sm(OAr^OMe^)_2_(THF)_3_] (**71**^**OMe**^**-RE**) and [Sm(OAr^OMe^)_3_(THF)] (**72**^**OMe**^**-RE**) are obtained, with the latter as the major product.^199^ RE-fluoride species can also be obtained because of the decomposition of RE-(C_6_F_5_) intermediates with certain supporting aryloxides.^199^ To solve this problem, Deacon and co-workers replaced Hg(C_6_F_5_)_2_ with Hg(CCPh)_2_ in their methodology and obtained the target homoleptic species **71**^**Ar**^**-RE** or **72**^**Ar**^**-RE**.^199^

**Scheme 28:**
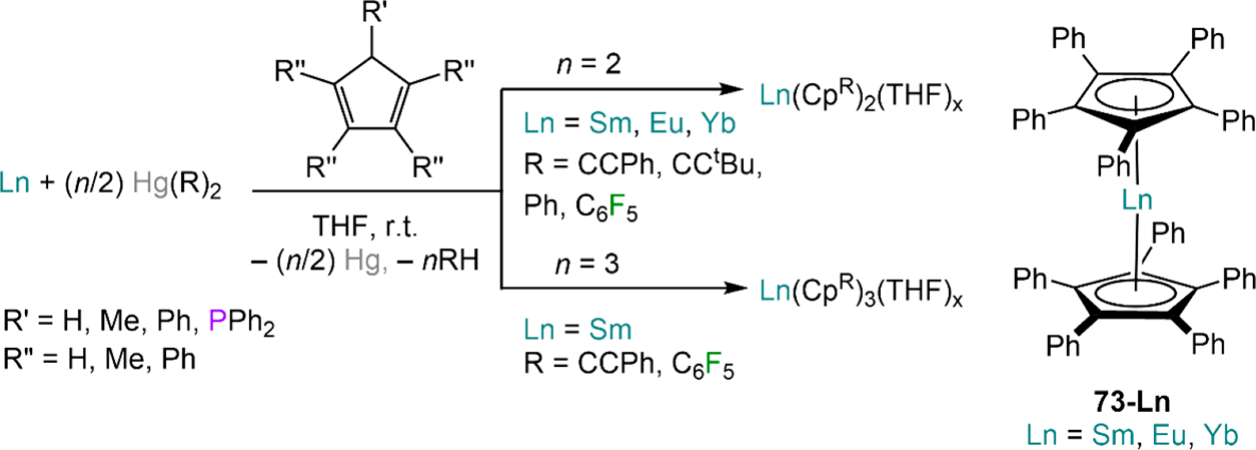
Examples of RTP Methodologies Involving Organomercurials and Cyclopentadienyl Ligands^180,201−203^

Some of the earliest applications of RTP methodologies were the preparation of Cp derivatives, comprising both divalent Ln(Cp)_2_ and trivalent Ln(Cp)_3_ complexes (Scheme 28).^197^ These methodologies have also been extended to substituted Cp ligands, such as {C_5_H_4_Me}^−^ (Cp^Me^),^197^ Cp*,^180^ and {C_5_H_4_PPh_2_}^−^ (Cp^PPh2^).^170^ Deacon and co-workers found that when Yb(C_6_F_5_)_2_ was used as protonolysis reagent with HCp^Me^ the reaction afforded an explosive solid, likely because of the formation of unstable fluorinated species.^197^ Therefore, the use of fluorine-free organomercurials is often preferable for these methodologies. RTP reactions are also capable of producing homoleptic Ln(Cp)_2_ complexes with extremely bulky ligands, such as {C_5_HPh_4_}^−^ (Cp^Ph4^) and {C_5_Ph_5_}^−^ (Cp^Ph5^), *e.g.* [Ln(Cp^Ph5^)_2_] (**73-Ln**; RE = Sm, Eu, Yb),^201,202^ [Yb(Cp^Ph4^)_2_(THF)].^202,203^ This is a particularly remarkable application as classic metathetical reactivity between alkali metal Cp salts and RE halides is often not suitable to obtain Cp derivatives with high steric congestions. Interestingly, Jaroschik, Deacon, and co-workers occasionally used also heteroleptic organomercurials, Hg(Ph)(C_6_F_5_) and Hg(Ph)(CCPh), for some of these reactions.^201,204,205^

**Scheme 29:**
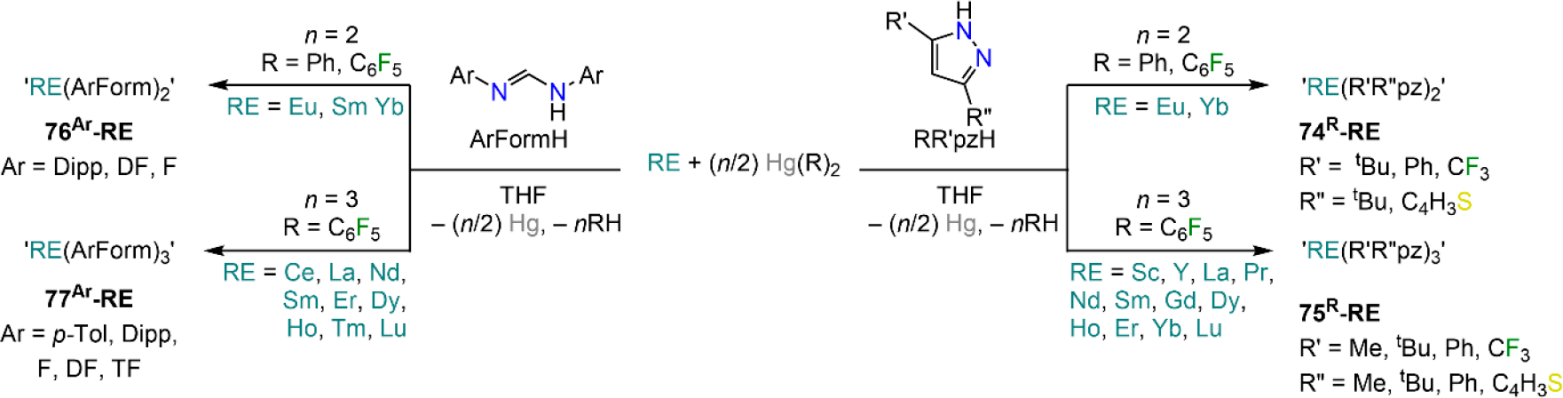
Synthesis of Pyrazolate^174,206−208^ and Amidinate^160,186,188−191,211^ RE Complexes *via* RTP Reactions with Organomercurials

Another successful application of RTP reactions with REs and organomercurials involves the use of mono- and multidentate *N*-donors. A large number of protic substrates have been employed, with silylamines, arylamines, formamidines, and pyrroles among the most popular.^12^ This is a particularly important use of RTP methodologies owing to the prominent role played by *N-*donors in RE coordination chemistry.^7,24^ Also in this case, the main products of these reactions are homoleptic bis-amide complexes, both with mono- and multidentate donors. Deacon and co-workers have applied this methodology to several substitute pyrazolates and showed that this approach is applicable to both divalent Lns (Eu and Yb) and trivalent REs (Scheme 29).^174,206−208^ The choice of mercurial reagent can be crucial for the outcome of these reactions. When HgPh_2_ is reacted with Yb and proligand in a 1:2 stoichiometry, the target bis-pyrazolates Yb(R_2_pz)_2_ (**74**^**R**^**-Yb**) are usually obtained.^174^ However, when Hg(C_6_F_5_)_2_ is employed, the reaction leads to oxidation to Yb(III) and formation of Yb(R_2_pz)_3_ (**75**^**R**^**-Yb**);^174,209,210^ it is noteworthy that short reaction times tend to favor the formation of **74**^**R**^**-Yb** over **75**^**R**^**-Yb**.^35^ Interestingly, direct protonolysis between Yb(C_6_F_5_)_2_ and Ph_2_pzH proceeds smoothly to give Yb(Ph_2_pz)_2_ (**75**^**Ph2**^**-Yb**).^174^ The use of RTP reactions is exemplified by the extensive work done by Junk, Deacon, and co-workers in the synthesis of RE formamidinate (ArForm; Ar = Dipp, *p*-Tol, *o*-(CF_3_)C_6_H_4_, DF, 2,3,4,5-F_4_C_6_H – TF, *o*-FC_6_H_4_ – F) complexes (Scheme 29).^160,186,188−191,211^ For these ligand systems, reactivity conditions and stoichiometries can be tailored to obtain either divalent or trivalent formamidinate complexes, “RE(ArForm)_2_” (**76**^**Ar**^**-RE**) and “RE(ArForm)_3_” (**77**^**Ar**^**-RE**), irrespective of the choice of organomercurial agent, although Hg(C_6_F_5_)_2_ is usually the oxidant of choice. It should be noted that a large excess of metal filings is used for Yb to afford clean conversion to divalent **76**^**Ar**^**-Yb**. Reactions are usually carried out in THF, and the resulting complexes are normally isolated as ethereal adducts (*e.g.*, THF, DME, diglyme) upon recrystallization, with varying coordinated solvents depending on steric congestion around the metal center and differences in ionic radii. As previously discussed, fluorinated mercurial reagents can lead to the formation of undesired byproducts through fluoride abstraction and oxidation of the RE metal. Junk and co-workers have been able to take advantage of this type of reactivity to selectively synthesize heteroleptic complexes of general formula RE(ArForm)_2_(X) (X = F, Br), such as [La(DippForm)_2_(F)(THF)]^211^ and [RE(DippForm)_2_(Br)(THF)] (RE = La, Nd, Yb),^188^ the latter obtained by employing Hg(2-BrC_5_F_4_)_2_.

**Scheme 30:**
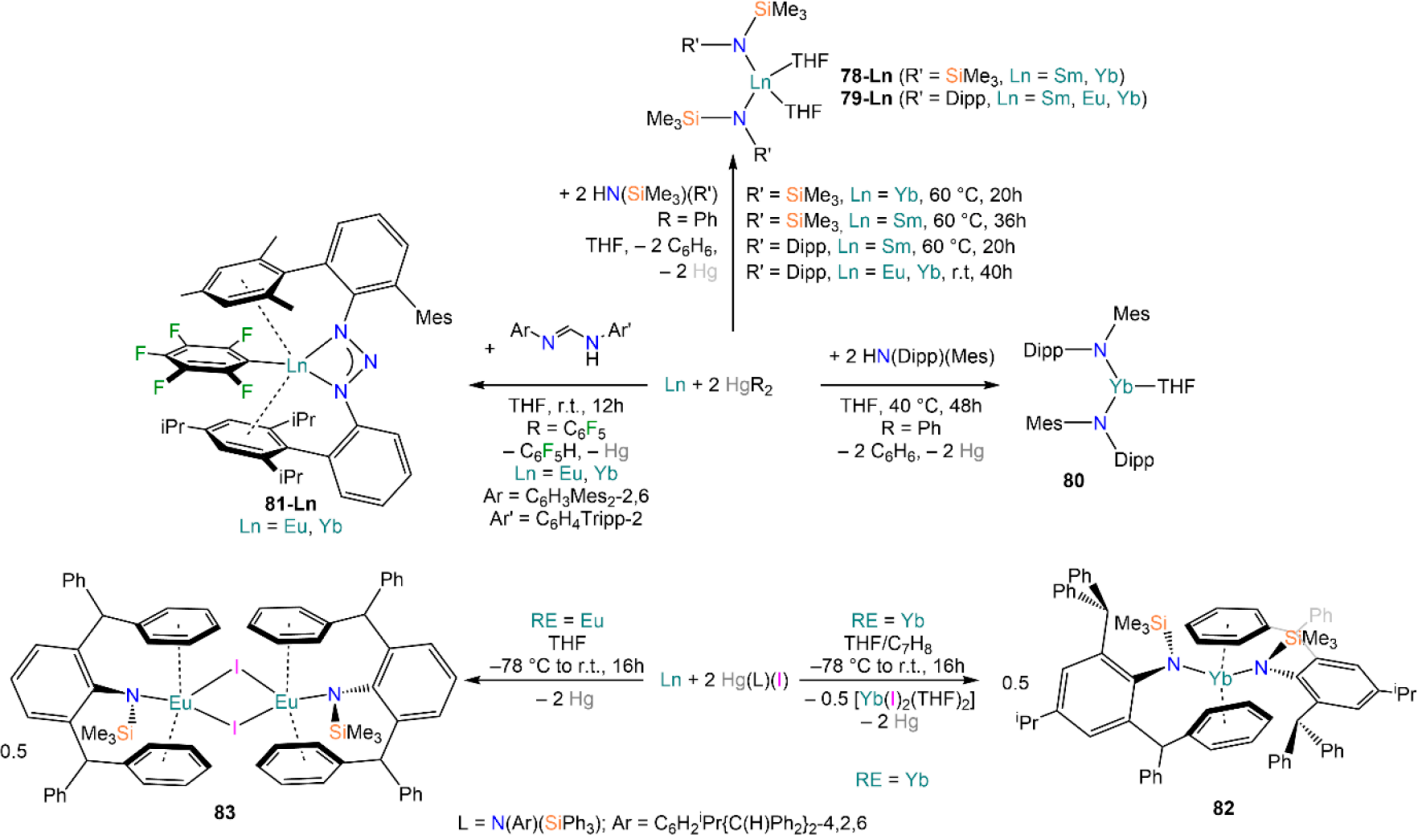
RTP and Direct RT Reactions with Sterically Demanding Amides^182,212,213^

Deacon and co-workers demonstrated that RTP methodologies can be of great utility for the stabilization of complexes with sterically demanding amides. Amines, HN(SiMe_3_)_2_, and HN(SiMe_3_)(Dipp) react smoothly with Sm, Eu, and Yb in THF in the presence of HgPh_2_, to give Ln(II) bis-amide complexes [Ln{N(SiMe_3_)_2_}_2_(THF)_2_] (**78-Ln**·2THF) and [Ln{N(SiMe_3_)(Dipp)}_2_(THF)_2_] (**79-Ln**).^212^ Deacon, Jones, and co-workers have also shown that RTP methodologies can afford complexes with very low coordination numbers, reporting the synthesis of 3-coordinate complex [Yb{N(Dipp)(Mes)}_2_(THF)] (**80**) from Yb metal, HgPh_2_, and HN(Dipp)(Mes) (Scheme 30).^182^ Niemeyer and Hauber reacted Eu and Yb powders with triazene HN_3_(Dmp)(Tph) (Dmp = C_6_H_3_Mes_2_-2,6; Tph = C_6_H_4_Tripp-2; Tripp = C_6_H_2_^i^Pr_3_-2,4,6) and Hg(C_6_F_5_)_2_, obtaining the heteroleptic 3-coordinate complexes [Ln{N_3_(Dmp)(Tph)}(C_6_F_5_)] (**81-Ln**; Ln = Eu, Yb).^213^ Additionally, Jones and co-workers showed that heteroleptic organomercurials of the type “Hg(L)(X)” (L = ligand, X = halide) can be employed as direct RT reagents (Scheme 30).^182^ Reaction of Hg(L^†^)(I) (L^†^ = N(Ar)(SiMe_3_); Ar = C_6_H_2_^i^Pr{C(H)Ph_2_}_2_-4,2,6) with metal powders yields two different results: with Yb, the homoleptic 2-coordinate complex [Yb(L^†^)_2_] (**82**) is obtained, with concomitant formation of [Yb(I)_2_(THF)_2_]; in the case of Eu, the halide-bridged dimer [{Eu(L^†^)(μ-I)(THF)}_2_] (**83**) is obtained instead. Analogous reactions with Sm and Tm were also attempted, but without success.

**Scheme 31:**
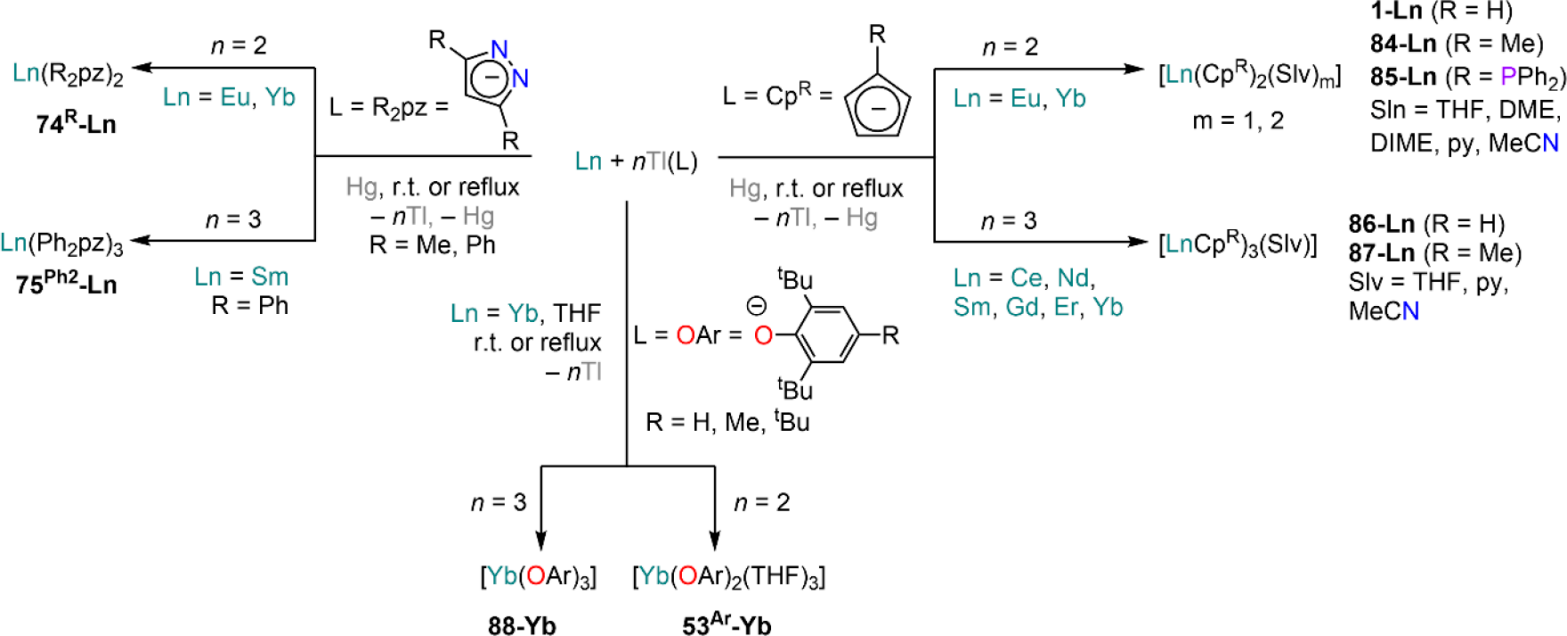
Synthesis of Cp,^168,169,172,173,215^ Pyrazolate,^174,206^ and Aryloxide,^91,170,198^ Ln Complexes *via* RT Reactions with Tl(I) Reagents

The use of Tl reagent in RT methodologies is also very well-established. Tl^+^ is not as oxidizing as Hg^2+^ (*E*^Tl^+^/Tl^ = −0.34 V; *E*^Hg^2+^/Hg^ = 0.85 V),^214^ thus allowing for more control of the reactivity particularly in the case of divalent Lns. Additionally, suitable transmetalation reagents “Tl(L)” are easily accessible by reacting Tl(OEt) with protic substrates such as cyclopentadienes,^168,169,172,173,215^ pyrazoles,^174,206^ and phenols.^91,170,198^ Methodologies are similar to those used for organomercurial RT and RTP reactions, requiring usually ethereal solvents (THF, DME), though pyridine can also be used in some cases.^179^ Additionally, small aliquots of mercury can also be added to these reactions to aid reactivity.^179^ The first compounds to be synthesized with these Tl(I) transmetalation reagents were Cp complexes, Ln(Cp^R^)_2_ (**1-Ln**, Cp^R^ = Cp; **84-Ln**, Cp^R^ = Cp^Me^; **85-Ln**, Cp^R^ = Cp^PPh2^; RE = Eu, Yb) and Ln(Cp^R^)_3_ (**86-Ln**, Cp^R^ = Cp; **87-Ln**, Cp^R^ = Cp^Me^; RE = Ce, Nd, Sm, Gd, Er, Yb) (Scheme 31).^168,169,172,173,215^ Despite the toxicity of Tl, Tl(I) reagents offer some advantages with respect to organomercurials, owing to their higher chemical and thermal stability and tolerance toward a wider variety of solvents (*e.g.*, pyridine and MeCN). It is noteworthy that the synthesis of divalent derivatives with Yb, **1-Yb** and **84-Yb**, often requires large excess of metal powder to avoid formation of trivalent **86-Yb** or **87-Yb**; however, in the case of Sm, only trivalent complexes **86-Sm** and **87-Sm** are obtained even in the presence of excess metal.^169,215^ Deacon and co-workers monitored the reaction between Yb powder and Tl(Cp) by IR spectroscopy, which revealed that the reaction proceeds first with the formation of **84-Yb** followed by reduction with the excess Yb metal to yield divalent **1-Yb**.^179^ Deacon^170,198^ and Lappert^91^ reported also the application of Tl(I) RT reactions to the synthesis of sterically encumbered aryloxides, [Yb(OAr)_2_(THF)_3_] (**53**^**Ar**^**-Yb**) and Yb(OAr)_3_ (**88-Yb**) (Ar = C_6_H_3_^t^Bu_2_-2,6, C_6_H_2_^t^Bu_2_-2,6-Me-4, C_6_H_2_^t^Bu_3_-2,4,6) (Scheme 31).^91^ Similar to what was reported for the synthesis of Cp complexes, a large excess of Yb is required to access divalent derivatives. In addition, pyrazolate complexes, RE(R_2_pz)_2_ (**74**^**R**^**-Ln**, R = Me, Ph), are readily accessible for Eu and Yb, while reaction of Sm powder with Tl(Ph_2_pz) affords trivalent Sm(Ph_2_pz)_3_ (**75**^**Ph2**^**-Sm**) even under strict stoichiometric control (Scheme 31).^174,206^

**Scheme 32:**
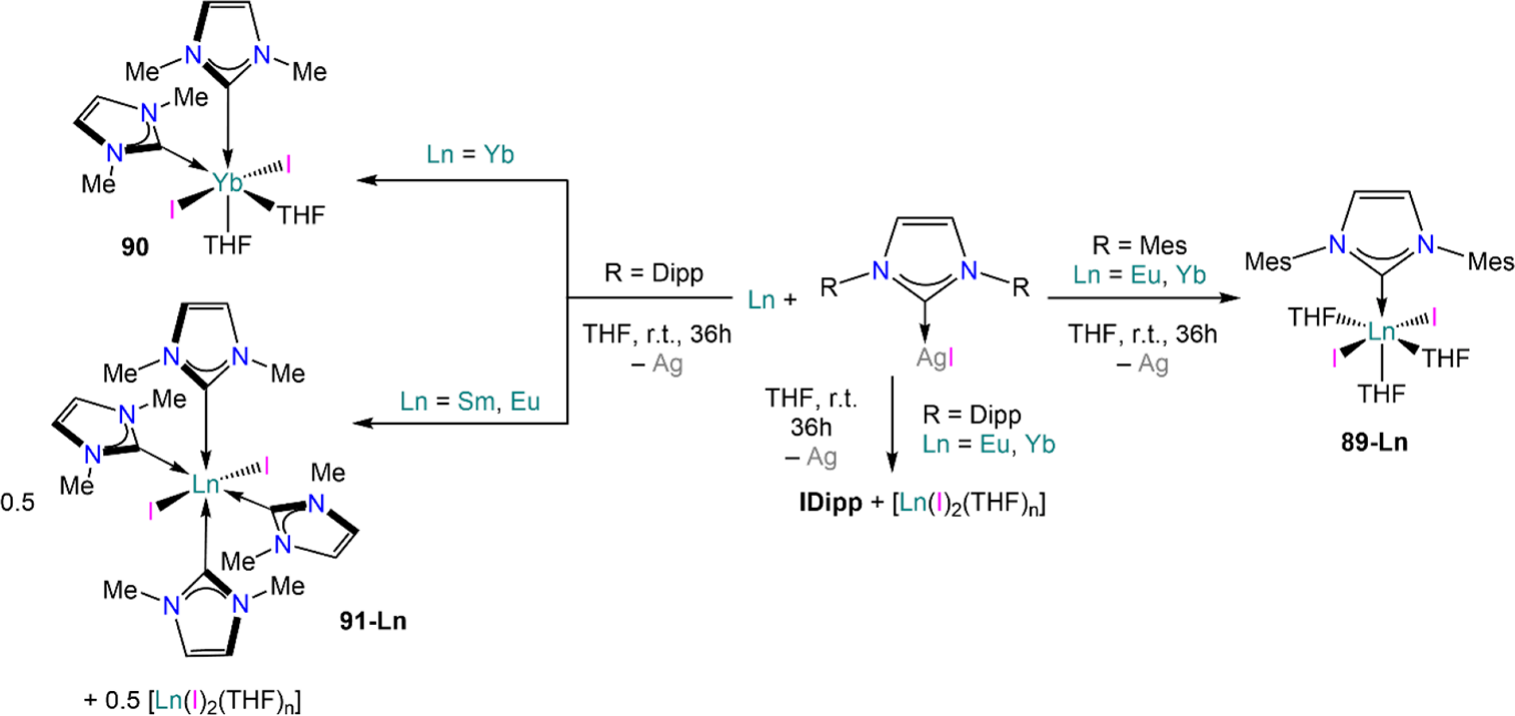
RT Reactions with Ag(I)-NHC Reagents by Roesky and Co-workers^195,196^

Bi and Ag reagents, BiPh_3_, [Bi(C_6_F_5_)_3_], and [Ag(C_6_F_5_)(py)], have also been used as alternatives to Hg and Tl in transmetalation reactions, though their application so far has been more limited compared to Hg and Tl reagents.^175−177^ BiPh_3_ was used by Bochkarev *et al.* to prepare [Er(Ph)_3_(THF)_3_] in good yields from Er powder and with a small quantity of ErI_3_ (4%).^176^ More recently, Deacon, Junk, and co-workers have demonstrated that [Bi(C_6_F_5_)_3_]^175^ and [Ag(C_6_F_5_)(py)]^177^ can be used in RTP reactions for the synthesis of RE pyrazolate and formamidinate complexes,^154^ thus proving that these species could provide an attractive alternative to the more toxic Hg and Tl regents. Roesky and co-workers have also shown that RT reactions with Ag(I)-NHC reagents can be employed for the synthesis of heteroleptic Ln-NHC complexes (Scheme 32).^195,196^ In these methodologies, Ag salts “(NHC)Ag(I)” [NHC = 1,3-bis(R)imidazolin-2-ylidene; R = Me (IMe_2_), Mes (IMes), and Dipp (IDipp)] are reacted with Sm, Eu or Yb powders in THF at room temperature, and the outcome of these reactions is dictated by the steric demands of the NHC ligand employed and the ionic radii of the metal centers. With the most sterically demanding IDipp, the products of this reaction are free carbene and [Ln(I)_2_(THF)_*n*_] (RE = Eu, Yb).^195^ On the other hand, when the smaller IMes ligand is employed, the heteroleptic NHC complexes [Ln(IMes)(I)_2_(THF)_3_] (**89-Ln**; Ln = Eu, Yb) are obtained.^195^ Finally, the reaction between (IMe_2_)Ag(I) and Yb affords the bis-NHC adduct [Yb(IMe_2_)_2_(I)_2_(THF)] (**90**), while the tetra-NHC adducts [Ln(IMe_2_)_4_(I)_2_] (**91-Ln**; Ln = Sm, Eu) are obtained with the larger divalent lanthanoids.^196^

**Scheme 33:**
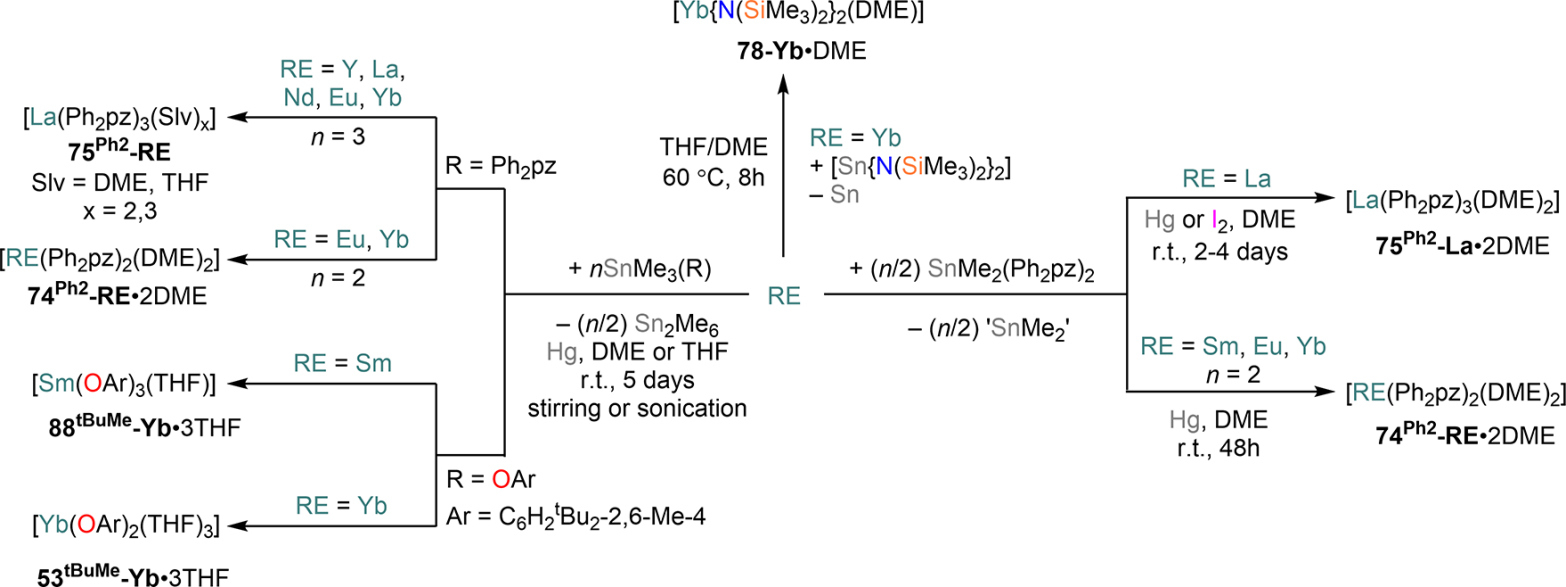
Use of Sn(II) and Sn(IV) Reagents in RT Reactions^90,166,167^

Sn reagents have also been employed in RT reactions. This methodology was first introduced into RE chemistry by Lappert and co-workers who reacted Yb powder with [Sn{N(SiMe_3_)_2_}_2_] in THF under reflux and obtained the bis-silylamide complex [Yb{N(SiMe_3_)_2_}_2_(DME)] (**78-Yb**·DME) upon crystallization from DME (Scheme 33).^90^ Lappert’s methodology involved a Sn(II)-to-Sn(0) reduction, whereas Deacon and co-workers later employed Sn(IV) reagents—SnMe_3_(Ph_2_pz), SnMe_3_(OAr) (Ar = C_6_H_2_^t^Bu_2_-2,6-Me-4), and SnMe_2_(Ph_2_pz)_2_—for the synthesis of RE pyrazolate and aryloxides, *i.e.*, RE(Ph_2_pz)_2_ (**74**^**Ph2**^**-RE**), RE(Ph_2_pz)_3_ (**75**^**Ph2**^**-RE**), RE(OAr)_2_ (**53**^**tBuMe**^**-Ln**), and RE(OAr_3_) (**88**^**tBuMe**^**-Ln**, Scheme 33).^166,167^ While the first two reagents afford a one-electron reduction, Sn(IV)/(III),^167^ forming Sn_2_Me_6_ as byproduct, the latter behaves as a two-electron oxidizing agent generating putative “SnMe_2_” (Scheme 33).^166^

### 3.5 Metal Vapor Synthesis

**Figure 3:**
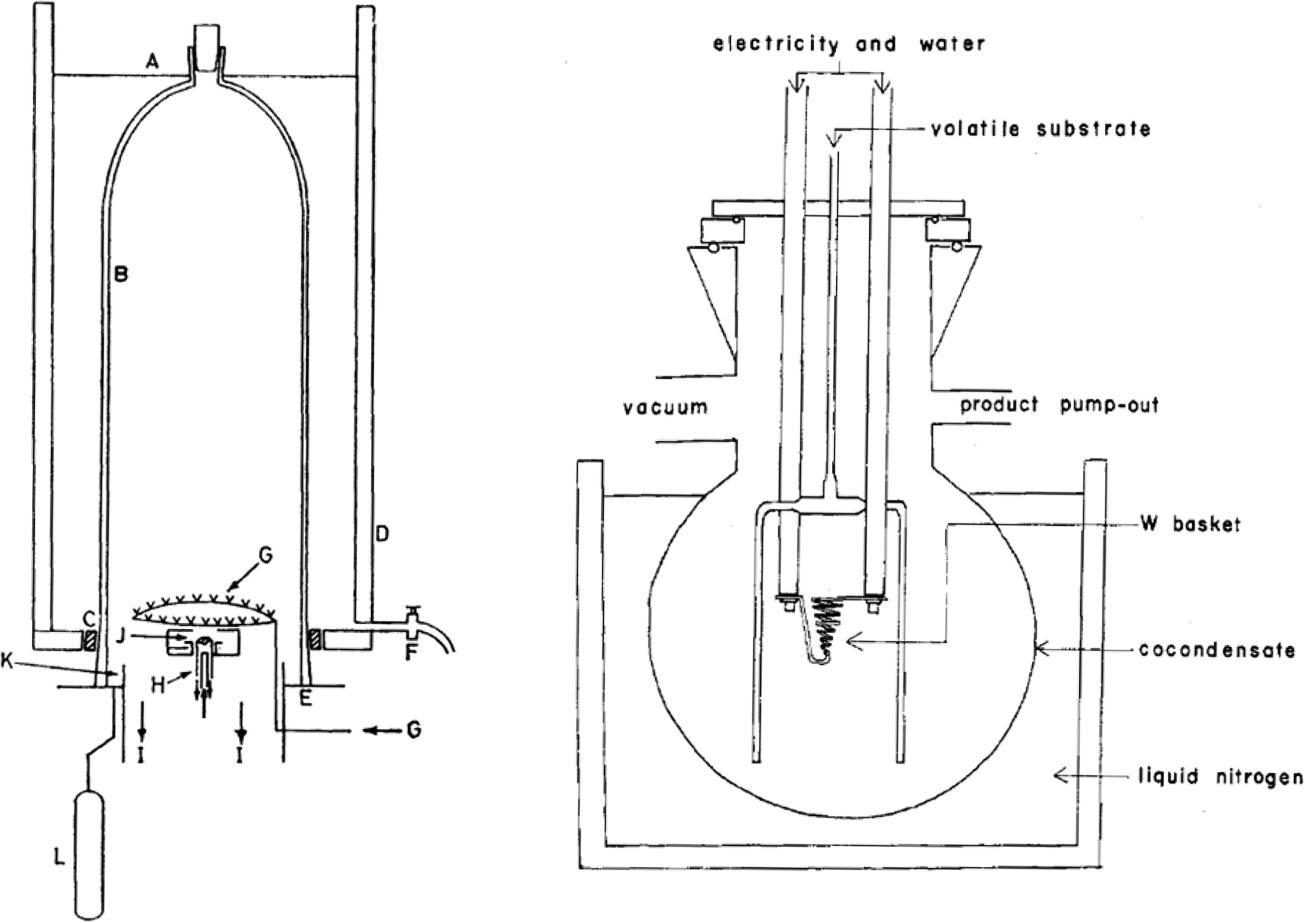
Examples of MVS reactors employed by Cloke (left) and DeKock (right). Parts in the reactor used by Cloke are **A**, coolant level; **B**, glass reaction vessel; **C**, gasket; **D**, insulated container for coolant; **E**, ground flange seating for Viton O-ring; **F**, coolant drain; **G**, ligand vapor inlet, “gas-ring”; **H**, electron-beam furnace; **I**, outlet to trap and diffusion pump; **J**, metal sample; **K**, gutter for collection of products; **L**, vessel for product receipt. Panel on the left reproduced with permission from ref (217). Copyright 1981 Royal Society of Chemistry Publishing. Panel on the right reproduced from ref (222). Copyright 1978 American Chemical Society.

The use of MVS techniques is not a very common methodology in RE coordination and organometallic chemistry and has been employed by very few research teams across the world.^13^ However, its application has led to some remarkable results such as the first synthesis of [Sm(Cp*)_2_(THF)_2_] (probably one of the most iconic and well-studied f-element complexes ever reported),^216^ the isolation of the first zerovalent RE molecular species [RE(_3_C_6_H_3_^t^Bu_3_-1,3,5)_2_] (RE = Y, La, Pr, Nd, Sm, Gd, Tb, Dy, Ho, Er, Lu),^217−219^ and the first examples of Sc(II) and Sc(I) complexes, [RE(C_6_H_3_^t^Bu_3_-1,3,5){1-CH_2_C(Me)_2_3,5-^t^Bu_2_C_6_H_3_}(H)]^220^ and [{Sc(η^5^-^t^Bu_2_C_2_P_3_)}_2_(μ-η^6^:η^6^-^t^Bu_3_C_3_P_3_)].^221^ With this technique, metal vapor is generated at low temperatures (−196 °C) under high vacuum and condensed with a substrate (Figure 3), and the resulting reaction mixture is then extracted in an organic solvent for recrystallization.^13,40,222^

**Scheme 34:**
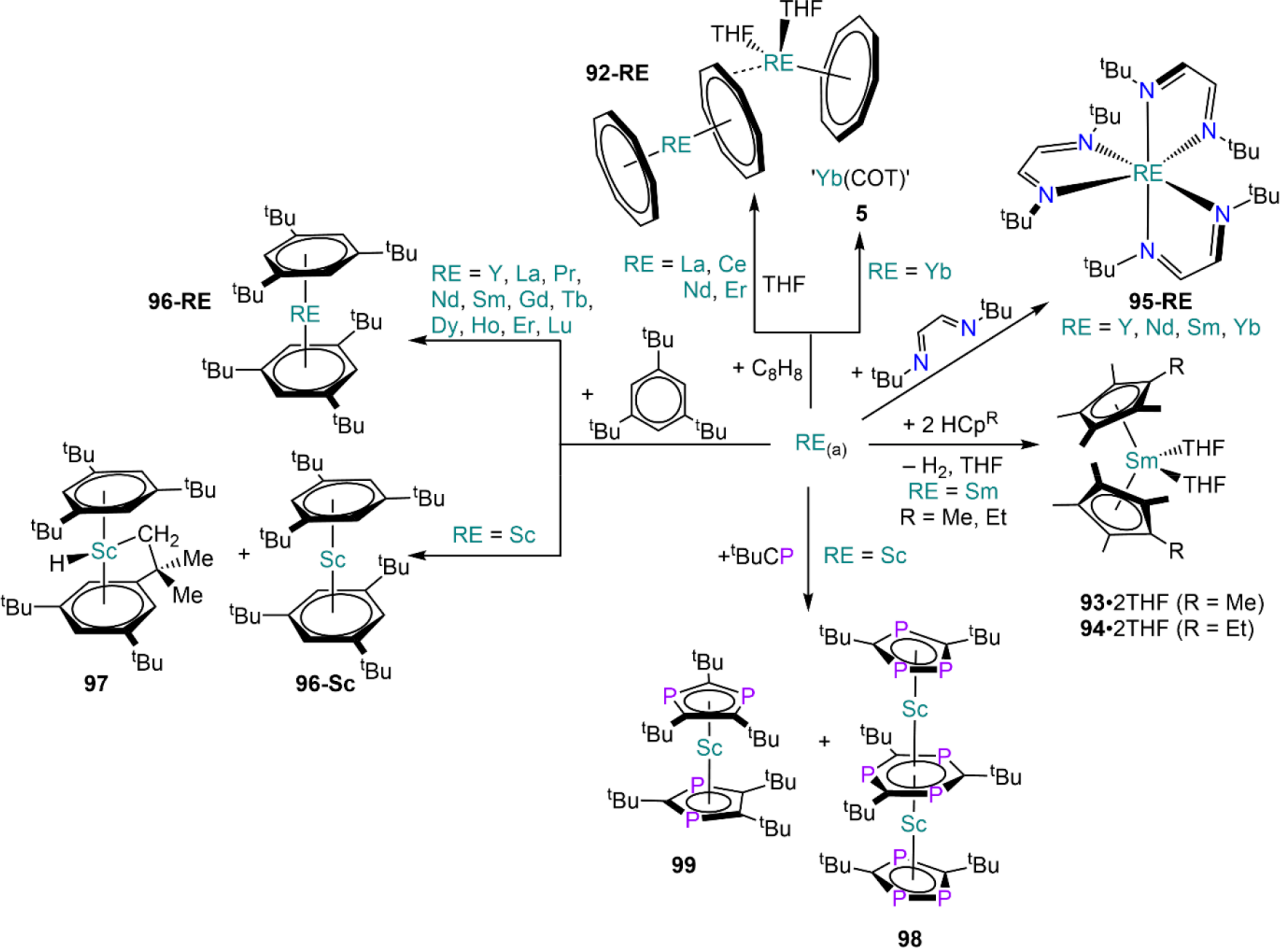
Examples of RE Complexes Obtained *via* Metal Vapor Co-condensation Methods^216−219,221,222,226,229^

The first reports on the reaction between RE metal vapors and organic substrates (*e.g.*, 1-hexyne, 3-hexyne, 1,3-butadiene, 2,4-pentanedione, and cyclooctatetraene) were presented by Blackborow,^223^ Evans,^224,225^ and DeKock.^222,226^ By reacting RE metal atoms (RE = La, Ce, Nd, Er) with C_8_H_8_ (Scheme 34), DeKock and co-workers isolated a family of asymmetric complexes of formula [RE(COT)(THF)_2_][RE(COT)_2_] (**92-RE**; RE = La, Ce, Nd, Er), while reactivity with Yb afforded “Yb(COT)” (**5**), previously obtained from reaction in liquid NH_3_ (*vide supra*, Scheme 4).^222,226^ Evans and co-workers successfully applied this technique to the preparation of [Sm(Cp*)_2_(THF)_2_] (**93**·2THF) and [Sm(Cp^Me4Et^)_2_(THF)_2_] (**94**·2THF; Cp^Me4Et^ = {C_5_EtMe_4_}^−^) by reacting Sm atoms with functionalized cyclopentadienes (Scheme 34).^216,227^ In general, products obtained from reactivity of RE atoms with unsaturated substrates show magnetic properties that are in agreement with the presence of oxidized RE ions (either +2 or +3) despite the formal 0 oxidation state,^13^ such as in the diazabutadiene complexes RE[{N(^t^Bu)CH}_2_]_3_ (**95-RE**; RE = Y, Nd, Sm, Yb) reported by Cloke and co-workers (Scheme 34).^228^ On the other hand, reaction of RE atoms with 1,3,5-*tri*-tertbutylbenzene, 1,3,5-^t^Bu_3_C_6_H_3_, generates sandwich complexes of general formula [RE(C_6_H_3_^t^Bu_3_-1,3,5)_2_] (**96-RE**; RE = Sc, Y, La, Pr, Nd, Sm, Gd, Tb, Dy, Ho, Er, Lu) where magnetic characterization reveals that the RE metals are unequivocally in a zerovalent state (Scheme 34).^217−219^ When the same reaction is performed with Sc atoms, together with the Sc(0) complex [Sc(C_6_H_3_^t^Bu_3_-1,3,5)_2_] (**96-Sc**) a C–H activation product is also isolated, [Sc(C_6_H_3_^t^Bu_3_-1,3,5){1-CH_2_C(Me)2–3,5-^t^Bu_2_C_6_H_3_}(H)] (**97**), in which the metal center is formally in the +2 oxidation state (Scheme 34).^220^ Cloke and co-workers extended this synthetic methodology to cyclization reactions involving phospalkyne ^t^BuCP.^221,229^ Trimerization of ^t^BuCP promoted by Sc atoms generated the triphosphabenzene ring C_3_P_3_^t^Bu_3_, together with formation of phospholyl ligand {C_2_P_3_^t^Bu_2_}^−^ and assembling of the double-decker Sc(I) complex [{Sc(η^5^-C_2_P_3_^t^Bu_2_)}_2_(μ–η^6^:η^6^-C_3_P_3_^t^Bu_3_)] (**98**, Scheme 34).^221^ A byproduct of the same reaction is also the scandocene complex [Sc(η^5^-C_3_P_2_^t^Bu_3_)_2_} (**99**), which was at the time only the second Sc(II) complex ever reported.^229^

## 4 Halides

RE halides are very common starting materials in RE coordination and organometallic chemistry. Divalent salts used in synthetic chemistry are usually diiodides of classic divalent Lns (Sm, Eu, and Yb), with the addition of Nd, Dy, and Tm. Both divalent and trivalent salts, LnI_2_ and REX_3_ are classically employed in salt metathesis reactions and with a variety of ligand sets. In this section some examples of their reactivity will be covered.

### 4.1 Divalent Ln Halides

Divalent halides of the Lns, LnX_2_ (Table 3), have been known for over a century, obtained primarily as diiodides.^230^ However, fluorides, chlorides, and bromides are also reported for classic divalent Lns (Sm, Eu, and Yb) with the addition in some cases of Nd, Dy, and Tm.^6,9^ Noticeably, ScI_2_ has also been reported, though it contains impurities of ScI_3_ and is best described as Sc_0.9_I_2_.^15,231,232^ Out of all these halides, only a handful of them are of some synthetic utility in coordination and organometallic chemistry. This is due to the different physicochemical behavior of binary halides: for dihalides of Nd, Sm, Eu, Dy, Tm, and Yb the metal is in a true divalent state similar to group 2 metals (RE^2+^), while in the case of the other REs these could be best described as trivalent metals with an electron residing in the 5d band, *i.e.*, RE^3+^(e^–^) (3d band in the case of Sc and 4d band in the case of Y).^15^ Nonetheless, NdI_2_ DyI_2_ and TmI_2_ are very reducing and difficult to handle, which poses challenges for their application in synthetic chemistry. In this section we will cover dihalides employed in synthetic chemistry, focusing primarily on the iodides as they are the ones that have found most widespread applications in synthetic laboratories.^42^

**Table 3:** Selected Examples of Solvent-free and Solvated LnI_2_ Salts Used in Synthesis^a^

	Cl	Br	I
Nd			NdI_2_^244^
			[Nd(I)_2_(THF)_5_]^254^
Sm	SmCl_2_^259^	SmBr_2_^260^	SmI_2_^262^
	*SmCl*_*2*_*(THF)*_*x*_(250)	*SmBr*_*2*_*(THF)*_*x*_(250,261)	[Sm(I)_2_(THF)_2_]^245^
			[Sm(I)_2_(THF)_5_]^249^
Eu	EuCl_2_^263^	EuBr_2_^241,263,264^	EuI_2_^241^
		[Eu(Br)_2_(THF)_2_]^247^	[Eu(I)_2_(THF)_2_]^245^
Dy			DyI_2_^244^
			[Dy(I)_2_(THF)_5_]^254^
			[Dy(I)_2_(DME)_3_]^254^
Tm			TmI_2_^244^
			[Tm(I)_2_(DME)_3_]^252,253^
			[Tm(I)_2_(THF)_5_]^252,253^
Yb	YbCl_2_^259,265,266^	YbBr_2_^266,267^	YbI_2_^268,269^
		[Yb(Br)_2_(THF)_2_]^247^	[Yb(I)_2_(THF)_2_]^245^
			[Yb(I)_2_(THF)_4_]^270^

a Compounds in italics have not been isolated.

**Scheme 35:**
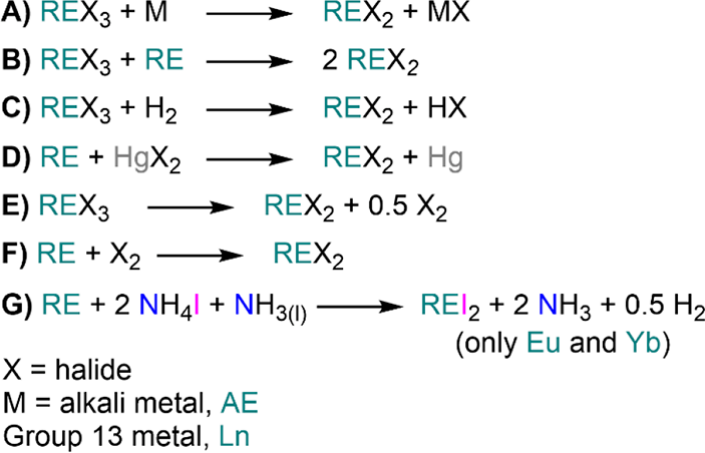
Preparation of Solvent-free RE Dihalides

Solvent-free dihalides can be obtained using a variety of methodologies which usually entail solid-state high-temperature methods.^15,233−236^ These are convenient starting materials whenever ethereal solvents are avoided, either for stabilization of complexes with low coordination numbers or because of potential side reactivity.^7,42^ Solid-state methodologies to obtain solvent-free REX_2_ salts comprise (1) metallothermic reduction (Wohler method, Scheme 35, **A**);^15,237^ (2) comproportionation of trivalent halides with metallic RE (Scheme 35, **B**);^238,239^ (3) reduction of trivalent halides with hydrogen (Scheme 35, **C**);^230,240^ (4) reaction of RE metal with HgX_2_, where X is usually an iodide (Scheme 35, **D**);^239^ (5) thermal decomposition (Scheme 35, **E**); and (6) direct reaction between elemental halogen and RE metal (Scheme 35, **F**).^15,236,241^ Additionally, solvent-free EuI_2_ and YbI_2_ can be obtained from the reaction of metal powders with NH_4_I in liquid ammonia (Scheme 35, **G**).^82,241^

**Figure 4:**
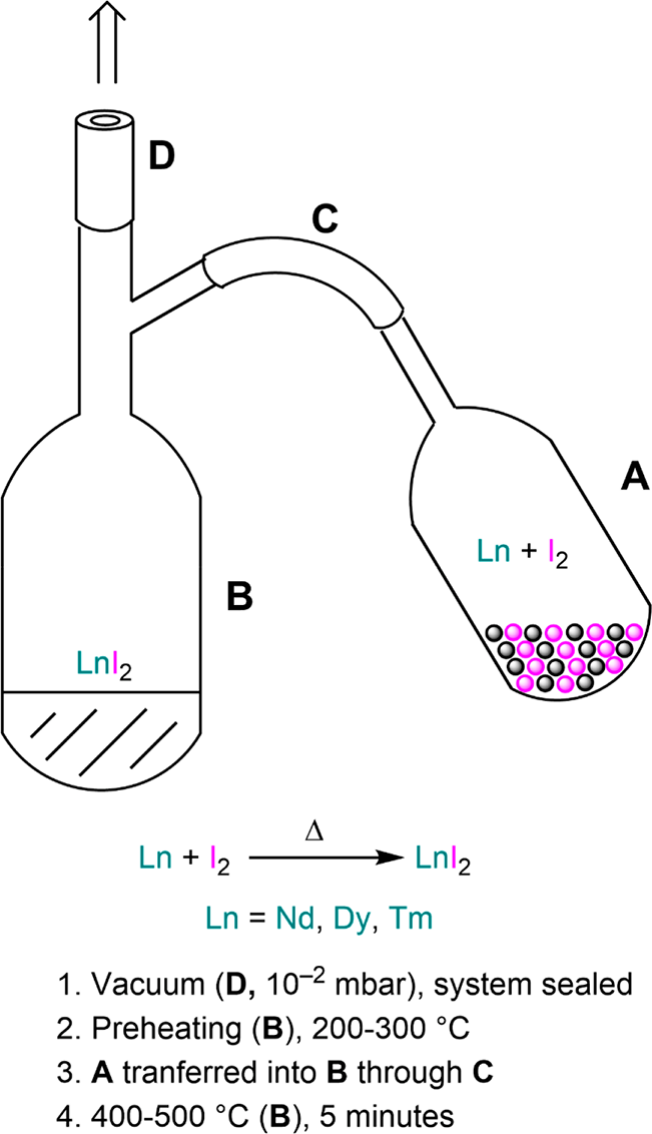
Schematic representation of the apparatus for the synthesis of solvent-free LnI_2_ salts used by Bochkarev and co-workers. **A** and **B**, thick-walled ampules; **C**, rubber tubing; **D**, rubber tubing to vacuum line.^242,243^ Adapted with permission from ref (243). Copyright 2003 Elsevier.

With the exception of liquid ammonia synthesis (method **G**, Scheme 35), these methods usually require specialized equipment classically used for solid-state synthesis and are unsuitable for standard synthetic laboratories.^15^ Bochkarev and Evans were able to adapt solid-state methods originally developed by Corbett, Kruse, and others and devised an approach which can be used in normal laboratory conditions (method **F**, Scheme 35).^242−244^ Bochkarev and Fagin initially reported the synthesis of NdI_2_, DyI_2_, and TmI_2_ using a simple apparatus consisting of two thick-walled glass ampules connected to a vacuum manifold (Figure 4).^242,243^ In this methodology, after charging ampule **A** with metal shavings and iodine, the system is thoroughly evacuated and then sealed off. Following this, the starting materials are gradually dispensed from ampule **A** to ampule **B** (while **A** is kept at room temperature throughout the operation, **B** has been preheated to 200–300 °C with a gas burner), followed by heating at 400–500 °C for a maximum of 5 min, generating the desired LnI_2_ with no further purifications. The authors also noted that after initiation molten metal was formed, thus indicating that the reaction core reached temperatures around 1500 °C.^242,243^ In Bochkarev’s original method the ampules are reported to be made of thick-walled borosilicate glass; however, owing to the high temperatures necessary for the initiation of the reaction and the heat subsequently generated, it is recommended that quartz reactors be used for this setup instead.

**Figure 5:**
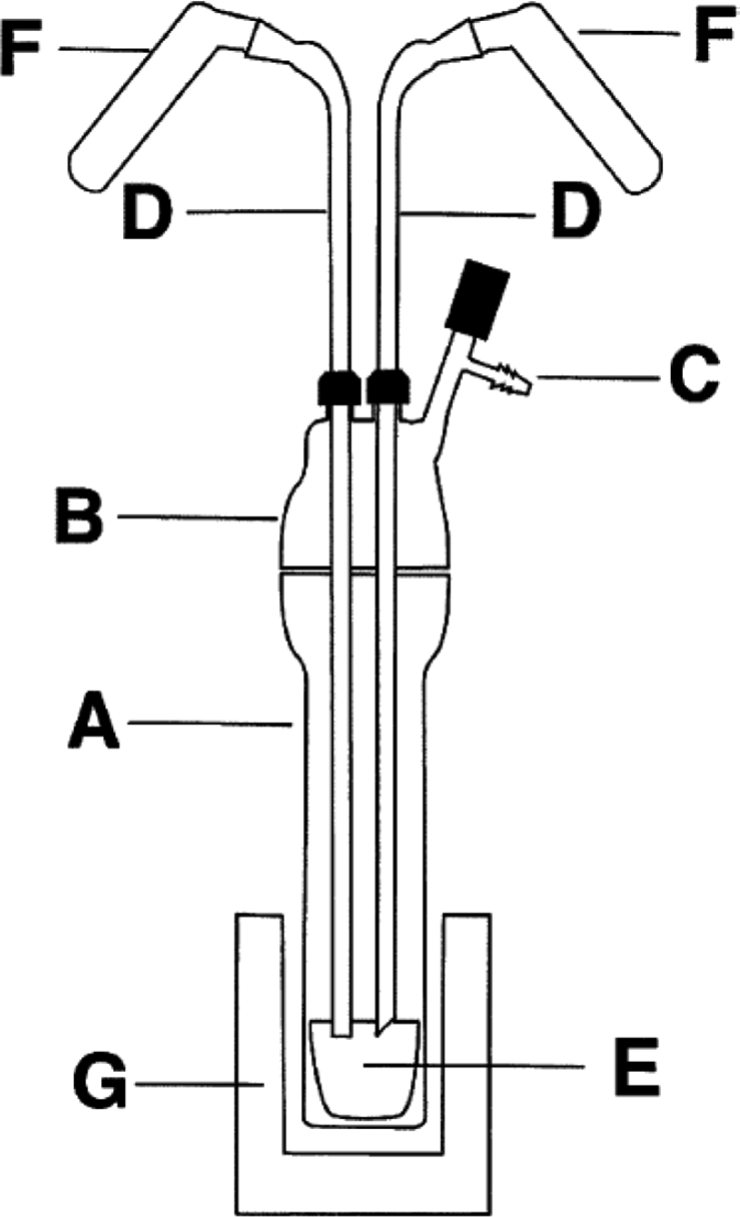
Apparatus for the synthesis of solvent-free LnI_2_ salts used by Evans and co-workers. **A**, quartz tube; **B**, O-ring joint; **C**, connection to vacuum line; **D**, quartz addition tubes; **E**, quartz crucible; **F**, addition funnel; **G**, furnace. Reproduced from ref (244). Copyright 2003 American Chemical Society.

Evans and co-workers developed a more sophisticated apparatus which can produce LnI_2_ salts in large quantities (up to 50 g) and with more controlled conditions (Figure 5).^244^ In Evans’ method, Ln metal powders (40 mesh) and iodine are placed in separate addition funnels (**F**) and the whole system is kept under static vacuum. The reactor is then heated to 450 °C inside a furnace, and the starting materials are added gradually into a quartz crucible (**E**) placed at the bottom of the reactor. After an initiation period, the temperature is raised above the melting point of the relative LnI_2_ (Ln = Nd 562 °C, Sm 520 °C, Eu 510 °C, Dy 659 °C, Tm 756 °C, Yb 772 °C),^9^ and the reagents are added portion-wise over a period of 2 h, giving the desired LnI_2_ material.

**Scheme 36:**
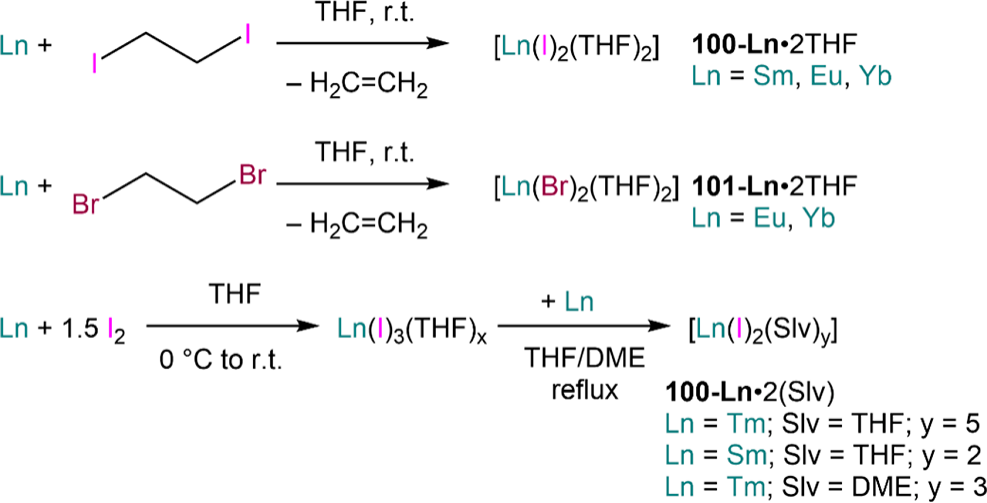
Synthesis of Solvated LnX_2_ Salts **100-Ln**·2THF (Ln = Sm, Eu, Yb),^246−248^**101-Ln**·2THF (Ln = Eu, Yb),^247^**100-Tm**·5THF and **100-Tm**·3DME^252,253^

Solvated iodide salts, [Ln(I)_2_(Slv)_*x*_] (**100-Ln**; Slv = THF, DME; *x* = 2–5) of divalent Lns can also be obtained *via* standard solution methods (Scheme 36). For the most stable divalent Lns (Sm, Eu, and Yb), the main methodology was developed by Kagan and co-workers for the synthesis of [Sm(I)_2_(THF)_2_] (**100-Sm**·2THF),^245^ which consists of direct reaction of Ln metal powder or chips with 1,2-diiodoethane in THF.^246−248^ Evans and co-workers crystallographically characterized the 7-coordinate THF adduct [Sm(I)_2_(THF)_5_],^249^ but the bis-THF adduct **100-Sm**·2THF is the predominant species after standard workups following Kagan’s methodology. In a similar fashion, Watson *et al.* reported the synthesis of [Eu(Br)_2_(THF)_2_] (**101-Eu**·2THF) and [Yb(Br)_2_(THF)_2_] (**101-Yb**·2THF), obtained from the reaction between Ln metal powders and 1,2-dibromoethane;^247^ this methodology cannot be applied to the synthesis of solvated SmBr_2_, which is obtained from the reduction of SmBr_3_ or substitution of SmI_2_.^250^ Solvated SmI_2_ can also be obtained from the comproportionation reaction of SmI_3_ with Sm metal;^251^ this method has also been successfully applied to the preparation of solvated TmI_2_, which can be then isolated as either THF or DME adduct, [Tm(I)_2_(THF)_5_] (**100-Tm**·5THF) and [Tm(I)_2_(DME)_3_] (**100-Tm**·3DME).^252,253^ Solvent-free NdI_2_ and DyI_2_ can also be converted into THF or DME adducts, [Ln(I)_2_(THF)_5_] (**100-Nd**·5THF) and [Dy(I)_2_(DME)_3_] (**100-Dy**·3DME), though manipulations have to be carried out at low temperature owing to the propensity of these species to react with ethereal solvents.^254^ Adducts of SmI_2_ and YbI_2_ with *N-*heterocycles (pyridine, lutidine, and 4-*tert*-butylpyridine) have also been reported by Sella and Maunder,^255^ while Wakatsuki and Hou isolated also the hexamethylphoshoramide (HMPA) adducts [Sm(I)_2_(HMPA)_4_] and [I]_2_[Yb(HMPA)_4_(THF)_2_].^256^ Finally, crown ether adducts of various LnI_2_ salts have also been reported: Xémard *et al.* reacted **100-Tm**·3DME with 18-crown-6 to obtain the adduct [Tm(I)_2_(18-crown-6)],^257^ while Huh *et al.* successfully attempted the encapsulation of LnI_2_ salts with 2.2.2-cryptand in DMF to give cationic adducts [I]_2_[Ln(crypt)(DMF)_*n*_] (Ln = Sm, Eu, *n* = 2; Ln = Yb, *n* = 1; crypt = 2.2.2-cryptand).^258^

**Scheme 37:**
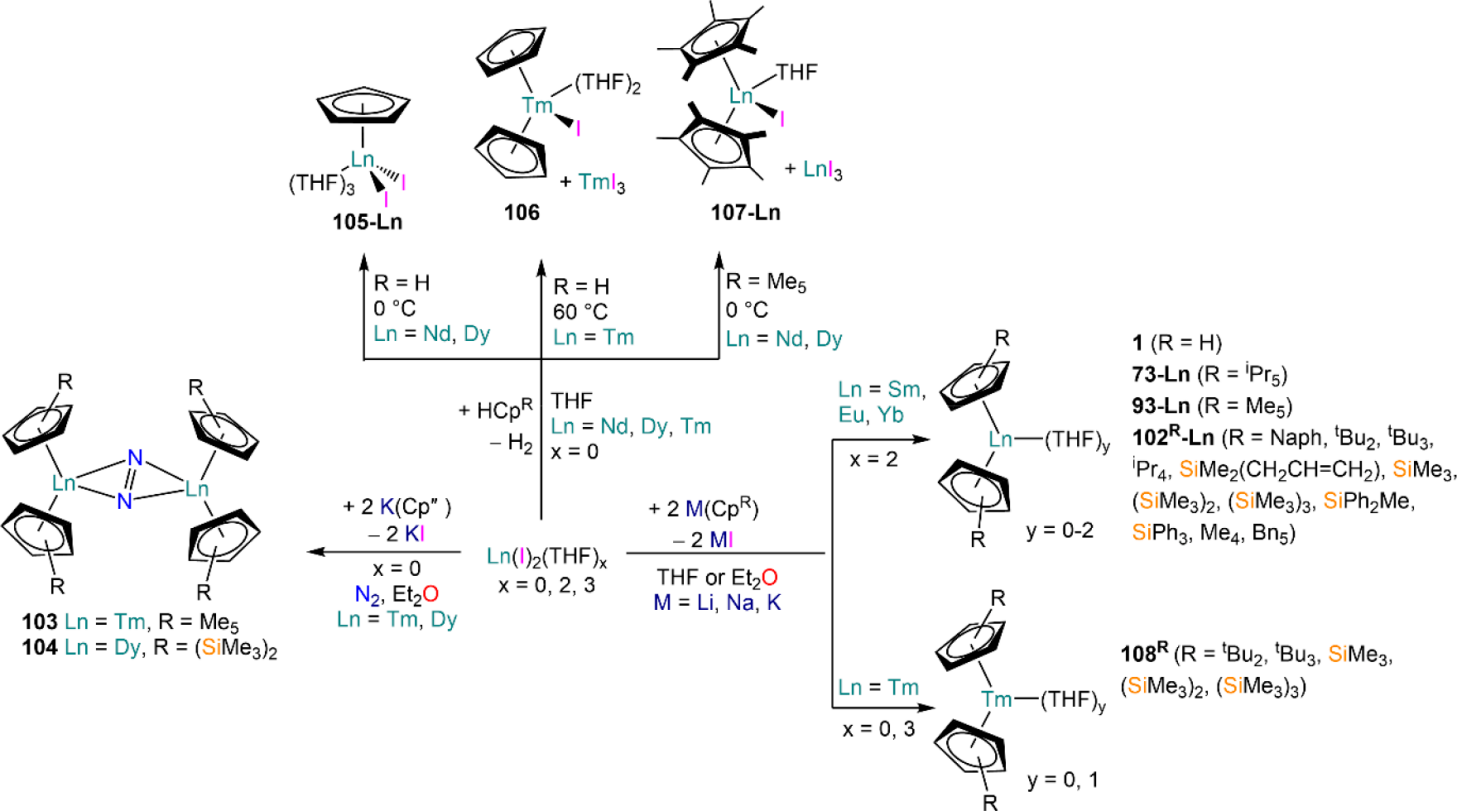
Reactivity of LnI_2_ (Ln = Nd, Sm, Eu, Dy, Tm, Yb) with Cyclopentadienes and Cyclopentadienyl Metal Salts^247,271,273−279,282,283^

**Figure 6:**
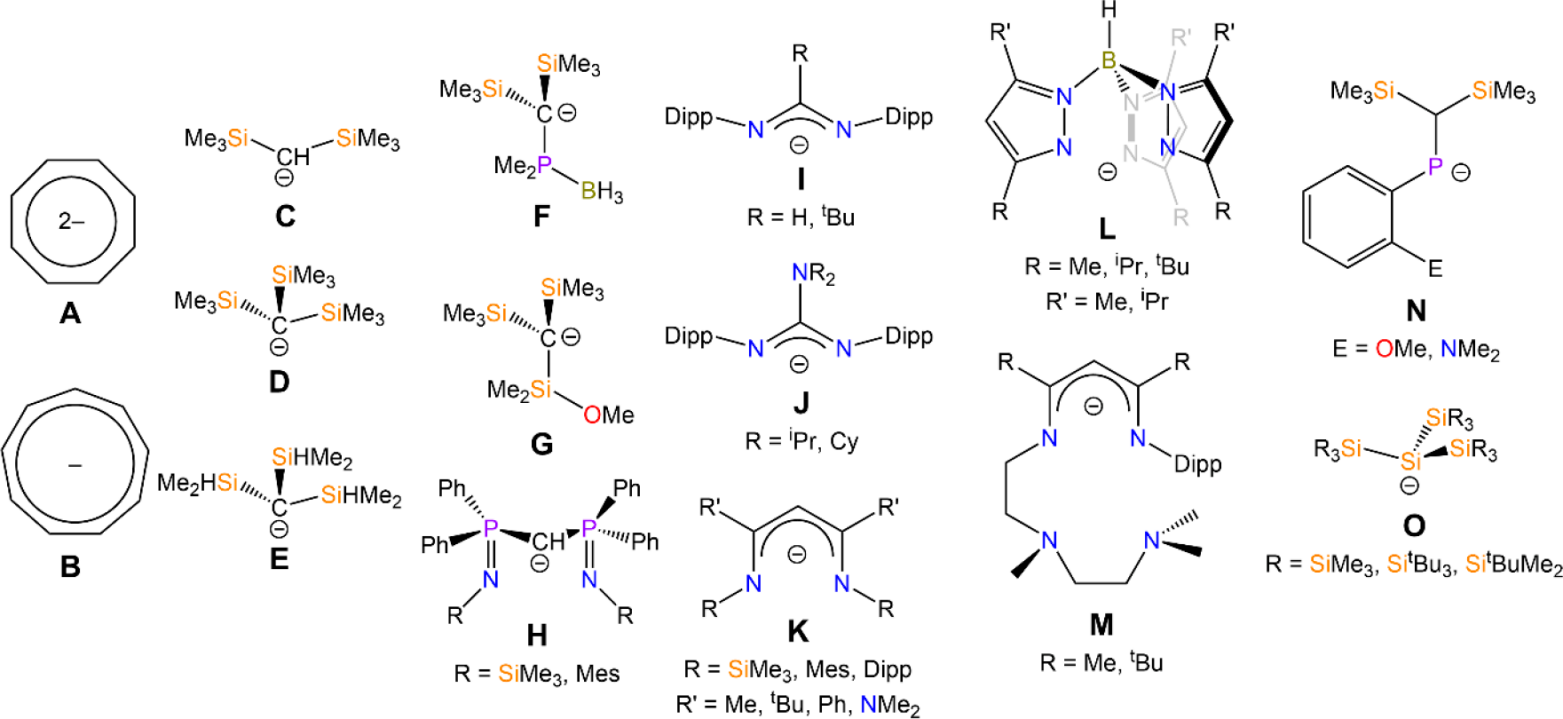
Selected examples of ligands used in salt elimination reactions with Ln diiodides.

LnI_2_ salts and their adducts are commonly used in salt metathesis reactions and are particularly effective for the synthesis of divalent Sm, Eu, and Yb complexes with a variety of supporting ligands (*vide infra*, Figure 6). Most of the complexes obtained with these starting materials are homoleptic derivatives, with varying coordination numbers depending on the presence of coordinated solvents, ligand denticity, and steric properties. Salt elimination reactions have been employed for several decades in the preparation of cyclopentadienyl derivatives, such as the archetypal [Ln(Cp*)_2_(THF)_2_] (**93-Ln**; Ln = Sm, Yb; Slv = Et_2_O, THF).^247,271^ Metallocene-type Ln(Cp)_2_ complexes can be obtained by reacting group 1 cyclopentadienyl salts with SmI_2_, EuI_2_, and YbI_2_ (**93-Yb** can also be obtained from YbCl_2_)^272^ and are isolated either as solvent-free or solvated adducts of formula [Ln(Cp^R^)_2_(Slv)_*x*_] (*x* = 0–2; **1**, Cp^R^ = Cp; **73-Ln**, Cp^R^ = Cp^iPr5^; **93-Ln** Cp^R^ = Cp*; **102**^**R**^**-Ln**, Cp′, Cp″, Cp‴, Cp^Naph^, Cp^SiPh3^, Cp^SiPh2Me^, Cp^tt^, Cp^ttt^, Cp^iPr4^, Cp^Bn5^,)^273−279^ (Cp′ = {C_5_H_3_SiMe_3_}^−^, Cp″ = {C_5_H_3_(SiMe_3_)_2_-1,3}^−^, Cp‴ = {C_5_H_3_(SiMe_3_)_3_-1,2,4}^−^, Cp^tt^ = {C_5_H_3_^t^Bu_2_-1,3}^−^, Cp^ttt^ = {C_5_H_3_^t^Bu_3_-1,3}^−^, Cp^Bn5^ = {C_5_(CH_2_Ph)_5_}^−^) (Scheme 37). These methodologies are more problematic with the highly reducing salts NdI_2_, DyI_2_, and TmI_2_. When TmI_2_ or DyI_2_ are reacted with KCp* or KCp″ under nitrogen, N_2_-activation products [{Tm(Cp*)_2_}_2_(μ-N_2_)] (**103**)^280^ and [{Dy(Cp′)_2_}_2_(μ-N_2_)] (**104**)^277^ are obtained, highlighting the reducing nature of these starting materials. The direct reaction of NdI_2_, DyI_2_, and TmI_2_ with cyclopentadiene (Scheme 37) was also reported by Bochkarev and co-workers, who observed oxidation of the metal to Ln^3+^ and formation of monoring complexes [Ln(Cp)(I)_2_(THF)_3_] (**105-Ln**) with Nd and Dy, whereas the metallocene-type complex [Tm(Cp)_2_(I)(THF)_2_] (**106**) was obtained from the reaction with TmI_3_, with concomitant formation of LnI_3_.^281^ When HCp* was reacted with NdI_2_ and DyI_2_, products of the reaction were metallocenes [Ln(Cp*)_2_(I)(THF)] (**107-Ln**, Ln = −Nd, Dy) (Scheme 37).^281^ Nief and Evans were able to obtain Tm metallocenes [Tm(Cp^R^)(THF)_*x*_] (**108**^**R**^, Cp^R^ = Cp″, Cp‴, Cp^tt^, Cp^ttt^; *x* = 0, 1) *via* salt elimination reaction between TmI_2_ and group 1 salts of sterically demanding Cp ligands.^277,282,283^

**Scheme 38:**
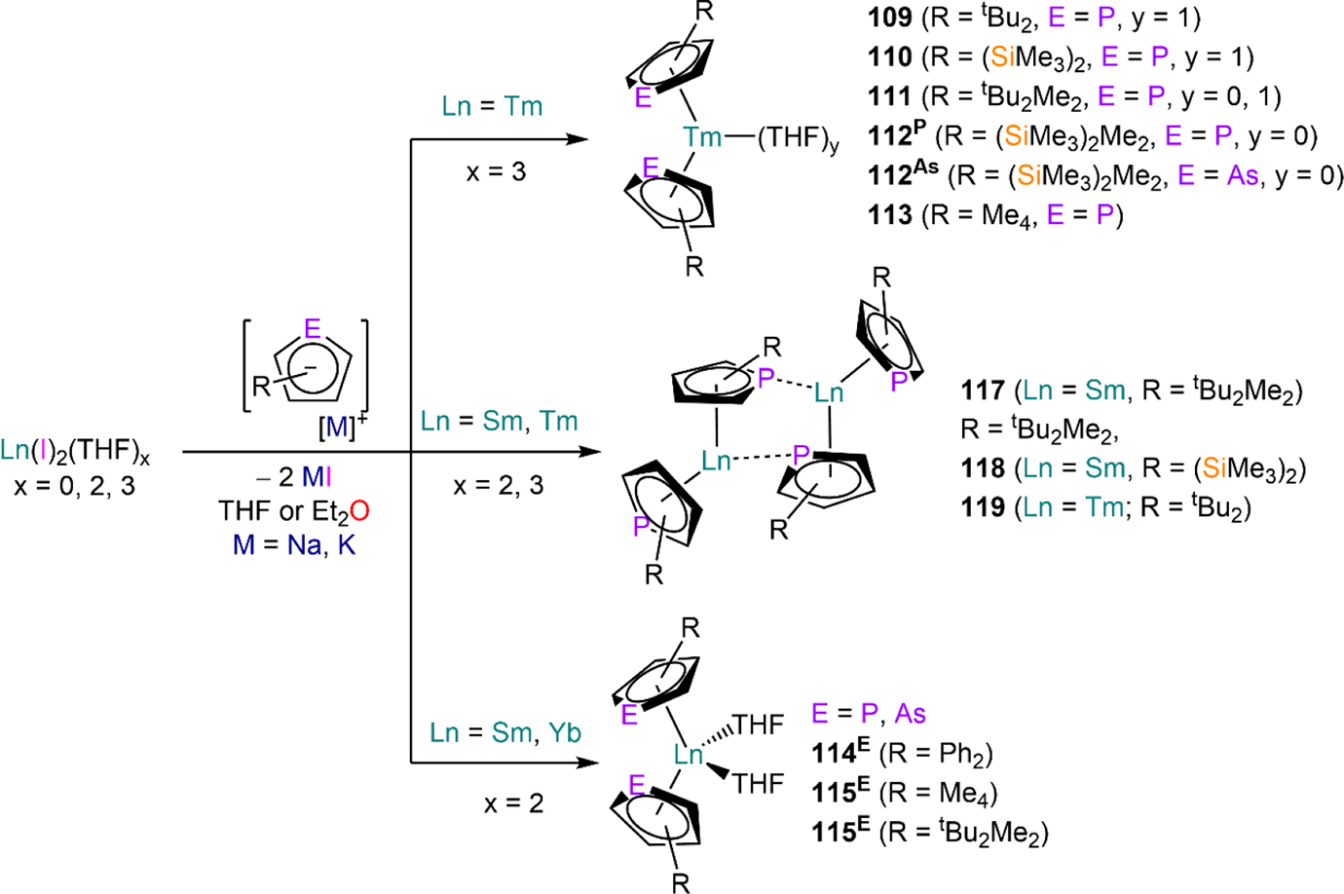
Synthesis of Sm, Tm, and Yb Bis-phospholyl Complexes^284−286^

The same methodologies have also been extended to the synthesis of phospholyl and arsolyl derivatives. Nief and co-workers were able to obtain several Tm(II) metallocene-type complexes, with varying degrees of substitution on the phosphorus or arsenic heterocycles (Scheme 38), *i.e.*, [Tm{η^5^-C_4_H_2_P^t^Bu_2_-2,5}_2_(THF)] (**109**),^284^ [Tm{η^5^-C_4_H_2_P(SiMe_3_)_2_-2,5}_2_(THF)] (**110**),^284^ [Tm{η^5^-C_4_P^t^Bu_2_-2,5-Me_2_-3,4}_2_(THF)_*x*_] (**111,***x* = 0, 1),^285,286^ [Tm{η^5^-C_4_E(SiMe_3_)_2_-2,5-Me_2_-3,4}_2_] (**112**^**E**^, E = P, As),^285^ and [Tm{η^5^-C_4_PMe_4_}_2_] (**113**). SmI_2_ and YbI_2_ have also been used for the preparation of bis-phospholyl (**114**^**P**^**-116**^**P**^) and arsolyl complexes (**114**^**As**^**-116**^**As**^);^283,286^ interestingly, in some cases with Sm and Tm the resulting phospholyl complexes are obtained as dimeric structures in which the lone pair of the phosphorus atom within the phospholyl ring bridges between two metallocene moieties, *i.e.,* [Ln{η^5^-C_4_P(R)}{μ:η^5^-C_4_H_2_P(R)}]_2_ (**117**, Ln = Sm, R = ^t^Bu_2_Me_2_; **118**, Ln = Sm, R = (SiMe_3_)_2_Me_2_; **119**, Ln = Tm, R = ^t^Bu_2_).^283,286^

**Scheme 39:**
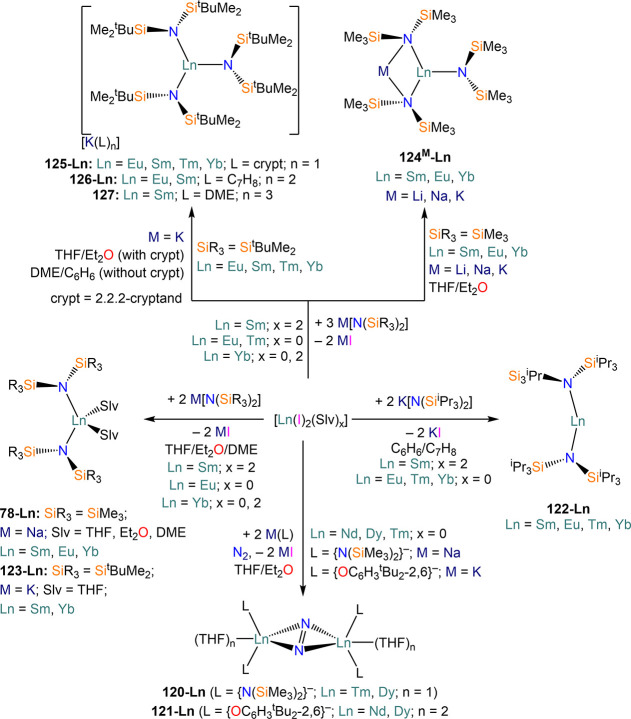
Reactivity of LnI_2_ Salts with Monodentate Silylamides and Aryloxides, Leading to the Isolation of Bis-amides, Tris-amides, and Dinitrogen Activation Products

The discussion on cyclopentadienyl, phospholyl, and arsolyl derivatives of Ln(II) metals provides a good snapshot of the possibilities offered by the species; classic divalent Lns (with the addition of Tm) are able to preserve the divalent state in the final products, while NdI_2_ and DyI_2_ are too reducing. As mentioned above, LnI_2_ salts can also be used in salt elimination reactions with a variety of ligand transfer reagents. Nitrogen donors are among the most popular ligand choices in RE chemistry, particularly monodentate silylamide {N(SiMe_3_)_2_}^−^ (Scheme 39). The homoleptic complexes [Yb{N(SiMe_3_)_2_}_2_(Et_2_O)_2_] (**78-Yb**·2OEt_2_) and [Eu{N(SiMe_3_)_2_}_2_(DME)_2_] (**78-Eu**·2DME) were first obtained by Andersen and co-workers from the reaction between solvent-free LnI_2_ and two equivalents of Na[N(SiMe_3_)_2_] in ethereal solvents (Et_2_O, THF or DME).^272,287^ Similarly, Evans *et al.* reported the synthesis of [Sm{N(SiMe_3_)_2_}_2_(THF)_2_] (**78-Sm**·2THF) from [Sm(I)_2_(THF)_2_] and Na[N(SiMe_3_)_2_] in THF.^288^ These salt elimination protocols cannot be applied to Tm(II), Dy(II), and Nd(II) because of their high reducing nature. In the case of Dy and Tm, dinitrogen activation products [{Ln{N(SiMe_3_)_2_}_2_(THF)}_2_(μ-N_2_)] (**120-Ln**; Ln = Dy, Tm) are obtained;^289^ when TmI_2_ is employed, Evans and co-workers isolated a purple solid which they assigned as putative Tm{N(SiMe_3_)_2_}_2_(THF)_*x*_ (**78-Tm**); however, no structural information has yet been reported.^289^ DyI_2_ is much more reducing than TmI_2_, and as a result, the solutions obtained from the reaction with Na[N(SiMe_3_)_2_] are highly temperature-unstable, affording dinitrogen activation product **120-Dy** even at −78 °C. It is noteworthy that the analogous Nd complex [{Nd{N(SiMe_3_)_2_}_2_(THF)}_2_(μ-N_2_)] (**120-Nd**) has been obtained only from the reduction of Ln/group 1 “ate” complexes, rather than through a transient Nd(II) complex, “Nd{N(SiMe_3_)_2_}_2_”.^290^ Additionally, Evans and co-workers applied a similar synthetic strategy to the synthesis of aryloxide complexes [{Ln(OC_6_H_2_^t^Bu_2_-2,6)_2_(THF)_2_}_2_(μ-N_2_)] (**121-Ln**; Ln = Nd, Dy), which were isolated from the reaction between LnI_2_ and K(OC_6_H_2_^t^Bu_2_-2,6).^289,291^ Mills and co-workers were able to isolate the first Tm(II) bis-amide complex, [Tm{N(Si^i^Pr_3_)_2_}_2_] (**122-Tm**), which was obtained from the reaction between TmI_2_ and K[N(Si^i^Pr_3_)_2_] in benzene;^292^ similar protocols were also used by the same authors for the synthesis of the analogous [Sm{N(Si^i^Pr_3_)_2_}_2_] (**122-Sm**),^293^ [Eu{N(Si^i^Pr_3_)_2_}_2_] (**122-Eu**), and [Yb{N(Si^i^Pr_3_)_2_}_2_] (**122-Yb**).^292^ When the less sterically demanding silylamide ligand {N(Si^t^BuMe_2_}^−^ is employed, the solvated species [Ln{N(Si^t^BuMe_2_)_2_}_2_(THF)_2_] (**123-Ln**; Ln = Sm, Yb) are obtained instead (Scheme 39). LnI_2_ salts can also be used for the preparation of heterobimetallic “ate” complexes of general formula LnM{N(SiMe_3_)_2_}_3_ (**124**^**M**^-**Ln**; M = alkali metal; Ln = Eu, Sm, Yb); the methodology normally consists of the reaction of three equivalents of ligand transfer reagent with LnI_2_ (Scheme 39) and has also been used to synthesize separated ion pair complexes [K(L)_*n*_][Ln{N(Si^t^BuMe_2_)_2_}_3_] (**125-Ln**, Ln = Sm, Eu, Tm, Yb, L = 2.2.2-cryptand, *n* = 1; **126-Ln**, Ln = Sm, Eu, L = C_7_H_8_, *n* = 2; **127** Ln = Sm, L = DME, *n* = 3).^294^

A multitude of other ligands have also been employed in salt elimination reactions with LnI_2_ salts, which include aromatic ligands (Figure 6, **A**, **B**),^295,296^ mono- and multidentate dentate alkyls (**C**–**H**),^114,191,297−304^ multidentate *N-*donors (**I**–**M**),^299,305−310^ phosphides (**N**),^311^ silanides (**O**),^312,313^ and gallyls.^132,314^ A major challenge in Ln(II) chemistry is the stabilization of heteroleptic complexes of the type “Ln(L)(I)” directly from salt elimination reactions, owing to the large steric demands of divalent Lns and the tendency in some cases to rearrange to homoleptic Ln(L)_2_ and LnI_2_. This interest originates from the possibility of further functionalizing the complexes by substituting the iodide ligand *via* metathetical reactivity. Multidentate donors such as amidinates (**I**), guanidinates (**J**), β-diketiminates (BDI, **K**, **M**), tris-pyrazolylborates (Tp, **L**), and bis-iminophosphorano-methanide (**E**) have been successfully employed to deliver such species.

**Scheme 40:**
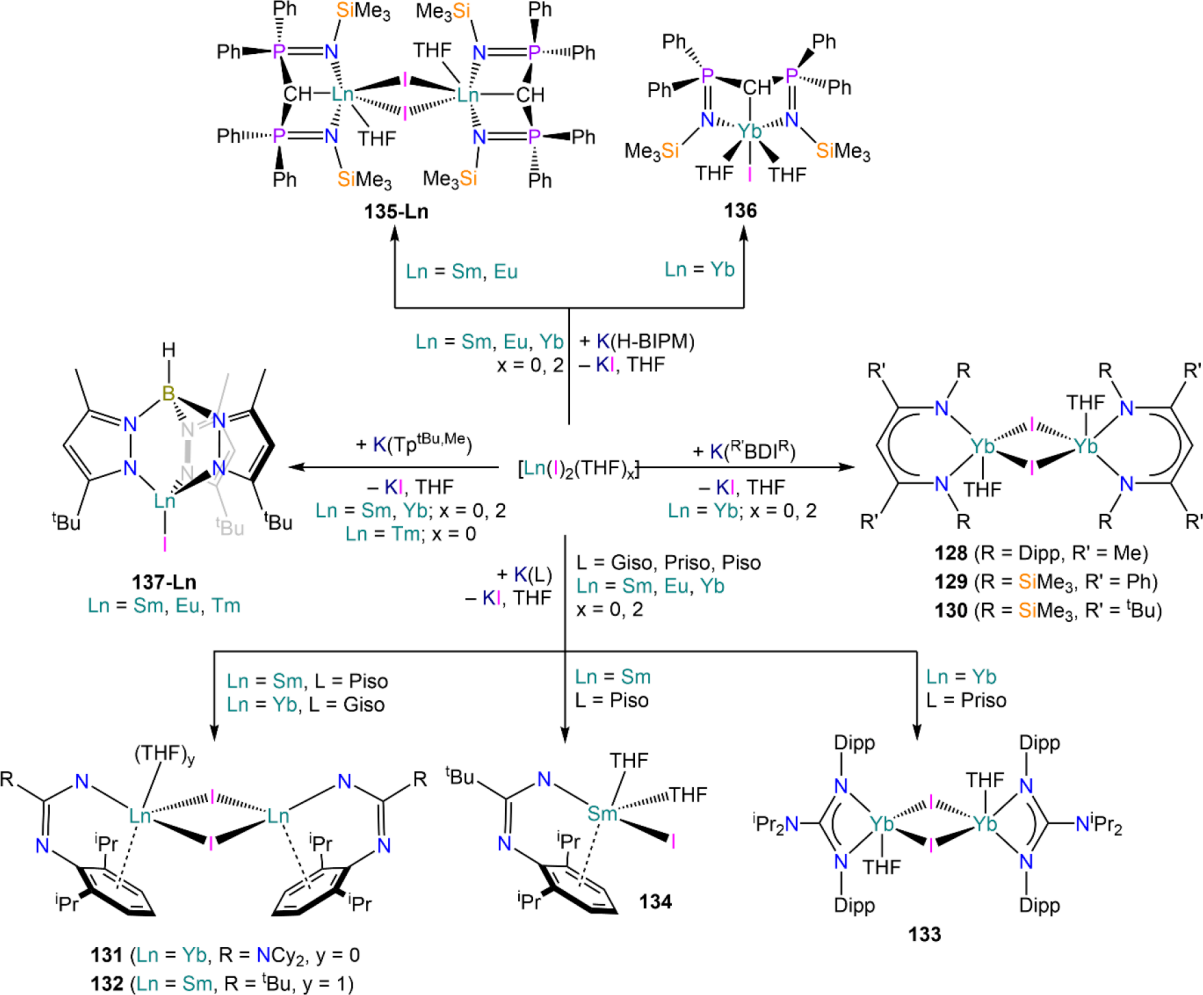
Selected Examples of Heteroleptic Ln(II) Complexes Obtained *via* Salt Elimination Reactions from LnI_2_ Salts

Lappert and co-workers reported the preparation of the heteroleptic complexes [{Yb(^R′^BDI^R^)(η-I)(THF)}_2_] (**128**, BDI = β-diketiminate; R = Dipp, R′ = Me; **129**, R **=** SiMe_3_; R′ = Ph) from K(^R′^BDI^R^) and YbI_2_ in THF (Scheme 40);^315^ Jones *et al.* also reported the analogous complex [{Yb(^tBu^BDI^Dipp^)(η-I)(THF)}_2_] (**130**) obtained with similar methodologies.^316^ Chen and co-workers reacted potassium salts of tethered BDI ligands, {(R)C(NDipp)CHC(R)NCH_2_CH_2_N(Me)CH_2_CH_2_NMe_2_}^−^ (^R^BDI^N2^, R = Me, ^t^Bu), with [Yb(I)_2_(THF)_2_] and [Sm(I)_2_(THF)_2_] to give dimeric heteroleptic complexes [{Ln(^R^BDI^N2^)(η-I)(THF)}_2_].^317−320^ Amidinate and guanidinate potassium salts afford similar results; interestingly, the flanking aryl groups can also interact with the metal center, thus supporting a switch in the coordination mode from bidentate to monodentate, such as in the dimeric complexes [{Yb(Giso)(η-I)}_2_] (**131**, Giso = {(DippN)_2_CN(C_6_H_11_)_2_}^−^),^321^ [{Sm(Piso)(η-I)}_2_] (**132**, Piso = {(DippN)_2_CN^t^Bu}^−^),^322^ [{Yb(Priso)(η-I)(THF)}_2_] (**133**, Priso = {(DippN)_2_CN^i^Pr_2_}^−^),^321^ and monomeric Sm complex [Sm(Piso)(I)(THF)_2_] (**134**, Scheme 40).^323^ Furthermore, Roesky and co-workers reacted the potassium salt K(H-BIPM) (H-BIPM = {CH(PPh_2_NSiMe_3_)_2_}^−^) with [Ln(I)_2_(THF)_2_] (Ln = Sm, Eu) in THF (Scheme 40) to give dimeric [{Ln(H-BIPM)(μ-I)(THF)}_2_] (**135-Ln**, Ln = Sm, Eu) and monomeric [Yb(H-BIPM)(I)(THF)_2_] (**136**).^324,325^ Finally, Takats and co-workers were able to isolate the heteroleptic complexes [Ln(Tp^tBu,Me^)(I)] (**137-Ln**, Ln = Sm, Yb) from facile reaction between K(Tp^tBu,Me^) and LnI_2_ in THF at room temperature;^309^ remarkably, the authors were also able to extend this chemistry to Tm(II) and isolated complex [Tm(Tp^tBu,Me^)(I)] (**137-Tm**), which is a very rare example of a heteroleptic Tm(II) species (Scheme 40).^326^

### 4.2 Trivalent RE Halides

Trivalent halides (Table 4) are the most common starting materials used in RE synthetic chemistry, particularly for salt elimination reactions; the only exceptions are trifluorides, which are very rarely employed in organometallic and coordination chemistry methodologies.^327,328^ These materials are usually commercially available, but the cost of anhydrous salts can be significant; additionally, a high degree of purity is required for these starting materials, which cannot always be guaranteed from commercial sources. There are also some special circumstances in which a knowledge of these basic methodologies can be extremely useful, as shown by Chilton and co-workers in the preparation of single molecule magnets with isotopically pure ^164^Dy;^329,330^ the only commercially available starting material containing pure ^164^Dy is ^164^Dy_2_O_3_, which has to be converted into ^164^DyCl_3_ for the preparation of organometallic complexes.^329,330^ Additionally, owing to the similarities between early Lns and trivalent actinides (Ans), knowledge of these preparative methods can be used to develop synthetic protocols with RE surrogates that can be transferred to the An family, which is particularly important when working with scarce and highly hazardous transuranic elements.^331−333^

**Table 4:** Anhydrous and THF Adducts of RE Trihalides^a^

	Cl	Br	I
Sc	ScCl_3_^339,356^	ScBr_3_^339,356^	ScI_3_^231,232^
	[Sc(Cl)_3_(THF)_3_]^357^	[Sc(Br)_3_(THF)_3_]^358^	*ScI*_*3*_*(THF)*_*3*_(359)
	*ScCl*_*3*_*(THF)*_*1.5*_(339)	*ScBr*_*2*_*(THF)*_*2.5*_(339)	
Y	YCl_3_^340^	YBr_3_^349^	YI_3_^233,363,364^
	[Y(Cl)(μ-Cl)_2_(THF)_2_]_*n*_^360^	*YBr*_*3*_*(THF)*_*2*_(362)	[Y(I)_3_(THF)_3.5_]^335^
	[Y(Cl)_3_(THF)_3.5_]^360^	*YBr*_*3*_*(THF)*_*3*_(334)	
	*YCl*_*3*_*(THF)*_*3*_(339,361)		
La	LaCl_3_^14,348^	LaBr_3_^14,348^	LaI_3_^14,233,348^
	[La(Cl)(μ-Cl)_2_(THF)_2_]_*n*_^365^	[La(Br)_3_(THF)_4_]^343^	[La(I)_3_(THF)_3.5_]^342^
	*LaCl*_*3*_*(THF)*_*3*_(361)	*LaBr*_*3*_*(THF)*_*2*_(367)	[La(I)_3_(THF)_4_]^335,369^
	*LaCl*_*3*_*(THF)*_*4*_(365,366)	*LaBr*_*3*_*(THF)*_*3*_(368)	
Ce	CeCl_3_^14,348^	CeBr_3_^351^	CeI_3_^14,233,348^
	[Ce(Cl)(μ-Cl)_2_(THF)_2_]_*n*_^370^	[Ce(Br)_3_(THF)_4_]^373^	[Ce(I)_3_(THF)_4_]^341,374^
	*CeCl*_*3*_*(THF)*_*3*_(371,372)		
Pr	PrCl_3_^14,348^	PrBr_3_^351^	PrI_3_^14,233,348^
	[Pr(Cl)(μ-Cl)_2_(THF)_2_]_*n*_^365,375^	[Pr(Br)_3_(THF)_4_]^344,351^	[Pr(I)_3_(THF)_4_]^335,341^
	*PrCl*_*3*_*(THF)*_*3*_(376)		
Nd	NdCl_3_^14,339,348^	NdBr_3_^339^	NdI_3_^14,233,348^
	[Nd(Cl)(μ-Cl)_2_(THF)_2_]_*n*_^370^	[Nd(Br)_3_(THF)_4_]^358^	[Nd(I)_3_(THF)_3.5_]^335,341^
	[Nd(Cl)_3_(THF)_4_]^377^		[Nd(I)_3_(THF)_4_]^378^
	*NdCl*_*3*_*(THF)*_*3*_(376)		*NdI*_*3*_*(THF)*_*3*_(379)
	*NdCl*_*3*_*(THF)*_*2.5*_(339)		
Sm	SmCl_3_^14,348^	SmBr_3_^14,348^	SmI_3_^14,233,348^
	[Sm(Cl)_3_(THF)_4_]^380^	[Sm(Br)_3_(THF)_4_]^344,351^	[Sm(I)_3_(THF)_3_]^382,383^
	*SmCl*_*3*_*(THF)*_*2*_(381)		[Sm(I)_3_(THF)_3.5_]^341,384^
	*SmCl*_*3*_*(THF)*_*3*_(376)		*SmI*_*2*_*(THF)*_*2*_(385)
Eu	EuCl_3_^14,348^	EuBr_3_^14,348^	EuI_3_^387^
	[Eu(Cl)_3_(THF)_4_]^386^	[Eu(Br)_3_(THF)_3.5_]^344^	*EuI*_*3*_*(THF)*_*3.5*_(341)
Gd	GdCl_3_^339^	GdBr_3_^339^	GdI_3_^14,233,348^
	[Gd(Cl)_3_(THF)_3.5_]^365^	*GdBr*_*3*_*(THF)*_*3.5*_(339)	[Gd(I)_3_(THF)_3.5_]^335^
	[Gd(Cl)_3_(THF)_4_]^375^		
	*GdCl*_*3*_*(THF)*_*2*_(388)		
	*GdCl*_*3*_*(THF)*_*3*_(171)		
Tb	TbCl_3_^340^	TbBr_3_^351^	TbI_3_^14,233,348^
	[Tb(Cl)_3_(THF)_3.5_]^370^		[Tb(I)_3_(THF)_3.5_]^341^
	*TbCl*_*3*_*(THF)*_*3*_(376,389)		
Dy	DyCl_3_^340^	DyBr_3_^349^	DyI_3_^14,233,348^
	[Dy(Cl)_3_(THF)_3.5_]^390^		*DyI*_*3*_*(THF)*_*3.5*_(335,341)
	*DyCl*_*3*_*(THF)*_*3*_(391,392)		
	[Dy(Cl)_2_(THF)_5_][BPh_4_]^393^		
Ho	HoCl_3_^339^	HoBr_3_^349^	HoI_3_^14,233,348^
	[Ho(Cl)_3_(THF)_3.5_]^394^		*HoI*_*3*_*(THF)*_*3.5*_(341)
	*HoCl*_*3*_*(THF)*_*3*_(392)		
	*HoCl*_*3*_*(THF)*_*2.5*_(365)		
Er	ErCl_3_^340^	ErBr_3_^349^	ErI_3_^14,233,348^
	[Er(Cl)_3_(THF)_3.5_]^365^		[Er(I)_3_(THF)_3_]^396^
	*ErCl*_*3*_*(THF)*_*2*_(395)		*ErI*_*3*_*(THF)*_*3.5*_(335,341)
	*ErCl*_*3*_*(THF)*_*3*_(376)		
Tm	TmCl_3_^351^	TmBr_3_^349^	TmI_3_^14,233,348^
	*TmCl*_*3*_*(THF)*_*3*_(391)		*TmI*_*3*_*(THF)*_*3.5*_(335,341)
	*TmCl*_*3*_*(THF)*_*2.7*_(365)		
Yb	YbCl_3_^14,348^	YbBr_3_^14,348^	YbI_3_^14,348^
	[Yb(Cl)_3_(THF)_3_]^171^		[Yb(I)_3_(THF)_3_]^398^
	[Yb(Cl)_3_(THF)_3.5_]^397^		[Yb(I)_3_(THF)_3.5_]^399^
	*YbCl*_*3*_*(THF)*_*2*_(395)		
Lu	LuCl_3_^339^	LuBr_3_^349^	LuI_3_^14,233,348^
	[Lu(Cl)_3_(THF)_3_]^339,365,400^		*LuI*_*3*_*(THF)*_*4*_(401)
	*LuCl*_*3*_*(THF)*_*2*_(367)		

a Solvated salts in italics have not been structurally authenticated.

**Scheme 41:**
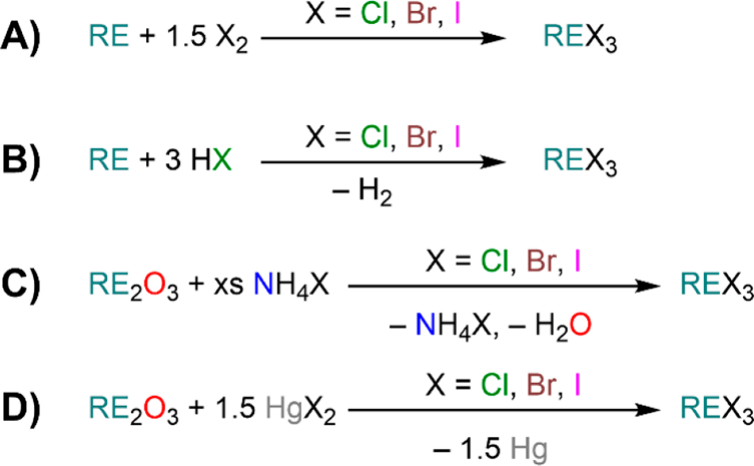
Main Synthetic Strategies for the Preparation of Trivalent RE Halides

Trivalent salts can be obtained from the direct reaction of the metal with either halide or hydrogen halides (**A** and **B**, Scheme 41), though this type of approach can be challenging for at least one of the following factors: (1) reactions need to be carried out at high temperatures above the melting point of the REX_3_ salt (usually above 700 °C); (2) the halide source needs to be of very high purity (particularly if using HCl); (3) anhydrous halides can react with the reaction vessels at high temperatures.^235^ This method can be problematic particularly for RECl_3_, while the preparation of tribromides^334^ and triiodides^335^*via* direct reaction of the metal with bromine or iodine under inert atmosphere is more straightforward, as both materials can be obtained with very high purity.^9,233^ A more convenient method consists of the reaction of the RE oxides, RE_2_O_3_, with NH_4_X (X = Cl, Br, I) at high temperatures. Reed *et al.* originally developed this methodology as a solid-state synthesis (“dry method”) for RECl_3_ and REBr_3_,^336,337^ which was later applied for the preparation of REI_3_ by Young and Hastings (**B**, Scheme 41).^338^ However, this approach can also be implemented in synthetic laboratories as a “wet method” by dissolving the RE oxides in acid (HCl and HBr) and in the presence of NH_4_X (X = Cl, Br). This methodology has been refined over the years and has now become a routine approach for the preparation of anhydrous RE chlorides and bromides in many synthetic laboratories.^339,340^ The synthesis of RE tribromides^334^ and triiodides^335^ is also easily achieved *via* direct reaction of the metal with bromine or iodine under inert atmosphere in ethereal solvents to yield solvated adducts.^334,335^ This is a particularly efficient method for the synthesis of triiodides, which can be prepared in very large scales as ethereal adducts [RE(I)_3_(Slv)_*x*_] (Slv = Et_2_O, THF; *x* = 3, 3.5, 4);^335,341^ Evans and co-workers have also reported the preparation of pyridine adducts,^342^ while Deacon and Petricěk have obtained adducts of various halides with DME and glyme.^343,344^ An alternative approach is based on the use of HgX_2_ salts with metallic REs (**D**, Scheme 40), analogous to the method described previously for divalent Lns (*vide supra*, section 4.1).^233,345,346^

**Figure 7:**
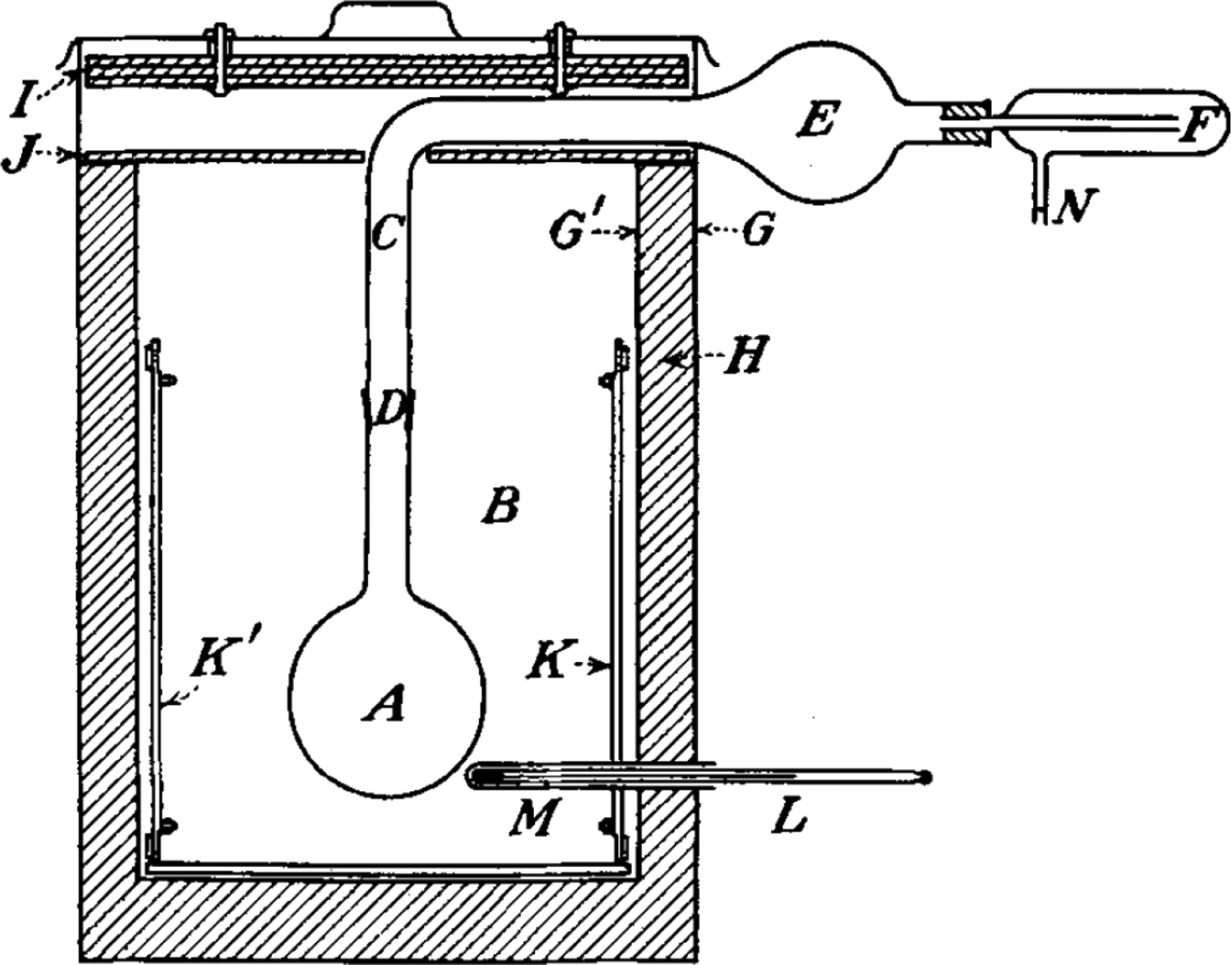
Apparatus for the “dry method” synthesis of anhydrous trivalent RE chlorides.^347^**A**, pyrex flask; **B**, furnace; **C**, delivery tube (internal diameter 28 mm); **D**, ground glass connection; **E**, ammonium chloride receiving bulb (500 mL); **F**, trap (connected *via* rubber stopper); **G–G′**, galvanized-iron cans; **H**, asbestos; **I**, asbestos boards; **J**, asbestos inner cover; **K**, heating unit (six Westinghouse No. 299-425 space heaters, 110 V and 220 W capacity); **L**, thermometer; **M**, pyrex thermometer cover; **N**, high-vacuum pump connection. Reproduced with permission from ref (347). Copyright 1939 Wiley.

In Reed’s original methodology, RE_2_O_3_ and NH_4_Cl are mixed by heating with a Bunsen burner, forming RE chlorides as ammonium salts adducts, (NH_4_)_2_RECl_5_ or (NH_4_)_3_RECl_6_,^340^ dispersed in an NH_4_Cl matrix;^336^ the quantity of ammonium salt is crucial for the success of this methodology, as incorrect stoichiometries could lead to the formation of decomposition products such as oxychlorides “REOCl”. The resulting mixture is then transferred into the bulb (**A**) of the apparatus shown in Figure 7.^347^**A** is connected to a bent delivery tube (Figure 7, **C**) equipped with a collection bulb at the end (Figure 7, **E**); the apparatus is connected to a high-vacuum line *via* a trap (Figure 7, **F**) positioned at the end of the apparatus. Once the reactor has been charged with the RECl_3_/NH_4_Cl mixture, the furnace is heated to 300 °C for up to 30 h, affording anhydrous RECl_3_ upon sublimation of NH_4_Cl. It is important to avoid higher temperatures at least at the initial stages of the final purification step, as unreacted RE_2_O_3_ can react with (NH_4_)_3_RECl_6_ at 320 °C to form YOCl, NH_3_, and H_2_O.^337^ A similar apparatus was developed by Taylor and Carter, and later refined by Kutscher and Schneider, for drying hydrated salts REX_3_(H_2_O)_*x*_ (X = Cl, Br, I) with ammonium halides NH_4_X (X = Cl, Br, I).^14,348^ Other iterations have also been reported by Corbett, Meyer, and Edelmann, and the method has also been applied to the synthesis of REBr_3_.^240,349^

**Scheme 42:**
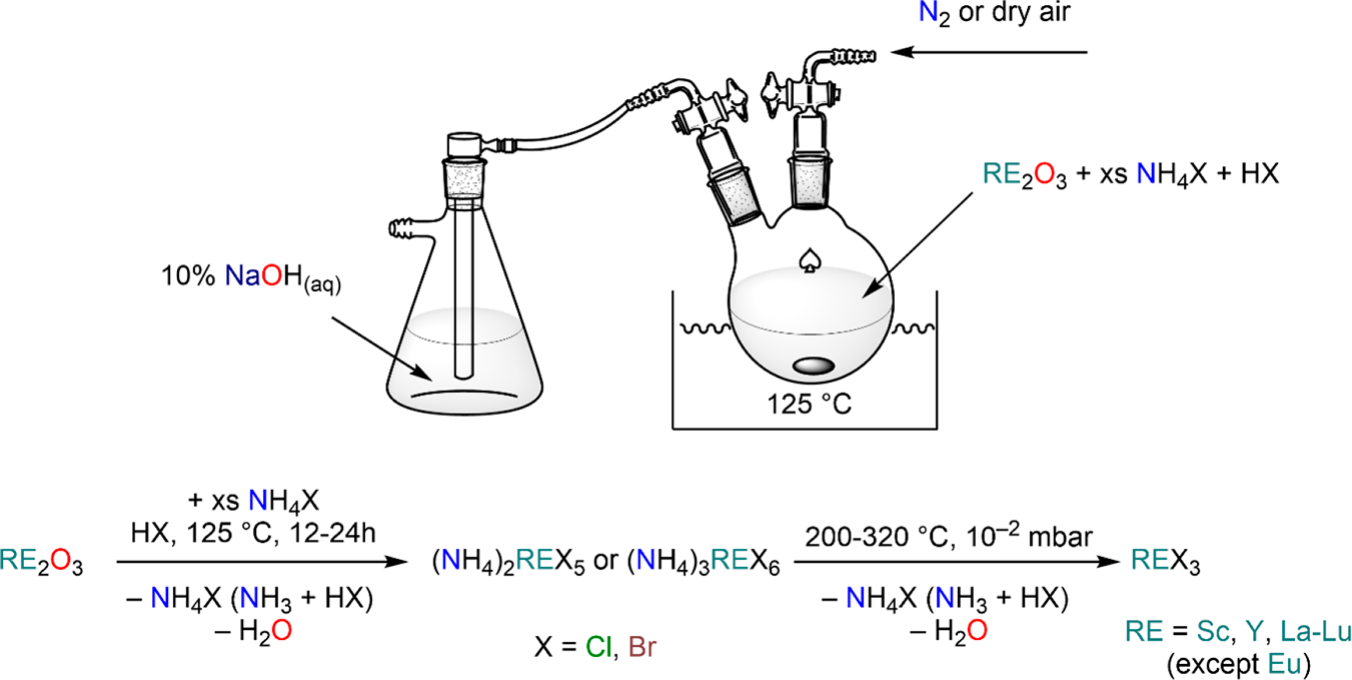
Synthesis of Anhydrous RECl_3_ and Apparatus for the “Wet Method” Synthesis^339,351^

A more convenient approach toward the preparation of REX_3_ salts (X = Cl, Br) is the so-called “wet method”. Different from the previously described “dry method”, RE_2_O_3_ (oxides containing tetravalent metals can also be used, *e.g.*, CeO_2_, Pr_2_O_7_, and Tb_4_O_7_) is first dissolved in a concentrated HCl or HBr solution containing excess NH_4_X (Scheme 42).^339,340,350,351^ The solution is boiled to dryness (125 °C) under a stream of dry air or nitrogen, and the fumes are passed through a trap containing a 10% NaOH solution. The resulting white material, (NH_4_)_2_REX_5_(H_2_O)_*x*_, is finely ground and transferred into a sublimation apparatus analogous to those illustrated by Meyer and Corbett.^240,349^ The final stage of this protocol is analogous to the one previously described in the “dry method”, which entails drying the crude material under reduced pressure. Different temperature programs are reported in the literature for this procedure; according to Marks and Diaconescu, the raw material should be heated at 200 °C maximum to remove excess water first, followed by removal of excess NH_4_X at higher temperatures (*T* > 300 °C).^339,351^

Anhydrous RECl_3_ can also be obtained by dehydration of RECl_3_(H_2_O)_*x*_. One method is briefly described above, which uses NH_4_X (X = Cl, Br, I) as a drying agent and can be applied to other halides.^352^ Another typical methodology employed for drying RECl_3_(H_2_O)_*x*_ involves the use of SOCl_2_ and was originally reported by Freeman and Smith.^352^ In their methodology, RECl_3_(H_2_O)_*x*_ is converted into a fine powder, transferred into a flask with excess SOCl_2_, and then refluxed gently until dehydration is complete; particular care should be taken to avoid overheating, as decomposition of SOCl_2_ could lead to the formation of undesired oxychlorides. The time required to achieve complete dehydration increases going across the Ln family, with 1 h required for LaCl_3_ and 110 h required for ErCl_3_; this is likely due to the increased Lewis acidity of the metals as a consequence of the higher charge density of the smaller Lns.^9,352^ It should be noted that anhydrous RE halides are not particularly soluble, and in many instances these have to be converted into solvated forms. Such procedures can be very time-consuming; therefore, simple and quick methodologies that deliver these species as ethereal adducts can be particularly desirable (*vide infra*).

**Scheme 43:**
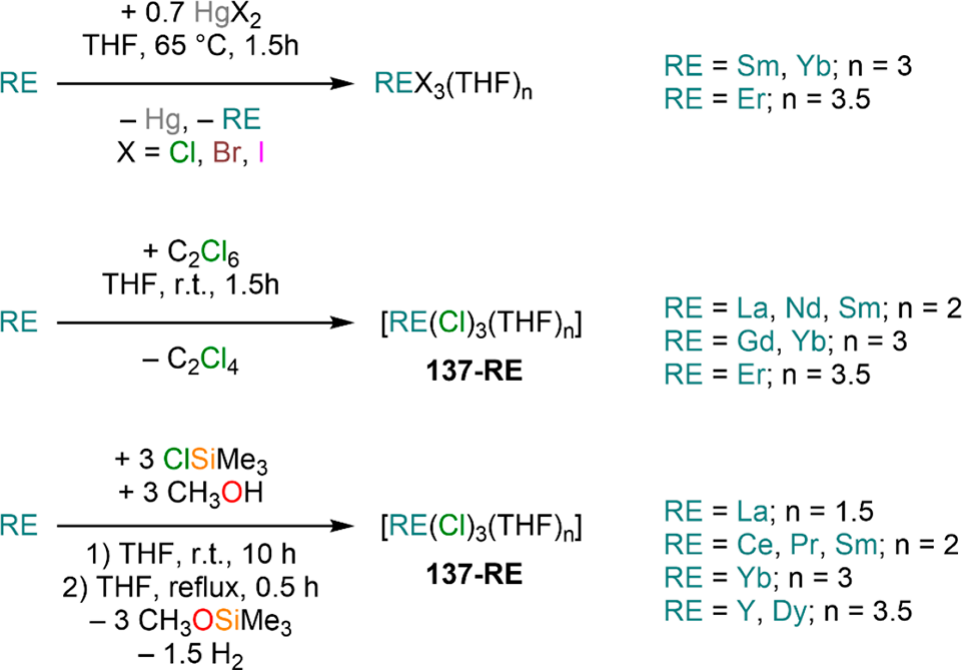
Synthesis of Solvated Trivalent RE Halides *via* RT Reactions with HgX_2_^126,345^ or Direct Reaction with Hexachloroethane^171^ or Trimethylsilylchloride^354^

High-temperature methods are used for the preparation of unsolvated REX_3_ with Hg(II) halides, but these methodologies have several drawbacks (*e.g.*, bespoke solid-state reactors, removal of Hg by distillation, and purification of REX_3_ by sublimation) which make it impractical for most synthetic laboratories. However, Deacon and co-workers showed that standard solution synthesis can also be used for laboratory-scale preparation of solvated RECl_3_; this methodology can also be adapted to other halides and is carried out using the same apparatus employed for other RT and RTP reactions (*vide supra*, Figure 2).^126,345^ In Deacon’s method, a solution of REX_3_ in THF is produced which allows removal of Hg residues *via* filtration (Scheme 43). The compounds obtained through this method are solvated species, which were originally reported by the authors as YbCl_3_(THF)_3_, YbBr_3_(THF)_3_, YbI_3_(THF)_3_, SmI_3_(THF)_3_ and ErCl_3_(THF)_3.5_.^345^ An alternative method was also reported by Deacon *et al.* in which RE powders are treated with hexachloroethane in THF, to give THF adducts [RE(Cl)_3_(THF)_*n*_] (**137-RE**; RE = La, Nd, Sm, *n* = 2; RE = Gd, Yb, *n* = 3; RE = Er, *n* = 3.5) (Scheme 43);^171^ additionally, the authors reported the structure of dimeric [{Yb(Cl)_2_(μ-Cl)(THF)_2_}_2_] (**138**), which was obtained upon treatment of [Yb(Cl)_3_(THF)_3_] (**137-Yb**·3THF) with pentane over several months. It is noteworthy that there are several different reports in the literature regarding the number of THF molecules present in solvated RECl_3_ salts. This is due to the variety of methods and conditions employed for their preparation, which lead to the isolation of these adducts as either neutral molecular complexes—monomeric [RE(Cl)_3_(THF)_3_] (**137-RE**·3THF) or polymeric [RE(Cl)_3_(THF)_2_]_*n*_ (**137-RE**·2THF)—or separated ion pair species [RE(Cl)_2_(THF)_4_][RE(Cl)_4_(THF)_2_], which are usually simplified as [RE(Cl)_3_(THF)_3.5_] (**137-RE**·3.5THF). The adducts reported by Deacon and co-workers were obtained by Soxhlet extraction,^171^ while other THF adducts are obtained by heating RECl_3_ in THF followed by simple removal of solvent, and their formula is usually reported as RECl_3_(THF)_2_.^339^ Finally, Wu *et al.* reported the direct reaction of RE powders with trimethylsilyl chloride and methanol at room temperature (Scheme 43).^354^ These reactions produce the desired RE chlorides in quantitative yields for all the metals tried in this protocol (Y, La, Ce, Pr, Sm, Dy, and Yb); additionally, different silanes (dimethylchlorosilane and silicon tetrachloride) and alcohols can be employed (ethanol, propanol, and 1-pentanol).

**Scheme 44:**
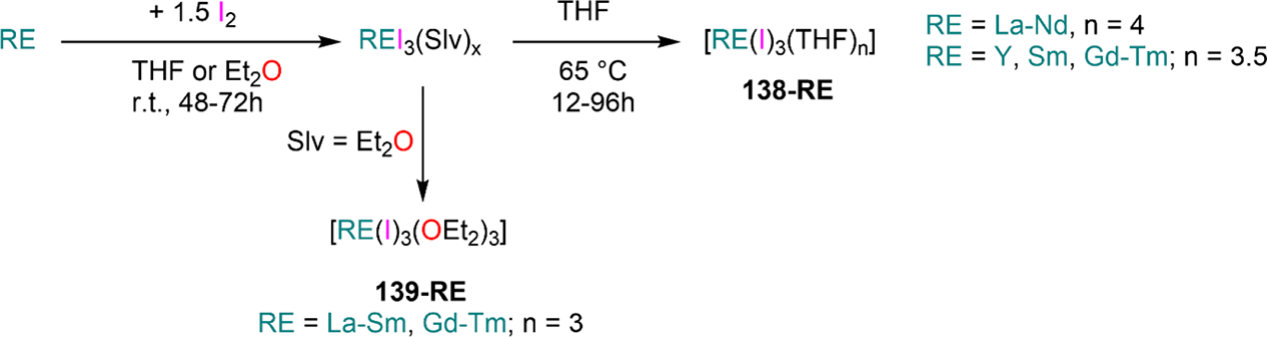
Synthesis of THF (**138-RE**) and Et_2_O Adducts (**139-RE**) or REI_3_^335,341^

Solvated RE triiodides can be obtained *via* various solution methods, including (1) RT reaction of RE metal with HgI_2_ (*vide supra*, Scheme 43);^345^ (2) reaction of RE metal with iodoethane or diiodoethane in THF;^343,355^ (3) reaction of RE metal with iodine in isopropanol;^335^ and (4) reaction of RE metal with iodine in THF.^335^ The last method was developed by Izod *et al.* and provides a very clean and consistent route toward obtaining triiodides of all REs (Scheme 44).^335^ In this method, iodine is added slowly to RE metal chips in THF, and the crude material is then baked to remove excess iodine, followed by Soxhlet extraction to afford solvated [RE(I)_3_(THF)_x_] (**138-RE**; *x* = 3.5 or 4 for La–Nd; Table 1).^335^ La Pierre and co-workers reported an analogous methodology in which the initial step is performed in diethyl ether; resulting diethyl ether adducts [RE(I)_3_(Et_2_O)_3_] (**139-RE**; RE = La, Ce, Nd, Pr, Sm, Gd, Tb, Dy, Ho, Er, Tm) were also isolated, and several of them were structurally authenticated by the authors (Scheme 44).^341^ It is noteworthy that these etherates easily lose solvent under reduced pressure, so the final solvent content and composition can vary.^341^

**Scheme 45:**
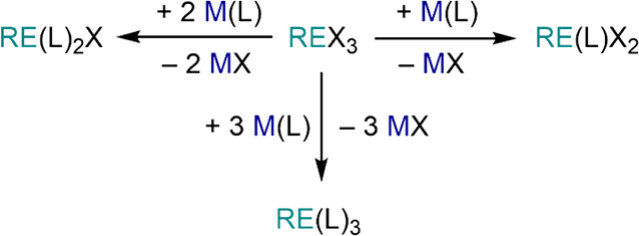
Schematic Representation of Salt Elimination Reactions with REX_3_ Salts

In principle, salt elimination reactions with REX_3_ salts can afford both homoleptic, “RE(L)_3_”, and heteroleptic complexes, “RE(L)_2_X” and “RE(L)X_2_” (Scheme 45). The outcome of these reactions is largely dictated by the electronic and steric features of the ligands employed, and the alkali metal and halide source chosen for the reaction (*vide supra*, section 2.1). The ligands used in salt elimination reactions with LnX_2_ (*vide supra*, Figure 6) have also been used in analogous chemistry with trivalent REs, for which the ligand scope is far greater owing to the greater stability of most RE^3+^ ions.

**Scheme 46:**
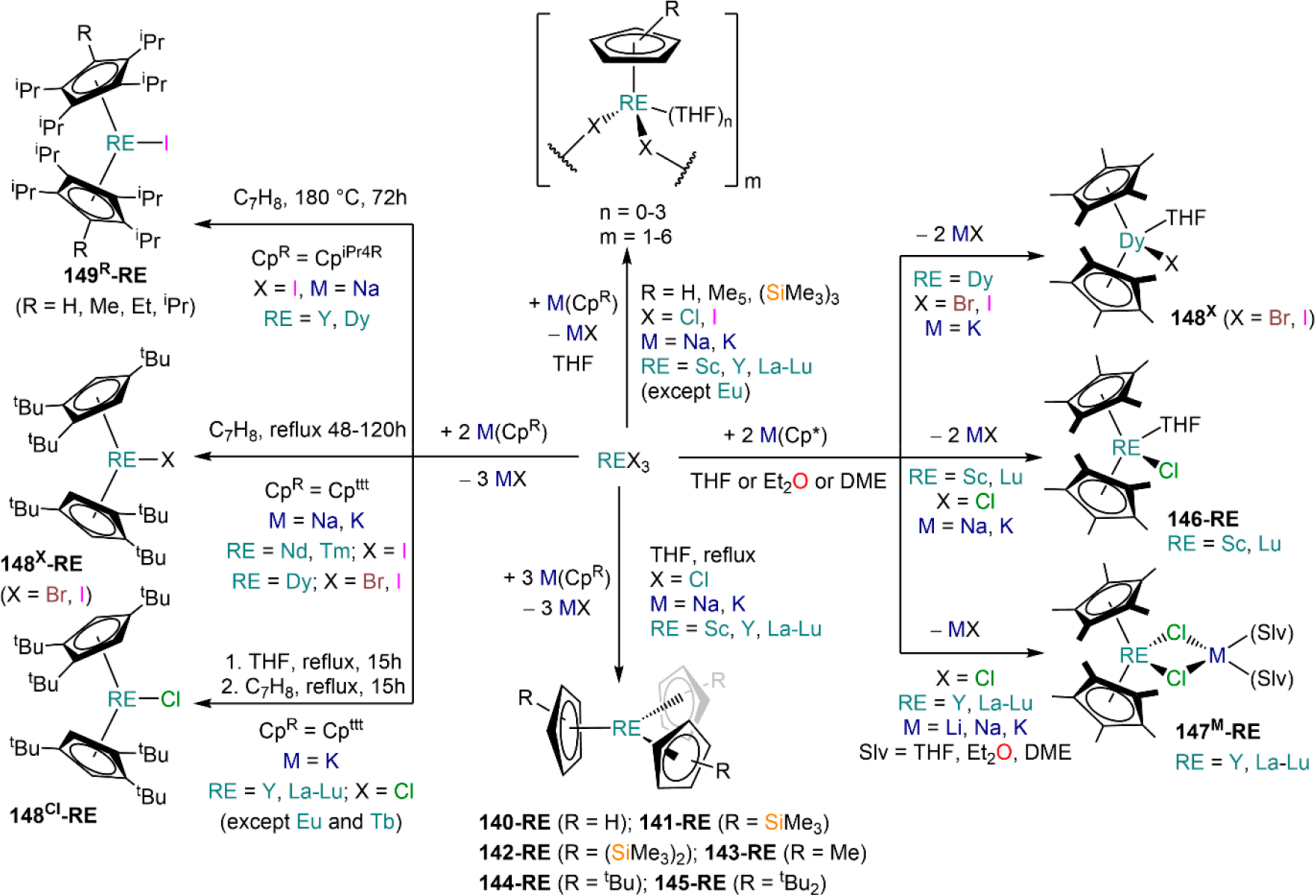
Selected Examples of Salt Elimination Reactions of REX_3_ with Cp Salts

Because of all the possible combinations of RE trihalides, ligand transfer reagents, stoichiometric ratios and reaction conditions, the number of salt elimination applications of REX_3_ salts is enormous, and a full account is beyond the scope of this work. Herein, some key examples are illustrated of their use with cyclopentadienyl salts to form homoleptic, RE(Cp^R^)_3_, and heteroleptic complexes, RE(Cp^R^)_2_X and RE(Cp^R^)X_2_ (Scheme 46). In 1954 Wilkinson and Birmingham used anhydrous RECl_3_ (RE = Sc, T, La, Ce, Pr, Nd, Sm, and Gd) to synthesize RE(Cp)_3_ (**140-RE**) complexes *via* salt elimination with Na(Cp), thus pioneering a synthetic route which quickly became a gold-standard for RE synthetic chemists.^2,402^ Group 1 salts of a variety of substituted Cp ligands have been used in salt metathesis reactions with RE halides; in some cases, group 2 (Be, Mg) and Tl(I) reagents have also been employed.^327^ Salt elimination reactions between RECl_3_ and Na(Cp) or K(Cp) salts are usually carried out in THF or Et_2_O and under reflux conditions.^2,402^ The same strategy is applicable to substituted Cps, though it becomes increasingly difficult to displace all three halides as the steric demands of the ligands increase; RECl_3_ are the salts that usually give the best results when attempting to isolate homoleptic RE(Cp^R^)_3_ compounds with the smaller Cp ligands, *e.g.*, Cp′ (**141-RE**),^403−408^ Cp″ (**142-RE**),^404^ Cp^Me^ (**143-RE**),^404,409−411^ Cp^t^ (**144-RE**),^412,413^ and Cp^tt^ (**145-RE**).^414^ Heteroleptic species can be obtained by altering the stoichiometric ratio of these reactions, thereby using a 2:1 ratio for metallocene-type complexes, RE(Cp^R^)_2_X,^282,283,415−419^ and 1:1 ratio for monoring complexes, RE(Cp^R^)X_2_ (Scheme 46).^355,420,421^ Metallocene-type complexes are particularly desirable as they provide a fine control of the coordination sphere of the metal center, with the additional possibility of functionalizing the complexes by displacing the halide ligand.^282,283,415−419^ However, often alkali metal salts are occluded in the resulting complexes; this occurrence can sometimes be avoided by using different combinations of alkali metal salts. Lappert *et al.* reported the reactivity of RECl_3_ (RE = Sc, Y, La, Ce, Pr, Nd, Yb) with two equivalents of LiCp″, in the attempt to obtain metallocene type complexes RE(Cp″)_2_Cl.^422^ However, the reactivity resulted in the formation of LiCl adducts of formula [RE(Cp″)_2_(μ-Cl)_2_Li(THF)_2_], which can be converted into the desired species, in this case dimers [{RE(Cp″)_2_(μ-Cl)}_2_], upon recrystallization or sublimation.^422^ Xie and co-workers showed that by using NaCp″ the alkali metal-free compounds [{RE(Cp″)_2_(μ-Cl)}_2_] are obtained instead.^423^ Nonetheless, it is not always possible to avoid alkali metal occlusion in salt elimination reaction, as shown with the synthesis of heteroleptic RE(III) metallocenes with Cp* as supporting ligand. With the exception of [Sc(Cp*)_2_(Cl)] (**146-Sc**)^424^ and [Lu(Cp*)_2_(Cl)(THF)] (**146-Lu**),^425^ all the complexes obtained from salt elimination reactions between RECl_3_ and M(Cp*) afford MCl adducts [RE(Cp*)_2_(μ-Cl)_2_M(THF)_2_] (**147**^**M**^**-RE**; Slv = Et_2_O, THF, DME; M = Li, Na, K; RE = Y,^426^ Ce,^427,428^ La,^429^ Pr,^429^ Nd,^430^ Sm,^431^ Gd,^429^ Tb,^429^ Dy,^429,432^ Ho,^429^ Er,^429^ Tm,^429,433^ Yb,^429,434^ Lu^435^) (Scheme 46). Despite the drawbacks of these salt elimination reactions, “ate” complexes are still excellent starting materials which can be further functionalized and converted into very useful reagents. Nonetheless, Meng *et al.* demonstrated that with the use of different RE starting materials it is possible to avoid alkali metal occlusion and were able to obtain [Dy(Cp*)_2_(X)(THF)] (**148**^**X**^; X = Br, I) from the reaction between KCp* and DyBr_3_ or DyI_3_ in THF.^432^ It is also possible to laboriously convert **147**^**M**^**-RE** into monomeric complexes [RE(Cp*)_2_(Cl)(THF)] or solvent-free [RE(Cp*)_2_(Cl)]_*n*_, usually *via* sublimation under high vacuum and subsequent recrystallization.^428,433^ Monomeric solvent-free RE(III) metallocenes [RE(Cp^R^)X] can be obtained *via* salt metathesis by employing bulkier Cp ligands, such as Cp^ttt^ (**148**^**X**^**-RE**; X = Cl, Br, I; RE = Y, La–Lu (except Eu and Tb)) and Cp^iPr4R^ (**149**^**R**^**-RE**; R = H, Me, Et, ^i^Pr), with anhydrous REX_3_ (X = Cl, Br, I) (Scheme 46).^282,283,415−419^

## 5 Borohydrides

**Figure 8:**
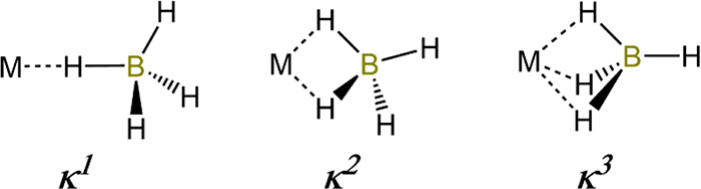
Typical binding modes of the borohydride ligand in metal complexes.

RE borohydrides (Table 5) have gained increasing popularity over the last three decades.^17−19^ These synthons have found numerous applications in salt metathesis reactions, especially for the stabilization of heteroleptic compounds of formulas Ln(L)(BH_4_), RE(L)(BH_4_)_2_, and RE(L)_2_(BH_4_). Part of this interest is due to their accessibility and ease of preparation, combined with their versatile coordination chemistry. The borohydride ligand displays various binding modes, mainly acting as mono-, bi-, or tridentate donor (Figure 8), and provides a certain degree of flexibility in salt elimination reactions compared to RE halides.^16,17^ Though the BH_4_^–^ ligand can act as a *pseudo*-halide, it can also occupy multiple coordination sites when binding in a *k*^2^- or *k*^3^-fashion.^19^ Furthermore, BH_4_^–^ is effectively a masked hydride which can readily decompose into H^–^ and “BH_3_”,^19^ or can be removed with hydride-abstracting agents.^436−439^ It is noteworthy that borohydride compounds have more covalent character compared to halides; for this reason, Ln(BH_4_)_2_ and RE(BH_4_)_3_ have a relatively higher solubility than their halide counterparts.^19^ Crucially, the formation of higher aggregates and “ate” complexes are less likely when these materials are employed in salt elimination reactions instead of REX_3_, thus making them excellent candidates for obtaining discrete molecular entities.^19,20^

**Table 5:** Anhydrous and THF Adducts of RE Borohydrides^a^

	Ln(BH_4_)_2_	RE(BH_4_)_3_
Sc		Sc(BH_4_)_3_^479^
		[Sc(BH_4_)_3_(THF)_2_]^480,481^
		*Sc(BH*_*4*_*)*_*3*_*(THF)*(458)
		*Sc(BH*_*4*_*)*_*3*_*(THF)*_*1.5*_(466)
		*Sc(BH*_*4*_*)*_*3*_*(THF)*_*2*_(480)
Y		Y(BH_4_)_3_^461,482,483^
		[Y(BH_4_)_3_(THF)_3_]^437,484^
		[Y(BH_4_)_2_(THF)_4_][Y(BH_4_)4]^485^
		*Y(BH*_*4*_*)*_*3*_*(THF)*(448)
		[Y(BH_4_)_2_(THF)_5_][BPh_4_]^473^
La		La(BH_4_)_3_^486^
		[La(BH_4_)_3_(THF)_3.5_]^b^
		[La(BH_4_)_3_(THF)_4_]^436^
		*La(BH*_*4*_*)*_*3*_*(THF)*_*3*_(449,455,487)
		*La(BH4)*_*3*_*(THF)*_*2*_(488)
Ce		Ce(BH_4_)_3_^489,490^
		[Ce(BH_4_)_3_(THF)_3.5_]^436,454^
		*Ce(BH*_*4*_*)*_*3*_*(THF)*_*3*_(449)
		[Ce(BH_4_)_2_(THF)_5_][BPh_4_]^474^
Pr		Pr(BH_4_)_3_^490,491^
		[Pr(BH_4_)_3_(THF)_3.5_]^492^
		*Pr(BH*_*4*_*)*_*3*_*(THF)*_*3*_(449)
Nd		Nd(BH_4_)_3_^339^
		[Nd(BH_4_)_3_(THF)_3.5_]^438,492^
		[Nd(BH_4_)_3_(THF)_3_]^449,453^
		[Nd(BH_4_)_2_(THF)_5_][BPh_4_]^473^
		[Nd(BH_4_)_2_(THF)_5_][B(C_6_F_5_)_4_]^457^
Sm	Sm(BH_4_)_2_^462,463,493,494^	Sm(BH_4_)_3_^488^
	[Sm(BH_4_)_2_(THF)_2_]_∞_^447^	[Sm(BH_4_)_3_(THF)_3_]^449,487,492^
		[Sm(BH_4_)_2_(THF)_5_][BPh_4_]^473^
Eu	Eu(BH_4_)_2_^462,463,494^	[Eu(BH_4_)_3_(THF)_3_]^492^
	[Eu(BH_4_)_2_(THF)_2_]_∞_^442^	
	[Eu(BH_4_)(THF)_5_][BPh_4_]_∞_^442^	
Gd		Gd(BH_4_)_3_^461−463^
		[Gd(BH_4_)_3_(THF)_3_]^449,488^
		*Gd(BH*_*4*_*)*_*3*_*(THF)*_*2*_(488)
		*Gd(BH*_*4*_*)*_*3*_*(THF)*(448)
Tb		Tb(BH_4_)_3_^460,462^
		[Tb(BH_4_)_3_(THF)_3_]^437,449^
		*Tb(BH*_*4*_*)*_*3*_*(THF)*(448)
Dy		Dy(BH_4_)_3_^461,463^
		[Dy(BH_4_)_3_(THF)_3_]^439,449,487^
		[Dy(BH_4_)_2_(THF)_5_][BPh_4_]^475,476^
Ho		Ho(BH_4_)_3_^462,463^
		*Ho(BH*_*4*_*)*_*3*_*(THF)*_*3*_(449)
		*Ho(BH*_*4*_*)*_*3*_*(THF)*(448)
Er		Er(BH_4_)_3_^463,495^
		[Er(BH_4_)_3_(THF)_3_]^449,488,492^
Tm	[Tm(BH_4_)_2_(DME)_2_]^443^	Tm(BH_4_)_3_^462,463^
		*Tm(BH*_*4*_*)*_*3*_*(THF)*_*3*_(443,449)
		*Tm(BH*_*4*_*)*_*3*_*(THF)*(448)
Yb	Yb(BH_4_)_2_^463^	Yb(BH_4_)_3_^463,488^
	[Yb(BH_4_)_2_(THF)_2_]^442^	[Yb(BH_4_)_3_(THF)_3_]^449,492^
	[Yb(BH_4_)(THF)_5_][BPh_4_]^442^	
Lu		Lu(BH_4_)_3_^463,488^
		[Lu(BH_4_)_3_(THF)_3_]^449,487,488^
		*Lu(BH*_*4*_*)*_*3*_*(THF)*(448)

a Compounds in italics have not been structurally authenticated.

b CSD entry 1198893.

### 5.1 Divalent Ln Borohydrides

**Scheme 47:**
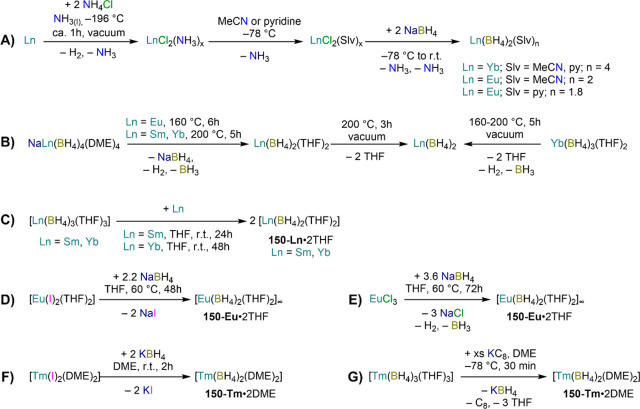
Synthesis of Divalent Ln(BH_4_)_2_ Complexes^19,440−443^

The syntheses of divalent Ln borohydrides [Ln(BH_4_)_2_(MeCN)_*n*_] (Ln = Eu, *n* = 2; Ln = Yb, *n* = 4) and [Ln(BH_4_)_2_(py)_*n*_] (Ln = Yb, *n* = 4; Ln = Eu, *n* = 1.8) were first reported by Shore and co-workers in 1991.^440^ To obtain these materials, the authors first prepared ammoniacal solutions of LnCl_2_ by reacting metal powders with NH_4_Cl in liquid ammonia; removal of ammonia afforded solid ammoniacal salts LnCl_2_(NH_3_)_*x*_ which were converted into MeCN or pyridine solvates, LnCl_2_(Slv)_*x*_ (Slv = MeCN, pyridine), and then reacted with two equivalents of NaBH_4_ (Scheme 47, **A**).^440^ Makhaev and Borisov were able to obtain Sm(BH_4_)_2_, Eu(BH_4_)_2_, and Yb(BH_4_)_2_ from the thermal decomposition of the corresponding NaLn(BH_4_)_4_ precursor (Scheme 47, **B**);^441^ interestingly, in the case of Yb it was also possible to obtain the divalent species from thermal decomposition of Yb(BH_4_)_3_(THF)_2_.^441^ A more convenient approach was devised by Visseaux and co-workers, who showed that treatment of [Sm(BH_4_)_3_(THF)_3_] with Sm metal in THF at room temperature can afford [Sm(BH_4_)_2_(THF)_2_] (**150-Sm·**2THF) in very good yields (Scheme 47, **C**).^19^ The same method was applied by Roesky and co-workers to synthesize the Yb analogue [Yb(BH_4_)_2_(THF)_2_] (**150-Yb**). [Eu(BH_4_)_2_(THF)_2_]_**∞**_ (**150-Eu·**2THF) was also prepared by Roesky and co-workers either *via* salt metathesis reaction between [Eu(I)_2_(THF)_2_] and NaBH_4_ or from the reaction between EuCl_3_ and NaBH_4_ with the concomitant formation of H_2_ and “BH_3_” (Scheme 47, **D** and **E**).^442^ Finally, Visseaux, Nief, and co-workers obtained [Tm(BH_4_)_2_(DME)_2_] (**150-Yb·**2THF) from direct salt metathesis between [Tm(I)_2_(DME)_3_] and KBH_4_ (Scheme 47, **F**) or by reduction of Tm(III) precursor [Tm(BH_4_)_3_(THF)_3_] with KC_8_ (Scheme 47, **G**).^443^ Interestingly, the authors noted that comproportionation reaction between [Tm(BH_4_)_3_(THF)_3_] and Tm did not yield a reduced Tm(II) species.

**Scheme 48:**
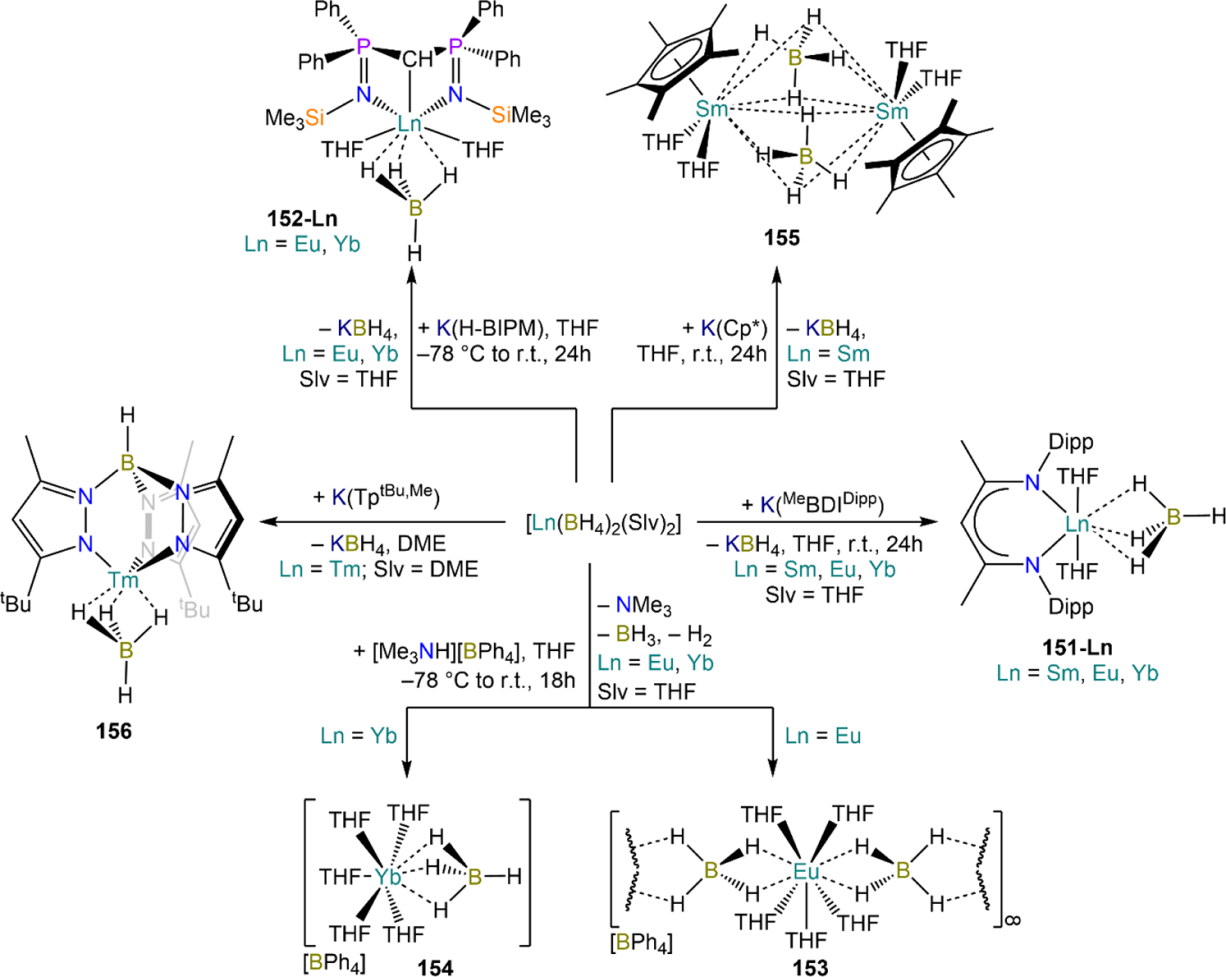
Selected Applications of Ln(BH_4_)_2_ Starting Materials^442−444,447^

Divalent borohydrides have found application as salt metathesis precursors with various ligands, including BDIs (**151-Ln**; Ln = Sm, Eu, Yb),^444^ bis-iminophosphorano-methanides,^442,445^ tris-pyrazolylborate,^443^ substituted pyrrolyls,^446^ and cyclopentadienyls.^447^ Roesky and co-workers reacted K(H-BIPM) with [Ln(BH_4_)_2_(THF)_2_] (Ln = Eu, Yb) to give heteroleptic borohydride complexes of formula [Ln(H-BIPM)(BH_4_)(THF)_2_] (**152-Ln**; Ln = Eu, Yb) (Scheme 48).^442^ The dual behavior of these reagents (*pseudo*-halide and masked hydride) was highlighted by the same authors who synthesized cationic [Eu(BH_4_)(THF)_5_][BPh_4_]_∞_ (**153**) and [Yb(BH_4_)(THF)_5_][BPh_4_] (**154**) by reacting [Ln(BH_4_)_2_(THF)_2_] with [Me_3_NH][BPh_4_] (Scheme 48);^442^ in this reaction the hydride is abstracted to form H_2_ with concomitant formation of “BH_3_” and NEt_3_. Finally, Momin *et al.* used salt metathesis to synthesize the dimeric Sm(II) monoring complex [{Sm(Cp*)_2_(μ-BH_4_)(THF)_2_}_2_] (**155**) and [Tm(Tp^tBu,Me^)(BH_4_)] (**156**) (Scheme 48), which have both been used as initiators for the polymerization of ε-caprolactone.^443,447^

### 5.2 Trivalent RE Borohydrides

**Scheme 49:**
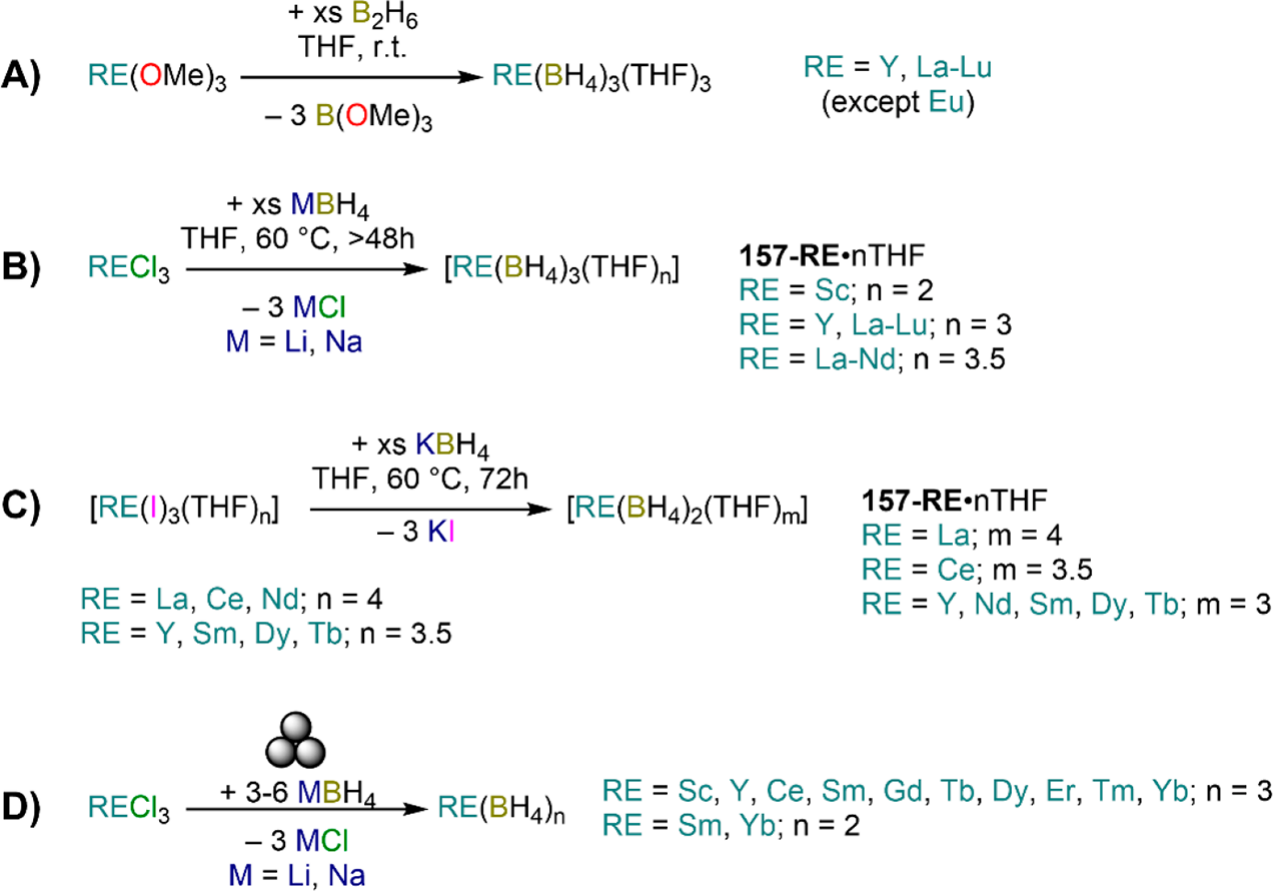
Synthesis of RE(BH_4_)_3_(THF)_*n*_ Complexes *via* Solution Methods and Synthesis of Solvent-free Ln(BH_4_)_2_ and RE(BH_4_)_3_*via* Ball-Milling^436−438,444,448,449,452−462^

Trivalent RE borohydrides, RE(BH_4_)_3_, are known for all the RE metals (Table 5 and Scheme 49). With the exception of Sc, Ce, and Eu, these species were first obtained as THF solvated salts RE(BH_4_)_3_(THF)_3_ from the reaction of RE(OMe)_3_ with B_2_H_6_ in THF at room temperature (Scheme 49, **A**).^448,449^ A more convenient approach is based on simple metathetical reactivity between RECl_3_ and MBH_4_ (M = Li, Na, K) in THF. This method was first attempted by Rossmanith and Muckenhuber in 1959, but in their methodology they could only obtain partial conversion to mixed borohydride-chloride salts RE(BH_4_)_2_Cl.^450,451^ Moreover, Andersen reported that reactivity of LiBH_4_ with NdCl_3_ was sluggish and did not afford the desired Nd(BH_4_)_3_ product.^452^ However, when these reactions are carried out under reflux for at least 48 h a good conversion to solvated RE(BH_4_)_3_ complexes is obtained, as demonstrated by Ephritikhine and co-workers with the preparation of [Nd(BH_4_)_3_(THF)_3_] (**157-Nd**·3THF, Scheme 49, **B**),^453^ and this method has been successfully applied to the synthesis of tris-borohydrides with all REs.^444,453−458^ Traces of halides are often present in the final products despite very long reaction times and use of large excess of MBH_4_.^456^ Mills and co-workers developed this methodology further by using triiodide RE salts with a large excess of KBH_4_ (Scheme 49, **C**) and were able to identify halide impurities *via* X-ray studies;^436−438^ the percentage of iodide present in [La(BH_4_)_3_(THF)_4_]^436^ (**157-La**·4THF) was measured at 3%, whereas the halide content in [Y(BH_4_)_3_(THF)_3_] (**157-Y**·3THF) and [Tb(BH_4_)_3_(THF)_3_] (**157-Yb**·3THF) was significantly higher (*ca.* 15%).^437^ Adducts with other Lewis bases are also reported, including pyridine and NH_3_. Finally, solvent-free RE(BH_4_)_3_ of several REs (Sm, Gd, Tb, Dy, Er, Tm, and Yb) have been obtained by Hauback, Jensen, and co-workers *via* ball-milling of RECl_3_ with LiBH_4_,^459−462^ including divalent Sm(BH_4_)_2_ and Yb(BH_4_)_2_ (Scheme 49, **D**). Solvent-free RE(BH4)_3_ can also be obtained from the reaction of REH_3_ with H_3_B·SMe_2_, both with dry (ball-milling) and solution methods.^463^

**Scheme 50:**
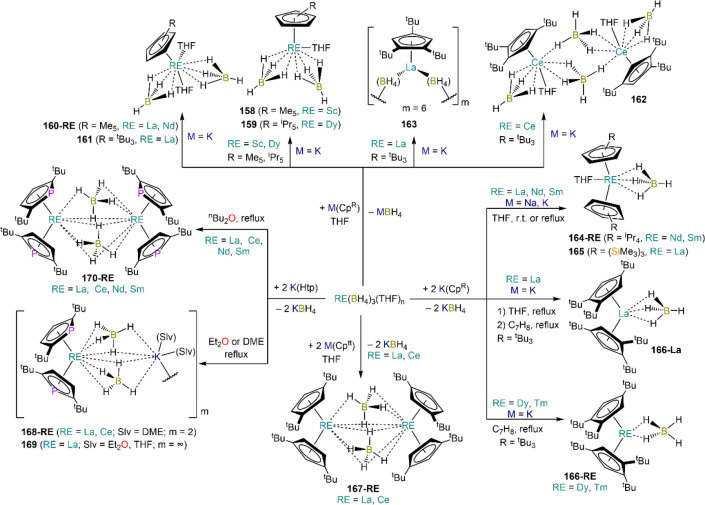
Selected Examples of Salt Elimination Reactions between RE(BH_4_)_3_ Reagents and Cyclopentadienyl^436,439,453,466−469^ and Phospholyl^438^ Ligand Transfer Reagents

Much like REX_3_ salts, trivalent RE borohydrides are very efficient reagents for salt metathesis reactions, and they have been used extensively in cyclopentadienyl chemistry for the synthesis of monoring and metallocene-type complexes (Scheme 50). It is noteworthy that RE(III) metallocenes have also bene synthesized by reacting Cp salts with RE(BH_4_)_3_ generated *in situ* from RECl_3_ and NBH_4_.^464^ Monoring complexes, RE(Cp)X_2_, can be particularly challenging to isolate when using halide starting materials, while they are usually readily accessible with borohydride reagents owing to the stabilizing properties of the borohydride ligand and its flexible coordination modes.^465^ This is exemplified by the variety of coordination motifs exhibited by this family of complexes: (1) monoring complexes, *e.g.* [RE(Cp^R^)(BH_4_)_2_(THF)] (**158**, Cp^R^ = Cp*, RE = Sc;^466^**159**, Cp^R^ = Cp^iPr5^; RE = Dy^439^) and [RE(Cp^R^)(BH_4_)_2_(THF)_2_] (**160-RE**, Cp^R^ = Cp*, RE = La,^467^ Nd;^453,468^**161**, Cp^R^ = Cp^ttt^, RE = La^436^); (2) borohydride-bridged dimers, *e.g.* [{Ce(Cp^ttt^)(μ-BH_4_)_2_(THF)}_2_] (**162**);^436^ (3) clusters, *e.g.* [{La(Cp^ttt^)(BH_4_)_2_}_6_] (**163**)^436^ (Scheme 50). Metallocene-type complexes, [RE(Cp^R^)_2_(BH_4_)], can be obtained with several supporting Cp ligands (**164-RE**, Cp^R^ = Cp^iPr4^, RE = Nd, Sm;^469^**165**, Cp^R^ = Cp‴, RE = La;^436^**166-RE**, Cp^R^ = Cp^ttt^, RE = La, Dy, Tm);^436^ in a similar vein to heteroleptic halide-Cp RE complexes, the formation of dimeric species is observed when steric hindrance of the ligands is reduced, *e.g.*, [{RE(Cp^tt^)_2_(μ-BH_4_)}_2_] (**167-RE**; RE = La, Ce).^436^ Unlike with halide-based starting materials, “ate” complex formation is usually not an issue in these methodologies. However, when similar reactions were carried out by Liu *et al.* between phospholyl K(Htp) (Htp = {C_4_H_2_P^t^Bu_2_-2,5}^−^) and various RE borohydrides (RE = La, Ce, Nd, Sm), the leading to the isolation of “ate” complexes [{RE(Htp)_2_(μ-BH_4_)_2_K(μ-DME)_2_}_2_] (**168-RE**; RE = La, Ce) and [La(Htp)(μ-Htp)(μ-BH_4_)_2_K(OEt_2_)(THF)]_∞_ (**169**) (Scheme 50) were isolated.^438^ Nonetheless, the authors were able to avoid salt occlusion by performing the reactions in ^n^Bu_2_O under reflux, which gave borohydride-bridged dimers [{RE(Htp)_2_(μ-BH_4_)}_2_] (**170-RE**; RE = La, Ce, Nd, Sm).^438^

**Scheme 51:**
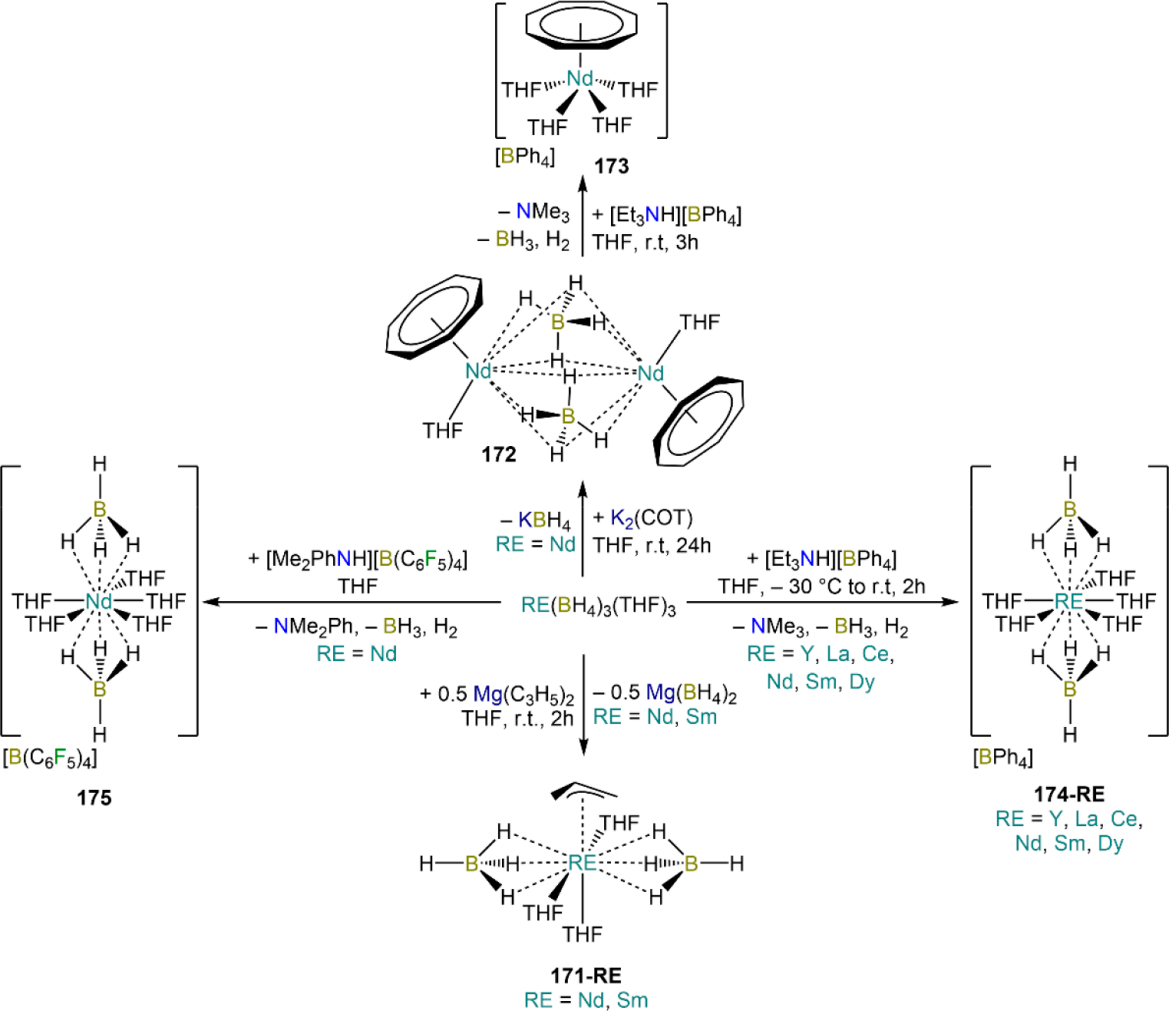
Synthesis of Mixed Allyl-borohydride Complexes^470^ and Protonolysis Reactions with Ammonium Salts^457,473−476^

While RE halides can be converted into alkyls and allyls from the reaction with organolithium and Grignard reagents, analogous reactivity of RE borohydrides is not as straightforward. Visseaux and co-workers reacted Nd(BH_4_)_3_ and Sm(BH_4_)_3_ with Grignard reagents and could not obtain pure products.^470^ Nonetheless, reactions with half an equivalent of Mg(C_3_H_5_)_2_ generated the heteroleptic allyl derivatives [RE(BH_4_)_2_(C_3_H_5_)(THF)_3_] (**171-RE**; RE = Nd, Sm) in crystalline form and with excellent yields (Scheme 51).^470^ In addition to this, RE borohydrides can be used in protonolysis reactions, similar to their divalent analogues (*vide supra*, Scheme 50). This particular application was pioneered by Ephritikhine and co-workers, inspired by their previous work with An borohydrides, which can form cationic species upon treatment with ammonium salts.^471^ Accordingly, Guillaume *et al.* synthesized the dimeric COT complex [{Nd(COT)(μ-BH_4_)(THF)}_2_] (**172**) *via* salt elimination reaction between K_2_(COT) and Nd(BH4)_3_(THF)_3_ and then reacted it with [Et_3_NH][BPh_4_] to give the cationic complex [Nd(COT)(THF)_4_][BPh_4_] (**173**) (Scheme 51).^472^ Furthermore, RE(BH_4_)_3_ starting materials can also be converted cleanly into cationic separated ion pair complexes [RE(BH_4_)_2_(THF)_5_][BPh_4_] (**174-RE**; RE = Y,^473^ La,^473^ Ce,^474^ Nd,^473,474^ Sm,^473^ Dy)^475,476^ upon reaction with [Et_3_NH][BPh_4_] (Scheme 51). Visseaux and co-workers obtained also the analogous complex [Nd(BH_4_)_2_(THF)_5_][B(C_6_F_5_)_4_] (**175**) from the reaction between [Me_2_PhNH][B(C_6_F_5_)_4_] and [Nd(BH_4_)_3_(THF)_3_].^457^ Other reagents that have been used with borohydride complexes for the formation of cations are highly electrophilic hydride abstracting reagents [Ph_3_C][B(C_6_F_5_)_4_]^437^ and [(Me_3_Si)_2_H][B(C_6_F_5_)_4_].^439,477,478^

## 6 Nitrogen Donors

Amide ligands are ubiquitous in RE chemistry and have found a multitude of applications.^22−24^ Among these, silylamides are probably the most popular ligand class; Bradley *et al.* pioneered the use of the bis(trimethylsilyl)amide ligand {N(SiMe_3_)_2_}^−^ in RE coordination chemistry in the early 1970s,^496,497^ and their work became a centerpiece of modern RE and f-element synthetic chemistry.^24^ More recently, the conjugated base of tetramethylsilazane, {N(SiHMe_2_)_2_}^−^, has also become a very important ligand in RE chemistry.^498^ Together with possessing a rich coordination chemistry, silylamides are also excellent Brønsted bases and are therefore extremely useful for protonolysis reactivity.^22−24^ Because of this, RE silylamides have now become essential starting materials for synthetic chemistry. Other RE amides have also been used in protonolysis reactions (*e.g.*, Ln(NH_2_)_2_ and RE(NH_2_)_3_,^499^ RE(NMe_2_)_3_, RE(N^iPr2^)_3_, and RE(NCy_2_)_3_),^22−24^ but these will not be covered in this review as their uses in RE chemistry are limited compared to the vast applications of silylamides.

### 6.1 Divalent Ln Silylamides

**Scheme 52:**
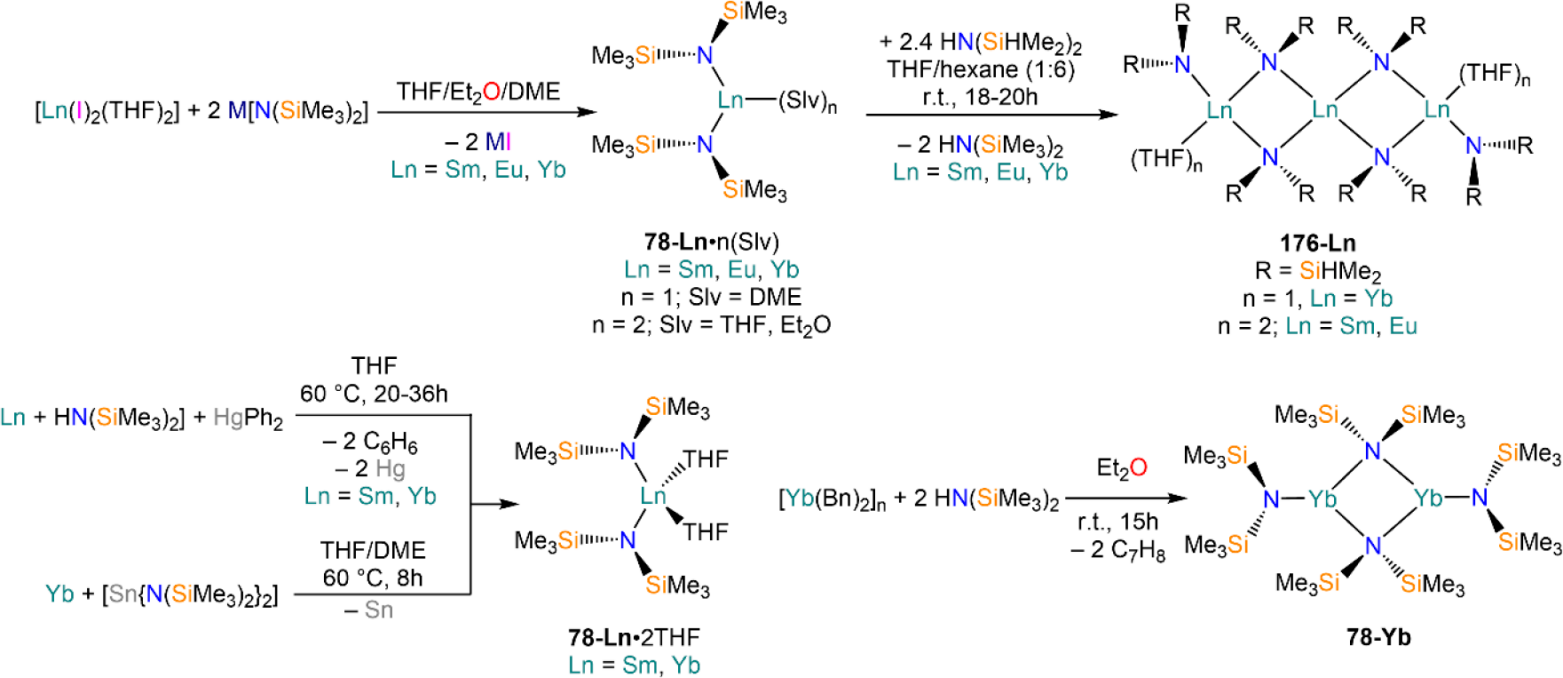
Synthesis of Ln(II) Silylamides **78-Ln** and **176-Ln**(90,162,500−502,504,506,507)

The first Ln(II) silylamides, Ln{N(SiMe_3_)_2_}_2_ (Ln = Yb, Eu), were originally synthesized by Tilley *et al. via* salt elimination reactions between Na[N(SiMe_3_)_2_] and EuI_2_ or YbI_2_ (divalent salts were both been obtained from reactions in liquid ammonia) and were isolated as Et_2_O or DME adducts, [Ln{N(SiMe_3_)_2_}_2_(Slv)_2_] (**78-Ln**; Ln = Yb, Eu; Slv = Et_2_O, DME) (Scheme 52 and Table 6).^500−502^ It is noteworthy that **78-Eu**·2THF was originally obtained from the reduction of Eu{N(SiMe_3_)_2_}_2_Cl with sodium napthalenide; however, current salt elimination methodologies are far more convenient.^503^ The Sm analogue [Sm{N(SiMe_3_)_2_}_2_(THF)_2_] (**78-Sm**·2THF) was obtained by Evans and co-workers with similar methodologies using [Sm(I)_2_(THF)_2_] and Na[N(SiMe_3_)_2_].^288^**78-Yb** can also be synthesized *via* (1) RT reaction of Yb metal with [Sn{N(SiMe_3_)_2_}_2_],^90^ (2) RTP reaction of Yb metal with HgPh_2_ and HN(SiMe_3_)_2_,^162^ and (3) protonolysis between Yb(Bn)_2_ (Bn = CH_2_Ph) and HN(SiMe_3_)_2_^504^ (Scheme 52; see section 3.4 for details on RT and RTP methods). All these alternative methods are highly desirable to avoid the presence of alkali metal or halide impurities. Additionally, the protonolysis route affords **78-Yb** as a solvent-free species, [Yb{N(SiMe_3_)_2_}{μ-N(SiMe_3_)_2_}]_2_, without the need to use labor-intensive desolvation protocols.^505^ The Tm analogue Tm{N(SiMe_3_)_2_}_2_ has been obtained as a transient species by Evans and co-workers, but its isolation and full characterization have not been achieved to date.^290^

**Table 6:** Divalent and Trivalent RE Silylamides and Tetravalent Ce Amides Used as Synthetic Precursors^a^

	Ln{N(SiR_3_)_2_}_2_	RE{N(SiR_3_)_2_}_3_	Ce(NR_2_)_4_
Sc		[Sc{N(SiMe_3_)_2_}_3_]^539,540^	
		[Sc{N(SiHMe_2_)_2_}_3_(THF)]^498^	
Y		[Y{N(SiMe_3_)_2_}_3_]^541^	
		[Y{N(SiMe_3_)_2_}_3_(THF)]^b^	
		[Y{N(SiHMe_2_)_2_}_2_{μ-N(SiHMe_2_)_2_}]_2_^542^	
		Y{N(SiMe_3_)_2_}_3_(THF)_2_]^543^	
La		*La{N(SiMe*_*3*_*)*_*2*_*}*_*3*_(342,496,497,544)	
		[La{N(SiHMe_2_)_2_}_2_{μ-N(SiHMe_2_)_2_}]_2_^498,517^	
		[La{N(SiHMe_2_)_2_}_3_(THF)_2_]^498^	
Ce		[Ce{N(SiMe_3_)_2_}_3_]^496,497,514,545^	[Ce{N(SiHMe_2_)_2_}_4_]^535^
		[Ce{N(SiHMe_2_)_2_}_3_(THF)_2_]^546^	[Ce(N^iPr2^)_4_]^536^
Pr		*[Pr{N(SiMe*_*3*_*)*_*2*_*}*_*3*_*]*(496,497)	
		[Pr{N(SiHMe_2_)_2_}_3_(THF)_2_]^546^	
Nd		[Nd{N(SiMe_3_)_2_}_3_]]^496,497,547^	
		[Nd{N(SiHMe_2_)_2_}_3_(THF)_2_]^543^	
Sm	[Sm{N(SiMe_3_)_2_}_2_(THF)_2_]^288^	[Sm{N(SiMe_3_)_2_}_3_]^496,497,548^	
	[Sm_3_{N(SiHMe_2_)_2_}_6_(THF)_2_]^506^	[Sm{N(SiMe_3_)_2_}_3_(THF)]^549^	
		[Sm{N(SiHMe_2_)_2_}_3_(THF)_2_]^550^	
Eu	*Eu{N(SiMe*_*3*_*)*_*2*_*}*_*2*_*(Et*_*2*_*O)*_*2*_(503,551)	[Eu{N(SiMe_3_)_2_}_3_]^496,497,540^	
	[Eu{N(SiMe_3_)_2_}_2_(THF)_2_]^503,551^	[Eu{N(SiHMe_2_)_2_}_3_(THF)_2_]^502^	
	[Eu{N(SiMe_3_)_2_}_2_(DME)_2_]^501,503^		
	[Eu_3_{N(SiHMe_2_)_2_}_6_(THF)_2_]^502^		
Gd		*Gd{N(SiMe*_*3*_*)*_*2*_*}*_*3*_(496,497)	
		[Gd{N(SiHMe_2_)_2_}_3_(THF)_2_]^519^	
Tb		[Tb{N(SiMe_3_)_2_}_3_]^496,497,514^	
Dy		[Dy{N(SiMe_3_)_2_}_3_]^543,552^	
Ho		*Ho{N(SiMe*_*3*_*)*_*2*_*}*_*3*_(496,497)	
		[Ho{N(SiHMe_2_)_2_}_3_(THF)_2_]^553^	
Er		[Er{N(SiMe_3_)_2_}_3_]^543^	
		*Er{N(SiHMe*_*2*_*)*_*2*_*}*_*3*_(498)	
Tm	*Tm{N(SiMe*_*3*_*)*_*2*_*}*_*2*_*(THF)x*(290)	[Tm{N(SiMe_3_)_2_}_3_]^543^	
Yb	*Yb{N(SiMe*_*3*_*)*_*2*_*}*_*2*_*(Et*_*2*_*O)*_*2*_(501)	[Yb{N(SiMe_3_)_2_}_3_]^496,497,557^	
	[Yb{N(SiMe_3_)_2_}_2_(THF)_2_]^212,554−556^	[Yb{N(SiHMe_2_)_2_}_3_(THF)_2_]^518^	
	*Yb{N(SiMe*_*3*_*)*_*2*_*}*_*2*_*(DME)*_*2*_(501)		
	[Yb{N(SiMe_3_)_2_}_2_]_2_^504^		
	[Yb{N(SiMe_3_)_2_}(BPh_4_)]^511^		
	[Yb_3_{N(SiHMe_2_)_2_}_6_(THF)_2_]^550^		
Lu		[Lu{N(SiMe_3_)_2_}_3_]^496,497,558^	
		[Lu{N(SiHMe_2_)_2_}_3_(THF)_2_]^498^	

a Compounds in italics have not been structrally autheticated.

b CSD entry 2056064.

Anwander and co-workers were also able to introduce the smaller silylamide {N(SiHMe_2_)_2_}^−^ to divalent Ln chemistry.^506^ In their work, they observed that salt metathesis reaction between [Sm(I)_2_(THF)_2_] and Li[N(SiHMe_2_)_2_] could not deliver clean products, so they decided to explore a protonolysis route.^506^**78-Sm**·2THF was an ideal candidate as precursor for this strategy, owing to the favorable difference in p*K*_a_ between HN(SiHMe_2_)_2_ and HN(SiMe_3_)_2_ (22.6 and 25.8 respectively).^506^ The reaction of **78-Sm**·2THF with HN(SiHMe_2_)_2_ afforded oligomeric silylamide [Sm_3_{N(SiHMe_2_)_2_}_6_(THF)_2_] (**176-Sm**),^506^ and the same method has been used for the synthesis of [Yb_3_{N(SiHMe_2_)_2_}_6_(THF)]^507^ (**176-Yb**) and [Eu_3_{N(SiHMe_2_)_2_}_6_(THF)_2_] (**176-Eu**) (Scheme 52 and Table 6).^502^

**Scheme 53:**
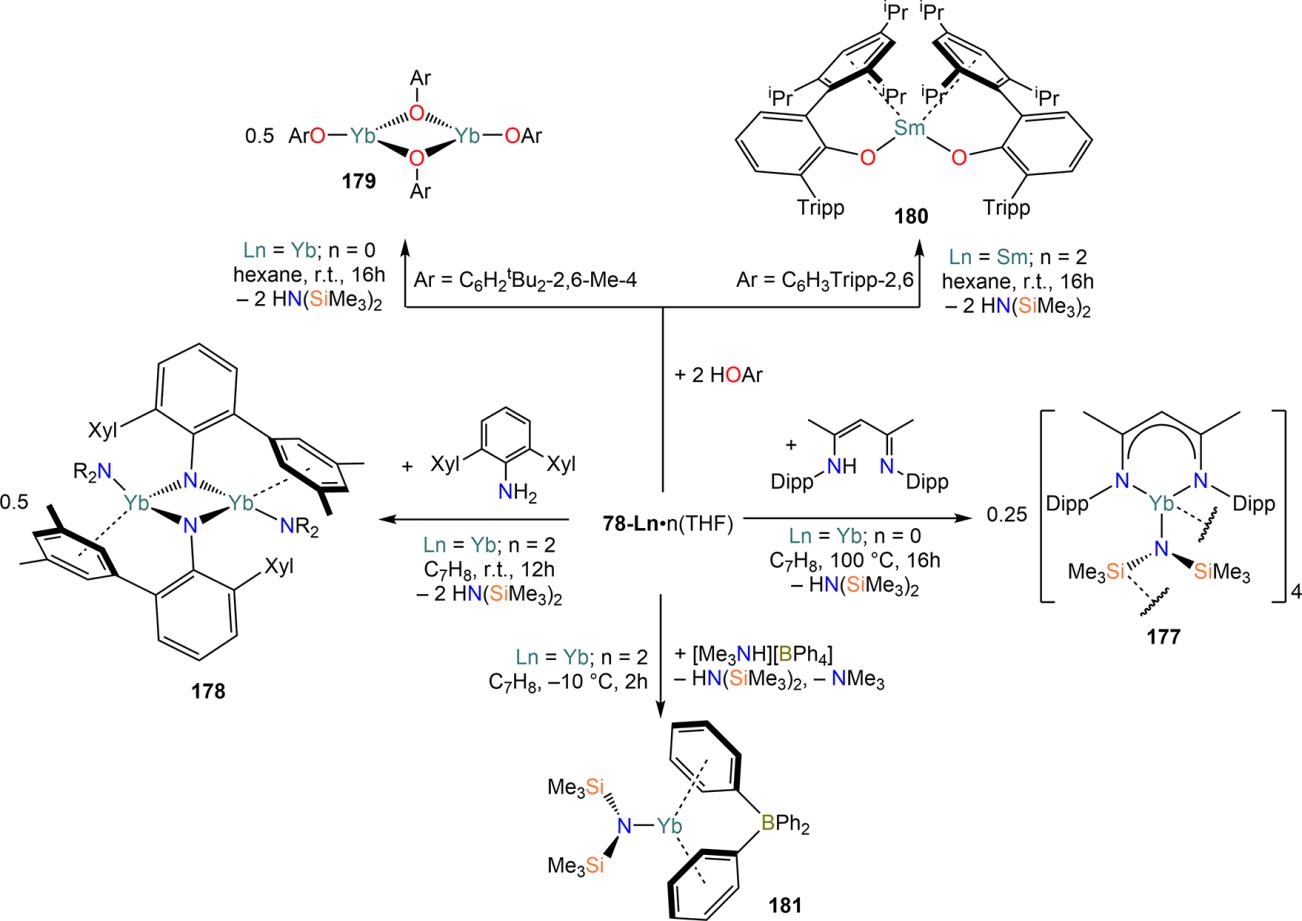
Selected Examples of Protonolysis Reactivity of **78-Ln** with Amides, Alcohols, and Ammonium Salts^191,508,718−510^

Amides **78-Ln** have been employed extensively as protonolysis reagents with various ligand systems, particularly multidentate amines and alcohols. **78-Yb** reacts with ^Me^BDI^Dipp^-H in toluene under reflux (Scheme 53) to give the heteroleptic tetrameric complex [{Yb(^Me^BDI^Dipp^){N(SiMe_3_)_2_}_2_}_4_] (**177**), which was further converted into the parent dimeric hydride [{Yb(^Me^BDI^Dipp^)(μ-H)}_2_] upon treatment with PhSiH_3_.^508^ Junk and Cole followed a similar approach with the synthesis of the sterically hindered bis-formamidinate complex [Sm(DippForm)_2_(THF)_2_] (**76**^**Dipp**^**-Sm**), which in addition was obtained *via* salt elimination and RTP methodologies (*vide supra*, Scheme 29).^191^ Moreover, Shi *et al.* treated the terphenyl-aniline H_2_N(C_6_H_3_Xyl-2,6) with **78-Yb**·2THF (Scheme 53), yielding the dimeric heteroleptic amide complex [{Yb{NH(C_6_H_3_Xyl-2,6)}{μ-N(SiMe_3_)_2_}] (**178**).^718^ Another very effective use of these silylamides is the synthesis of aryloxide complexes. This methodology can provide an excellent route toward low-coordinate complexes, especially because of the possibility of operating in the absence of ethereal solvents. Lappert and co-workers isolated the dimeric complex [{Yb(OAr)(μ-OAr)}_2_] (**179**; Ar = C_6_H_2_(^t^Bu)_2_-2,6-Me-4) from the reaction between **78-Yb** and two equivalents of HOC_6_H_2_(^t^Bu)_2_-2,6-Me-4 in hexane (Scheme 53).^509^ By using the highly sterically demanding ligand {OC_6_H_3_Tripp_2_-2,6}^−^, Zhao *et al.* stabilized the bis-aryloxide complex [Sm(OC_6_H_3_Tripp_2_-2,6)_2_] (**180**) from the reaction between the parent phenol, HOC_6_H_3_Tripp_2_-2,6, and **78-Sm**·2THF (Scheme 53).^510^ The ability of silylamides to act as Brønsted bases has also been exploited by Deacon and co-workers in the reaction of **78-Yb**·2THF with the ammonium salt [Me_3_NH][BPh_4_] (Scheme 53), which generates the *pseudo*-metallocene cationic complex [Yb{N(SiMe_3_)_2_}{(η^6^-Ph)_2_BPh_2_}(THF)_*n*_] (**181**; *n* = 0, 1).^511^**181** is itself an excellent synthetic precursor for protonolysis reactivity, as demonstrated by the same authors in the reaction with ^t^Bu_2_pzH and subsequent formation of the pyrazolate complex [Yb(^t^Bu_2_pz){(η^6^-Ph)_2_BPh_2_}(THF)].^511^

**Scheme 54:**
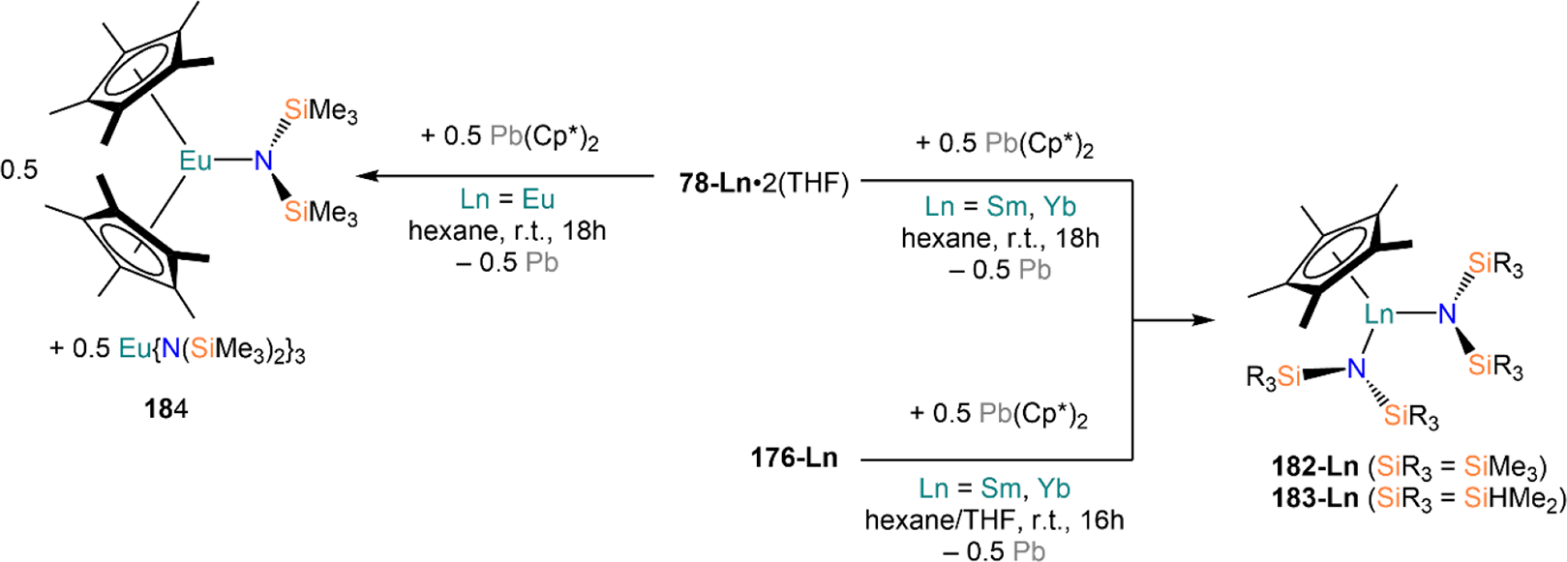
Redox Reactivity of **78-Ln** and **176-Ln** with Pb(Cp*)_2_^512^

Another interesting application of **78-Ln** was demonstrated by Anwander and co-workers with the synthesis of heteroleptic mono-Cp complexes using the lead reagent Pb(Cp*)_2_ (Scheme 54).^512^ In this methodology, **78-Ln**·2THF and **176-Ln** are reacted with 0.5 equiv of Pb(Cp*)_2_, causing oxidation of the Ln(II) center to Ln(III) and formation of piano-stool complexes [Ln(Cp*){N(SiMe_3_)_2_}_2_] (**182-Ln**; Ln = Sm, Yb) and [Ln(Cp*){N(SiHMe_2_)_2_}_2_(THF)] (**183-Ln**; Ln = Sm, Yb), with concomitant formation of metallic Pb.^512^ Conversely, when **78-Eu**·2THF is employed, the metallocene complex [Eu(Cp*)_2_{N(SiMe_3_)_2_}(THF)] (**184**) is isolated.

### 6.2 Trivalent RE Silylamides

**Scheme 55:**
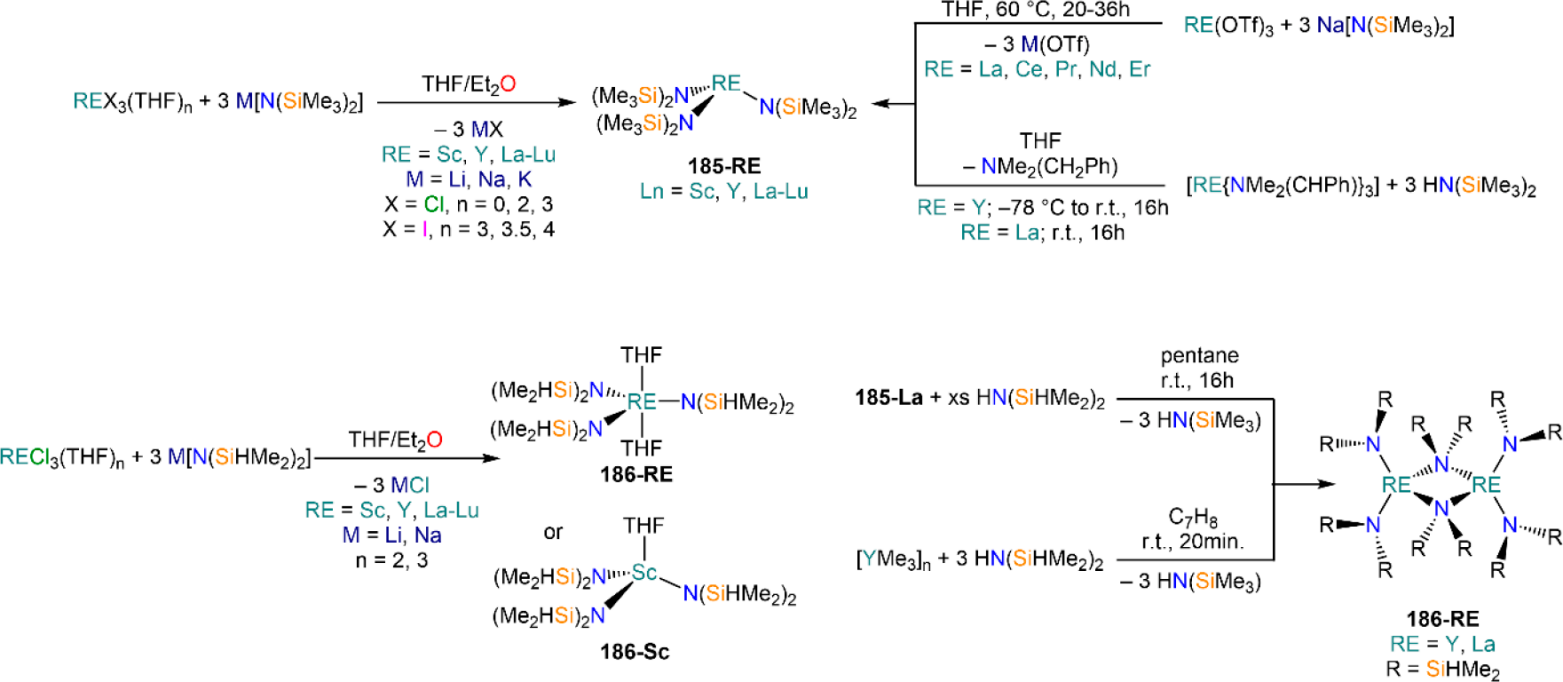
Synthesis of Trivalent Silylamides **185-RE** and **186-RE**(496−498,513−517,520)

RE(III) silylamides [RE{N(SiMe_3_)_2_}_3_] (**185-RE**) were originally synthesized by Bradley *et al.* in 1972 *via* salt elimination reactions between RE trichlorides and Li[N(SiMe_3_)_2_] in THF (Scheme 55 and Table 6).^496,497^ The methodology developed by Bradley is still widely used, but it can suffer from salt occlusion or “ate” complex formation. However, the use of heavier alkali metal salts and RE triiodides can suppress salt occlusion, and recrystallization or sublimation can be used for further purifications.^24,342^ Alternative procedures have also been used, such as (1) salt elimination reaction between RE(OTf)_3_ and Na[N(SiMe_3_)_2_]^513−515^ and (2) salt elimination reaction between benzyl-potassium reagents and RECl_3_ followed by protonolysis with HN(SiMe_3_)_2_ (Scheme 55).^513,516^ The dimethylsilyl analogues [RE{N(SiHMe_2_)_2_}_3_(THF)_*n*_] (**186-RE·***n*THF; RE = Sc, *n* = 1; RE = Y, La–Lu, *n* = 2) can be prepared following similar salt elimination methods using RE chlorides and group 1 transfer reagents M[N(SiHMe_2_)_2_] (Scheme 55), though reactions involving chloride salts and K[N(SiHMe_2_)_2_] lead to the isolation of products containing halide impurities.^498^**185-La** can also be used as a starting material for the preparation of solvent-free **186-La***via* protonolysis, similar to the strategy used for obtaining their divalent congeners **176-Ln** (Scheme 55).^502,517−519^ Moreover, Dietrich *et al.* obtained **186-Y** from the protonolysis reaction between [Y(Me)_3_]_*n*_ and HN(SiHMe_2_)_2_ (Scheme 55; *vide infra*, section 9.1).^520^ Finally, Anwander and co-workers attempted to prepare **186-RE** using RE(OTf)_3_ and M[N(SiHMe_2_)_2_] (M = Na, K); however, they could not obtain the desired products in pure form because of the contamination of M(OTf).^498^

**Scheme 56:**
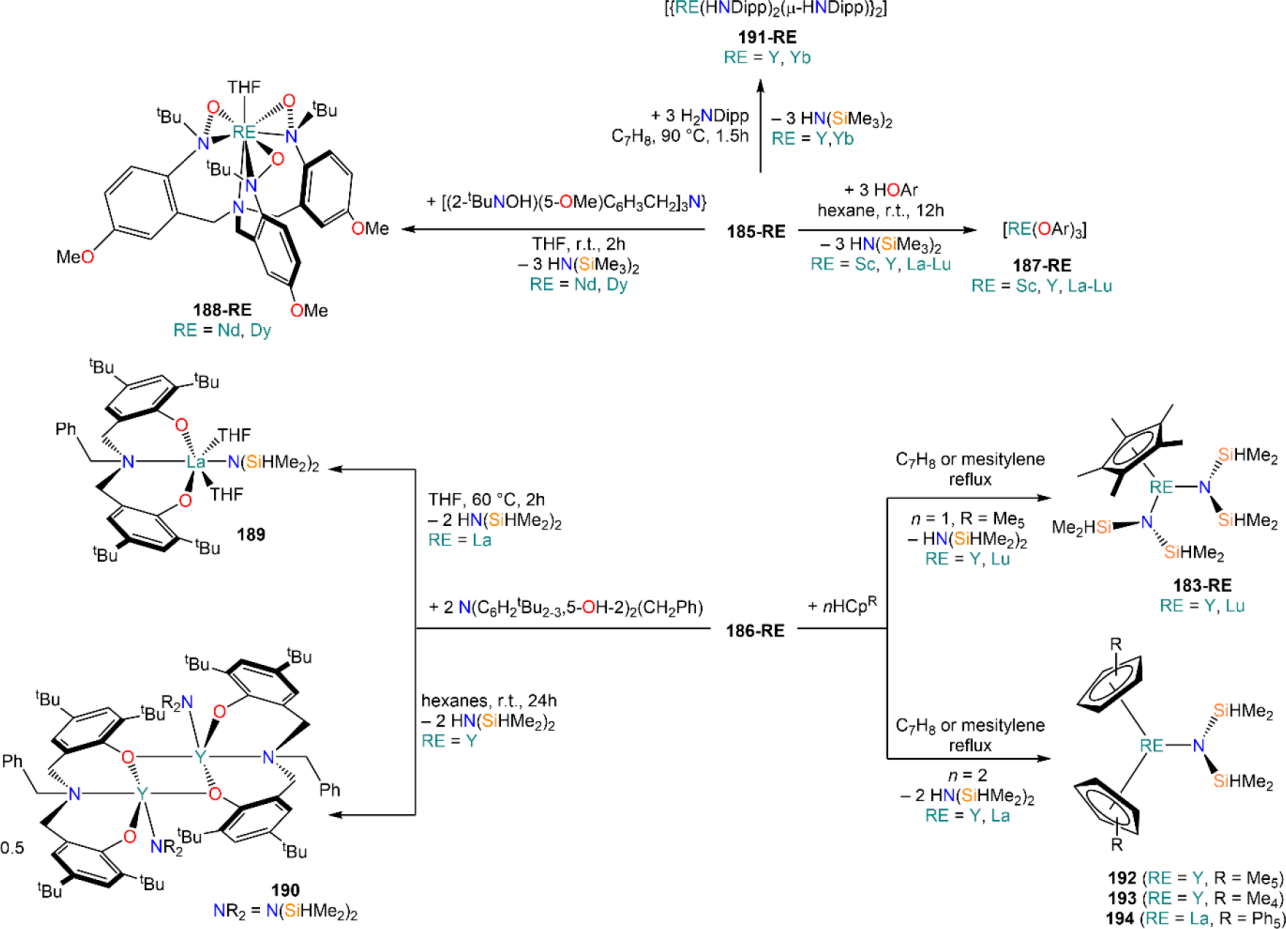
Selected Examples of Reactivity of **185-RE** and **186-RE**(521,525−529)

The applications of RE silylamides in protonolysis reactivity are numerous, and a full account is beyond the scope of this review.^22−24^ Some representative examples will be listed here to outline their main applications. Lappert and co-workers used **185-RE** (RE = Sc, Y, La Pr, Nd, Dy, Ho, Er, Yb) in protonolysis reactions with HODbmp, obtaining homoleptic aryloxo complexes [RE(ODbmp)_3_] (**187-RE**; RE = Sc, Y, La Pr, Nd, Dy, Ho, Er, Yb) (Scheme 56).^521^ This method has found many applications, particularly for obtaining RE aryloxides (especially whenever targeting solvent-free derivatives) and to exclude the possibility of halide and alkali metal contamination.^522−524^ Additionally, these protonolysis methodologies can be employed toward the synthesis of complexes supported by multidentate oxygen donors. Examples of this synthetic approach have been shown by Schelter and co-workers with the preparation of [RE(TriNOx^OMe^)(THF)] (**188-RE**; RE = Nd, Dy; TriNOx^OMe^ = {[(2-^t^BuNO)(5-OMe)C_6_H_3_CH_2_]_3_N}^3–^) from the deprotonation of parent hydroxylamine H_3_TriNOx^OMe^ with **185-Nd** and **185-Dy** (Scheme 56),^525^ or by Dong and Robinson with the use of **186-RE** (RE = Y, La) for the synthesis of the heteroleptic complexes [La{κ^3^*N*,*O*,*O*′-N(C_6_H_2_^t^Bu_2_-3,5-O-2)_2_(CH_2_Ph)}{N(SiMe_3_)_2_}(THF)_2_] (**189**) and [Y{μ:κ^3^*N*,*O*,*O*′-N(C_6_H_2_^t^Bu_2_-3,5-O-2)_2_(CH_2_Ph)}{N(SiMe_3_)_2_}]_2_ (**190**) (Scheme 56).^526^ Similarly, **185-RE** can also be used for the deprotonation of amines to target the isolation of solvent-free amides. Evans and co-workers reacted H_2_NDipp with **185-RE** (RE = Y, Yb), which led to the deprotonation of the aniline and formation of the target anilido complexes RE(HNDipp)_3_ (**191-RE**; RE = Y, Yb), though only **191-Y** was structurally authenticated, revealing a dimeric arrangement in the solid state (Scheme 56).^527^ Anwander *et al.* have also shown that **186-Y** and **185-La** are very effective deprotonating agents toward cyclopentadienes, obtaining a range of heteroleptic metallocene-silylamide complexes, *e.g.* [RE(Cp*){N(SiHMe_2_)_2_}_2_] (**183-RE;** RE = Y, Lu), [Y(Cp*)_2_{N(SiHMe_2_)_2_}] (**192**), [Y(Cp^tet^)_2_{N(SiHMe_2_)_2_}] (**193**; Cp^tet^ = {C_5_HMe_4_}^−^), and [La(Cp^Ph4^)_2_{N(SiHMe_2_)_2_}] (**194**) (Scheme 56).^528,529^ Interestingly, Teuben and co-workers could not obtain clean products when reacting **185-RE** (RE = Y, La, Ce) with HCp*, thus highlighting an advantage in using {N(HSiMe_2_)_2_}^−^ over {N(SiMe_3_)_2_}^−^ as a base for this type of reaction.^530^

**Scheme 57:**
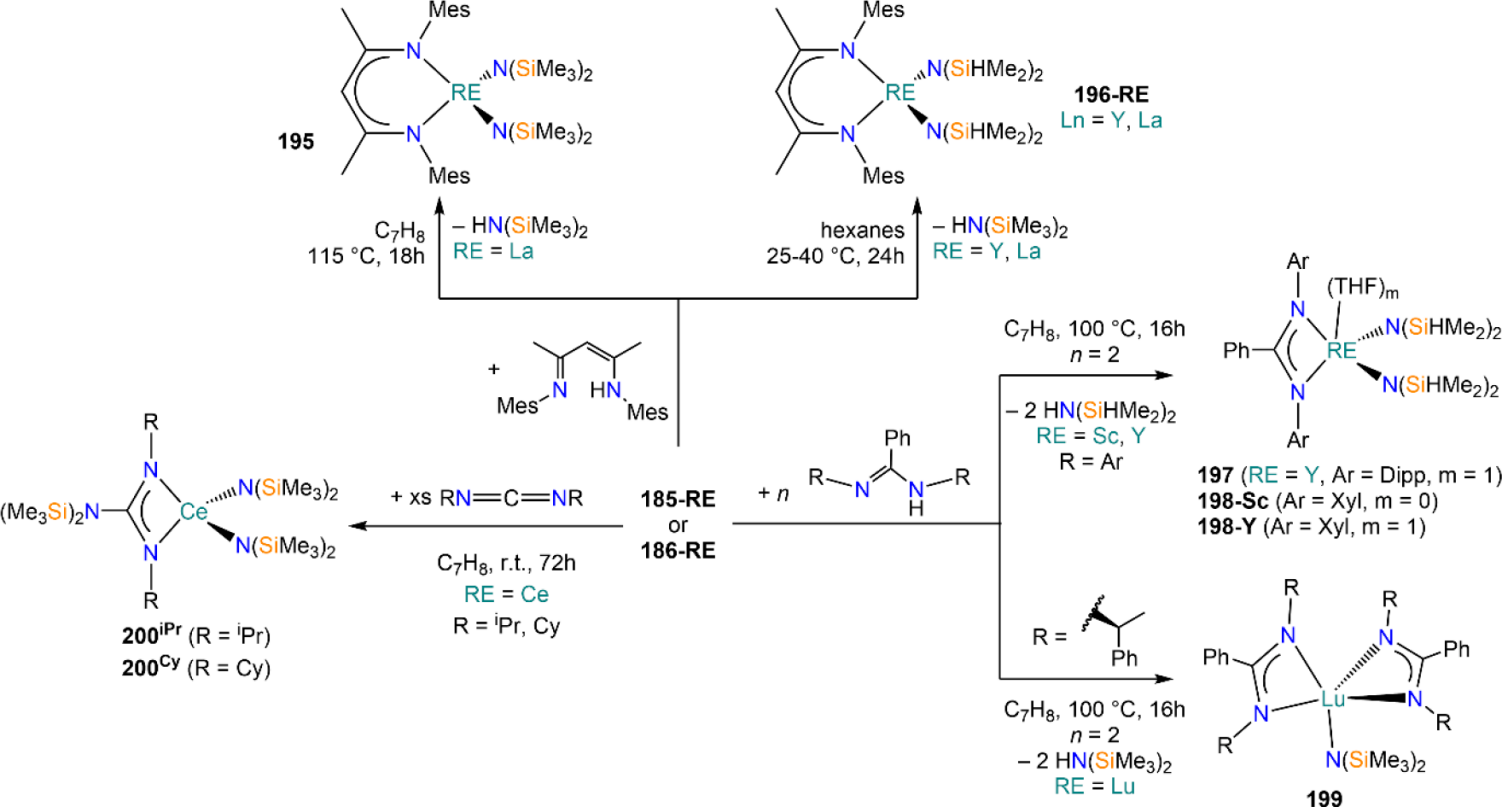
Deprotonation of Multidentate Amines with **185-RE** and **186-RE**(531−533) and Insertion Reactivity of **185-Ce** with Carbodiimides^524,534^

This strategy is also very effective for obtaining heteroleptic complexes supported by multidentate amides. Vitanova *et al.* reacted ^Me^BDI^Mes^-H with **185-La** and **186-RE** (RE = Y, La) and obtained the heteroleptic complexes [La(^Me^BDI^Mes^){N(SiMe_3_)_2_}_2_] (**195**) and [RE(^Me^BDI^Mes^){N(SiHMe_2_)_2_}_2_] (**196-RE**; RE = Y, La) (Scheme 57).^531^ A similar strategy was adopted by Luo and co-workers to synthesize the heteroleptic species [Y{(NDipp)_2_CPh}{N(SiHMe_2_)_2_}_2_(THF)] (**197**) and [RE{(NXyl)_2_CPh}{N(SiHMe_2_)_2_}_2_(THF)_*n*_] (**198-RE**; RE = Sc, Y; *n* = 0, 1) (Scheme 57).^532^ Moreover, Roesky and co-workers deprotonated the chiral amidine (*S*,*S*)-*N*,*N*-bis(1-phenylethyl)benzamidine, (*S*)-HPEBA-H, with **185-Lu**, affording the bis-amidinate complex [Lu{(*S*)-PEBA}_2_{N(SiMe_3_)_2_}] (**199**, Scheme 57).^533^ Another approach toward the stabilization of heteroleptic silylamide complexes was demonstrated by Schelter and co-workers with the preparation of heteroleptic guanidinate complexes [Ce{(NR)_2_CN(SiMe_3_)_2_}{N(SiMe_3_)_2_}] (**200**^**R**^; R = ^i^Pr, Cy); in their study, they reacted **185-RE** with carbodiimides R–N=C=N–R (R = ^i^Pr, Cy) leading to insertion into one of the C–N bonds (Scheme 57).^524,534^

### 6.3 Tetravalent Ce Amides

**Scheme 58:**
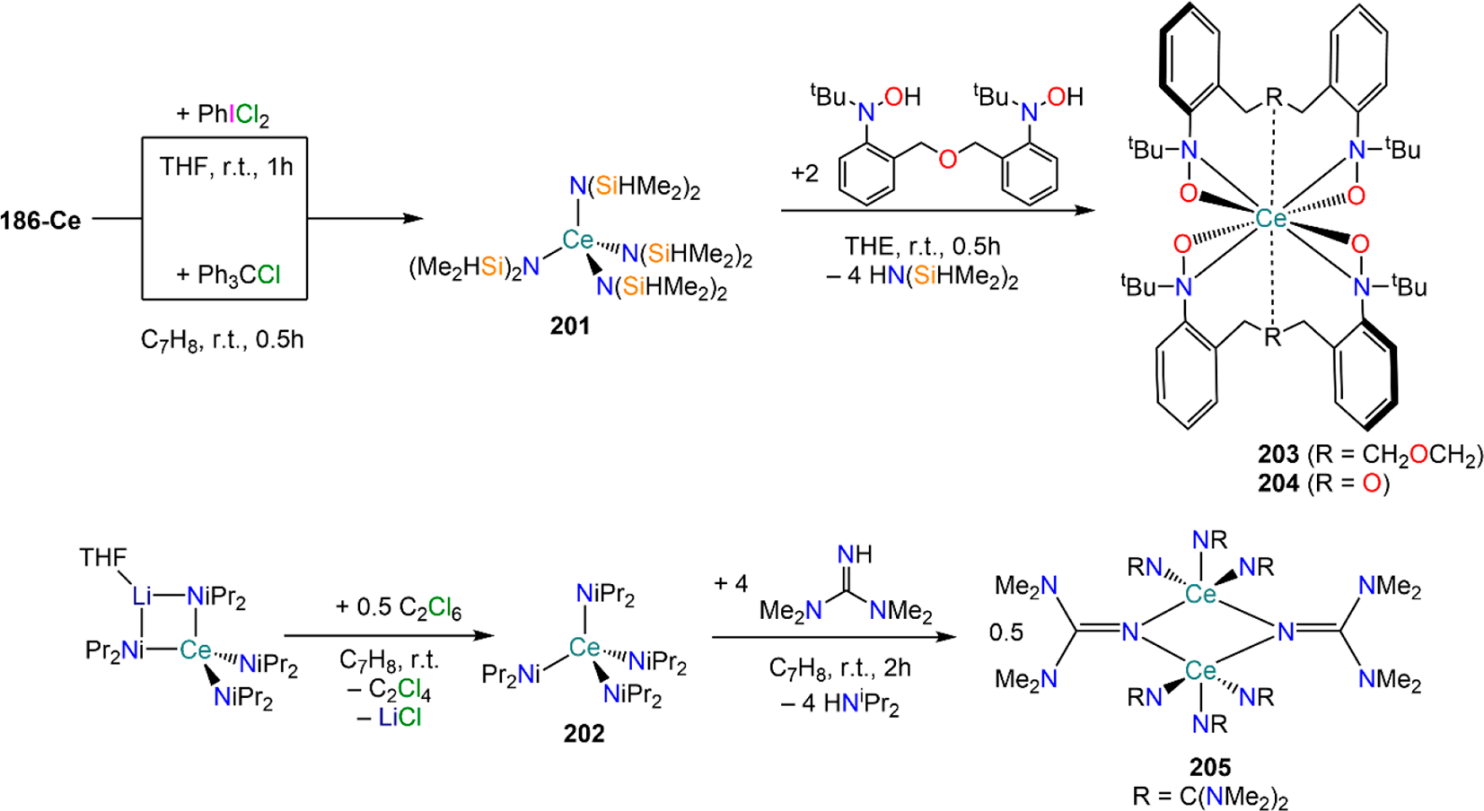
Synthesis of Tetravalent Ce(IV) Amides **201** and **202** and Protonolysis Reactivity^535−537^

Amide ligands can function as supporting ligands for Ce(IV) complexes, and some of these species are now routinely used as Ce(IV) starting materials in protonolysis reactions. Two of the most common Ce(IV) amide starting materials are complexes [Ce{N(SiHMe_2_)_2_}_4_] (**201**)^535^ and [Ce(N^iPr4^)_4_] (**202**).^536^ Both of these species are obtained from oxidation of Ce(III) precursors, **186-Ce** and [Ce(N^iPr4^)_4_Li], respectively, using PhICl_2_, Ph_3_CCl, or C_2_Cl_6_ (Scheme 58). Both **201** and **202** can react with protic substrates to generate new Ce(IV) complexes (Scheme 58), as shown by Kim *et al.* with the synthesis of the 8-coordinate complexes [Ce{Ph[C_6_H_4_N(^t^Bu)(O)-2]_2_}_2_] (**203**)^537^ and [Ce{O[CH_2_–C_6_H_4_N(^t^Bu)(O)-2]_2_}_2_] (**204**)^538^ and by Schneider *et al.* with the stabilization of the tetramethylguanidinate (TMG) complex [{Ce(TMG)_3_(μ-TMG)}_2_] (**205**) (Scheme 58).^536^

## 7 Oxygen Donors

The RE metals are very electropositive and highly oxophilic and therefore form very stable complexes with *O*-based donors.^26,28,44,559^ The coordination chemistry of aliphatic alkoxides and aryloxides with the REs is extremely well-developed, and throughout this review various methodologies which are employed for the synthesis of Ln(OR)_2_ and RE(OR)_3_ derivatives are discussed (see section 3). Alkoxides have attracted a lot of interest because of their high volatility, which makes them very desirable precursors for the fabrication of new materials.^29^ In principle, these derivatives can be used as starting materials for protonolysis reactivity by matching them with substrates with higher p*K*_a_ (*vide supra*, Table 2); however, their applications are limited compared to other protonolysis reagents illustrated in this review (*e.g.*, amides and alkyls). Nonetheless, aryloxide RE complexes have found applications as protonolysis or metathesis reagents for the synthesis of challenging coordination complexes and organometallic derivatives, and Ce(IV) alkoxides have also been used in protonolysis and metathesis reactions.^560−562^ Additionally, RE inorganic derivatives containing oxygen donors have also found several applications in synthesis, particularly triflates and nitrates. For the purpose of this review we will focus on the following reagents: (1) aryloxides (section 7.1) and (2) Ce(IV) alkoxides and CAN (section 7.2). Triflates are discussed in a separate section (section 8).

### 7.1 Aryloxides

**Scheme 59:**
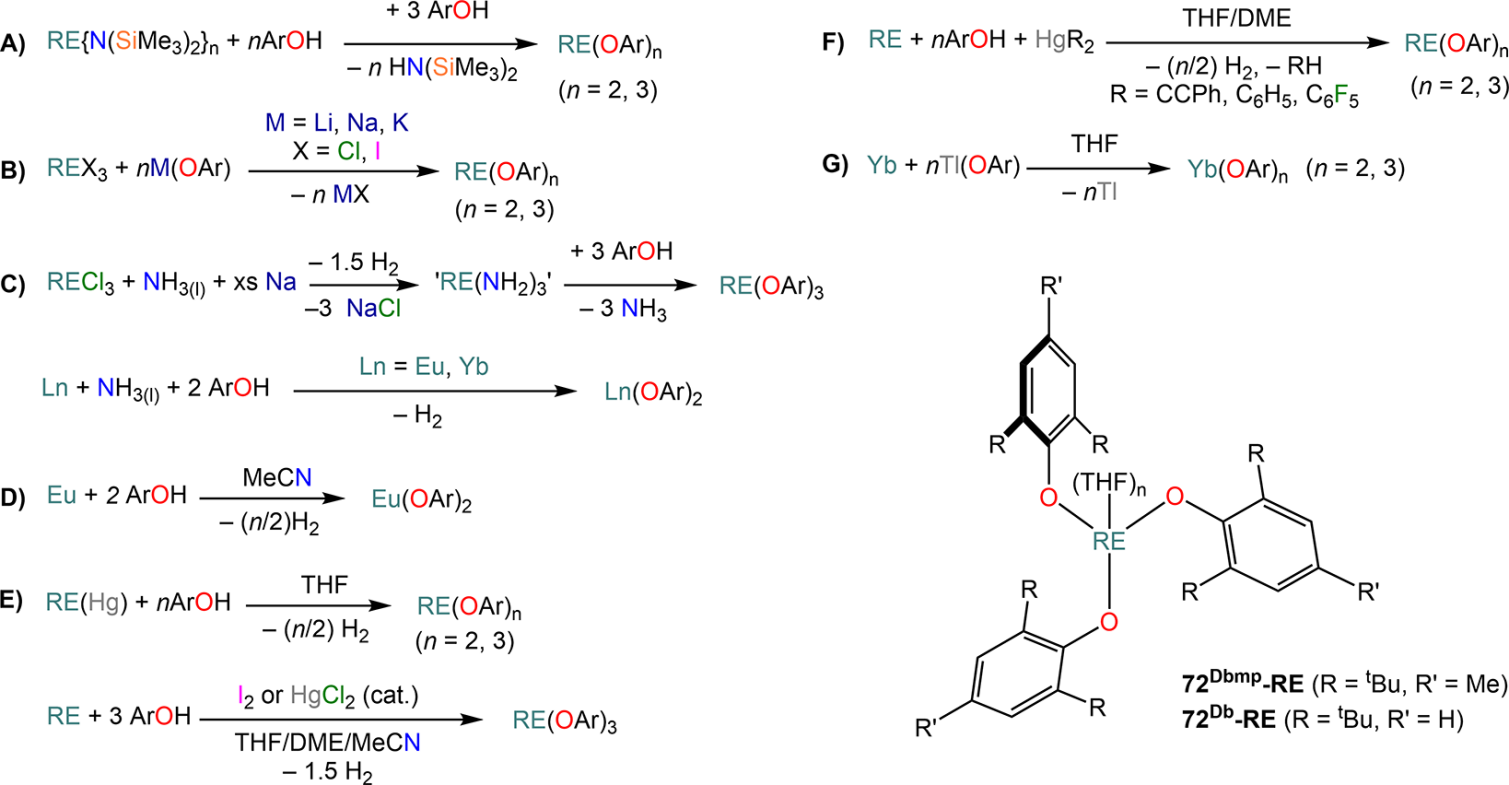
Synthetic Strategies for the Synthesis of Divalent and Trivalent RE Aryloxides

The synthesis of RE aryloxides [RE(ODbmp)_3_] (**72**^**Dbmp**^**-RE**) was first reported by Lappert and co-workers in 1983, based on protonolysis of substituted phenol HODbmp with tris-silyalamide precursors **185-RE** (**A**, Scheme 59).^521^ This method is still widely applied to RE aryloxide synthesis and has been used also for the synthesis of very sterically congested systems which cannot be obtained using other synthetic strategies, *e.g.* [Y(OC_6_H_2_Ad_2_-2,6-^t^Bu-4)_3_].^563^ Alcoholysis with Ln(II) silylamides can also be used to obtain divalent Ln alkoxides, and by using solvent-free **78-Yb** it was possible to obtain the homoleptic aryloxide **179** (**A**, Scheme 59; *vide supra*, Scheme 53).^505,509^ Other approaches, some of which are illustrated in detail in other sections of this review, include (1) salt elimination between REX_3_ and MOAr (**B**, Scheme 59);^270,522,564^ (2) ammoniacal synthesis (**C**, Scheme 59; *vide supra*, Scheme 9);^90,92,565^ (3) direct reaction with metals (**D**, Scheme 59; *vide supra*, Scheme 14);^120^ (4) direct reaction with metals in the presence of activators, *e.g.* Hg, HgCl_2_, I_2_ (**E**, Scheme 59; *vide supra*, Schemes 17 and 23);^134,135,150,153,566^ (5) RTP with organomercurials and HOAr (**F**, Scheme 59; *vide supra*, Scheme 27);^112,123,135,153,183,198,199^ and (6) RT with Tl(OAr) reagents (**G**, Scheme 59; *vide supra*, Scheme 31).^91,170,198^

**Scheme 60:**
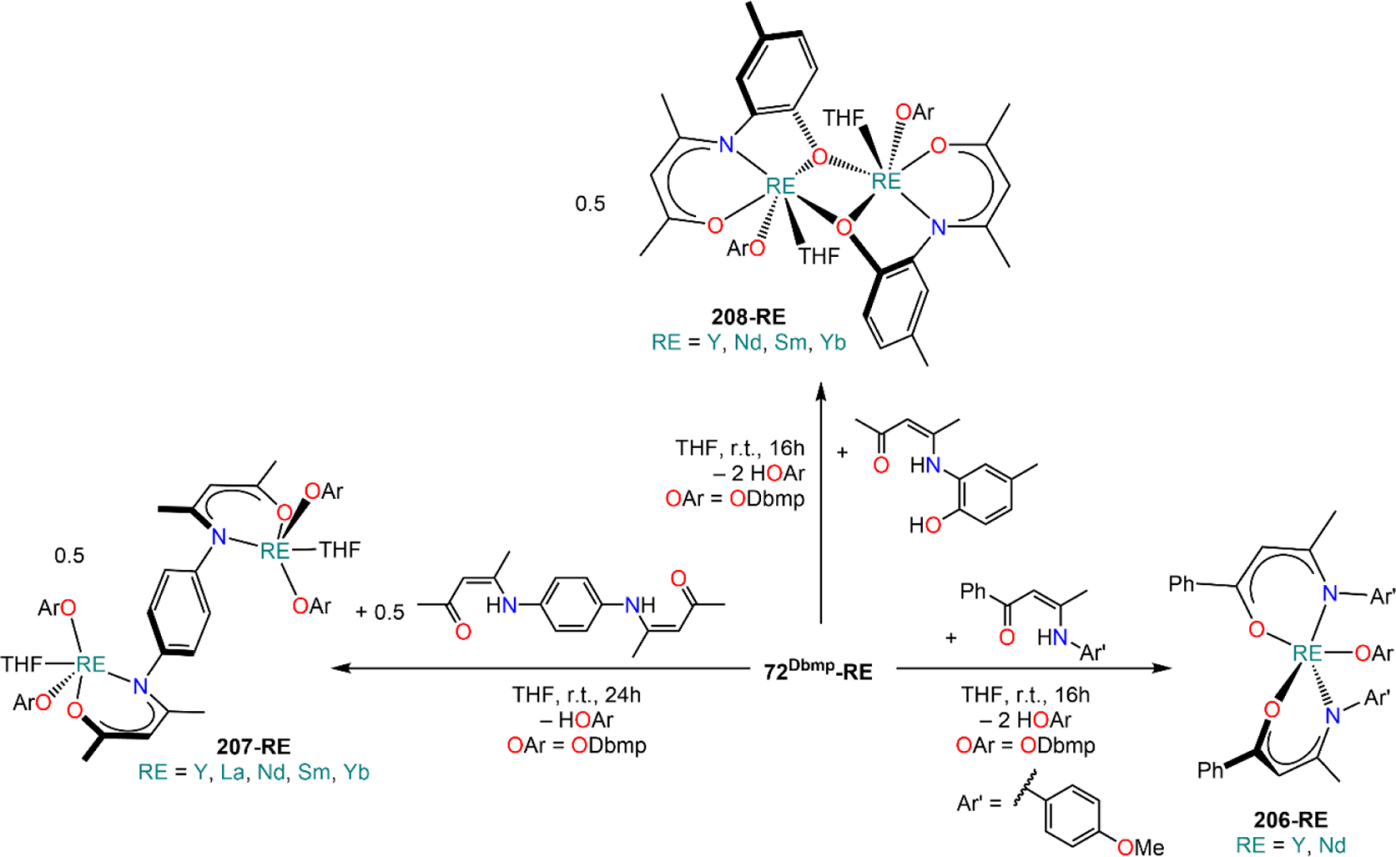
Application of **72**^**Dbmp**^**-RE** as Precursor in Protonolysis Reactions with Various β-Ketoimines^567−569^

RE aryloxides (Table 7) have found extensive usage in protonolysis reactions with various substrates, particularly with multidentate donors containing aryloxide functionalities.^567−570^ An example of this is the alcoholysis reaction of β-ketoimines (Scheme 60) with **72**^**Dbmp**^**-RE** (RE = Y, La, Nd, Sm, Yb).^567−569^ β-Ketoimine 1-phenyl-3-N-(*p*-methoxyphenylimino)-1-butanone reacts smoothly in a 2:1 fashion with **72**^**Dbmp**^**-RE** (RE = Y, Nd) in THF at room temperature to give heteroleptic complexes of formula [RE{N(C_6_H_4_OMe-3)C(Me)CHC(Ph)O}_2_(ODbmp)] (**206-RE**; RE = Y, Nd).^567^ In a similar fashion, Shen and co-workers were able to obtain a series of bimetallic RE complexes supported by a phenyl-bridged BDI ligand (**207-RE**; RE = Y, La, Nd, Sm, Yb)^568^ and a tridentate β-ketoiminate containing an additional aryloxo functionality (**208-RE**; RE = Y, Nd, Sm, Yb).^569^

**Table 7:** Selected Divalent and Trivalent RE Aryloxides and Ce(IV) Alkoxides Used as Synthetic Precursors^a^

	Ln(OAr)_2_	RE(OAr)_3_	Ce(OR)_4_
Sc		[Sc(ODbmp)_3_]^521^	
Y		[Y(ODbmp)_3_]^521,585^	
La		[La(ODbmp)_3_]^521,586^	
		[La(ODb)_3_(MeCN)]^153^	
Ce		[Ce(ODbmp)_3_]^521,585^	Ce(OMe)_4_^576,577,579^
		[Ce(ODb)_3_]^587,588^	Ce(OEt)_4_^576,577^
		[Ce(ODb)_3_(MeCN)]^587^	Ce(O^i^Pr)_4_^576,577,579,580^
			[{Ce(O^t^Bu)_3_(μ-O^t^Bu)}_2_]^589^
			[Ce(O^t^Bu)_4_(py)_2_]^583,590^
			Ce(O^t^Bu)_4_^581^
			Ce(O^t^Bu)_3_(NO_3_)^581^
			Ce(O^t^Bu)_2_(NO_3_)_2_^581^
			Ce(O^t^Bu)(NO_3_)_3_^581^
			[{Ce(OCH_2_^t^Bu)_4_}_3_]^582^
Pr		*Pr(ODbmp)_3_*(521)	
		[Pr(ODb)_3_]^591^	
Nd		*Nd(ODbmp)_3_*(521)	
		[Nd(ODb)_3_]^591^	
Sm	[Sm(ODbmp)_2_(THF)_3_]^592−595^	[Sm(ODbmp)_3_(THF)]^596^	
		[Sm(ODbmp)_3_(MeCN)_2_]^153^	
		[Sm(ODb)_3_]^588^	
Eu	[Eu(ODbmp)_2_(THF)_3_]^153,270^		
	[Eu(ODb)_2_(THF)_2_]^597^		
Gd		[La(ODbmp)_3_]^521,586^	
Tb		[Tb(ODipp)_3_(THF)_2_]^566^	
Dy		Dy(ODbmp)_3_^521^	
		[Dy(ODb)_3_]^499^	
		[Dy(ODb)_3_(THF)]^499^	
		[Dy(ODb)_3_(py)]^499^	
		[Dy(ODb)_3_(NH_3_)]^499^	
Ho		Ho(ODbmp)_3_^521^	
Er		[Er(ODbmp)_3_]^598^	
		[Er(Dbmp)_3_(THF)]^564^	
		[Er(ODb)_3_]^588^	
		[Er(ODb)_3_(py)_2_]	
Tm		[Tb(OC_6_H_3_^t^Bu_3_-2,4,6)]^599^	
Yb	[{Yb(ODbmp)(μ-ODbmp)}_2_]^505,509^	[Yb(ODbmp)_3_(THF)]^170^	
	[Yb(ODbmp)_2_(THF)_2_]^91^	[Yb(ODbmp)_3_(MeCN)]^602^	
	[Yb(ODbmp)_2_(THF)_3_]^600^	[Yb(ODb)_3_]^588^	
	[Yb(ODbmp)_2_(OEt_2_)_2_]^91^		
	*Yb(ODb)*_*2*_*(THF)*_*2*_(601)		
Lu		Lu(ODbmp)_3_^521^	
		[Lu(ODb)_3_]^588^	

a Compounds in italics have not been structurally authenticated.

**Scheme 61:**
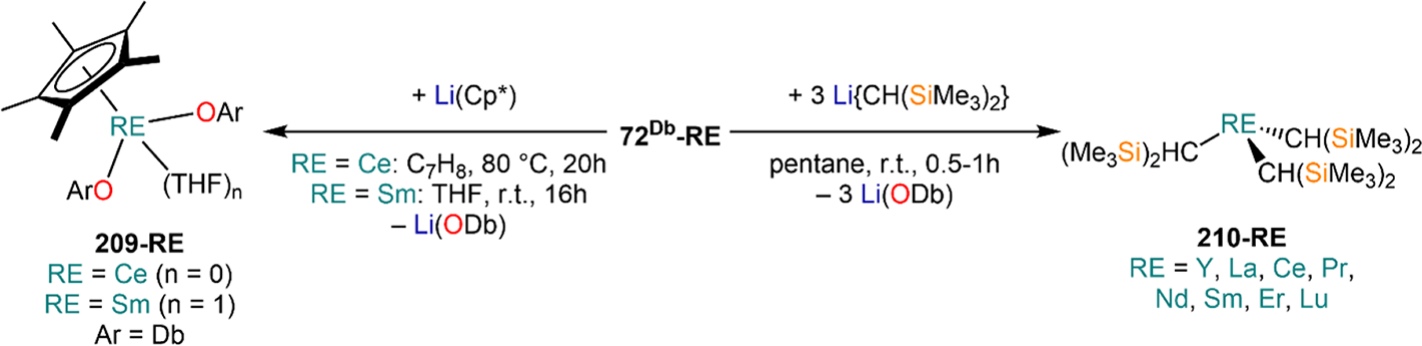
Use of **72**^**Db**^**-RE** in Metathesis Reactions with Organolithium Reagents^571−574^

A particularly important application of **72**^**Dbmp**^**-RE** is their use as synthetic precursors for the preparation of new RE organometallic complexes. In this type of methodology, the aryloxide donor is reacted with an organolithium reagent, resulting in transmetalation and formation of insoluble Li(OAr) as a byproduct. This strategy was applied successfully to the synthesis of heteroleptic aryloxo-Cp RE complexes by Teuben and Watkin, who reacted **72**^**Db**^**-RE** (RE = Ce, Sm) with one equivalent of Li(Cp*) to give the monoring complex [RE(Cp*)(ODb)_2_(THF)_*n*_] (**209-RE**; RE = Ce, *n* = 0; RE = Sm, *n* = 1) (Scheme 61).^571,572^ Additionally, several σ-bonded alkyl complexes have been obtained with this methodology, using alkyl ligands stabilized by silyl substituents on the β-position. Lappert originally reported the reaction of **72**^**Db**^**-La** and **72**^**Db**^**-Sm** with Li{CH(SiMe_3_)_2_}, which gave homoleptic tris-alkyls [RE{CH(SiMe_3_)_2_}_3_] (**210-RE**; RE = La, Sm).^51^ This method was then extended to the synthesis of mid- and late-Lns and is now the main methodology used for the preparation of this class of compounds (Scheme 61).^573,574^

### 7.2 Ce(IV) Alkoxides and CAN

**Scheme 62:**
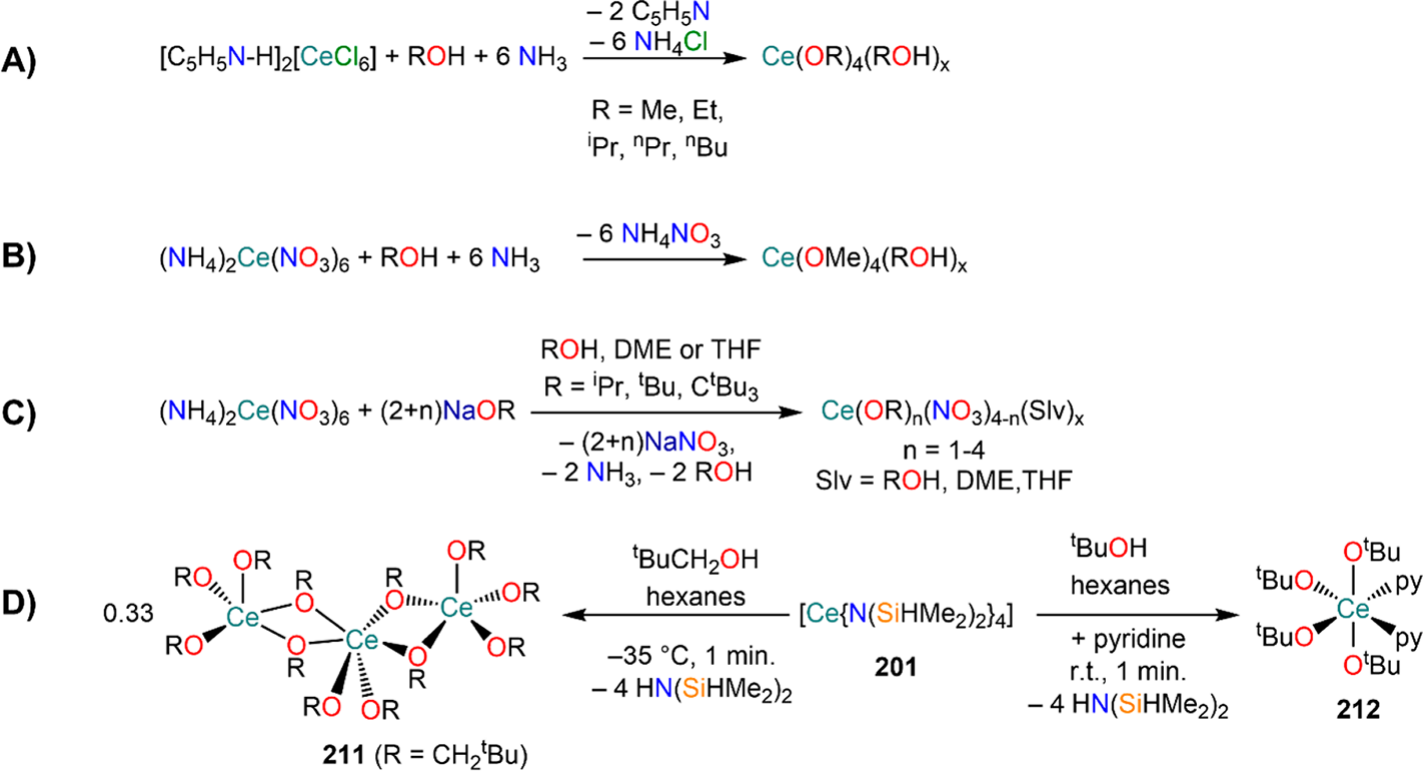
Preparation of Ce(IV) Alkoxides *via* the “Ammonia Method”^576,577^ and “CAN Method”^579−581^ and Synthesis of **211** and **212***via* Alcoholysis with Silylamide Complex **201**(583)

The coordination chemistry of tetravalent cerium has attracted a lot interest, especially because of the use of CAN as a selective oxidizing agent in organic transformations.^575^ However, advances in the coordination chemistry of Ce(IV) have been hampered by the lack of well-defined and hydrocarbon-soluble starting materials, especially compared to the ample variety of compounds available for trivalent REs. A strong electron-donating environment is usually required to stabilize Ce^4+^, and for this reason there has been a lot of progress in developing Ce synthons containing oxygen donors.^559^ Bradley and co-workers reported the preparation of Ce(OR)_4_ (Et, ^i^Pr, ^n^Pr, ^n^Bu) compounds by passing ammonia in an alcoholic solution of dipyridinium Ce hexachloride, [C_5_H_5_N–H]_2_[CeCl_6_] (“ammonia method”, Scheme 62, **A**).^576−578^ Gardeff and co-workers showed that CAN can also be used as a starting material in a very similar methodology for the preparation of Ce(OMe)_4_ (“modified ammonia method”, Scheme 62, **B**).^579^ Alternatively, CAN is dissolved in an alcohol of choice and then reacted with NaOMe, thus allowing for an easier control of reaction conditions and stoichiometry (“CAN method”, Scheme 62, **C**); the authors also noted that Ce(O^i^Pr)_4_ obtained with the original “ammonia method” is more difficult to purify, in comparison with the “CAN method” which allows for quick reaction times and easier purification procedures. Additionally, the reactions can be carried out in ethereal solvents, such as THF or DME.^579,580^ Evans *et al.* used Gardeff’s method to obtain Ce(O^t^Bu)_4_, together with other mixed nitrate-alkoxide Ce(IV) salts, *e.g.* Ce(O^t^Bu)_*n*_ (NO_3_)_4–*n*_ (*n* = 1–4).^581^ More recently Anwander and co-workers have used the CAN method to obtain Ce(OCH_2_^t^Bu)_4_, which can also be synthesized from alcoholysis with silylamide **201** and crystallizes as the trimer [{Ce(OCH_2_^t^Bu)_4_}_3_] (**211**).^582^ Finally, Schelter and Anwander converted Ce(IV) silylamide **201** into [Ce(O^t^Bu)_4_(py)_2_] (**212**) *via* fast alcoholysis with ^t^BuOH (Scheme 62, **D**).^583^

**Scheme 63:**
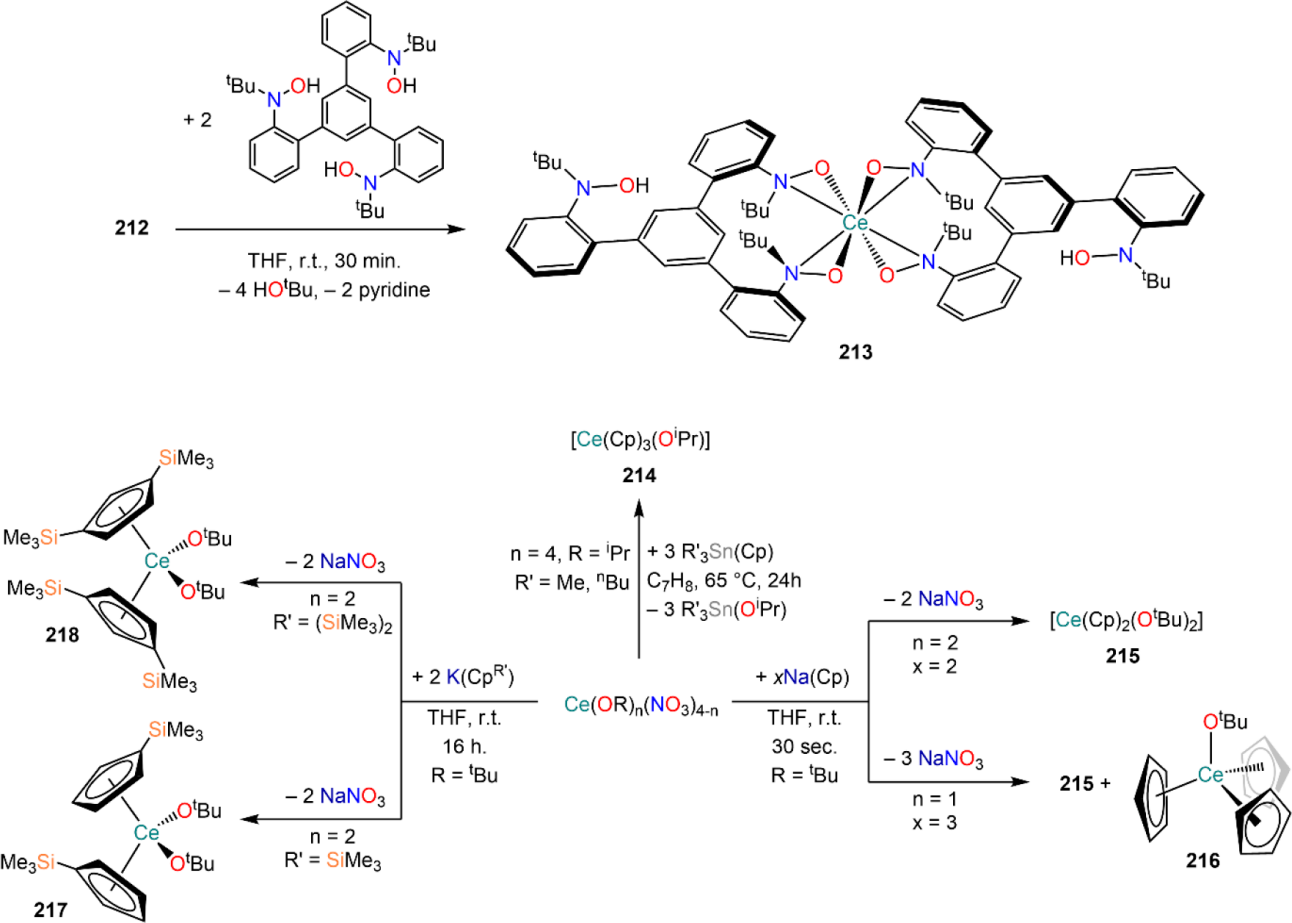
Reactivity of Ce(IV) Alkoxides with Protic Substrates and Cp Reagents^560−562^

Ce(IV) alkoxides (Table 7) have been applied as protonolysis reagents for the synthesis of Ce(IV) complexes, as demonstrated by Kim *et al.* with the synthesis of 8-coordinate complex [Ce(Harene-TriNOx)_2_] (**213**, HareneTriNOx = {[(2-^t^BuNO)C_6_H_4_]_2_[(2-^t^BuNOH)C_6_H_4_]C_6_H_3_}^2–^) (Scheme 63).^560^ Gulino *et al.* attempted to use Ce(O^i^Pr)_4_ in salt elimination reactions with Tl(Cp) or Mg(Cp)_2_ without success, observing reduction to Ce(III) instead.^561^ Nonetheless, when the tin reagent R_3_SnCp (R = Me, ^n^Bu) was employed, they were able to isolate Ce(Cp)_3_(O^i^Pr) (**214**), though structural authentication was not obtained (Scheme 63).^561^ A different approach was followed by Evans *et al.*, who reacted mixed Ce(IV) nitrate-alkoxides Ce(O^t^Bu)_2_(NO_3_)_2_ and Ce(O^t^Bu)(NO_3_)_3_ with Na(Cp). In the case of the reaction between Ce(O^t^Bu)_2_(NO_3_)_2_ and two equivalents of Na(Cp), the metallocene Ce(IV) complex Cp(Cp)_2_(O^t^Bu)_2_ (**215)** was obtained in excellent yields but could not be structurally authenticated (Scheme 63).^562^ However, when Ce(O^t^Bu)(NO_3_)_3_ was reacted with three equivalents of Na(Cp), a mixture containing **215** and [Cp(Cp)_3_(O^t^Bu)] (**216**) was obtained, and the latter was structurally characterized (Scheme 63).^562^ Finally, Gordon and co-workers used the same methodology to isolate metallocene derivatives [Ce(Cp′)_2_(O^t^Bu)_2_] (**217**) and [Ce(Cp″)_2_(O^t^Bu)_2_] (**218**) (Scheme 63).^584^

## 8 Triflates

**Scheme 64:**
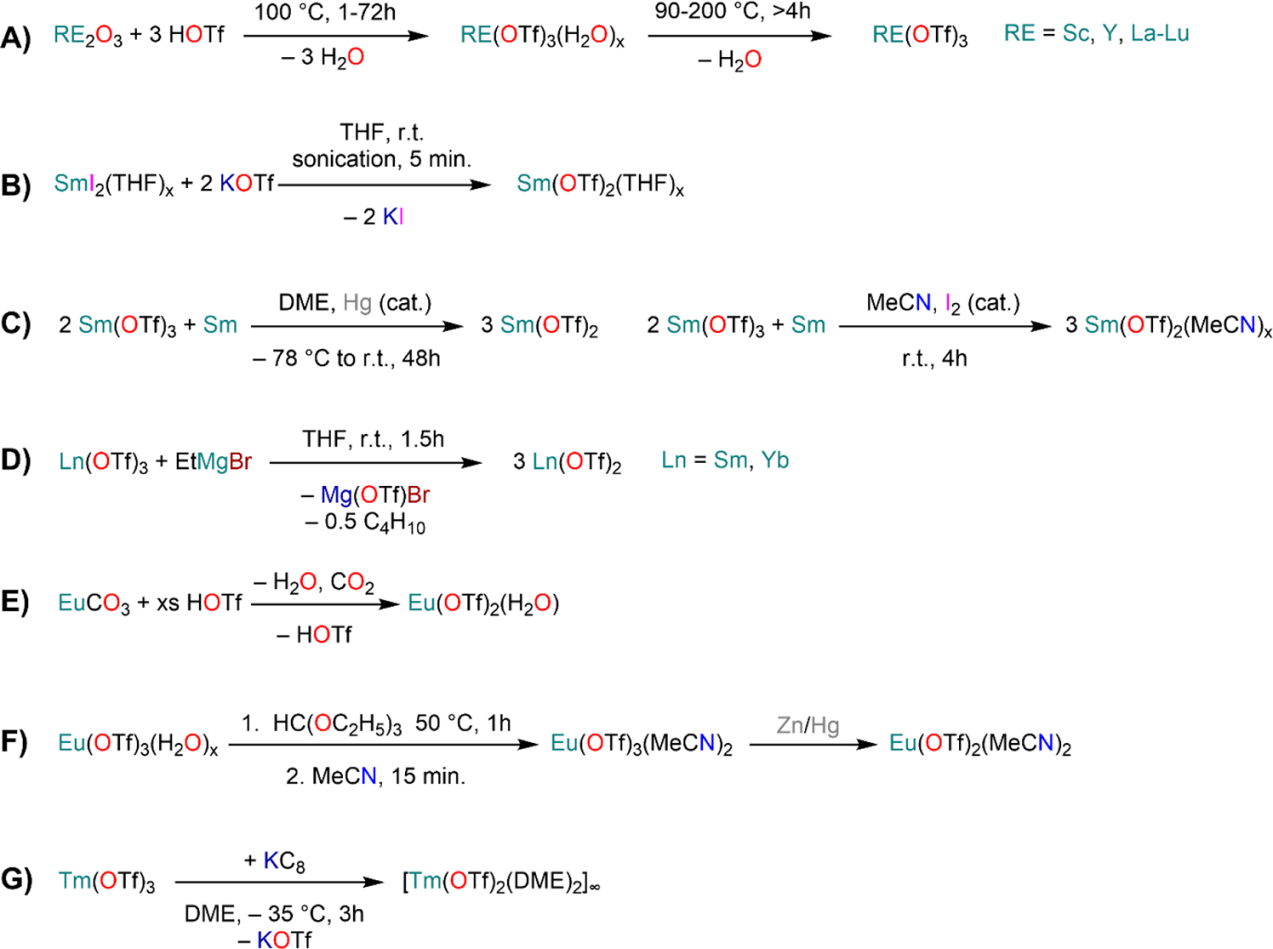
Preparation of Divalent and Trivalent RE Triflate Salts

Triflate (OTf, {CF_3_SO_3_}^−^) salts are a very popular choice of starting materials in RE synthetic chemistry (Table 8). These salts are used as more soluble replacements of halides in salt elimination reactions.^9,31^ Additionally, triflates are better leaving groups and can be easily displaced from the metal coordination sphere.^9,31^ Similar to RECl_3_, RE(OTf)_3_ salts are usually commercially available both in anhydrous and hydrate form. However, they can also be conveniently made from the direct reaction of the oxide (RE_2_O_3_) with triflic acid (HOTf), followed by thorough desiccation under reduced pressure; reports vary from a minimum of 4 h to several days (Scheme 64, **A**).^9,603−606^ This method was originally reported by Massaux and is the most common strategy for obtaining anhydrous RE(OTf)_3_ salts.^605^ Triflate salts of divalent Sm, Eu, and Yb are obtained *via* various methodologies. Sm(OTf)_2_ can be prepared from direct salt elimination reaction of K(OTf) with SmI_2_ (Scheme 64, **B**)^607^ or by reducing Sm(OTf)_3_ with Sm metal in the presence of iodine^608^ or mercury^609^ (Scheme 64, **C**). Nief and co-workers obtained poor results when performing this reaction in THF, whereas they achieved excellent conversions when using DME, ultimately leading to the isolation of the DME adduct Sm(OTf)_2_(DME)_2_.^609^ Another method for the preparation of Ln(OTf)_2_ is the reduction of Ln(OTf)_3_ with Grignard reagents, which has also been successfully employed for the preparation of Yb(OTf)_2_(THF)_3_ (Scheme 64, **D**).^610^ The DME-solvate analogue, Yb(OTf)_2_(DME), has also been obtained from the reaction of HOTf with [Yb{N(SiMe_3_)_2_}_2_(OEt_2_)_2_].^611^

**Table 8:** RE Triflate Salts Used in Anaerobic Synthesis^a^

	Ln(OTf)_2_	RE(OTf)_3_	RE(OTf)_4_
Sc		Sc(OTf)_3_^603^	
Y		Y(OTf)_3_^603,605,616^	
La		La(OTf)_3_^605,616,627^	
Ce		Ce(OTf)_3_^414^	Ce(OTf)_4_^614,615^
Pr		Pr(OTf)_3_^604^	
Nd		Nd(OTf)_3_^605,616^	
Sm	*Sm(OTf)*_*2*_(610)	Sm(OTf)_3_^605,628^	
	Sm(OTf)_2_(DME)_2_^609^		
Eu	Eu(OTf)_2_(H_2_O)^606^	Eu(OTf)_3_^605,621^	
	Eu(OTf)_2_(MeCN)_2_^612^		
Gd		Gd(OTf)_3_^616^	
Tb		Tb(OTf)_3_^629^	
Dy		Dy(OTf)_3_^630^	
Ho		Ho(OTf)_3_^630^	
Er		Er(OTf)_3_^605,616^	
Tm	[Tm(OTf)_2_(DME)_2_]_∞_^613^	Tm(OTf)_3_^629^	
Yb	*Yb(OTf)*_*2*_(610,611)	Yb(OTf)_3_^605^	
	Yb (OTf)_2_(THF)_2_^611^		
Lu		Lu(OTf)_3_^604^	

a Compounds in italics have not been isolated.

Eu(OTf)_2_ can be obtained by treating EuCO_3_ with excess triflic acid, affording monohydrate Eu(OTf)_2_(H_2_O) upon drying *in vacuo* at 90 °C overnight (Scheme 64, **E**).^606^ Alternatively, water-free Eu(OTf)_2_ can be obtained with the method described by Krossing and co-workers, which starts from hydrated Eu(OTf)_3_(H_2_O)_*x*_.^612^ Eu(III) triflate is dissolved in triethylorthoformiate and heated at 50 °C for 1 h, followed by treatment with MeCN and subsequent crystallization of Eu(OTf)_3_(MeCN)_2_ at room temperature. The MeCN solution of Eu(OTf)_3_(MeCN)_2_ is then passed through a column filled with Zn/Hg amalgam to give the final product Eu(OTf)_2_(MeCN)_2_ (Scheme 64, **F**).^612^ Outside the triad of classic Ln^2+^ ions, only Tm(II) forms a stable triflate salt, [Tm(OTf)_2_(DME)_2_]_∞_.^613^ This was reported by Nocton and co-workers in 2017 and was obtained *via* reduction of Tm(OTf)_3_ with KC_8_ in DME at −35 °C (Scheme 64, **G**).^613^ Finally, tetravalent Ce(OTf)_4_ can be obtained from the reaction between CAN, K_2_CO_3_, and HOTf.^614^ However, Berthet and co-workers reported that anhydrous material cannot be obtained following this methodology because drying above 100 °C results in reduction to Ce(III).^615^ The authors were able to dry commercial Ce(OTf)_4_(H_2_O)_*x*_ by treating it with trifluoromethanesulfonic anhydride, TfOTf, followed by drying at 100 °C for 15 h.^615^

**Scheme 65:**
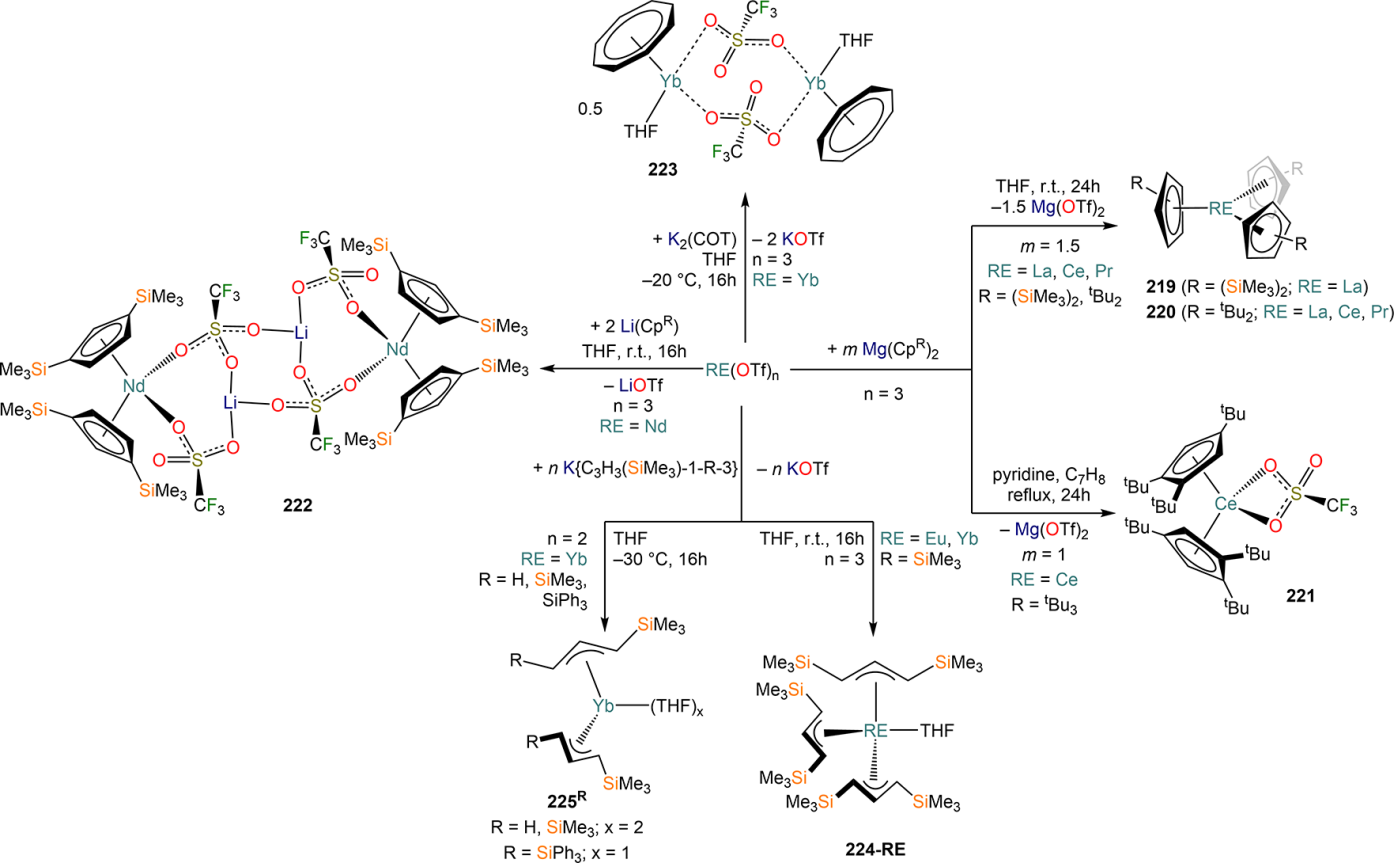
Selected Applications of Triflate Salts for the Synthesis of Cyclopentadienyl, COT, and Allyl RE Complexes^622−626^

RE(III) triflates have numerous applications as salt metathesis reagents, with a scope very similar to RE halides including numerous ligands, such as cyclopentadienyls, allyls, and a variety of mono- and multidentate donors.^616−621^ Therefore, only some selected applications are listed in this section to give an overview of the type of coordination chemistry accessible with these synthons. Because triflates are very good leaving groups, they have been used for the synthesis of sterically congested RE(Cp^R^)_3_ systems, such as [La(Cp″)_3_] (**219**) and [RE(Cp^tt^)_3_] (**220-RE**; RE = La, Ce, Pr),^622^ whose synthesis is typically more challenging when using halide precursors.^414^ For example, **219** can be obtained from the reaction between LaCl_3_ and K(Cp″) requiring first treatment in THF at room temperature for 24 h (to form transient La(Cp′′)_2_Cl), followed by reflux in toluene for an additional 24 h; whereas with La(OTf)_3_ and Mg(Cp″)_2_ the reaction only requires stirring at room temperature in THF overnight.^622^ Triflate salts have also been used for the synthesis of heteroleptic open metallocene complexes (*e.g.*, [Ce(Cp^ttt^)_2_(OTf)], **221**), though in some cases alkali metal salts are occluded in the products resulting in “ate” complex formation, as observed by Lappert and co-workers with the isolation of [{Nd(Cp″)_2_(μ-OTf)_2_Li}_2_] (**222**).^623^ From the reaction of K_2_COT with Y(OTf)_3_, Edelmann and Kilimann obtained dimeric [{Y(COT)(μ-OTf)(THF)}_2_] (**223**) (Scheme 65),^624^ which can then be functionalized further by substituting the triflate anion with various ancillary ligands *via* salt elimination. Moreover, John and co-workers have shown that triflate salts of various REs (Ce, Nd, Eu, Tb, and Yb) can react with the potassium salts of substituted allyls (Scheme 65) to form homoleptic allyl derivatives [RE{C_3_H_3_(SiMe_3_)_2_-1,3}_3_(THF)] (**224-RE**; RE = Ce, Nd, Tb).^625^ The same metathetical protocol is also effective for Yb(OTf)_2_ (Scheme 65) and the authors were able to obtain the bis-allyl complexes [Yb{C_3_H_3_(SiMe_3_)-1-R-3̅}_2_(THF)_*n*_] (**225**^**R**^; R = H, SiMe_3_, SiPh_3_; *n* = 1, 2).^626^ This is noteworthy, as there are very few applications in the literature of Ln(II) triflates as synthetic precursors in anaerobic synthesis.

## 9 Organometallic Reagents

Organometallic compounds of the REs are generally very reactive because of the high polarization of metal–carbon interactions. This is a problem typical of σ-bonded organometallic compounds but is also true for π-bonded organometallics, such as Cp, allyl, and COT derivatives. Additionally, RE organometallics can easily undergo β-hydride elimination and intramolecular C–H activation reactions. All these issues, together with the high electropositive character and large ionic radii of the REs, make the stabilization of organometallic species extremely challenging. However, over the years there has been a lot of progress in the stabilization of relatively simple RE organometallics which can function as synthons for the preparation of new compounds, particularly as reagents in protonolysis reactions. Simple σ-bonded RE alkyls (Me, ^n^Bu) and aryls (Ph, C_6_F_5_) have been well-investigated (section 9.1 and Table 9), though such compounds tend to be extremely reactive and often thermally sensitive. In this section Ln and RE aryls will not be discussed in detail because an extensive account of their preparation and application is provided in section 3 (*vide supra*). Alkyl complexes can be further stabilized by substituting the central carbon donor with silyl substituents, and this strategy has been used very effectively for the preparation of RE complexes α-silyl ligands {CH_2_SiMe_2_R}^−^ (R = Me, Ph) and {CH(SiMe_3_)_2_}^−^; such complexes are now common synthetic precursors in RE organometallic chemistry (section 9.2 and Table 10). Moreover, RE benzyl (Bn, {CH_2_Ph}^−^) complexes constitute another important class of organometallic starting materials (section 9.3 and Table 11); benzyl ligands are nominally alkyl-type donors, though they ligate the metal center in a multihapto fashion. Finally, organoaluminates, Ln(AlR_4_)_2_ and RE(AlR_4_)_3_ (R = Me, Et), are special types of organometallic reagents which can be effectively considered as masked alkyls stabilized by the formation of an adduct with AlR_3_ (section 9.4 and Table 12). Most of these synthetic precursors have been the subject of a recent comprehensive review by Zimmermann and Anwander;^11^ therefore, this section will aim to provide a broad overview of these reagents, with some highlights on the emergence of new reagents and new applications.

**Table 9:** Selected Simple RE Hydrocarbyls Used as Starting Materials^a^

RE	“RE(R)_3_”
Sc	*[Sc(Me)*_*3*_*]*_*n*_(633)
	*Sc(μ-Me)*_*6*_*Li*_*3*_*(THF)*_*1.2*_(639)
	[Sc_2_(μ-Me)_6_Li_3_(Et_2_O)_3_(THF)_2_]^639^
	*Sc(μ-*^*n*^*Bu)*_*6*_*Li*_*3*_*(THF)*_*x*_(640)
Y	*[Y(Me*_*3*_*)]*_*n*_(631,632)
	[Y_2_(μ-Me)_6_Li_3_(Et_2_O)_2_(THF)_3_]^639^
	*Y(μ-Me)*_*6*_*Li*_*3*_*(THF)*_*1.3*_(639)
	*Y(μ-Me)*_*6*_*Li*_*3*_*(TMEDA)*_*3*_(638)
La	*“LaMe*_*3*_*”*(632)
	*La(μ-Me)*_*6*_*Li*_*3*_*(TMEDA)*_*3*_(638)
	*La(μ-*^*n*^*Bu)*_*6*_*Li*_*3*_*(THF)*_*x*_(640)
Ce	[Ce(μ-Me)_6_Li_3_(TMEDA)_3_]^640^
	[Ce(μ-^n^Bu)_4_Li_2_(TMEDA)_2_]^640^
	*Ce(μ-*^*n*^*Bu)*_*6*_*Li*_*3*_*(THF)*_*x*_(640)
Pr	*Pr(μ-Me)*_*6*_*Li*_*3*_*(TMEDA)*_*3*_(638)
Nd	*Nd(μ-Me)*_*6*_*Li*_*3*_*(TMEDA)*_*3*_(638)
Sm	*Sm(μ-Me)*_*6*_*Li*_*3*_*(TMEDA)*_*3*_(638)
Eu	
Gd	*Gd(μ-Me)*_*6*_*Li*_*3*_*(TMEDA)*_*3*_(638)
	*Gd(μ-Me)*_*6*_*Li*_*3*_*(THF)*_*2.4*_(639)
Tb	[Tb_2_(μ-Me)_6_Li_3_(Et_2_O)_2_(THF)_3_]^639^
	*Tb(μ-Me)*_*6*_*Li*_*3*_*(TMEDA)*_*3*_(638)
	*Tb(μ-Me)*_*6*_*Li*_*3*_*(THF)*_*1.5*_(639)
Dy	*Dy(μ-Me)*_*6*_*Li*_*3*_*(TMEDA)*_*3*_(638)
	*Dy(μ-Me)*_*6*_*Li*_*3*_*(THF)*_*1.65*_(639)
Ho	*HoMe*_*3*_*- [Ho(Me)*_*3*_*]*_*n*_(634)
	[Ho(μ-Me)_6_Li_3_(TMEDA)_3_]^638^
	*Ho(μ-Me)*_*6*_*Li*_*3*_*(THF)*_*1.2*_(639)
Er	[Er(μ-Me)_6_Li_3_(TMEDA)_3_]^635,636^
	*Er(μ-Me)*_*6*_*Li*_*3*_*(THF)*_*2*_(639)
Tm	*Tm(μ-Me)*_*6*_*Li*_*3*_*(TMEDA)*_*3*_(638)
	*Tm(μ-Me)*_*6*_*Li*_*3*_*(THF)*_*1.7*_(639)
Yb	*[Yb(Me)*_*2*_*]*_*n*_(504)
	[Yb_2_(μ-Me)_6_Li_3_(Et_2_O)_3_(THF)_2_]^639^
	*Yb(μ-Me)*_*6*_*Li*_*3*_*(THF)*(639)
Lu	*[Lu(Me*_*3*_*)]*_*n*_(631,632)
	[Lu(μ-Me)_6_Li_3_(DME)_3_]^635,637,638^
	*Lu(μ-Me)*_*6*_*Li*_*3*_*(THF)*(639)
	[Lu(μ-^n^Bu)_4_Li_2_(TMEDA)_2_]^640^
	[Lu(μ-^n^Bu)_6_Li_3_(THF)_4_]^640^

a Compounds in italics have not been structurally authenticated.

**Table 10:** Selected α-Silyl-alkyl RE Complexes Used as Starting Materials^a^

RE	Ln(CR_3_)_2_	RE(CR_3_)_3_
Sc		[Sc(CH_2_SiMe_3_)_3_(THF)_2_]^641,656,657^
		[Sc(CH_2_SiMe_2_Ph)_3_(THF)_2_]^658^
		*Sc{CH(SiMe*_*3*_*)*_*2*_*}*(646)
Y		[Y(CH_2_SiMe_3_)_3_(THF)_2_]^641−643,659,660^
		[Y(CH_2_SiMe_3_)_3_(THF)_3_]^645^
		[Y{CH(SiMe_3_)_2_}_3_]^51^
La		*La(CH*_*2*_*SiMe*_*3*_*)*_*3*_*(THF)*_*3*_(661)
		[La{CH(SiMe_3_)_2_}_3_]^51^
Ce		[Ce{CH(SiMe_3_)_2_}_3_]^662^
Pr		
Nd		
Sm	[Sm{C(SiMe_3_)_3_}_2_]^663^	[Sm(CH_2_SiMe_3_)_3_(THF)_3_]^643^
		[Sm{CH(SiMe_3_)_2_}_3_]^51^
Eu	[Eu{C(SiMe_3_)_3_}_2_]^115^	
Gd		[Gd(CH_2_SiMe_3_)_3_(THF)_2_]^664^
Tb		[Tb(CH_2_SiMe_3_)_3_(THF)_2_]^660^
Dy		[Dy(CH_2_SiMe_3_)_3_(THF)_2_]^660,665^
Ho		[Ho(CH_2_SiMe_3_)_3_(THF)_2_]^660^
Er		[Er(CH_2_SiMe_3_)_3_(THF)_2_]^643^
		[Er{CH(SiMe_3_)_2_}_3_]^598^
Tm		[Tm(CH_2_SiMe_3_)_3_(THF)_2_]^660,666^
Yb	*Yb{CH(SiMe*_*3*_*)*_*2*_*}(Et*_*2*_*O)*_*2*_(299)	[Yb(CH_2_SiMe_3_)_3_(THF)_2_]^664,667^
	[Yb{C(SiMe_3_)_3_}_2_]^114^	
Lu		[Lu(CH_2_SiMe_3_)_3_(THF)_2_]^642,643,656^
		[Lu(CH_2_SiMe_3_)_3_(py)_2_]^668^
		[Lu(CH_2_SiMe_3_)_3_(THF)(DME)]^669^
		[Lu{CH(SiMe_3_)_2_}_3_]^573^

a Compounds in italics have not been structurally authenticated.

**Table 11:** Selected Benzyl Complexes of Divalent Lns and Trivalent REs^a^

RE	Ln(Bn)_2_	RE(Bn)_3_
Sc		[Sc(Bn)_3_(THF)_2_]^677^
		[Sc(Bn)_3_(THF)_3_]^677^
		*Sc(Bn*^*NMe2*^*)*_*3*_(670)
Y		[Y(Bn)_3_(THF)_3_]^673,685^
		[Y(Bn^NMe2^)_3_]^671^
		[Y(Bn)_2_(I)(THF)_3_]^671^
La		[La(Bn)_3_(THF)_3_]^675^
		[La(Bn^tBu^)_3_(THF)_3_]^675^
		*La(Bn*^*Me*^*)*_*3*_*(THF)*_*3*_(673)
		[La(Bn^NMe2^)_3_]^671,690^
Ce		[Ce(Bn)_3_(THF)_3_]^678^
Pr		[Pr(Bn)_3_(THF)_3_]^678^
		*Pr(Bn*^*NMe2*^*)*_*3*_(672)
Nd		[Nd(Bn)_3_(THF)_3_]^678^
		[Nd(Bn^NMe2^)_3_]^676^
Sm	*[Sm(Bn)*_*2*_*]*_*n*_(681)	[Sm(Bn)_3_(THF)_3_]^678^
	[Sm_2_(Bn)_4_(THF)(THP)_2_]_∞_^681^	[Sm(Bn^NMe2^)_3_]^672,689^
	[Sm{CH(C_6_H_4_NMe_2_)(SiMe_3_)}_2_(THF)_2_]^680^	
Eu	*[Eu(Bn)*_*2*_*]*_*n*_(681)	
	[Eu_4_(Bn)_8_(THF)_2_]^681^	
	*Eu{CH(C*_*6*_*H*_*4*_*NMe*_*2*_*)(SiMe*_*3*_*)}*_*2*_*(THF)*_*2*_(673)	
Gd		[Gd(Bn)_3_(THF)_3_]^339,678^
		*Gd(Bn*^*NMe2*^*)*_*3*_(672)
Tb		
Dy		[Dy(Bn)_3_(THF)_3_]^678^
		[Dy(Bn^NMe2^)_3_]^673^
Ho		[Ho(Bn)_3_(THF)_3_]^339^
		[Ho(Bn^NMe2^)_3_]^673^
Er		[Er(Bn)_3_(THF)_2_]^339^
		[Er(Bn)_2_(THF)_3_]^339,678^
		[Er(Bn)_2_(I)(THF)_3_]^679^
Tm		
Yb	*[Yb(Bn)*_*2*_*]*_*n*_(681)	[Yb(Bn^NMe2^)_3_]^672^
	[Yb(Bn)_2_(DME)_2_]^681^	
	[Yb(Bn)_2_(THP)_4_]^681^	
	[Yb{CH(C_6_H_4_NMe_2_)(SiMe_3_)}(THF)_2_]^680^	
Lu		[Lu(Bn)_3_(THF)_2_]^677^
		[Lu(Bn)_3_(THF)_3_]^677^
		*Lu(Bn*^*NMe2*^*)*_*3*_(672)

a Compounds in italics have not been structurally authenticated.

**Table 12:** Selected Alkylaluminate Complexes of Divalent Lns and Trivalent REs^a^

RE	Ln(AlR_4_)_2_	RE(AlR_4_)_3_
Sc		[Sc(AlMe_4_)_3_]·Al_2_Me_6_^633^
Y		*Y(AlMe*_*4*_*)*_*3*_(631,691)
		[Y(AlMe_4_)_3_]·Al_2_Me_6_^691^
La		[La(AlMe_4_)_3_]^692^
		[La(AlEt_4_)_3_]^704^
Ce		[Ce(AlMe_4_)_3_]^518,693^
Pr		[Pr(AlMe_4_)_3_]^692^
Nd		[Nd(AlMe_4_)_3_]^691^
		[Nd(AlMe_4_)_3_]·Al_2_Me_6_^694^
Sm	*[Sm(AlMe*_*4*_*)*_*2*_*]*_*n*_(556,696)	[Sm(AlMe_4_)_3_]^692^
	[Sm(AlEt_4_)_2_]_∞_^556^	
	[Sm(AlEt_4_)_2_(THF)_2_]^696^	
Eu	*[Eu(AlMe*_*4*_*)*_*2*_*]*_*n*_(518)	
	[Eu(AlEt_4_)_2_]_∞_^518,705^	
Gd		[Gd(AlMe_4_)_3_]^706^
Tb		[Tb(AlMe_4_)_3_]^706^
Dy		[Dy(AlMe_4_)_3_]^707^
Ho		[Ho(AlMe_4_)_3_]^693^
Er		[Er(AlMe_4_)_3_]^693^
Tm		[Tm(AlMe_4_)_3_]^518^
Yb	*[Yb(AlMe*_*4*_*)*_*2*_*]*_*n*_(556,705)	[Yb(AlMe_4_)_3_]^518^
	[Yb(AlEt_4_)_2_]_∞_^705^	[Yb(AlMe_4_)_3_]·Al_2_Me_6_^518^
	[Yb(AlEt_4_)_2_(THF)_2_]^696^	
Lu		[Lu(AlMe_4_)_3_]^692^

a Compounds in italics have not been structurally authenticated.

### 9.1 Simple Hydrocarbyls (Me, ^n^Bu, Ar)

**Scheme 66:**
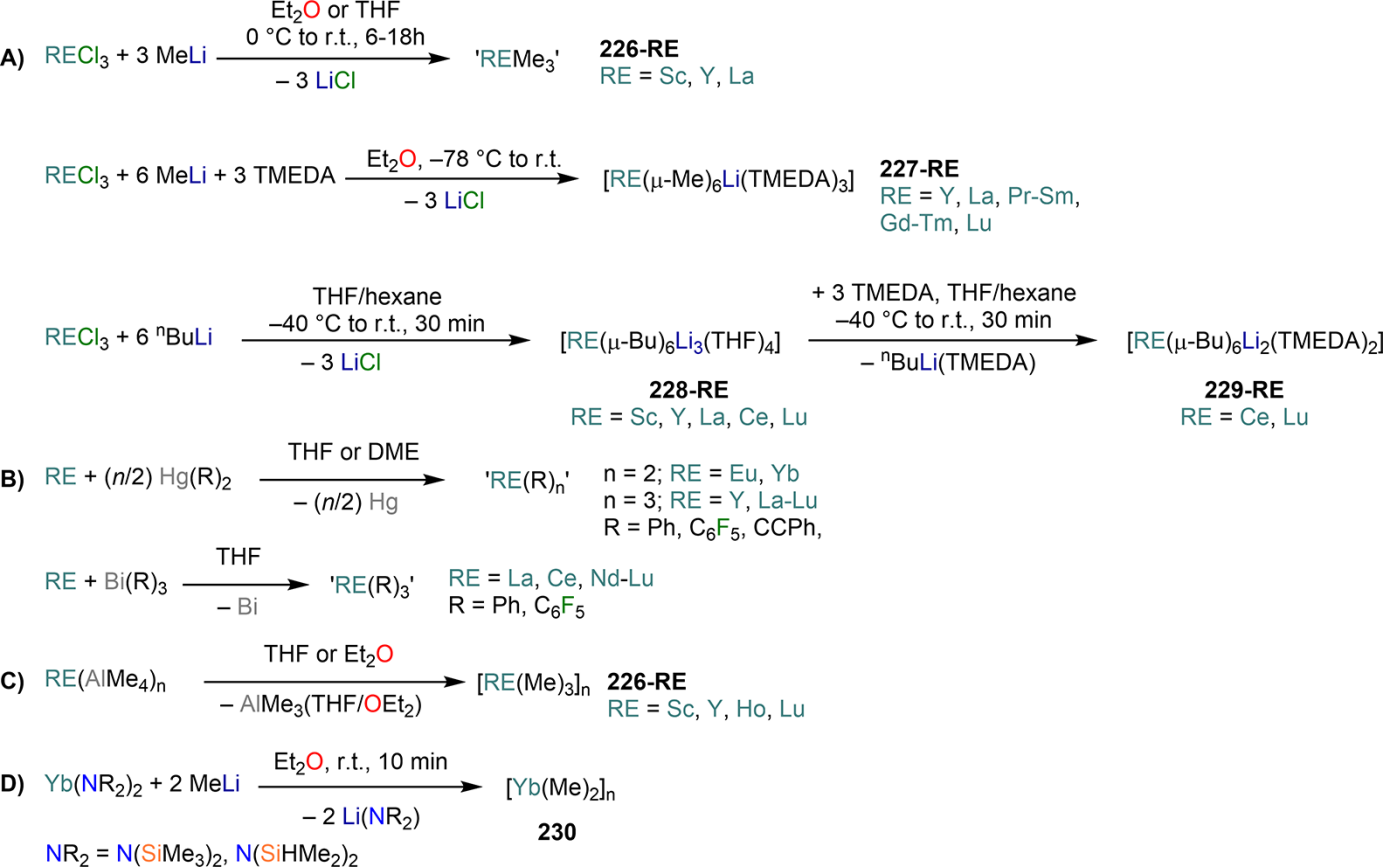
Synthesis of RE Hydrocarbyls *via* Salt Elimination,^635−638,640^ Donor-Cleavage,^631^ and Transmetalation Reactions^12,504^

Salt metathesis reactivity is the most common methodology employed for the synthesis of binary alkyl and aryl complexes (Scheme 66, **A**), though there are some notable exceptions such as (1) RT reactions with organomercurials and organobismuth reagents for the synthesis of aryl complexes (Scheme 66, **B**; *vide supra*, section 3.4),^12^ (2) donor cleavage reactions from organoaluminate precursors (Scheme 66, **C**),^631^ and (3) transmetalation reactions between amide precursors and MeLi (Scheme 66, **D**).^504^ Saran and co-workers reported the reaction of MeLi and PhLi with RECl_3_ (Sc, Y, La, and Pr) in 1970.^632^ In the case of reactivity with PhLi, the authors identified Sc(Ph)_3_ and Y(Ph)_3_*via* IR spectroscopy and elemental analysis, though with La and Pr they identified the formation of “ate” complex LiLa(Ph)_4_ from analogous reactivity. However, the formulas of the methyl derivatives “RE(Me)_3_” (**226**-**RE**; RE = Sc, Y, La) obtained from the reactions between RECl_3_ and MeLi could not be unequivocally identified.^632^ Neutral methyl complexes have been obtained with Ho, Lu, and Y by Anwander and co-workers, isolated as polymeric [RE(Me)_3_]_*n*_ (**226-RE**; RE = Sc,^633^ Y,^631^ Ho,^634^ Lu^631^) from the donor-cleavage reaction of aluminate precursors (Scheme 66; *vide infra*, section 9.4). Schumann reacted RECl_3_ with six equivalents of MeLi in the presence of TMEDA and obtained a series of hexamethyl “ate” complexes of formula [RE(μ-Me)_6_Li_3_(TMEDA)_3_] (**227-RE**; RE = Y, La, Pr, Nd, Sm, Gd, Tb, Dy, Ho, Er, Tm, and Lu).^635−638^ Furthermore, Okuda and co-workers carried out an in-depth study on the stability and reactivity of **227-RE** and were also able to obtain the pentametallic complexes [RE_2_(μ-Me)_6_Li_3_(Et_2_O)_*n*_(THF)_*m*_] (RE = Sc, *n* = 3, *m* = 2; RE = Y, Tb, *n* = 2, *m* = 3).^639^ Anwander and co-workers recently developed analogous chemistry with Ce, and in addition they extend this approach to the preparation of butyl derivatives [RE(μ-^n^Bu)_4_Li_3_(THF)_4_] (**228-RE**; RE = Sc, Y, La, Ce, Lu) and [RE(μ-^n^Bu)_4_Li_2_(TMEDA)_2_] (**229-RE**; RE = Ce, Lu).^640^ Finally, Anwander and co-workers reported the synthesis and reactivity of the neutral methyl complex [Yb(Me)_2_]_*n*_ (**230**), which is the only example of methyl derivative with a divalent Ln; **230** was obtained *via* transmetalation reaction between bis-silylamide precursors and MeLi.^504^

**Scheme 67:**
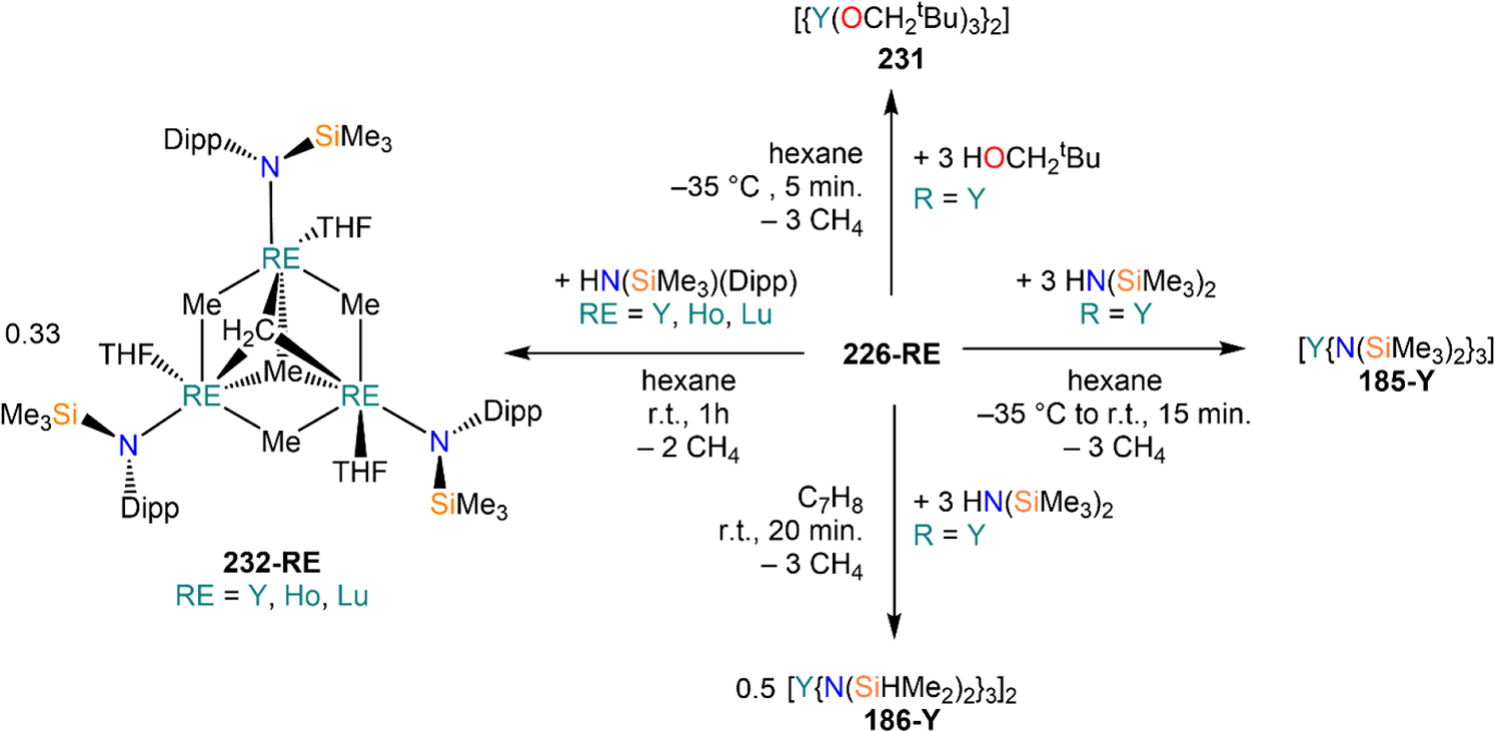
Selected Examples of Protonolysis Reactivity of **226-RE** with Brønsted Acids^542,631,634^

**Scheme 68:**
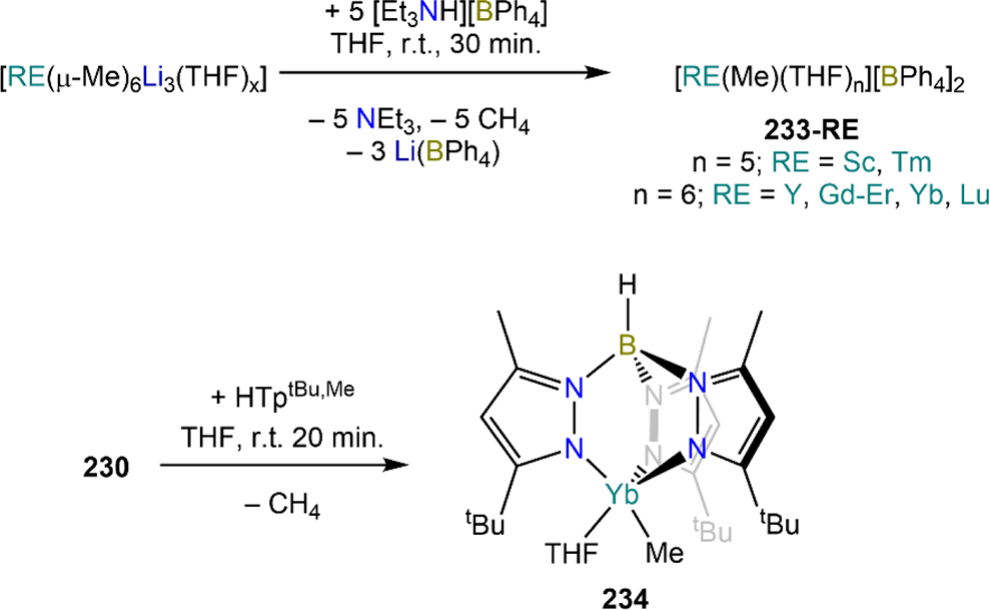
Reactivity of Hexamethyl Complexes with [Et_3_NH][BPh_4_]^639^ and Synthesis of Terminal Yb Methyl Complex **243** from **230**(504)

RE alkyl complexes (Table 9) are excellent protonolysis reagents owing to the very favorable p*K*_a_ (*vide supra*, Table 2) and react promptly with Brønsted acids. Anwander and co-workers treated **226-Y** with HN(SiMe_3_)_2_, HN(SiHMe_2_)_2_, and HOCH^t^Bu_2_ (Scheme 67), obtaining the *tris*-amide complexes **185-Y** and **186-Y** and alkoxide derivative Y(OCH^t^Bu_2_)_3_ (**231**).^542,631^ Interestingly, reactivity of **226-RE** with HN(SiMe_3_)(Dipp) leads to the deprotonation of one methyl ligand and formation of methylidene bridged cluster [RE_3_{N(SiMe_3_)(Dipp)}_3_(μ_2_-Me)_3_(μ_3_-Me)(THF)_3_] (**232-RE**; RE = Y, Ho, Lu) (Scheme 67).^634^ In a similar fashion, Berger *et al.* investigated the reactivity of **229-Ce** with HOCH^t^Bu_2_, which led to the isolation of the cluster [Ce_2_Li_3_(OCH_2_^t^Bu)_9_(HOCH_2_^t^Bu)_2_(THF)].^640^ Kramer *et al.* reacted the THF adducts of hexamethyl complexes **227-RE** with five equivalents [Et_3_NH][BPh_4_] (Scheme 68), affording cationic complexes [RE(Me)(THF)_*n*_][BPh_4_]_2_ (**233-RE**; RE = Sc, Tm, *n* = 5; RE = Y, Gd–Er, Yb, Lu, *n* = 6).^639^ As would be expected, **230** is also an excellent protonolysis reagent and reacts smoothly with HTp^tBu,Me^ (Scheme 68) to give the terminal methyl complex [Yb(Tp^tBu,Me^)(Me)(THF)] (**234**).^504^

### 9.2 α-Silyl-alkyls

**Scheme 69:**
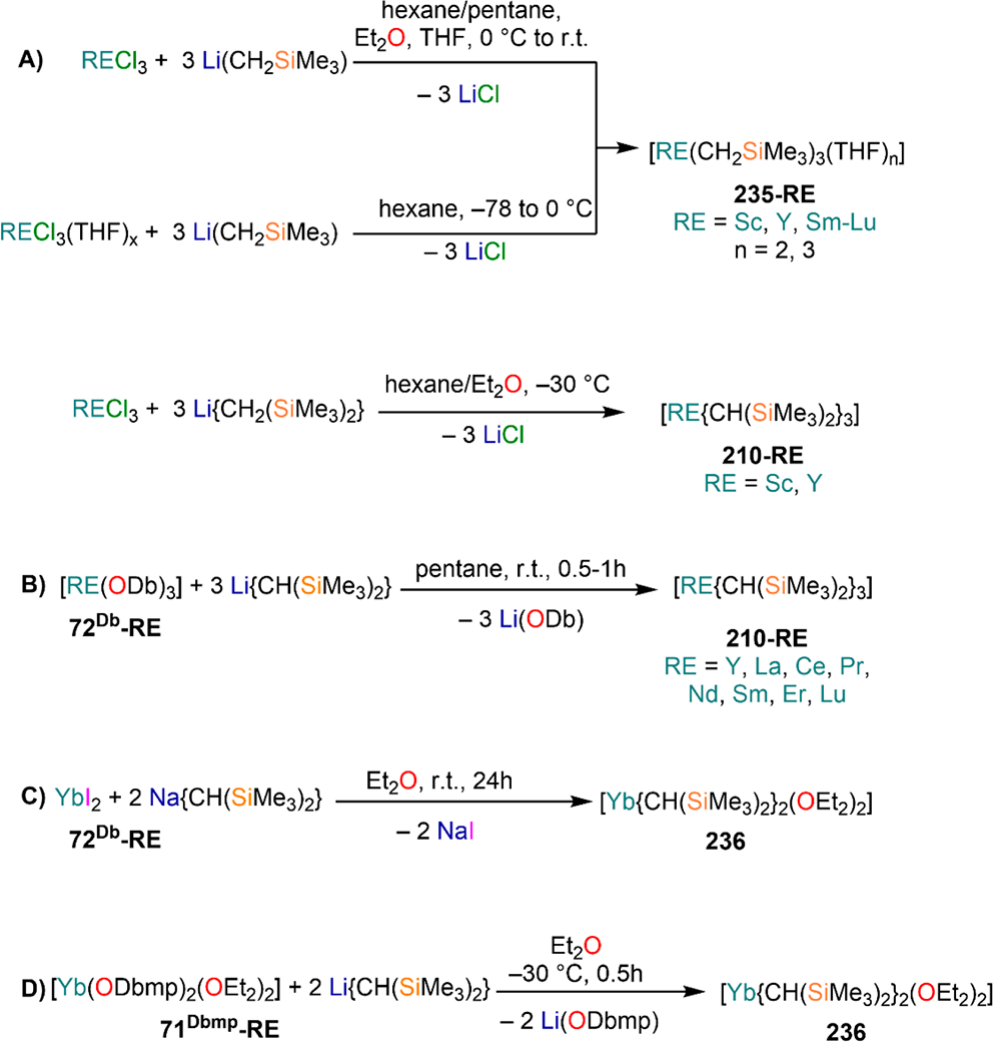
Synthesis of α-Silyl Alkyl complexes **235-RE**,^641^**210-RE**,^51^ and **236**(299)

RE(III) complexes with α-silyl-alkyl ligands (Table 10) were first reported by Lappert and Pearce; in their seminal report, the authors obtained [RE(CH_2_SiMe_3_)_3_(THF)_2_] (**235-RE**; RE = Sc, Y) from the reaction of RECl_3_(THF)_*x*_ with Li{CH_2_SiMe_3_} in a mixture of hexane and ether (Scheme 69, **A**).^641^ This salt elimination method has since been applied to obtain analogous complexes with most of the trivalent REs,^642−645^ with the exception of the larger Lns La–Nd. The larger {CH(SiMe_3_)_2_}^−^ ligand was originally introduced in RE chemistry by Lappert and Barker in 1974, who obtained Sc and Y complexes [RE{CH(SiMe_3_)_2_}_3_] (**210-RE**) *via* salt elimination reactions, though no structural authentication was provided (Scheme 69, **A**).^646^ However, it has been amply demonstrated that salt elimination reactions can often lead to the formation of “ate” complexes with these systems.^647^ Nonetheless, salt occlusion can be avoided by following the transmetalation route devised by Lappert and co-workers, in which aryloxide complexes [RE(ODb)_3_] (**72**^**Db**^**-RE**) are treated with Li{CH(SiMe_3_)_2_} to give neutral *tris-*alkyl complexes **210-RE** (Scheme 69, **B**).^51^ It is important to note that these ligands are not ideal for stabilizing neutral *bis*-alkyl Ln(II) complexes; Lappert and co-workers obtained [Yb{CH(SiMe_3_)_2_}_2_(OEt_2_)_2_] (**236**) *via* both analogous salt metathesis and transmetalation methodologies, but no structural information has been reported to validate these results (Scheme 69, **C** and **D**).^299^ It is also noteworthy that other ligand variations have been introduced, particularly the very sterically demanding {C(SiMe_3_)_3_}^−^, which has enabled the stabilization and structural authentication of neutral homoleptic *bis-*alkyl complexes [Ln{C(SiMe_3_)_3_}_3_] (Ln = Sm, Eu, Yb), which were obtained *via* salt elimination reactions.^114^

**Scheme 70:**
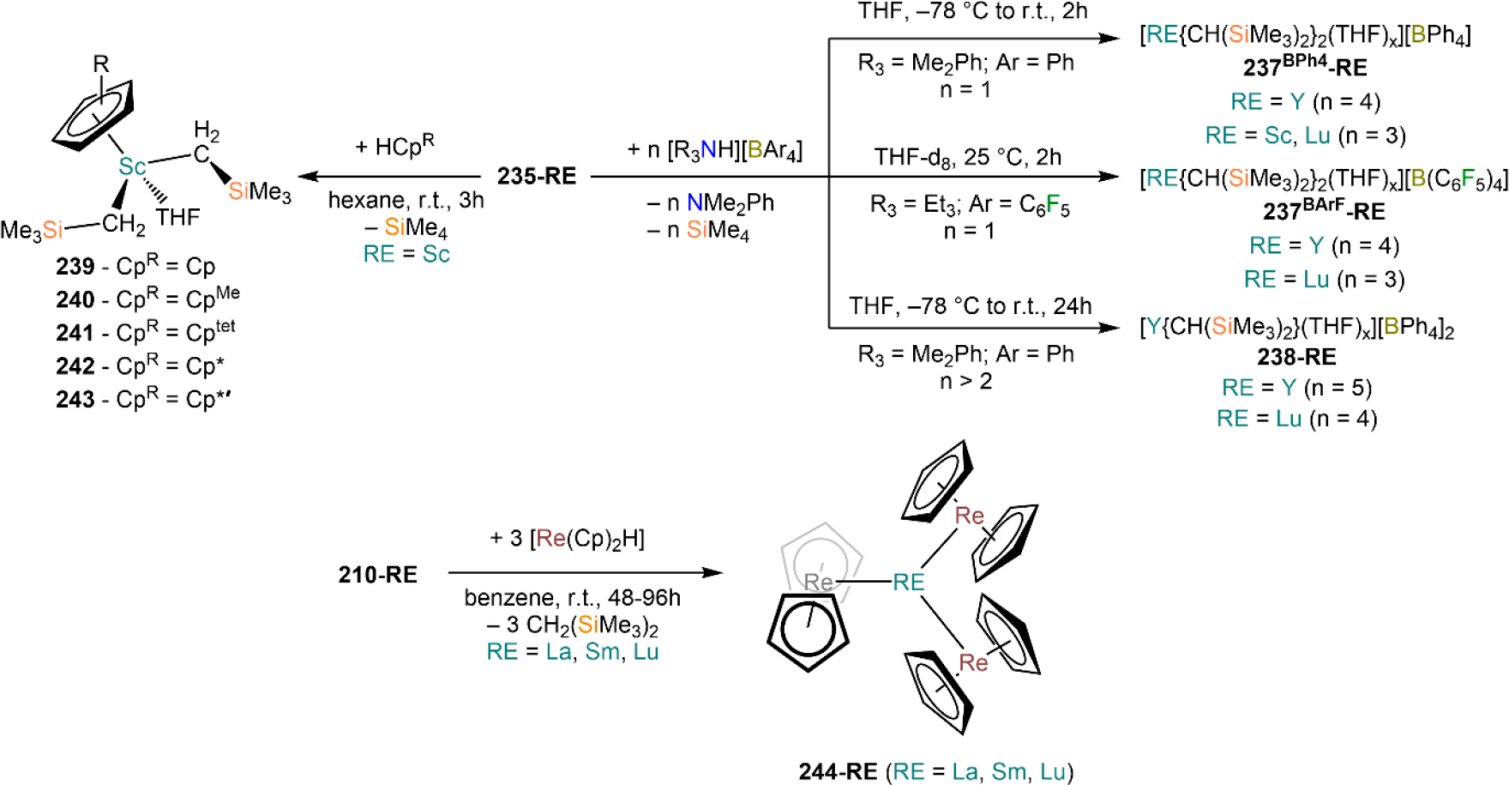
Selected Examples of Protonolysis Reactivity of **235-RE** with Ammonium Salts^648,649^ and Substituted Cyclopentadienes^650^ and Reactivity of **210-RE** with [Re(Cp)_2_H]^655^

The applications of both **235-RE** and **210-RE** as synthetic precursors in protonolysis reactions are numerous and well-established.^11^**235-RE** is a particularly useful reagent owing to the production of volatile SiMe_4_ as byproduct upon treatment with Brønsted acids. This strategy has been applied to produce complexes with a plethora of supporting ligands, and their utility as synthetic precursors has been extensively reviewed before.^11^ Therefore, only some representative examples are discussed herein. Okuda and co-workers showed that by treating **235-RE** (RE = Sc, Y, Lu) with [Me_2_PhNH][B(C_6_F_5_)_4_] or [Et_3_NH][BPh_4_] the cationic complexes [RE(CH_2_SiMe_3_)_2_(THF)_*n*_][BR_4_] (**237**^**BPh4**^**-RE**: R = BPh_4_, RE = Sc, Y, Lu; **237**^**BArF**^**-RE**: R = C_6_F_5_, RE = Y, Lu; *n* = 3, 4) are produced (Scheme 70).^648,649^ When at least two equivalents of Brønsted acid are employed, the dicationic complexes [RE(CH_2_SiMe_3_)(THF)_*n*_][BPh_4_]_2_ (**238-RE**: RE = Y, *n* = 5; RE = Lu, *n* = 4) are obtained (Scheme 70).^648,649^ Interestingly, **235-RE** react also with Lewis acids (*e.g.*, BPh_3_, B(C_6_F_5_)_3_, AlR_3_) to give analogous cationic complexes upon abstraction of one or more alkyl ligands.^648,649^ The efficacy of **235-RE** as a protonolysis reagent has also been demonstrated by Li *et al.* with the reaction of **235-Sc** with a series of substituted cyclopentadienes (HCp, HCp^Me^, HCp^tet^, HCp*, and HCp*′) to produce half-sandwich dialkyl complexes [Sc(Cp^R^)(CH_2_SiMe_3_)_2_(THF)] [Cp^R^ = Cp (**239**), Cp^Me^ (**240**), Cp^tet^ (**241**), Cp* (**242**), Cp*′ (**243**) - Cp^tet^ = {C_5_HMe_4_}^−^, Cp*′ = {C_5_Me_4_SiMe_3_}^−^] (Scheme 70).^650^ This approach can be extended to different REs, as demonstrated by Hou and co-workers with the preparation of **244-RE** (RE = Sc, Y, Gd, Tb, Dy, Ho, Er, Tm, Yb, Lu).^651^ The use of **210-RE** in similar alkane elimination reactions has also been explored, but these applications are more limited compared to **235-RE** owing to the kinetic inertia provided by the more sterically demanding {CH(SiMe_3_)_2_}^−^ ligand.^11^ Nonetheless, an important application of these reagents has been demonstrated by Kempe with the preparation of heterobimetallic RE-TM species *via* alkane elimination reactions between RE alkyls and TM hydride precursors.^652−654^**210-RE** (RE = La, Sm, and Lu) reacts with [RE(Cp)_2_(H)] in benzene at room temperature to give the tetrametallic complexes [RE{Re(Cp)_2_}_3_] (**244-RE**; RE = La, Sm, Lu);^655^ these species are extremely sensitive, which according to the authors is the reason behind the low yields of these reactions. The choice of reagent and solvent conditions in Kempe’s methodology is crucial, as the presence of THF leads to undesired side-reactivity. Because of this, only unsolvated **210-RE** can be employed.

### 9.3 Benzyls

**Scheme 71:**
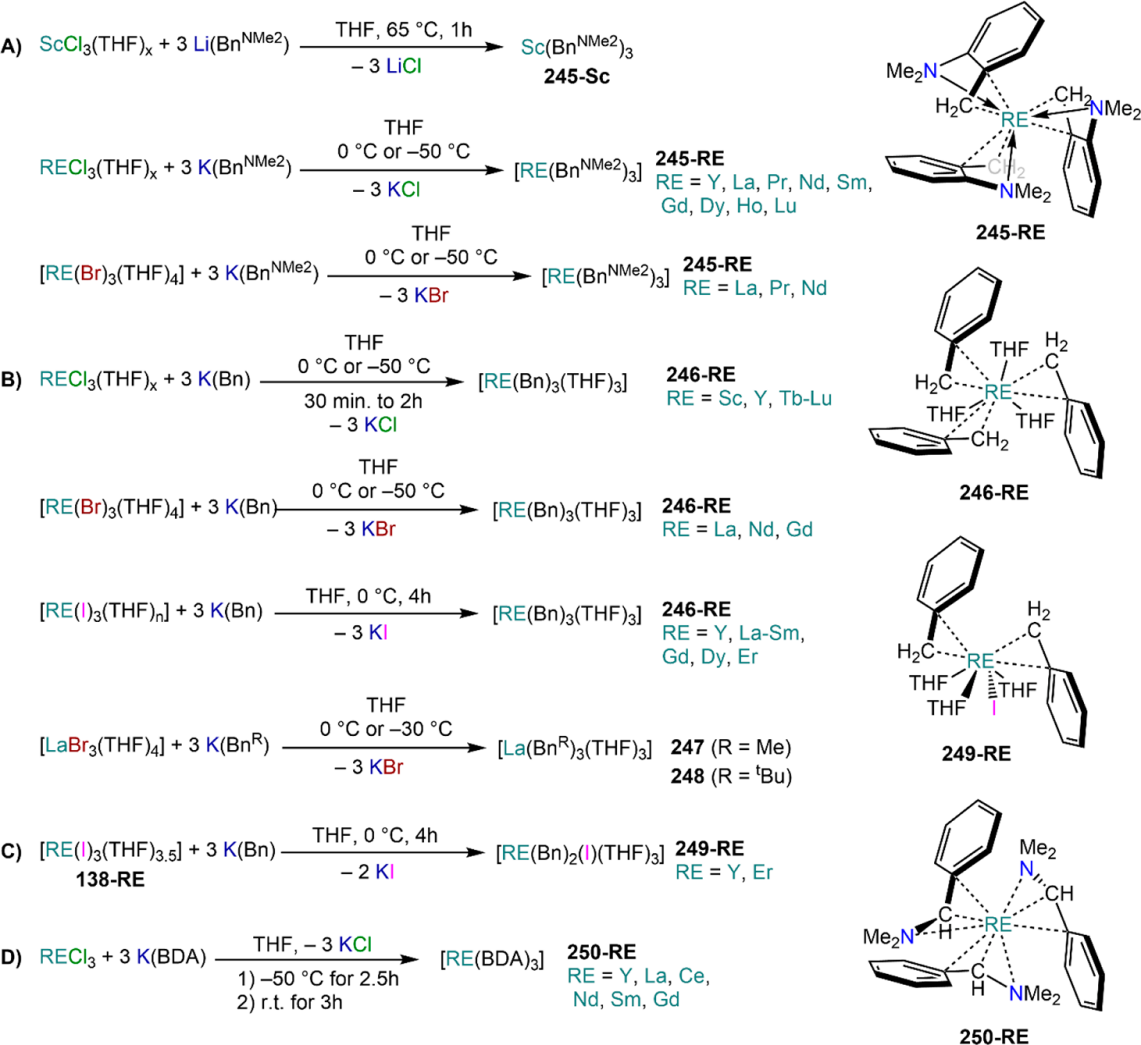
Synthesis of RE(III) Benzyl Complexes

A range of benzyl (Bn, {CH_2_Ar}^−^) complexes have been reported with trivalent REs, which comprise (1) unsubstituted benzyls, (2) substituted benzyls, and (3) bidentate benzyls (Table 11). Compared to other hydrocarbyls, benzyls offer the advantage of acting as multihapto ligands and can delocalize the negative charge from the benzylic position into the phenyl ring; because of this they impart greater stability to resulting RE complexes while still preserving the basicity (or nucleophilicity) of alkyl fragments and are therefore very useful synthetic precursors for protonolysis reactivity. Ln(Bn)_2_ and RE(Bn)_3_ are obtained *via* salt elimination reactions between LnI_2_ or REX_3_ and benzylpotassium salts, though occasionally Li salts are also employed. That was indeed the case when Mazer first reported in 1978 the reaction between ScCl_3_ and three equivalents of Li(Bn^NMe2^) (Bn^NMe2^ = {CH_2_[C_6_H_4_(NMe_2_)-2]}^−^), leading to the formation of *tris-*benzyl complex Sc(Bn^NMe2^)_3_ (**245-Sc**) (Scheme 71, **A**).^670^ Workup of the reaction following Mazer’s method can be frustrated by the solubility of the byproduct (LiCl), so more convenient routes entail the use of benzylpotassium salts. Harder^671−673^ and then Hou^674^ obtained benzyl complexes **245-RE** (RE = Y, La, Pr, Nd, Sm, Gd, Dy, Ho, Lu) by employing K(Bn^NMe2^) with either RECl_3_ (RE = Y, Nd, Gd, Sm, Dy, Ho, Yb, Lu—both anhydrous and solvated)^671−673^ or [RE(Br)_3_(THF)_4_] (RE = La, Pr, Nd) (Scheme 71, **A**).^674^ RE complexes with monodentate benzyl ligands were first reported by Hessen and co-workers, who prepared [La(Bn)_3_(THF)_3_] (**246-La**) and [La(Bn^Me^)_3_(THF)_3_] (**247**; Bn^Me^ = {CH_2_(C_6_H_4_Me-4)}^−^) from [La(Br)_3_(THF)_4_] and KBn^R^ in THF at 0 °C (Scheme 71, **B**).^675^ In a similar vein, Harder reacted [La(Br)_3_(THF)_4_] with K(Bn^tBu^) (Bn^tBu^ = {CH_2_(C_6_H_4_^t^Bu-4)}^−^) in THF at −50 °C to give [La(Bn^tBu^)_3_(THF)_3_] (**248**).^676^ RECl_3_ precursors are also a good match with benzylpotassium for the preparation of **246-RE***via* salt metathesis, as demonstrated by Harder,^676^ Roesky,^677^ and Diaconescu.^339^ Interestingly, Diaconescu and co-workers observed that **246-Ho** and **246-Er** could be obtained in good yields starting from corresponding RECl_3_; however, bromide salts were required to obtain benzyl complexes of larger metals, *i.e.*, Nd and Gd.^339^ Liddle and co-workers reported the convenient synthesis of **246-RE** (RE = Y, La–Sm, Gd, Dy, Er) from [RE(I)_3_(THF)_*n*_] (**138-RE**) and K(Bn) in THF at 0 °C (Scheme 71, **C**).^678^ It is noteworthy that attempts to obtain a Yb(III) analogue led to the isolation of the mixed-valent Yb(II)/Yb(III) complex [Yb(Bn)(THF)_5_][Yb(Bn)_4_(THF)_2_].^678^ Additionally, Liddle and co-workers reported the heteroleptic benzyl complexes [RE(Bn)_2_(I)(THF)_3_] (**249-RE**; RE = Y, Er) from the reaction of **138-Y** and **138-Er** with two equivalents of K(Bn) (Scheme 71, **C**).^679^ Finally, another class of benzyl complexes has been reported by Schmidt and Behrle using *N*,*N*-dimethylbenzylamine (DBA) metalated in the α-position.^516^ Analogously to the other methodologies employed for the synthesis of **245-RE** and **246-RE**, Schmidt and Behrle reacted three equivalents of K(DBA) with RECl_3_ (RE = Y, La, Ce, Nd, Sm, Gd) in THF at −50 °C (Scheme 71, **D**), obtaining [RE(DBA)_3_] (**250-RE**; RE = Y, La, Ce, Nd, Sm, Gd).^516^ X-ray studies of **250-RE** reveal that DBA ligands coordinate the metal centers in an η^4^-fashion.^516^

**Scheme 72:**
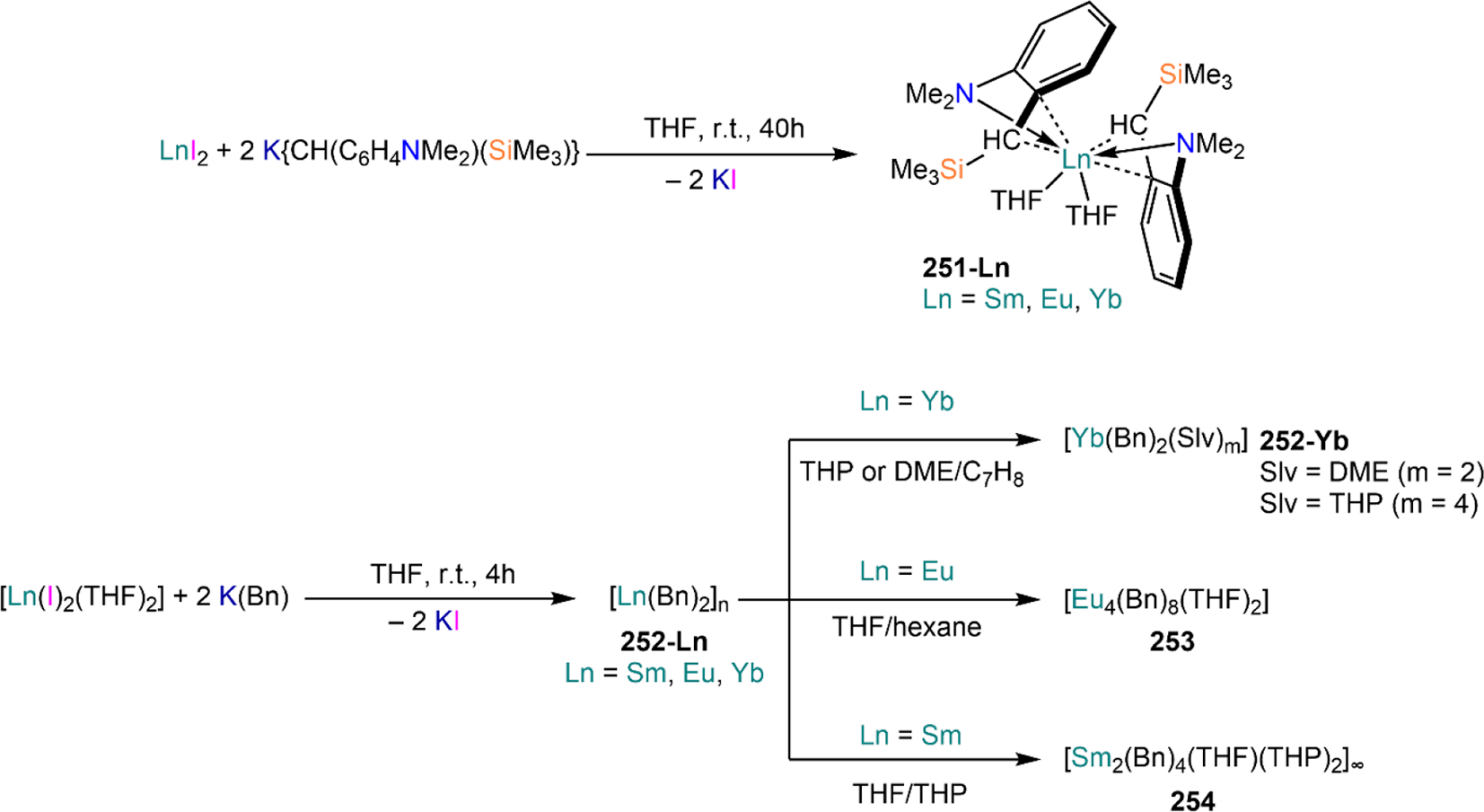
Synthesis of Ln(II) Benzyl Complexes **251-Ln**,^680^**252-Ln**, **253**, and **254**(681)

Benzyl derivatives of divalent Lns have been reported by Harder and Anwander. Harder and co-workers obtained Sm, Eu, and Yb benzyls [Ln{CH(C_6_H_4_NMe_2_)(SiMe_3_)}_2_(THF)_2_] (**251-Ln**; Ln = Sm, Eu, Yb) by reacting LnI_2_ with K{CH(C_6_H_4_NMe_2_)(SiMe_3_)} (Scheme 72).^680^ This bidentate benzyl ligand imparts greater stability to the complexes owing to the stabilization of the carbanion by the α-silyl substituent. Recently, Anwander and co-workers have reported the synthesis of [Ln(Bn)_2_]_*n*_ (**252-Ln**; Ln = Sm, Eu, Yb) from [Ln(I)_2_(THF)_2_] and two equivalents of K(Bn) (Scheme 72).^681^ The amorphous **252-Ln** can be recrystallyzed in the presence of donors to give discrete molecular species, *i.e.*, [Eu_4_(Bn)_8_(THF)_2_] (**253**), [Sm_2_(Bn)_4_(THF)(THP)_2_]_∞_ (**254**), [Yb(Bn)_2_(THP)_4_] (**252-Yb·**4THP), and [Yb(Bn)_2_(DME)_2_] (**252-Yb·**2DME).^681^

**Scheme 73:**
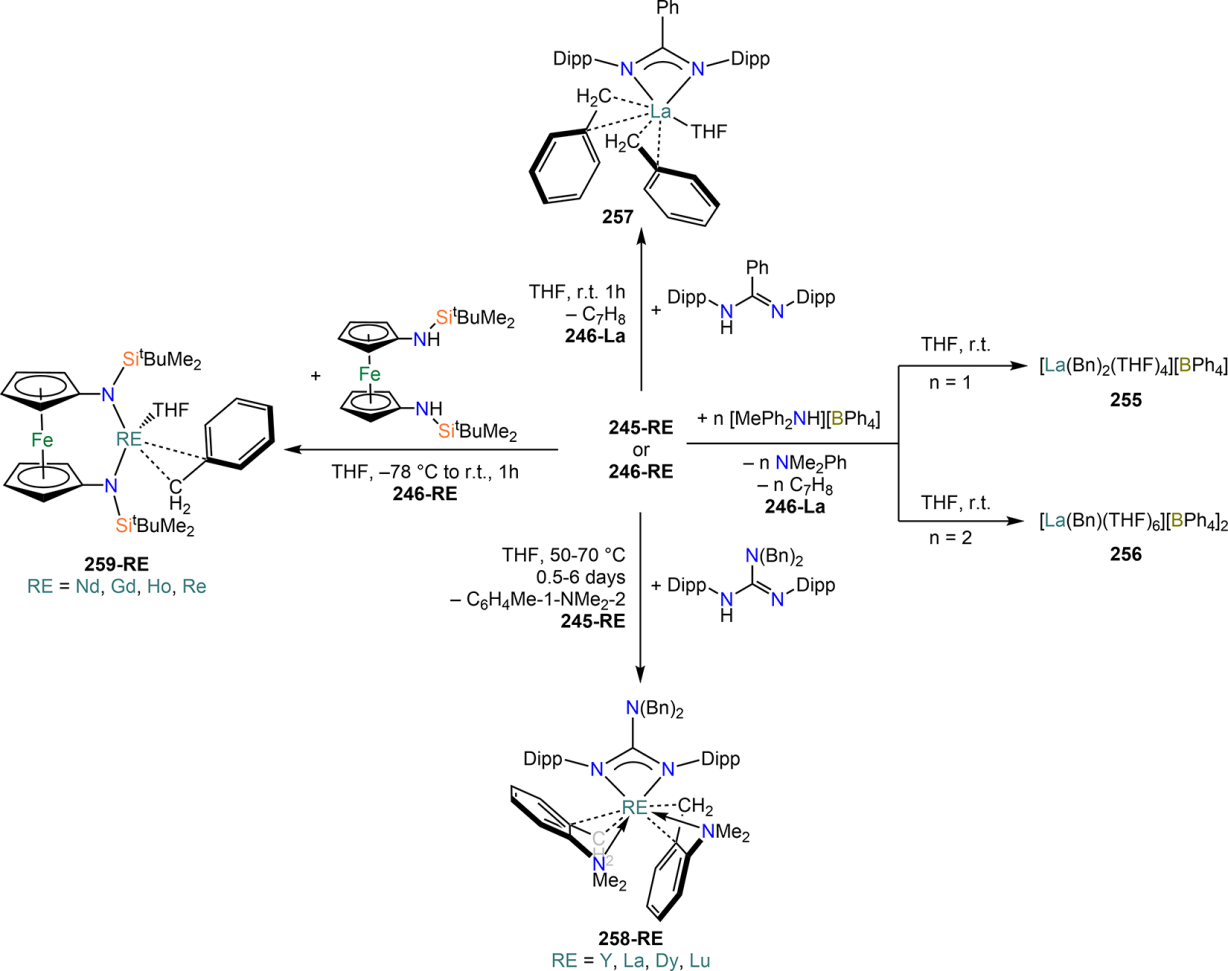
Reactivity of RE(III) Benzyl Precursors with Bidentate *N*-Donors^339,675,682,683^

RE benzyl complexes have been used as synthetic precursors in protonolysis reactions with a range of substrates. Hessen and co-workers treated **246** and **247-La** with either one or two equivalents of [MePh_2_NH][BPh_4_] and obtained the corresponding monocationic or dicationic complex, *i.e.*, [La(Bn)_2_(THF)_4_][BPh_4_] (**255**), [La(Bn^Me^)_2_(THF)_4_][BPh_4_], [La(Bn)(THF)_6_][BPh_4_]_2_ (**256**), and [La(Bn^Me^)(THF)_6_][BPh_4_]_2_ (Scheme 73).^675^ These starting materials can also be used to generate heteroleptic alkyl complexes with bidentate nitrogen donors, such as amidinates (**257**),^675^ guanidinates (**258-RE**; RE = Y, La, Dy, Lu),^682,683^ and ferrocenyl *bis-*amides (**259-RE**; RE = Nd, Gd, Ho, Er) (Scheme 73).^339^

**Scheme 74:**
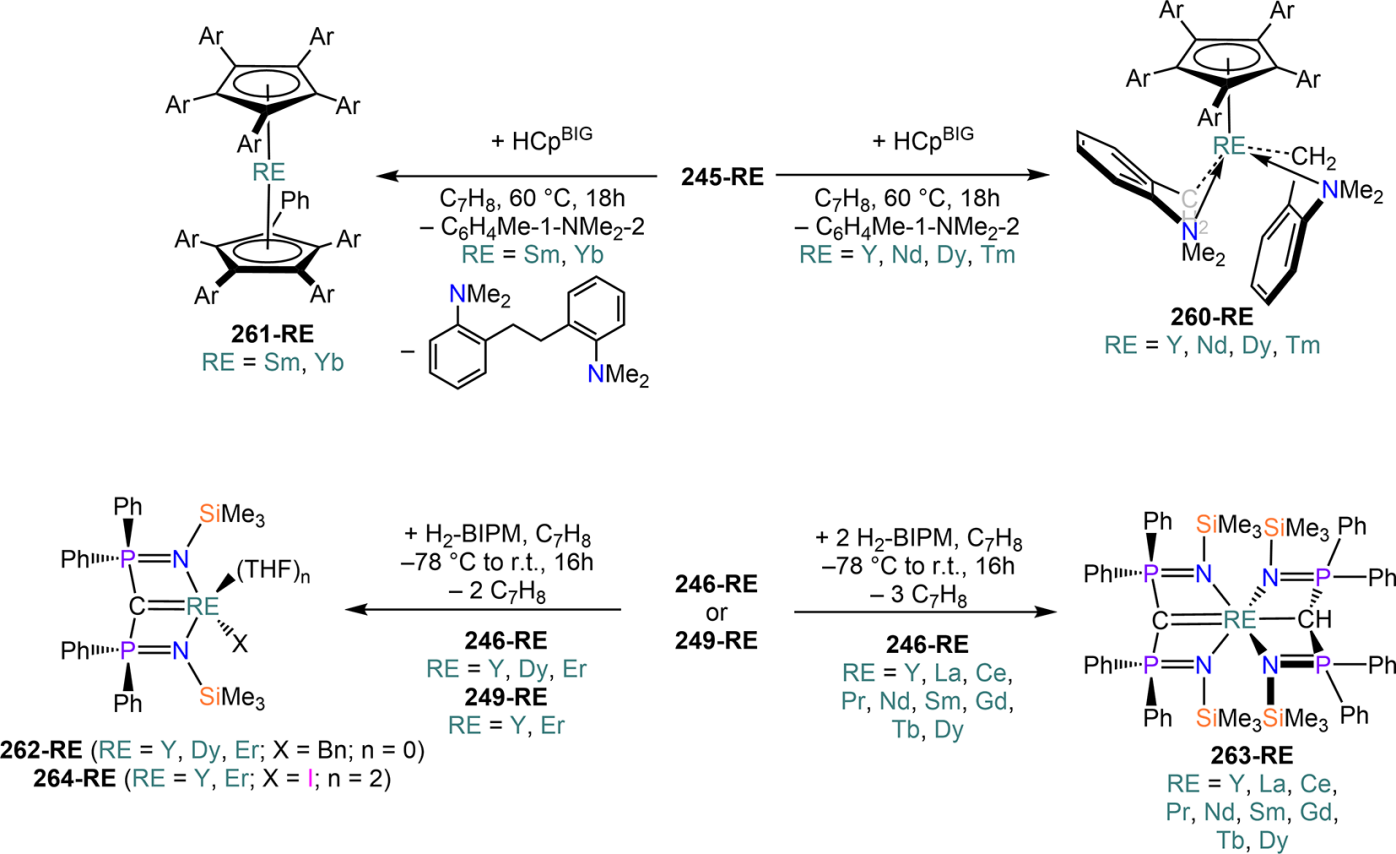
Reactivity of RE(III) Benzyl Precursors with H_2_-BIPM^678,679,685−688^ and HCp^BIG^^672,684^

Furthermore, RE benzyls can be employed to generate new organometallic complexes. Harder first showed that **245-RE** can deprotonate HCp^BIG^ (Cp^BIG^ = {C_5_Ar_5_}^−^; Ar = C_6_H_4_R-4; R = Et, ^i^Pr, ^n^Bu) to afford the heteroleptic Cp derivatives [RE(Cp^BIG^)(Bn^NMe2^)_2_] (**260-RE**; RE = Y, Nd, Dy, Tm).^672,684^ Interestingly, with **245-Sm** and **245-Yb** the metals are reduced to their divalent state and the homoleptic metallocenes [RE(Cp^BIG^)_2_] (**261-RE**; RE = Sm, Yb) are formed, with concomitant formation of byproduct 1,2-di(2-Me_2_N-phenyl)ethane as a result of the coupling of two benzyl radicals (Scheme 74).^672,684^ In addition to this, Liddle and co-workers used **246-RE** and **249-RE** to perform a double-deprotonation of H_2_–BIPM and generate alkylidene complexes [RE(BIPM)(Bn)] (**262-RE**; RE = Y, Dy, Er),^678,685^ [RE(BIPM)(H-BIPM)] (**263-RE**; RE = Y, La, Ce, Pr, Nd, Sm, Gd, Tb, Dy),^678,686,687^ and [RE(BIPM)(I)(THF)_2_] (**264-RE**; RE = Y, Er) (Scheme 74).^679,688^ Finally, Schmidt and Behrle screened a series of protic substrates (*i.e.*, HODb, H_2_NDipp, HN(SiMe_3_)_2_) with the DBA complexes **250-Y** and **250-La** and obtained clean conversion to [RE(ODb)_3_] (**72**^**Db**^**-RE**; RE = Y, La), RE(HNDipp)_3_, and [RE{N(SiMe_3_)_2_}_3_] (**185-RE**; RE = Y, La).^516^

**Scheme 75:**
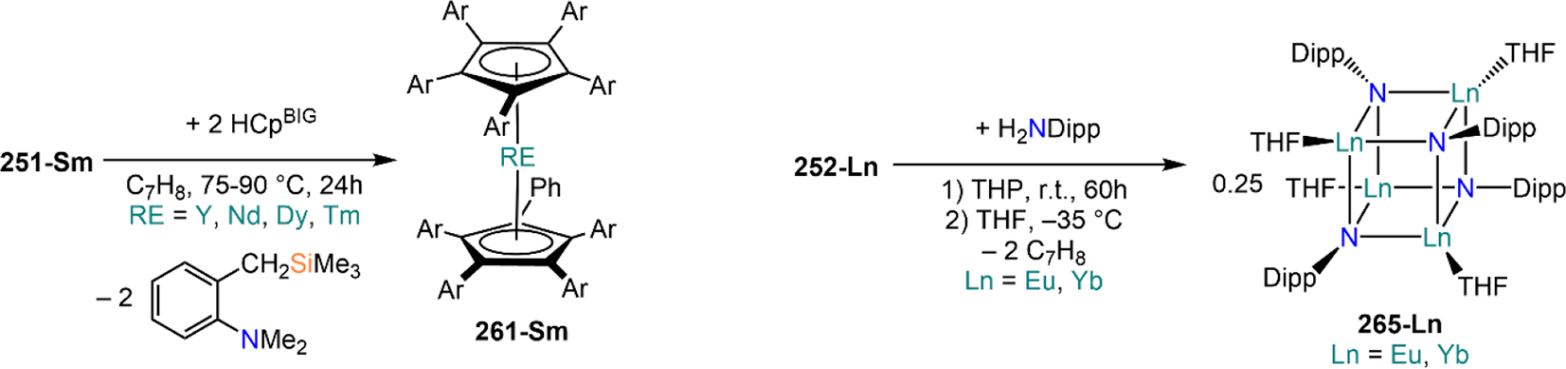
Reactivity of Ln(II) Benzyls with HCp^BIG^ and H_2_NDipp^681,689^

Applications of Ln(II) benzyl complexes are more scarce compared to their trivalent counterparts. Harder obtained Sm(II) metallocene **261-Sm** from the direct deprotonation of HCp^BIG^ with **251-Sm** (Scheme 75).^689^ Moreover, Anwander and co-workers reacted **252-Eu** and **252-Yb** with H_2_NDipp (Scheme 75), obtaining a cubane cluster with bridging imido ligands, [{Ln(η^3^-NDipp)(THF)}_4_] (**265-Ln**; Ln = Eu, Yb).^681^

### 9.4 Organoaluminates

Since the first report of [Y(AlMe_4_)_3_] (**266-Y**) and [Nd(AlMe_4_)_3_] (**266-Nd**) by Evans *et al.* in 1995,^691^ RE tetraalkylaluminates have emerged as a very interesting class of synthetic precursors in RE and f-element chemistry.^11^ One of the intriguing aspects of organoaluminates is their dual chemical behavior: on the one hand they can be deemed “masked-alkyl” complexes formed as adducts of AlR_3_, which can be used for protonolysis reactivity; on the other hand they could be regarded as ionic complexes of the {AlR_4_}^−^ ligand, which can act as a *pseudo*-halide in salt elimination reactions. Their preparation and chemical properties were incorporated in the detailed account on RE alkyl chemistry by Anwander and Zimmermann in 2010;^11^ therefore, this section aims to give a broad overview of their preparation and synthetic applications.

**Scheme 76:**
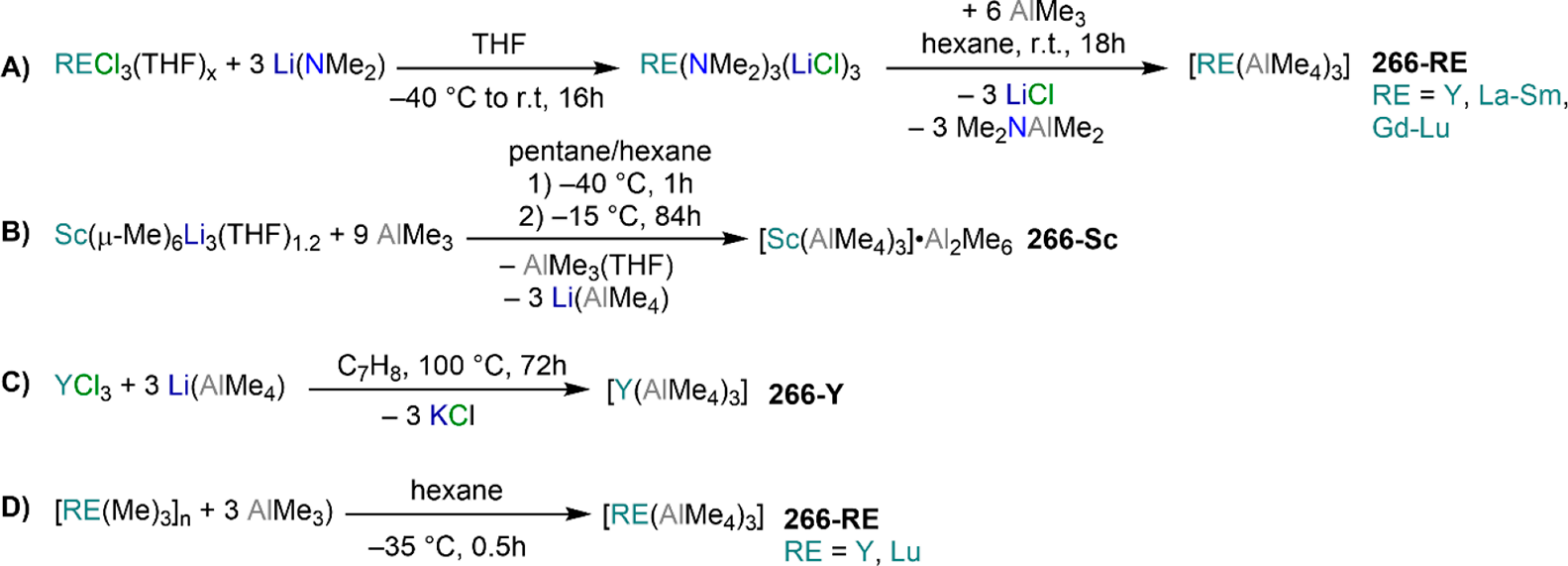
Synthesis of RE(III) Aluminates^691,692,694^

Evans *et al.* prepared [RE(AlMe_4_)_3_] (**266-RE**; RE = Y, Nd) by treating amide precursor RE(NMe_2_)_3_(LiCl)_3_ with excess AlMe_3_ (Scheme 76, **A**).^691^ This synthetic approach has since been optimized by generating the amide precursors *in situ* from RECl_3_(THF)_*x*_ and Li(NMe_2_), and the methodology has been extended to most of the REs.^518,692,693^ The overall reaction can be viewed as an amide-methyl metathesis generating Me_2_NAlMe_2_ and putative methyl complex “RE(Me)_3_”, with the latter converted into **266-RE** upon adduct formation with excess AlMe_3_.^11^ The Sc analogue **266-Sc** cannot be obtained *via* amide-methyl exchange (Scheme 76, **B**); nonetheless, treatment of the hexamethyl Sc complex Sc(μ-Me)_6_Li_3_(THF)_1.2_^639^ with more than six equivalents of AlMe_3_ affords [Sc(AlMe_4_)_3_]·Al_2_Me_6_ (**266-Sc**·Al_2_Me_6_). Organoaluminates obtained with these methods can cocrystallize with Al_2_Me_6_, which can be removed upon recrystallization.^691,692,694^ Most of these species are also relatively thermally robust, especially if compared to alkyl derivatives, and can also be sublimed. However, an important aspect of the chemistry of **266-RE** is the ability of polar solvents to trigger their degradation *via* donor-induced cleavage of the aluminate ligand (*vide infra*).^11,695^ It is possible to obtain **266-RE** also by exchanging other ligands with AlMe_3_, such as different amides and alkoxides, though the purification of the desired organoaluminates is complicated by the low volatility of the Me_2_Al(L) byproducts (L = N(SiHMe_2_)_2_, OCH_2_^t^Bu).^692^ Additionally, synthesis of **266-Y***via* direct salt elimination reaction between YCl_3_ and Li(AlMe_4_) has also been reported (Scheme 74, **C**).^692^ Alternatively, homoleptic trimethyl precursors [Y(Me)_3_]_*n*_ (**226-Y**) and [Lu(Me)_3_]_*n*_ (**226-Lu**) can be converted into the corresponding organoaluminates by adduct formation with AlMe_3_ (Scheme 74, **D**).^631,692^

**Scheme 77:**
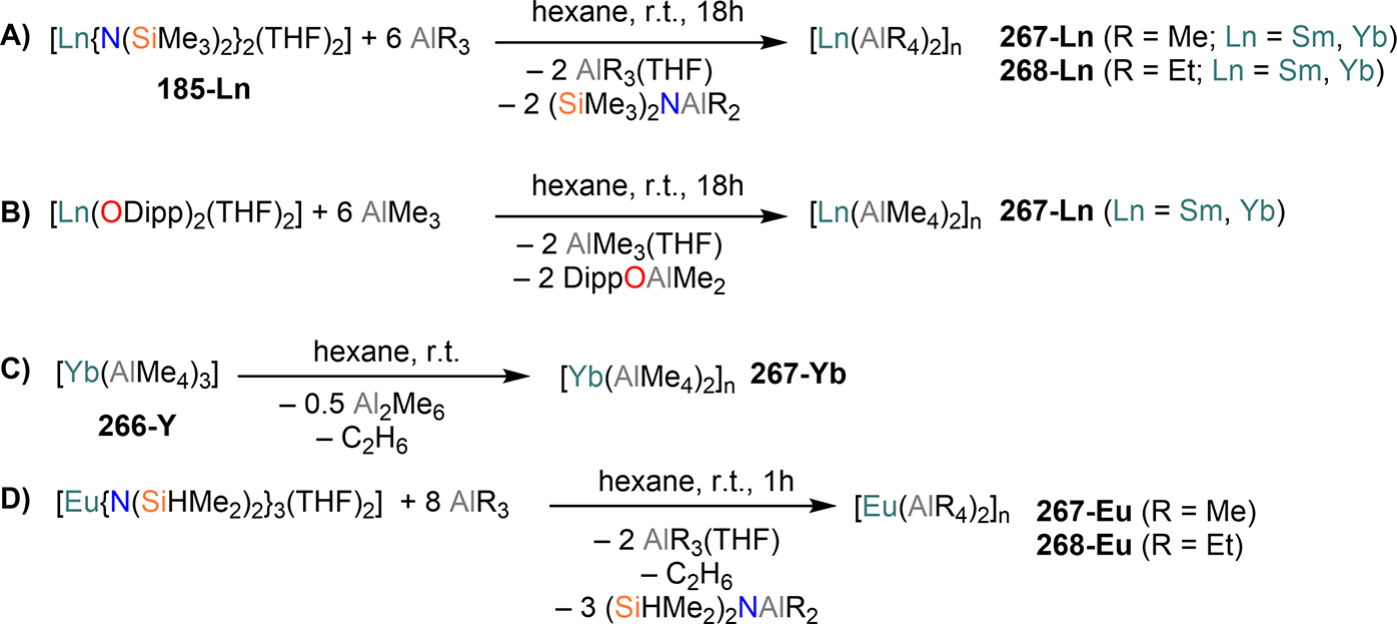
Synthesis of Divalent Alkylaluminates^518,696^

Amide-alkyl or alkoxide-alkyl exchange reactions can be used to access divalent Ln organoaluminates [Ln(AlMe_4_)_2_]_*n*_ (**267-Ln**; Ln = Sm, Yb) and [Ln(AlEt_4_)_2_]_*n*_ (**268-Ln**; Ln = Sm, Yb);^556,696^ these are achieved by reacting *bis*-silylamide precursors **185-Sm** and **185-Yb** (Scheme 77, **A**) or *bis-*alkoxides Ln(ODipp)_2_(THF)_*x*_ with AlR_3_ (R = Me, Et) (Scheme 77, **B**).^556,601,696^ Interestingly, **267-Ln** and **268-Ln** do not degrade in the presence of donors or polar solvents, and the solid-state structures of THF adducts [Ln(AlEt_4_)_2_(THF)_2_] (**269-Ln**·2THF; Ln = Sm, Yb) have also been reported.^696^**267-Yb** can also be obtained *via* thermally induced self-reduction of **266-Yb**, with concomitant formation of Al_2_Me_6_ and C_2_H_6_ (Scheme 77, **C**).^518^ Finally, Eu(III) *tris-*amide **186-Eu**·2THF can be converted into [Eu(AlR_4_)_2_]_*n*_ (**267-Eu** – R = Me; **268-Eu** – R = Et) upon treatment with an excess of AlR_3_ (Scheme 77, **D**).^518^

**Scheme 78:**
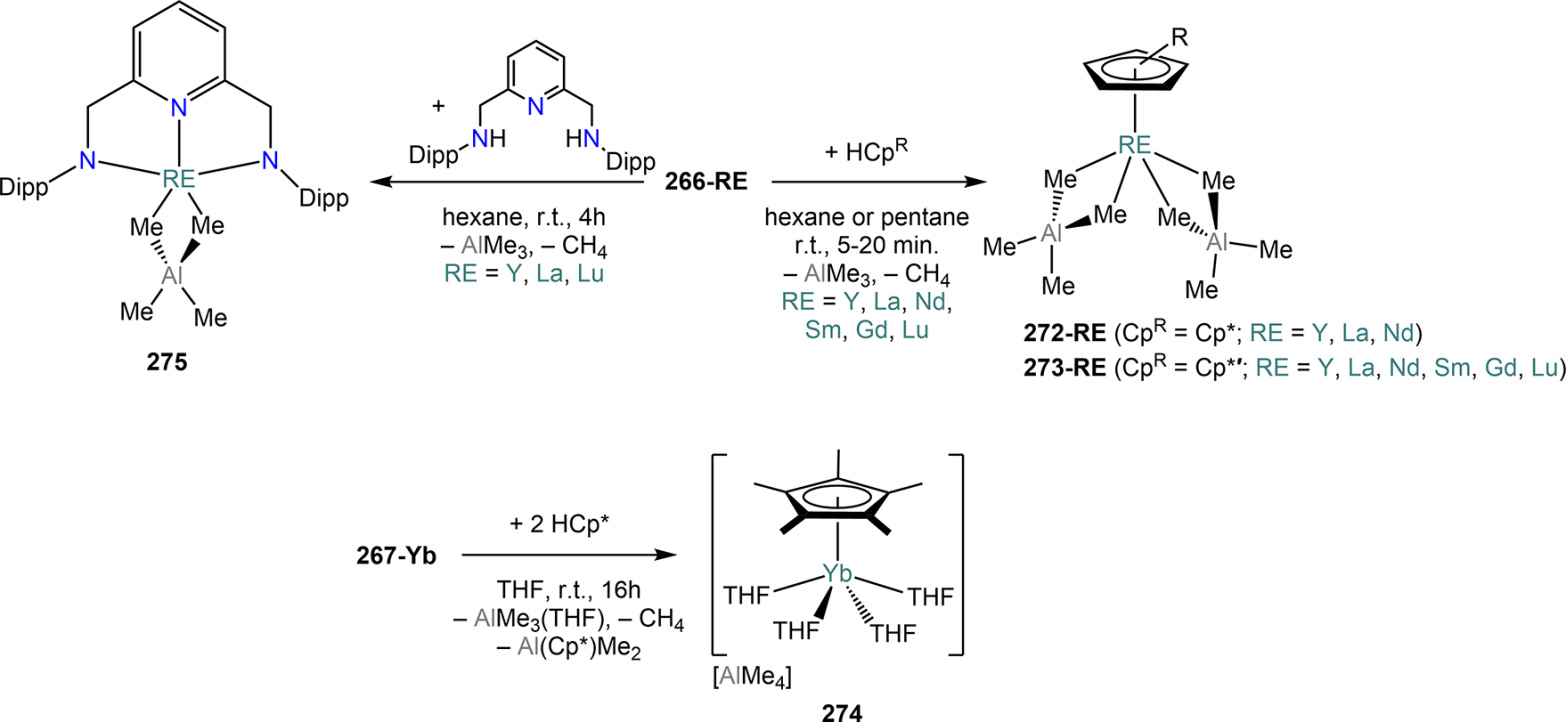
Selected Examples of Protonolysis Reactivity of **266-RE** and **267-Yb**(697−700)

RE organoaluminates (Table 12) are excellent synthetic precursors in protonolysis reactivity with a range of substrates. Anwander demonstrated this with the preparation of Cp derivatives [RE(Cp^R^)(AlMe_4_)_2_] (**272-RE**; Cp^R^ = Cp*, RE = Y, La, Nd, and Lu; **273-RE**; Cp^R^ = Cp*′; RE = Y, La, Nd, Sm, Gd, and Lu) *via* deprotonation of the corresponding cyclopentadiene in hexane or pentane under mild conditions (Scheme 78).^697,698^ Lappert and co-workers first showed that heteroleptic Cp-alkylaluminate complexes can undergo donor-induced cleavage of AlMe_3_ upon treatment with a Lewis base.^695^ In a similar vein, **272-RE** can be converted to the “unmasked” alkyl analogues [RE(Cp*)(Me)_2_]_3_ (RE = Y, Lu) by addition of THF; the reaction is fully reversible, and **272-RE** is reobtained *via* donor addition reaction with AlMe_3_ at −35 °C.^698^ Furthermore, the difference in the bonding character between divalent Ln and trivalent RE aluminates is exemplified by their divergent reactivity with cyclopentadienyls. When divalent **267-Yb** is reacted with HCp*, the {AlMe_4_}^−^ ligand acts as a weakly coordinating anion and the separated ion pair complex [Yb(Cp*)(THF)_4_][AlMe_4_] (**274**) is isolated from the reaction.^556^ Interestingly, complex **274** is obtained even when two equivalents of HCp* are used in the reaction, and the authors detected unreacted pentamethylcyclopentadiene in the reaction mixture, together with AlMe_3_(THF) and traces of Cp*AlMe_2_.^556^ Zimmermann *et al.* have also shown that pro-ligand C_6_NH_3_[CH_2_NH(Dipp)]_2_-2,6 can be deprotonated by **266-RE** (RE = Y, La, Lu), yielding heteroleptic aluminate complex [RE(BDPPpyr)(AlMe_4_)] (**275**; BDPPpyr = {C_6_NH_3_[CH_2_N(Dipp)]_2_-2,6)}^−^) (Scheme 78).^699^

**Scheme 79:**
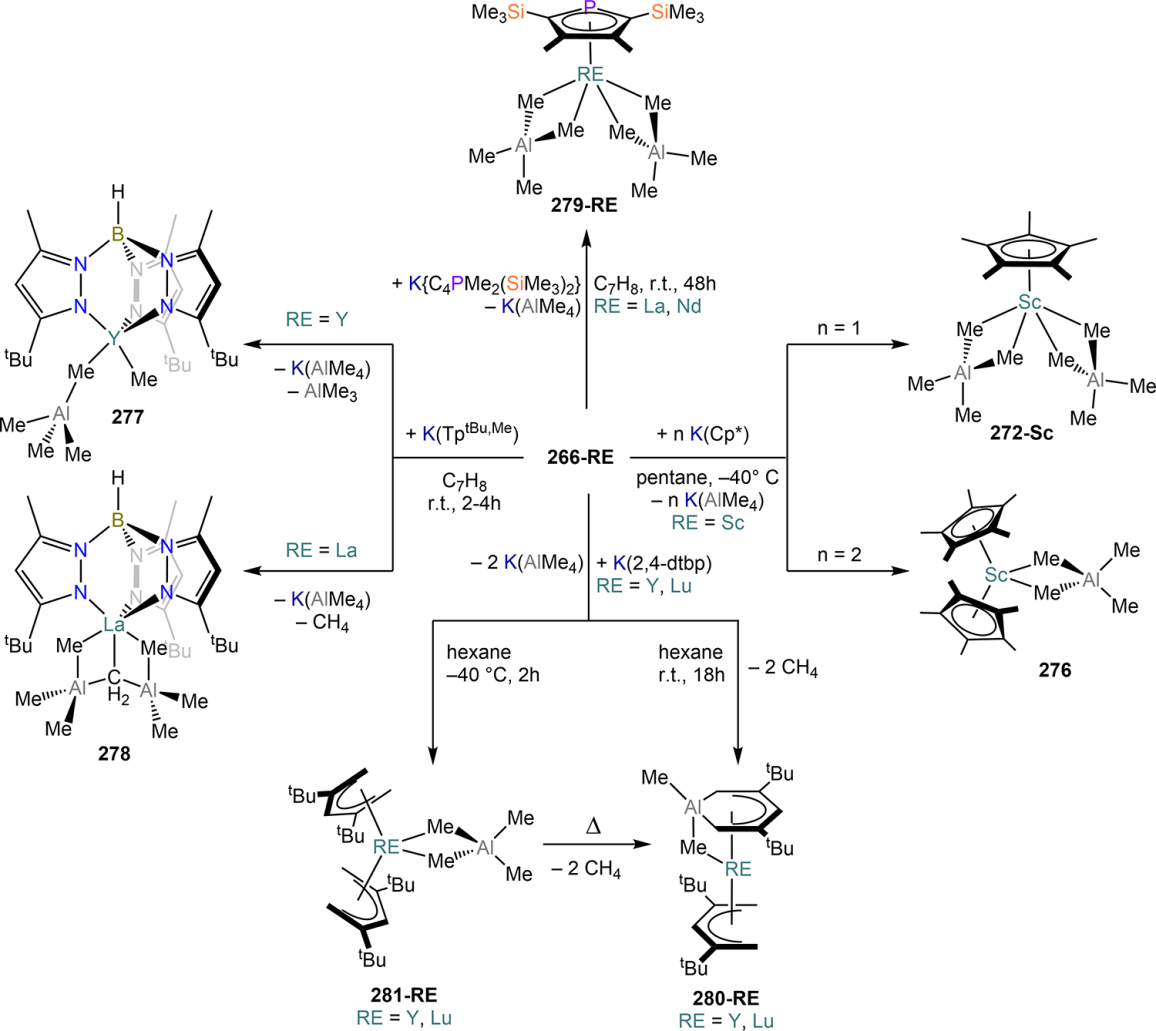
Selected Examples of Salt Elimination Reactivity of **266-RE**(701−703)

Anwander and co-workers have also demonstrated that RE organoaluminates can be employed for salt elimination reactivity with various ligand transfer reagents.^11^ Piano-stool derivative **272-Sc** is obtained from the 1:1 reaction between K(Cp*) and **266-Sc**, while the metallocene complex [Sc(Cp*)_2_(AlMe_4_)] (**276**) is obtained when two equivalents of K(Cp*) are used (Scheme 79).^633^ When K(Tp^tBu,Me^) is reacted with **266-Y**, K(AlMe_3_)_4_ is eliminated together with one equivalent of AlMe_3_ and the mixed alkyaluminate/alkyl complex [Y(Tp^tBu,Me^)(AlMe_4_)(Me)] (**277**) is isolated (Scheme 79),^701^ which can also be obtained *via* protonolysis reactivity between HTp^tBu,Me^ and **266-Y**.^700^ However, when **266-La** is used as a starting material, the reaction produces the “Tebbe-like” methylidene complex [La(Tp^tBu,Me^)(μ_3_-CH_2_)(AlMe_3_)_2_] (**278**) (Scheme 79).^701^ Salt elimination protocols were also employed by Le Roux *et al.* to produce the piano-stool phospholyl complex [RE{C_4_PMe_2_(SiMe_3_)_2_}(AlMe_4_)_2_] (**279-RE**; RE = La, Nd) (Scheme 79).^702^ Another example of the interesting reactivity of RE organoaluminates has been shown recently by Barisic *et al.* with the salt metathesis reaction between **266-RE** (RE = Y, Lu) and two equivalents of K(2,4-dtbp) (2,4-dtbp = {CH_2_C(^t^Bu)CHC(^t^Bu)CH_2_}^−^). At room temperature the reaction affords the aluminabenzene complexes [RE(2,4-dtbp)(C_5_H_3_AlMe-1-^t^Bu_2_-3,5)] (**280-RE**; RE = Y, Lu) (Scheme 75).^703^ However, when the same reaction is carried out at −40 °C, the *pseudo*-metallocenes [RE(2,4-dtbp)_2_(AlMe_4_)] (**281-RE**; RE = Y, Lu) are obtained instead, which can undergo thermal decomposition to give aluminabenzene derivatives **280-RE**.^703^

## 10 Conclusions and Future Perspectives

The landscape of RE and Ln synthetic chemistry has grown immensely since the first adventurous steps taken more than half a century ago by various pioneers of the discipline. This impressive growth has been supported by the enormous expansion of the RE synthetic toolbox and the opening of a myriad of synthetic possibilities, the potential of which is still far from being fulfilled. Nonetheless, there is still progress to be made in the development of synthetic precursors and methodologies applicable to divalent Lns outside the Sm, Eu, and Yb triad, which is exemplified by the relatively small number of synthetic applications compared to the trivalent counterparts.^42^ Similarly, the landscape of molecular Ln(IV) chemistry—historically limited to Ce(IV)—has been recently expanded with the stabilization of the first molecular Pr(IV) and Tb(IV) species;^708−713^ as a result, new advances will likely emerge for the preparation of specific starting materials and methodologies applicable to tetravalent Ln chemistry. Recently, Evans and Daly have demonstrated that mechanochemical synthesis can be used for the preparation of RE derivatives using salt elimination methodologies, thus offering a viable alternative to standard solution methods.^714−716^ Additionally, direct activation of metals is a synthetic technique that is still used by only a few groups around the world, largely because of the historic involvement of toxic Hg and Tl reagents; however, these methods have now been extended to more benign metals (Bi and Ag),^175^ which should encourage research teams to incorporate these methods into their synthetic repertoire. Many of the limitations that historically frustrated the progress of RE synthetic chemistry are gradually being challenged and overcome; an example of this is the successful stabilization and identification of the once elusive “RE(Me)_3_” species by Anwander and co-workers,^504,631,633^ though this still has not been achieved with the larger Lns.^717^ Finally, several of the synthetic precursors presented in this work have emerged over the past decade; therefore, because of these recent discoveries, there will certainly be many new and exciting synthetic avenues that will be opened-up in the years to come.

## Abbreviations

Ad: adamantyl
AE: alkaline Earth
An: actinide
Ar: aryl
BDI: β-diketiminate
BDPPpyr: [C_6_NH_3_{CH_2_N(Dipp)}_2_-2,6)]^−^
BIPM-H_2_: bis(iminophosphorano)methane
BIPM-H: bis(iminophosphorano)methanide
BIPM-: bis(iminophosphorano)methanediide
bipy: 2,2′-bipyridine
Bn: benzyl, {CH_2_Ph}^−^
Bn^Me^: {CH_2_(C_6_H_4_Me-4)}^−^
Bn^tBu^: {CH_2_(C_6_H_4_^t^Bu-4)}^−^
Bn^NMe2^: {CH_2_(C_6_H_4_(NMe_2_)-2)}^−^
CAN: ceric ammonium nitrate
Carb: carbazolide, {C_12_H_8_N}^−^
COT: cyclooctatetraenyl, {C_8_H_8_}^−^
Cp: cyclopentadienyl, {C_5_H_5_}^−^
Cp^BIG^: {C_5_Ar_5_}^−^ (Ar = C_6_H_4_R-4; R = Et, ^i^Pr, ^n^Bu)
Cp^iPr4^: {C_5_H^i^Pr_4_}^−^
Cp^iPr5^: {C_5_^i^Pr_5_}^−^
Cp^Ph5^: {C_5_Ph_5_}^−^
Cp′: {C_5_H_4_SiMe_3_}^−^
Cp″: {C_5_H_3_(SiMe_3_)_2_}^−^
Cp‴: {C_5_H_2_(SiMe_3_)_3_}^−^
Cp*: {C_5_Me_5_}^−^
Cp^tet^: {C_5_HMe_4_}^−^
Cp^t^: {C_5_H_4_^t^Bu}^−^
Cp^tt^: {C_5_H_3_^t^Bu_2_}^−^
Cp^ttt^: {C_5_H_2_^t^Bu_3_}^−^
DF: 2,6-difluorophenyl, C_6_H_3_F_2_-2,6
Db: 2,6-di-*tert*-butylphenyl, C_6_H_3_^t^Bu_2_-2,6
DBA: *N,N*-dimethylbenzylamine
Dbmp: 2,6-di-*tert*-butyl-4-methylphenyl, C_6_H_2_^t^Bu_2_-2,6-Me-4
Dipp: 2,6-di-*iso*-propylphenyl, C_6_H_3_^i^Pr_2_-2,6
Dipp-Bian: 1,2-bis[(2,6-diisopropylphenyl)imino]acenaphthene
Dmp: C_6_H_3_Mes_2_-2,6
dpa: 2,2′-dipyridylamide, {(C_6_H_4_N)_2_N}^−^
Dpp: 2,6-diphenylphenyl, C_6_H_3_Ph_2_-2,6
2,4-dtbp: {CH_2_C(^t^Bu)CHC(^t^Bu)CH_2_}^−^)
EPR: electron paramagnetic resonance
Form: formamidinate
HMPA: hexamethylphoshoramide
Htp: {C_4_H_2_P^t^Bu_2_-2,5}^−^
IDipp: 1,3-bis(Dipp)_2_imidazolin-2-ylidene
IMe_2_: 1,3-bis(Me)_2_imidazolin-2-ylidene
IMes: 1,3-bis(Mes)_2_imidazolin-2-ylidene
Ln: lanthanoid (La–Lu)
Mes: 2,4,6-trimethylphenyl
MVS: metal vapor synthesis
OTf: triflate, {O_3_SCF_3_}^−^
Ph_2_pz: {3,5-Ph_2_C_3_HN_2_}^−^
Phen: phenantroline
Phpip: *N-*phenylpiperazine
pyr: pyrrolide, {C_4_H_4_N}^−^
pz: pyrazolate, {C_3_H_3_N_2_}^−^
RE: rare earth (Sc, Y, La–Lu)
RT: redox transmetalation
RTP: redox transmetalation protononlysis/protolysis
THF: tetrahydrofuran
TM: transition metal
TMG: tetramethylguanidinate
Tph: C_6_H_4_Tripp-2
Tripp: 2,4,6-triisopropylphenyl
